# Radical–carbanion relay enabled by earth-abundant titanium: carboxylation of 1°, 2°, and 3° benzyl chlorides with CO_2_

**DOI:** 10.1039/d6sc04813g

**Published:** 2026-08-25

**Authors:** Lei Fan, Jiwu Zhao, Huaming Sun, Weiqiang Zhang, Guofang Zhang, Yajun Jian, Ziwei Gao

**Affiliations:** a Key Laboratory of Applied Surface and Colloid Chemistry, MOE, Xi'an Key Laboratory of Organometallic Material Chemistry, School of Chemistry and Chemical Engineering, Shaanxi Normal University Xi'an, 710119 P. R. China zwgao@snnu.edu.cn yajunjian@snnu.edu.cn +86-29-81530727; b School of Chemistry & Chemical Engineering, Xinjiang Normal University Urumqi 830054 P. R. China; c College of Chemistry & Chemical Engineering, Yan'an University, Research Institute of Comprehensive Energy Industry Technology Yan'an 716000 P. R. China

## Abstract

The catalytic conversion of CO_2_ into arylacetic acids is of significant interest owing to the prevalence of these scaffolds in nonsteroidal anti-inflammatory drugs (NSAIDs) and fine chemicals. However, existing methods remain constrained by limited substrate scope, reliance on precious metals, or the need for stoichiometric additives. Herein, we report an earth-abundant titanium(iii)-catalyzed reductive carboxylation of benzyl halides with CO_2_ that operates *via* a radical–carbanion relay mechanism. This protocol accommodates primary, secondary, and tertiary C(sp^3^)–Cl bonds, benzyl bromides, and allylic chlorides, and proceeds without magnesium salt additives under oxygen-tolerant conditions. The synthetic utility is demonstrated by gram-scale reactions and the direct one-step synthesis of several NSAIDs. Mechanistic studies combining radical trapping, deuterium-labeling, intermediate characterization, and DFT calculations establish that Ti(iii) promotes inner-sphere chlorine-atom abstraction to generate a benzyl radical, which is captured, reduced to a benzyl anion, and subsequently trapped by CO_2_, thereby circumventing the prohibitive direct activation of CO_2_ at the metal center.

## Abstract 2

A Ti(iii)-catalyzed reductive carboxylation of 1°, 2°, and 3° benzyl chlorides with CO_2_ proceeds *via* a radical-carbanion relay, enabling the additive-free synthesis of arylacetic acid drugs under O_2_-tolerant conditions without precious metals.

## Graphical abstract

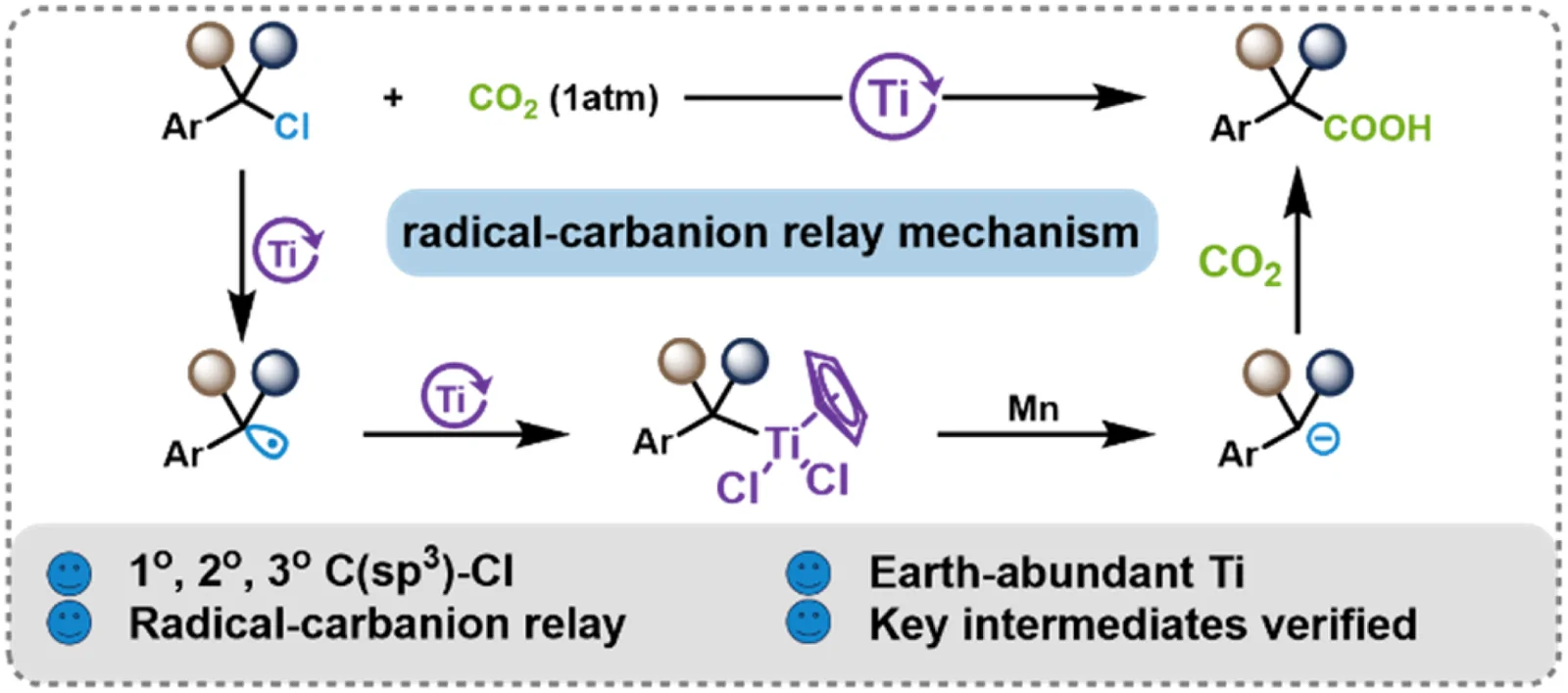

## Introduction

Carbon dioxide (CO_2_), as an abundant, inexpensive, and atom-economical C1 synthon,^1–17^ holds significant scientific and practical importance for advancing the sustainable utilization of carbon resources through high-value transformations in organic synthesis. In this context, the construction of arylacetic acid scaffolds using CO_2_ is particularly valuable.^18–26^ Arylacetic acids not only serve as key pharmacophores in numerous nonsteroidal anti-inflammatory drugs (NSAIDs, Fig. 1) such as naproxen,^27^ flurbiprofen,^28,29^ ibuprofen,^30^ and diclofenac,^31^ but also constitute indispensable core intermediates in fine chemical manufacturing.^32,33^ Benzyl halides, owing to their low cost, ready availability, and favorable reactivity, are considered ideal electrophilic precursors for the assembly of arylacetic acid frameworks.^34,35^ Transition metal-catalyzed carboxylation has been extensively explored for both unsaturated and saturated substrates;^36^ in this context, the development of reductive cross-coupling reactions between benzyl halides and CO_2_ for the targeted, high-value conversion of CO_2_ has emerged as a focal point of contemporary synthetic methodology.

**Fig. 1:**
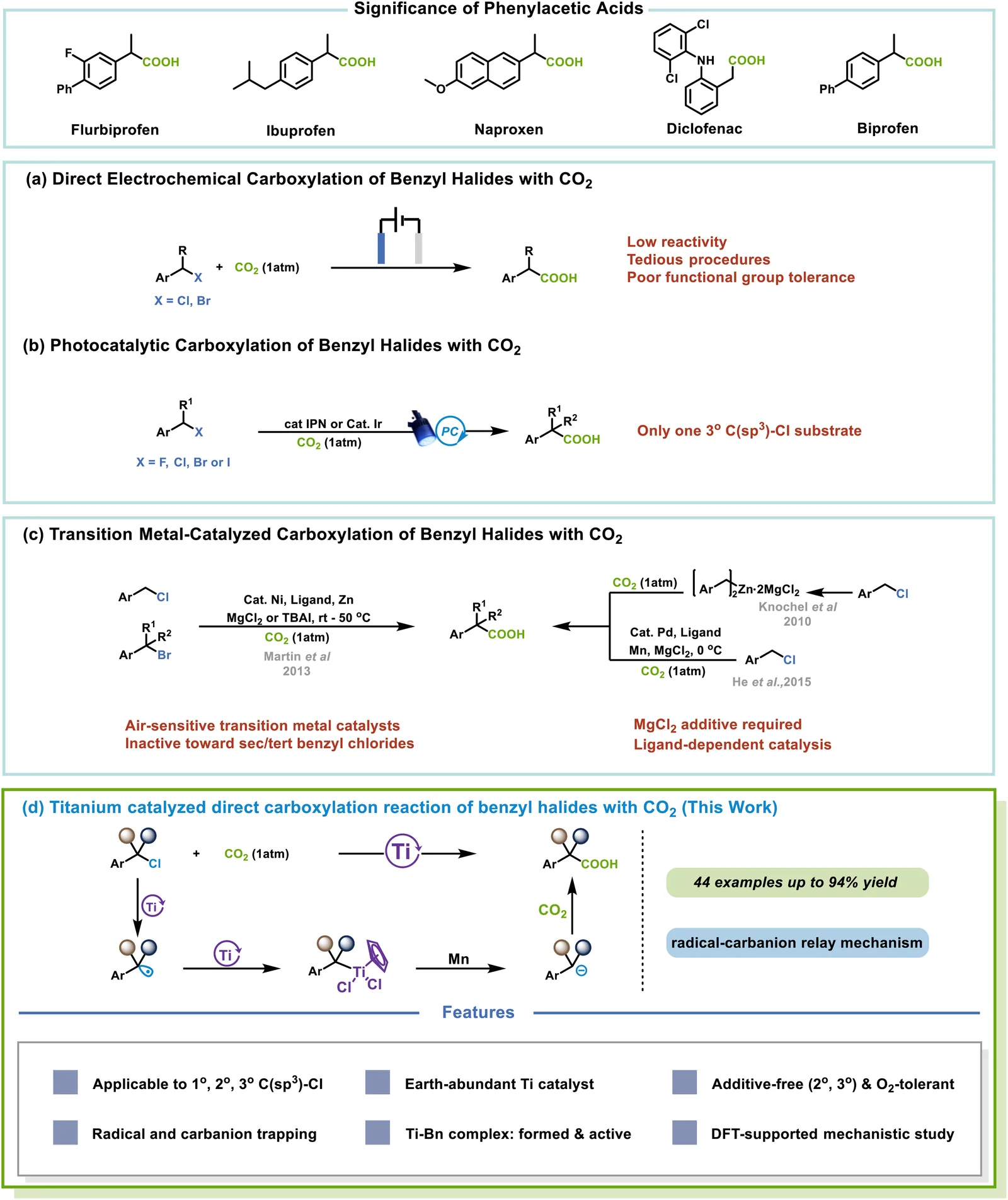
Overview of the carboxylation of benzyl halides using CO_2_ as a C1 synthon.

Currently, methods for the carboxylation of benzyl halides with CO_2_ to afford arylacetic acids can be broadly classified into three strategic categories (Fig. 1): transition-metal catalysis,^37–39^ photocatalysis,^40–42^ and electrocatalysis.^43–47^ Although electrochemical approaches enable the direct carboxylation of benzyl halides with CO_2_, they are often constrained by the use of sacrificial anodes, precious metal electrodes, and high concentrations of supporting electrolytes (Fig. 1a). A recent Ni-catalyzed electrochemical system avoids sacrificial anodes, yet has not been demonstrated for benzyl chlorides.^48^ Photocatalytic approaches, while mild and operationally simple, have been extensively reviewed recently with a focus on free-radical processes,^49^ still face narrow substrate scope. A visible-light photoredox-catalyzed carboxylation of C(sp^3^)–F bonds with CO_2_ was reported in 2021, wherein CO_2_ served a dual role as both electron carrier and electrophile.^50^ Subsequent work extended this strategy to benzyl chlorides and bromides (Yu,^40^ 2022) and to benzyl bromide (Xi,^41^ 2023). A halogen atom transfer strategy further accommodated benzyl fluorides and iodides (Masaoka,^42^ 2025), and a photocatalytic defluorocarboxylation using formate as both reductant and CO_2_ source gave arylacetic acids such as flurbiprofen.^51^ Despite these advances, tertiary substrates remain either unreported or limited to a single example with moderate efficiency (Fig. 1b). In a complementary approach, the boryl radical activation of benzylic C–OH bonds for cross-electrophile coupling with CO_2_ was also demonstrated, further expanding the scope of photoredox-driven carboxylation.^52^ In parallel, transition-metal catalysis has also advanced significantly. Classical routes employ stoichiometric Grignard or organozinc reagents, which suffer from limited functional group tolerance, low reactivity, and tedious anhydrous manipulations.^37^ To overcome these drawbacks, catalytic systems have been developed, as exemplified by Martin's nickel-catalyzed reductive carboxylation (2013)^38^ and He's palladium-catalyzed system (2015) (Fig. 1c).^39^ Nevertheless, these methods still face challenges: limited substrate scope (especially for benzyl chlorides), air/moisture sensitivity, and reliance on external ligands or stoichiometric additives (*e.g.*, MgCl_2_). Collectively, these three complementary strategies each offer distinct mechanistic insights, but earth-abundant metal catalysis, with its low cost and resource advantages, holds particular promise for surmounting existing limitations.

Titanium (Ti), an earth-abundant early transition metal, features low toxicity, low cost,^53^ and readily accessible oxidation states (commonly +2, +3, +4).^54^ The potential of titanium complexes in CO_2_ functionalization has been widely recognized;^55–57^ however, existing systems mostly rely on stoichiometric low-valent titanium reagents (*e.g.*, Ti(ii) complexes) or highly reactive titanium hydride species to directly activate CO_2_, making the realization of a catalytic cycle extremely challenging, whereas the present work exploits the single-electron reducing ability of trivalent titanium to activate organic halides rather than CO_2_ itself. This strategy benefits from three key attributes of the titanium center: a suitable Ti(iii)/(iv) reduction potential for efficient radical generation^58–60^—a strategy that has recently been comprehensively reviewed in the context of photocatalytic activation of unactivated alkyl chlorides *via* single-electron transfer,^61^ strong oxophilicity that stabilizes oxygen-containing inter-mediates,^62–64^ and the tunability of electronic/steric properties through ligand and oxidation-state modulation to optimize electron transfer and radical trapping.^65–68^ On the basis of these characteristics, we report a trivalent titanium-mediated carboxylation of benzyl halides with CO_2_ that proceeds *via* sequential halide activation, radical relay, carbanion generation, and nucleophilic CO_2_ capture, thereby circumventing the difficulty of direct metal-centered CO_2_ activation. This protocol accommodates primary, secondary, and tertiary C(sp^3^)–Cl bonds and extends to benzyl bromides and allylic chlorides, offering a step- and atom-economical route to high-value arylacetic acids from inexpensive bulk feedstocks (Fig. 1d).

## Results and discussion

We commenced our studies by optimizing the reaction conditions for the titanium-catalyzed carboxylation of benzyl halides with CO_2_. Given that the steric hindrance and radical reactivity of secondary benzyl chlorides are intermediate between those of primary and tertiary substrates, (1-chloroethyl)benzene (1a) was selected as a representative model substrate (Table 1). Through systematic evaluation of a series of reaction parameters, the optimal conditions were identified as follows: DMF as the solvent, CpTiCl_3_ (10 mol%) as the catalyst, Mn (6.0 equiv.) as the reductant, and a reaction temperature of 40 °C for 12 hours. Under these conditions, the desired product 2a was obtained in 87% yield (entry 1). Steric effects play a dominant role in this titanium-catalyzed system: the more sterically encumbered Cp_2_TiCl_2_ failed to catalyze the reaction (entry 2), while the electron-richer Cp*_2_TiCl_2_ afforded the product in only 8% yield (entry 3). Cp*TiCl_2_ [*E*_1/2_(iv/iii) = −1.12 V], which possesses a stronger reducing capability than CpTiCl_3_ [*E*_1/2_(iv/iii) = −0.79 V],^59^ exhibited slightly improved activity but still gave unsatisfactory results (25%, entry 4). Notably, replacing the reductant with Zn resulted in complete loss of activity (entry 5), which may be attributed to the stronger reducing power of Mn relative to Zn. In contrast, reducing the Mn loading from 6.0 to 4.0 equivalents led to only a slight decrease in yield, with 2a still obtained in a good 76% yield (entry 6). Although the combination of Cp*TiCl_3_ and Zn has proven effective in related radical alkylations of tertiary chlorides involving trivalent titanium species,^59^ the present system involves a second electron-transfer step wherein a reductant is required to reduce an intermediate adduct. Consequently, the combination of CpTiCl_3_, which possesses a relatively positive reduction potential, with the stronger reductant Mn may be kinetically more favorable for driving the reaction forward. Furthermore, screening of alternative solvents and reaction temperatures led to diminished yields (entries 7–10). Control experiments confirmed that Mn, the titanium catalyst, and CO_2_ are all indispensable components for this transformation (entries 11–13).

**Table 1:** Optimization of the reaction conditions^a^

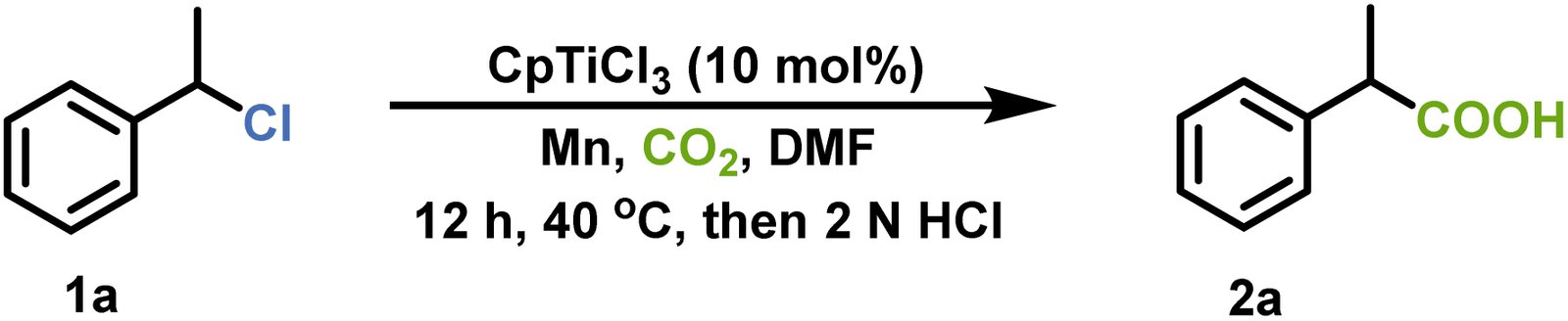		
Entry	Variations	2a (%)^b^
1	None	87
2	Cp_2_TiCl_2_ instead of CpTiCl_3_	Trace
3	Cp*_2_TiCl_2_ instead of CpTiCl_3_	8
4	Cp*TiCl_3_ instead of CpTiCl_3_	25
5	Zn instead of Mn	Trace
6	Mn(4.0 equiv.) instead of Mn(6.0 equiv.)	76
7	DMSO instead of DMF	0
8	DMA instead of DMF	29
9	0 °C instead of 40 °C	7
10	80 °C instead of 40 °C	25
11	Without Mn	0
12	Without CpTiCl_3_	0
13	Without CO_2_	0

a Reaction conditions: 1a (0.5 mmol), Mn (6.0 equiv.), CpTiCl_3_ (10 mol%), in DMF (2.5 mL) at 40 °C in a CO_2_ atmosphere for 12 h.

b ^1^H NMR yields using CH_2_Br_2_ as an internal standard. DMF = *N*,*N*-dimethylformamide. DMSO = dimethyl sulfoxide. DMA = dimethylacetamide.

After establishing the optimal reaction conditions, the substrate scope was systematically investigated (Table 2). The reactivity of secondary benzyl chlorides was first evaluated (2a–2o). When the α-substituent of the substrate was a single aryl group, variations in the electronic nature of the substituents on the aromatic ring (2a–2e), the substitution position (2f–2h and 2i), or the number of substituents (2j) had no pronounced effect on the reactivity, and the target arylacetic acids were obtained in good yields in all cases. From an electronic perspective, substrates bearing electron-withdrawing groups (EWGs, *e.g.*, 2b–2d, 2f–2h) generally gave higher yields than those with electron-donating groups (EDGs, *e.g.*, 2e, 2j). When both α-substituents were aryl groups, the reaction efficiency was further improved (2k–2o); for instance, substrates 2k and 2l afforded the products in 92% and 94% yield, respectively. To probe the scope boundaries, 1-chloroadamantane, an unactivated tertiary alkyl chloride, gave no carboxylation product, consistent with its higher C–Cl bond dissociation energy (∼86 kcal mol^−1^*vs.* <70 kcal mol^−1^ for benzyl chlorides^69^). A tertiary benzyl chloride bearing a single phenyl group afforded the corresponding carboxylic acid in only 8% yield (2p), along with 74% of the homo-coupling product, indicating that the benzylic radical is formed but its further reduction to the carbanion is inefficient, and radical–radical coupling dominates. Notably, tertiary benzyl chlorides with significant steric hindrance (2q–2t) still furnished the corresponding carboxylation products in moderate yields under the present conditions. The structure of 2s was unambiguously confirmed by X-ray crystallography (CCDC 2556758). The only exception was the substrate bearing two strongly electron-donating methoxy groups (2t), which gave a yield of only 30%; this is tentatively attributed to the destabilization of the *in situ* generated alkyl radical intermediate caused by the strong electron-donating effect. More importantly, this catalytic system enabled the direct one-step synthesis of several clinically important nonsteroidal anti-inflammatory drug molecules in high yields, including naproxen (2u, 80%), flurbiprofen (2v, 86%), biprofen (2w, 84%), and ibuprofen (2x, 40%). The relatively lower yield for the ibuprofen substrate is presumably due to the additional steric hindrance of the α-isobutyl group and its adverse effect on the stability of the radical intermediate. Nevertheless, these results clearly demonstrate the promising potential of this catalytic system for the efficient construction of pharmaceutical scaffolds.

**Table 2:** Substrate scope of secondary and tertiary benzyl chlorides^a^

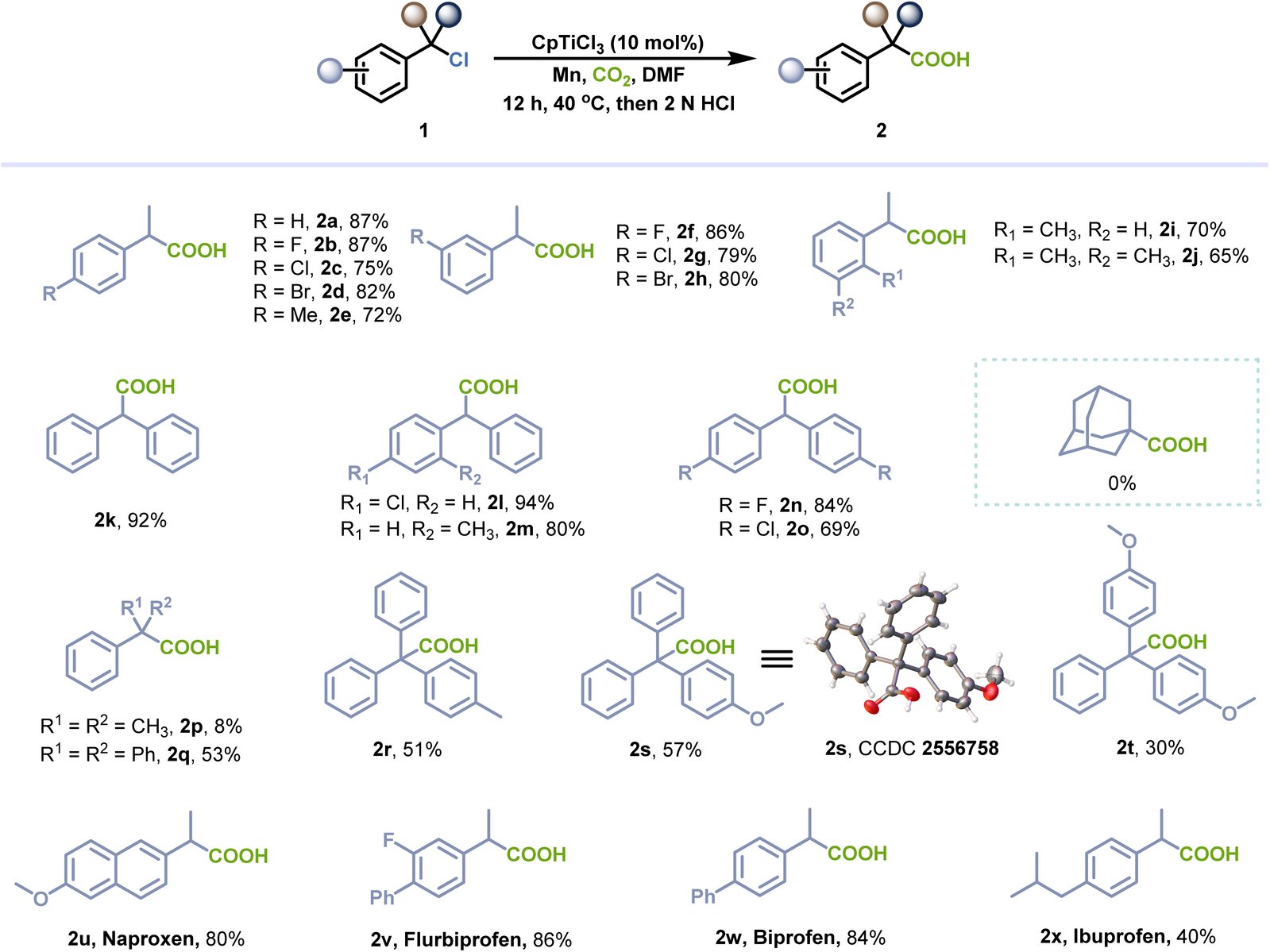

a Standard conditions as shown for Table 1, entry 1.

Following the successful extension of the protocol to secondary and tertiary benzyl chlorides, we turned our attention to primary benzyl chlorides (Table 3). Initial attempts under the standard conditions gave moderate results (*e.g.*, 4a, 58% yield), indicating that the less reactive primary substrates benefit from further optimization. Gratifyingly, a slight modification—addition of MgCl_2_ as a Lewis acidic additive and a reduced Mn loading—improved the yield of 4a to 76%. It should be noted that MgCl_2_ proved beneficial only for the less reactive primary benzyl chlorides; for secondary and tertiary substrates, the protocol operates without any external salt additives. The selective carboxylation of primary benzyl chlorides *via* a radical pathway is inherently challenging due to the limited stability of primary radical intermediates and their pronounced tendency to undergo unproductive side reactions. With the modified conditions, a variety of primary benzyl chlorides underwent smooth carboxylation to afford the desired arylacetic acids in moderate to good yields. Substrates bearing electronically diverse substituents at the *para*-position of the aromatic ring generally performed well (4a–4i), although certain cases gave relatively lower yields, such as *p*-fluoro (4b, 45%), *p*-chloro (4c, 41%), and *p*-methyl (4g, 40%) derivatives. Notably, substrates containing functional groups such as trifluoromethyl (4e), cyano (4f), and an ether linkage (4h) exhibited excellent compatibility, affording the corresponding carboxylic acids in 76%, 84%, and 62% yield, respectively. The trifluoromethyl and cyano groups, as strong electron-withdrawing substituents, can effectively stabilize the benzylic radical intermediate through polar effects, whereas the ethereal oxygen, despite being electron-donating, may contribute to radical stabilization *via* hyperconjugation of its lone pairs, thereby facilitating the carboxylation step. Furthermore, substrates bearing substituents at the *meta*- (4j–4m) or *ortho*-positions (4n–4p) also proved amenable to the reaction conditions. Substrates incorporating fused-ring (4q, 60%) and heteroaromatic (4r, 71%) motifs underwent smooth conversion to afford the target products in good yields. Even substrates bearing reduction-sensitive vinyl (4s) and ester (4t) groups were tolerated, furnishing the desired products in 52% and 56% yield, respectively, underscoring the good chemo-selectivity of this protocol. Notably, the method enabled the efficient synthesis of a key intermediate (4u) of the clinically important nonsteroidal anti-inflammatory drug loxoprofen in 71% yield. Benzyl bromides, as a class of readily accessible and stable electrophiles, also demonstrated viability within this catalytic manifold. A variety of mono- and poly-substituted benzyl bromides were smoothly converted to the corresponding arylacetic acid derivatives in moderate yields (4v–4y). However, compatibility with heterocyclic benzyl bromides remains relatively limited; for instance, substrate 4z afforded the target product in only 10% yield, indicating that the current conditions possess certain limitations with respect to this substrate class and warrant further optimization in future studies.

**Table 3:** Substrate scope of primary benzyl halides^a^

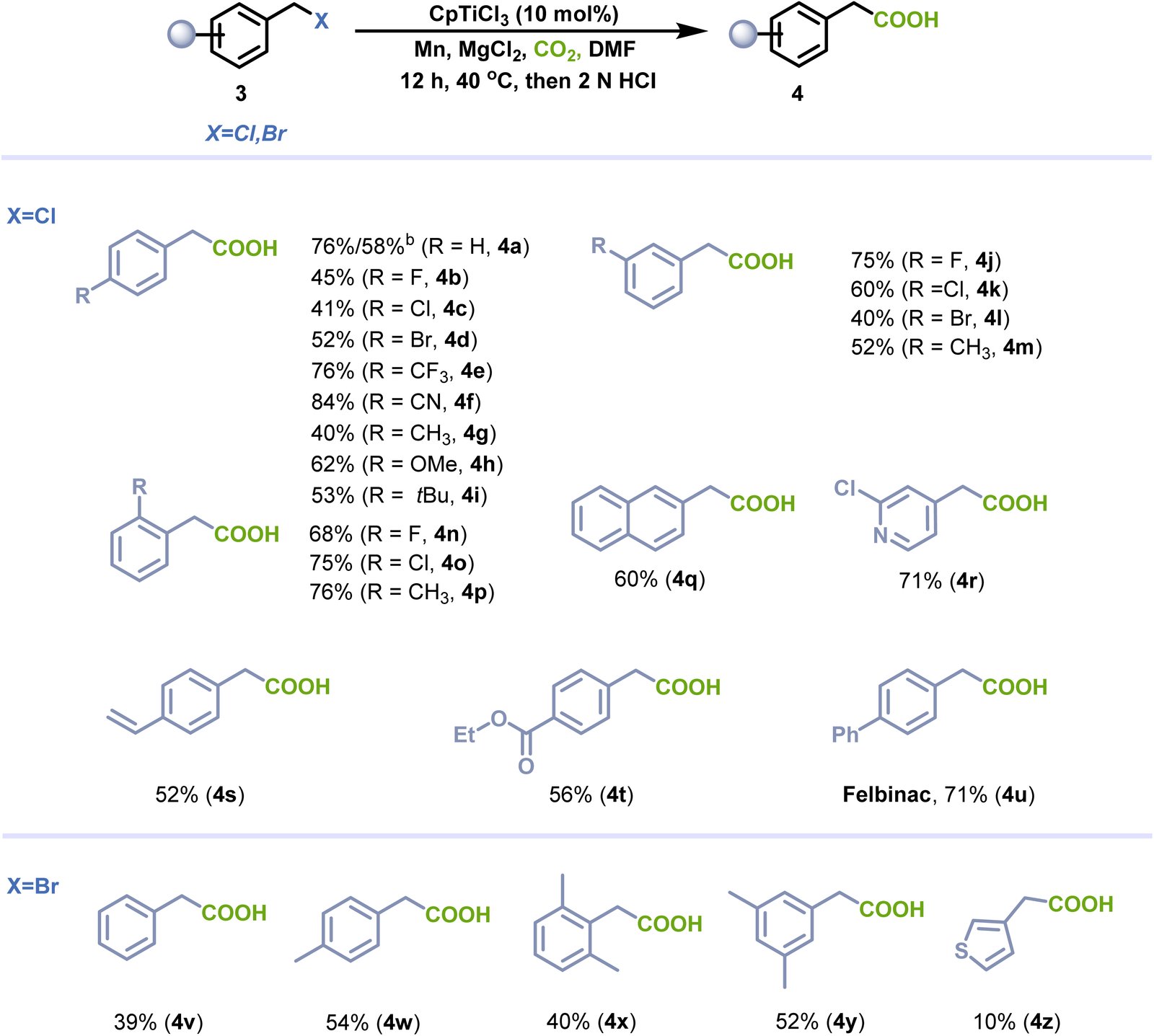

a Reaction conditions: 3a (0.5 mmol), Mn (4.0 equiv.), Cp*TiCl_3_ (10 mol%), MgCl_2_ (3.0 equiv.) in DMF (2.5 mL) at 40 °C in a CO_2_ atmosphere for 12 h.

b Standard conditions as shown in Table 1, entry 1.

The synthetic utility of the present protocol was further de-monstrated through a series of derivatization and scale-up experiments (Fig. 2). Allylic chloride 5 was converted to product 6a in 54% yield with 1 : 1 regioselectivity under the standard reaction conditions (eqn (1)). The tolerance of the reaction toward oxygen was assessed by introducing a controlled amount of O_2_ under the standard conditions, and the desired product 2a was still obtained in 70% yield (eqn (2)). Furthermore, the gram-scale synthesis of 2-phenylpropanoic acid 2a was readily achieved in 81% isolated yield (eqn (3)). Gratifyingly, the catalytic system was found to be capable of activating sp^2^ C–I bond as well: 4-iodoanisole underwent carboxylation to give the corresponding aryl carboxylic acid in 64% yield (eqn (4)). A one-pot procedure starting from substrate 1a, involving *in situ* carboxylation followed by methylation, afforded ester 7 in 62% overall yield (eqn (5)). Product 2k was smoothly transformed into the nicotinic receptor inhibitor adiphenine 8*via* a two-step nucleophilic substitution sequence (eqn (6)).

**Fig. 2:**
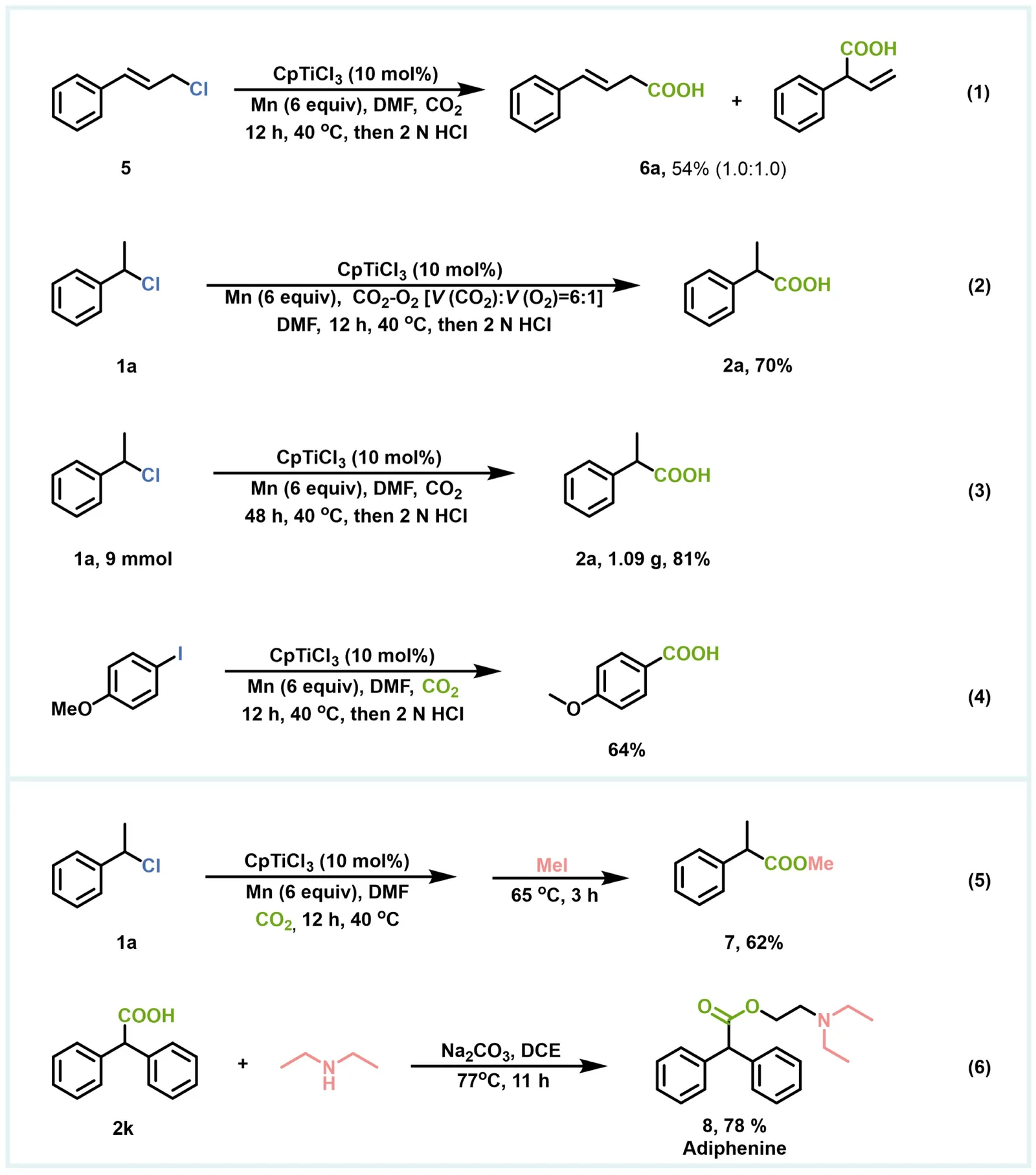
Synthetic applications and derivatization studies.

To elucidate the reaction mechanism, we conducted a series of control experiments (Fig. 3). Based on existing studies, two plausible catalytic cycles were initially considered (Fig. 5): (path a) sequential chlorine-atom abstraction by an *in situ* gen-erated Ti(iii) species to give a benzyl radical, followed by radical recombination with Ti(iii), reduction to a benzyl anion, and nucleophilic attack on CO_2_; or (path b) simultaneous activation of both the benzyl chloride and CO_2_ by Ti(iii) to generate a benzyl radical and a Ti–CO_2_ radical adduct, which then combine and are further reduced to the carboxylate. To distinguish between these alternatives, a series of mechanistic probes was performed. Radical trapping experiments were first carried out. Addition of TEMPO to the standard reaction afforded the TEMPO-adduct 9 in 11% yield, with no desired product being observed. The structure of 9 was confirmed by ^1^H NMR, ^13^C NMR, and high-resolution mass spectrometry (HRMS), which showed an [M + H]^+^ ion at *m*/*z* 262.2163 (calcd for C_16_H_28_NO^+^, 262.2165). When diphenyl diselenide was used, the phenylselenyl adduct 10 was detected in 13% yield, accompanied by a sharp decrease in the yield of 2a from 87% to 36% (Fig. 3a). In addition, to avoid the strong EPR signal of Mn^2+^ ions, a parallel experiment was performed using Zn as the reductant. The *in situ*-generated benzyl radical was trapped by DMPO, and EPR analysis revealed a characteristic signal for the α-methylbenzyl–DMPO adduct with *g* = 2.0186, *A*_N_ = 14.6 G, and *A*_H_ = 20.1 G, in good agreement with literature data.^70,71^ Collectively, these results confirm the intermediacy of a benzyl radical and strongly support a radical-mediated pathway. Isotopic labeling experiments were next performed under an Ar atmosphere with D_2_O as the deuterium source. The reductive dehalogenation product 11 was obtained with up to 97% deuterium incorporation (yields: 54%, 95%, and 97% at 3, 5, and 10 equivalents of D_2_O, respectively), providing compelling evidence for the formation of a benzyl anion intermediate (Fig. 3b). Additionally, a benzyl–titanium complex 12 was detected by high-resolution mass spectrometry (HRMS) in the reaction mixture (the methoxy group observed in this species originates from the methanol used as the solvent during the analysis, Fig. 3c), and an independently synthesized analogue 13 afforded the carboxylation product 4a in 24% yield under the standard conditions; a control experiment confirmed that Mn is essential for this conversion—no product was observed in its absence—supporting the intermediacy of a benzyl anion. To provide direct spectroscopic evidence for the Ti(iii) species, a parallel EPR experiment was conducted using Zn powder as the reductant, thereby avoiding interference from Mn^2+^ ions. A distinct Ti(iii) signal was observed (*g* = 1.9982; see Fig. S4 in SI), corroborating the formation of the low-valent titanium active species. Since the alternative path b does not involve a benzyl–titanium intermediate, it can be largely ruled out on the basis of these observations.

**Fig. 3:**
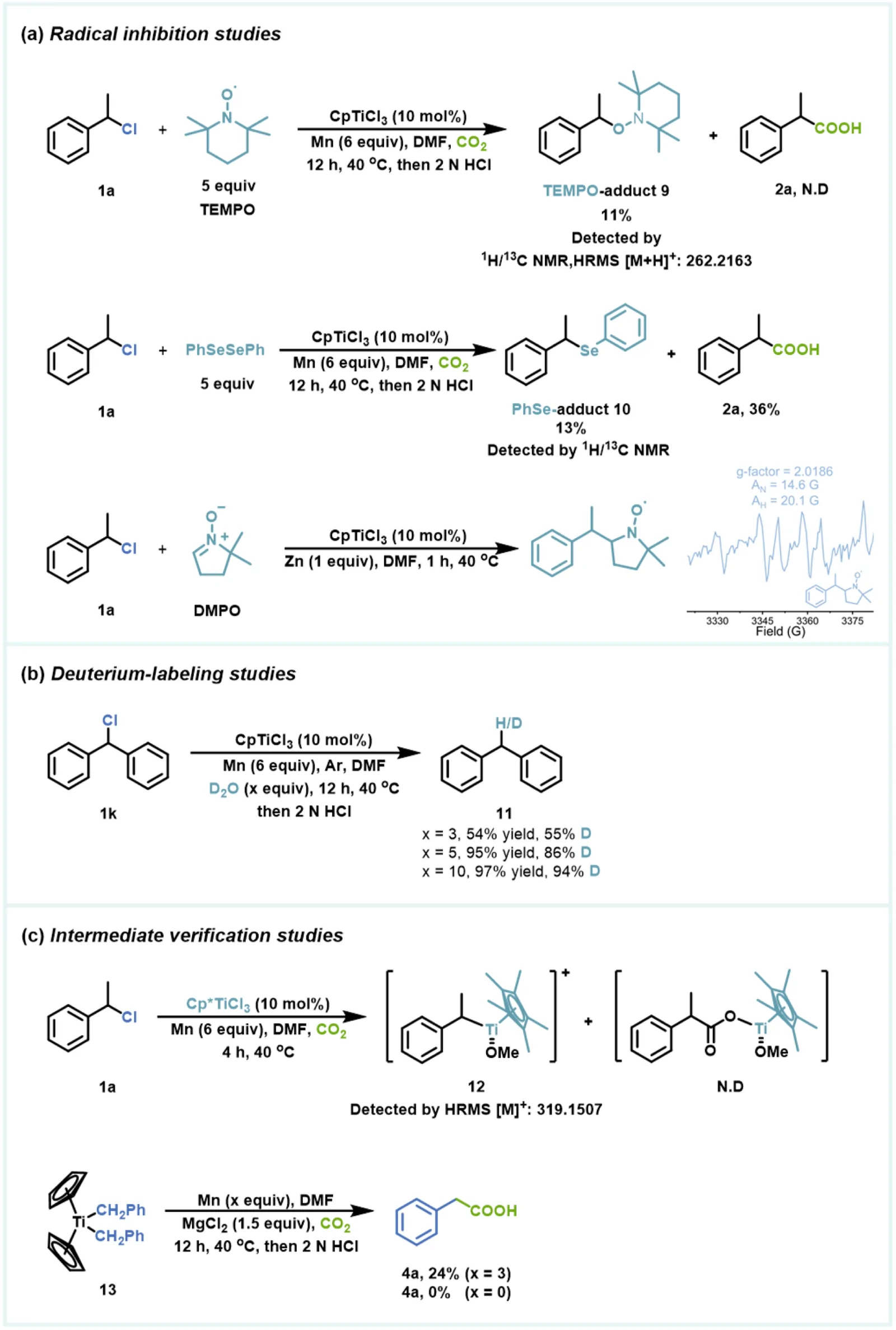
Control experiments for mechanistic investigation.

With experimental evidence supporting a radical-carbanion pathway, we next turned to density functional theory (DFT) calculations to better understand the energetic feasibility and detailed course of the reaction (Fig. 4).^72–74^ These studies examined both the C–Cl bond activation by Ti(III) and the alternative direct activation of CO_2_ (see SI for details). The computational results indicate that C–Cl bond activation by Ti(iii) is significantly more favorable than direct CO_2_ activation: the free energy barrier for generating CO_2_˙^−^*via* single-electron transfer (SET) is as high as 61.4 kcal mol^−1^ (path b), whereas chlorine atom abstraction by Ti(iii) through an inner-sphere mechanism requires a barrier of only 12.6 kcal mol^−1^ (path a). Specifically, CpTiCl_3_ is first reduced by Mn to CpTiCl_2_, which subsequently coordinates with substrate 1a to form intermedi-ate IM1. This intermediate then undergoes inner-sphere chlorine atom abstraction *via* transition state TS1, with an activation free energy of merely Δ*G*^‡^ = 4.9 kcal mol^−1^ relative to IM1. Spin density analysis of TS1 reveals a spin density of 0.14 on the secondary carbon atom and a significantly elongated C–Cl bond (*ca.* 2.36 Å) indicating that the transition state already possesses incipient benzyl radical character. Upon radical release, CpTiCl_3_ is regenerated, and the benzyl radical combines with another molecule of CpTiCl_2_ to afford intermediate IM3. Subsequent reduction of IM3 by Mn delivers the benzyl anion, which then undergoes nucleophilic attack on CO_2_ with a substantial thermodynamic driving force (Δ*G* = −20.1 kcal mol^−1^).

**Fig. 4:**
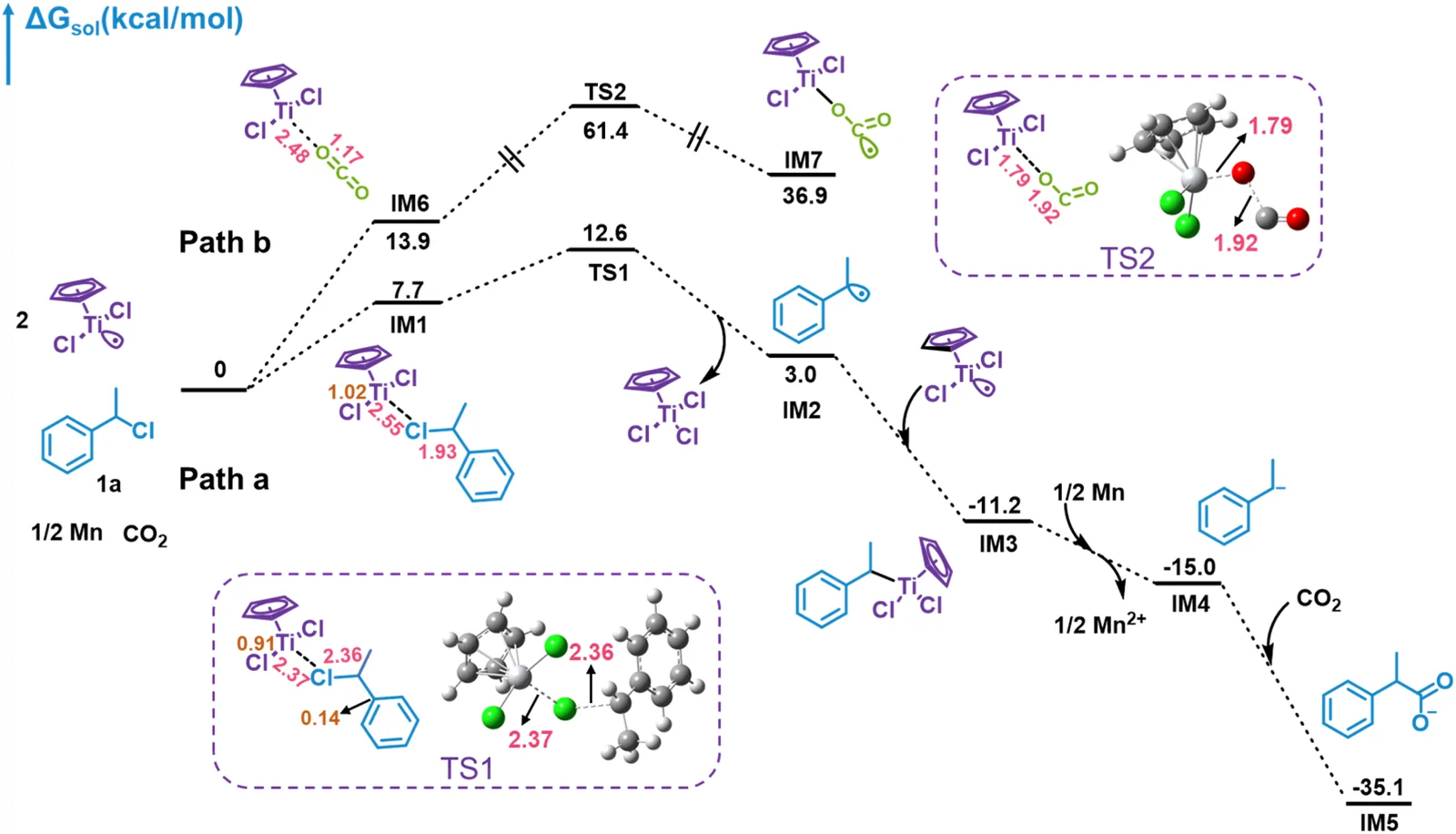
Free energy profile for the Ti(iii)-catalyzed C–Cl bond activation of substrate 1a (path a) *versus* direct single-electron transfer to CO_2_ (path b). Selected bond distances in pink font are given in Å. The numbers in brown font on selected atoms in IM1 and TS1 denote spin densities.

Taken together, the experimental and computational results support the catalytic cycle depicted in Fig. 5: CpTi^IV^Cl_3_ is reduced by Mn to CpTi^III^Cl_2_, which effects chlorine atom abstraction *via* an inner-sphere electron-transfer process to liberate the benzyl radical IM2. The radical is trapped by CpTi^III^Cl_2_ to form a benzyltitanium(iv) complex IM3, which is further reduced by Mn to generate the benzyl anion IM4 while regenerating CpTi^III^Cl_2_. Nucleophilic attack of the anion on CO_2_ followed by acidic workup affords the final arylacetic acid product 2a. This mechanistic picture reveals that Ti^III^ species promote sequential single-electron transfer events to accomplish C–Cl bond cleavage, radical capture, and anionic carboxylation, with inner-sphere C–Cl bond activation constituting the kinetically decisive step of the catalytic cycle. For comparison, the alternative pathway involving direct CO_2_ activation by Ti^III^ (path b, Fig. 5) is disfavored on both kinetic and thermodynamic grounds, as evidenced by the prohibitively high barrier (61.4 kcal mol^−1^) computed for CO_2_˙^−^ formation. Further experimental support for this assignment comes from HRMS analysis: the key benzyl–titanium intermediate IM3 (detected as complex 12) was clearly detected, consistent with the operative path a, whereas the putative Ti–CO_2_ adduct IM7, or its downstream derivative IM8, anticipated for path b was not observed, providing additional evidence against this pathway.

**Fig. 5:**
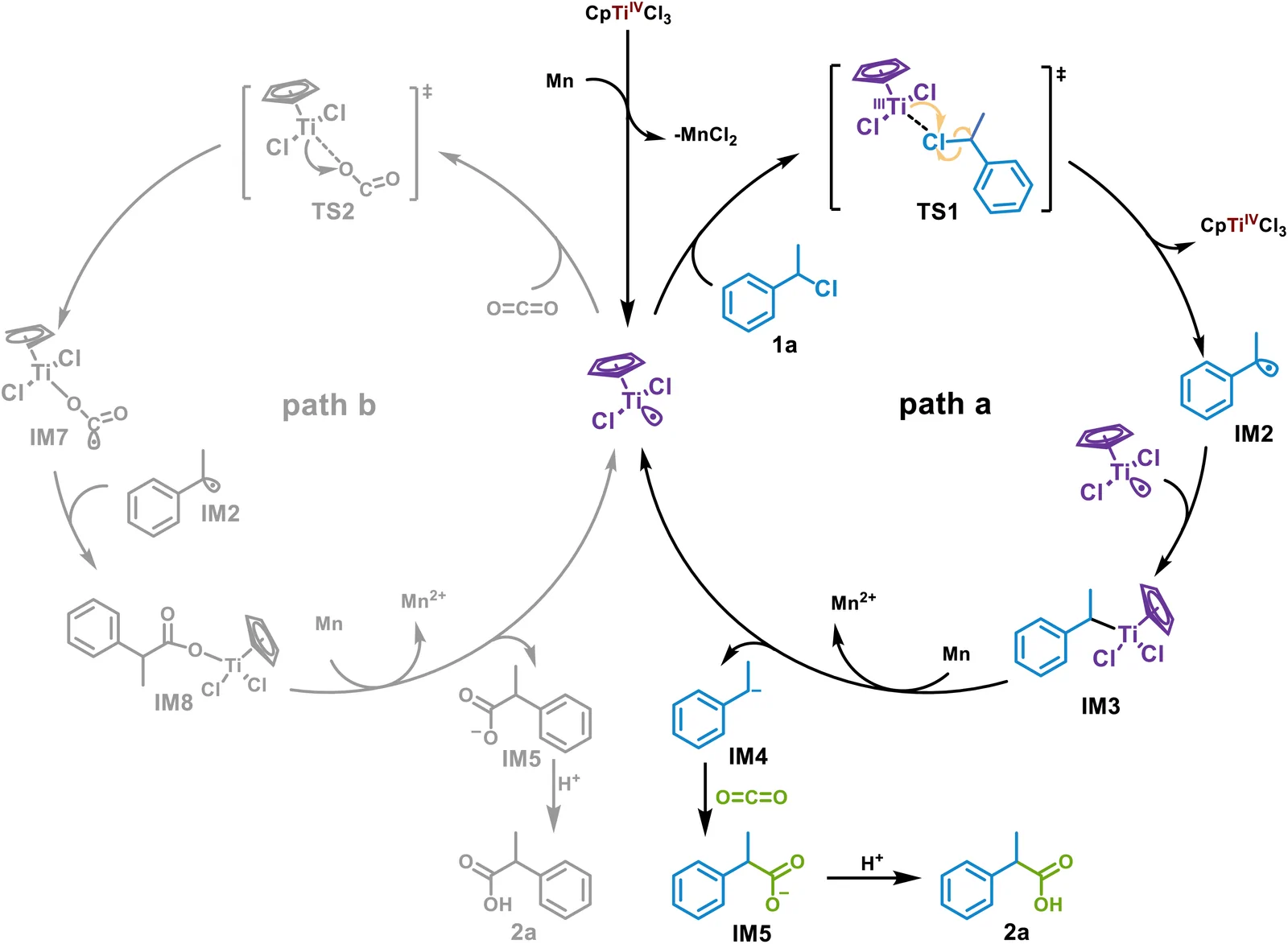
Proposed catalytic cycle.

## Conclusions

We have developed a titanium(iii)-catalyzed reductive carboxylation of benzyl halides with CO_2_ that proceeds through a radical–carbanion relay pathway. The key features of this method—broad substrate scope (1°, 2°, and 3° C (sp^3^)–Cl, benzyl bromides, and allylic chlorides), oxygen tolerance, and the absence of salt additives for secondary and tertiary substrates—address several long-standing limitations in CO_2_ carboxylation chemistry. The direct synthesis of flurbiprofen, naproxen, and ibuprofen illustrates the practical relevance of this protocol for pharmaceutical synthesis. Mechanistically, combined experimental and computational evidence reveals that the inner-sphere chlorine-atom abstraction by Ti(iii) constitutes the kinetically decisive step, followed by radical capture, carbanion formation, and nucleophilic CO_2_ trapping. This work demonstrates that exploiting the single-electron reactivity of earth-abundant titanium, rather than its direct CO_2_ activation capability, offers a viable and complementary strategy for CO_2_ valorization. Future efforts will focus on extending this concept to other classes of electrophiles and developing asymmetric variants for enantioselective carboxylation.

## Author contributions

L. Fan: investigation, methodology, formal analysis, writing – original draft. J. Zhao: investigation (DFT calculations), formal analysis, writing – original draft. H. Sun: formal analysis (X-ray crystallography, EPR test). W. Zhang: investigation (DFT calculations), formal analysis. G. Zhang: mechanistic investigation. Y. Jian: conceptualization, supervision, writing – review and editing, funding acquisition. Z. Gao: conceptualization, supervision, project administration, funding acquisition, writing – review and editing.

## Conflicts of interest

There are no conflicts to declare.

## Supplementary Material

SC-017-D6SC04813G-s001

SC-017-D6SC04813G-s002

## Data Availability

CCDC 2556758 contains the supplementary crystallographic data for this paper.^75^ The data that support the findings of this study are openly available in the supplementary information (SI). Supplementary information: experimental procedures, NMR spectra, high-resolution mass spectrometry data, crystallographic data, DFT calculations, and EPR spectroscopic data. See DOI: https://doi.org/10.1039/d6sc04813g.
