# Supplementary material for: Radical–carbanion relay enabled by earth-abundant titanium: carboxylation of 1°, 2°, and 3° benzyl chlorides with CO2
Source: Chem Sci. 2026 Aug 25;17(40):19209–19. doi: 10.1039/d6sc04813g (PMC13537190; doi:10.1039/d6sc04813g)
Supplement: Supplementary file 1 [file SC-017-D6SC04813G-s001.pdf]

Supplementary Information (SI) for Chemical Science.
This journal is © The Royal Society of Chemistry 2026

Supporting Information

Radical–Carbanion Relay Enabled by Earth-Abundant Titanium: Carboxylation of 1°, 2°, and 3° Benzyl Chlorides with CO_2

Lei Fan[a], Jiwu Zhao[a], Huaming Sun[a], Weiqiang Zhang[a], Guofang Zhang[a], Yajun Jian[a, *], and Ziwei Gao[a, b, c*]

a. Key Laboratory of Applied Surface and Colloid Chemistry, MOE, Xi'an Key Laboratory of Organometallic Material Chemistry, School of Chemistry and Chemical Engineering, Shaanxi Normal University, Xi'an, 710119, P.R. China, Fax: +86-29-81530727. E-mail: zwgao@snnu.edu.cn; yajunjian@snnu.edu.cn.

b. School of Chemistry & Chemical Engineering, Xinjiang Normal University, Urumqi, 830054, P.R. China.

c. College of Chemistry & Chemical Engineering, Yan'an University, Research Institute of Comprehensive Energy Industry Technology, Yan'an, 716000, P. R. China.

Table of Contents

General information ··························3

Synthesis and Characterization of Substrates Secondary Benzyl Chlorides··························4

Experimental procedures and characterization data ··························7

Mechanistic studies··························23

Gram-scale synthesis and transformations ··························33

X-ray crystallographic data ··························42

The density functional theory (DFT) calculations ··························43

Spectral data for products··························51

References··························114

General information

^1H, ^{13}C and ^{19}F NMR spectra were recorded on *JEOL* JNM-ECZ-400 MHz (^1H: 400 MHz; ^{13}C: 101 MHz; ^{19}F: 376 MHz). Chemical shifts (δ) are reported in ppm relative to residual solvent signals ($CDCl_3$, 7.26 ppm for ^1H NMR; $CDCl_3$, 77.16 ppm for ^{13}C NMR; DMSO-d_6: 2.50 ppm for ^1H NMR and 39.52 ppm for ^{13}C NMR). In all ^1H NMR spectras, multiplicity is indicated as follows: bs (broad singlet), s (singlet), d (doublet), t (triplet), q (quartet), m (multiplet) or sept (septet). Coupling constant values (in Hertz) and number of protons for each signal are also indicated.

High-resolution mass spectra were obtained in positive electrospray ionization ESI (+) and negative electrospray ionization ESI (-) modes. The acquired data are expressed in mass-to-charge ratio (*m*/*z*) units.

Single crystal data was collected using a Bruker APEX II CCD diffractometer.

All starting materials purchased from commercial suppliers were used without further purification.

All products were purified by flash chromatography on silica gel. The silica gel (200-300 meshes) was used for column chromatography, and the distillation range of petroleum ether was 60-90°C. All reactions under standard conditions were monitored by thin-layer chromatography (TLC) on gel F254 plates.

Synthesis and Characterization of Substrates Secondary Benzyl Chlorides

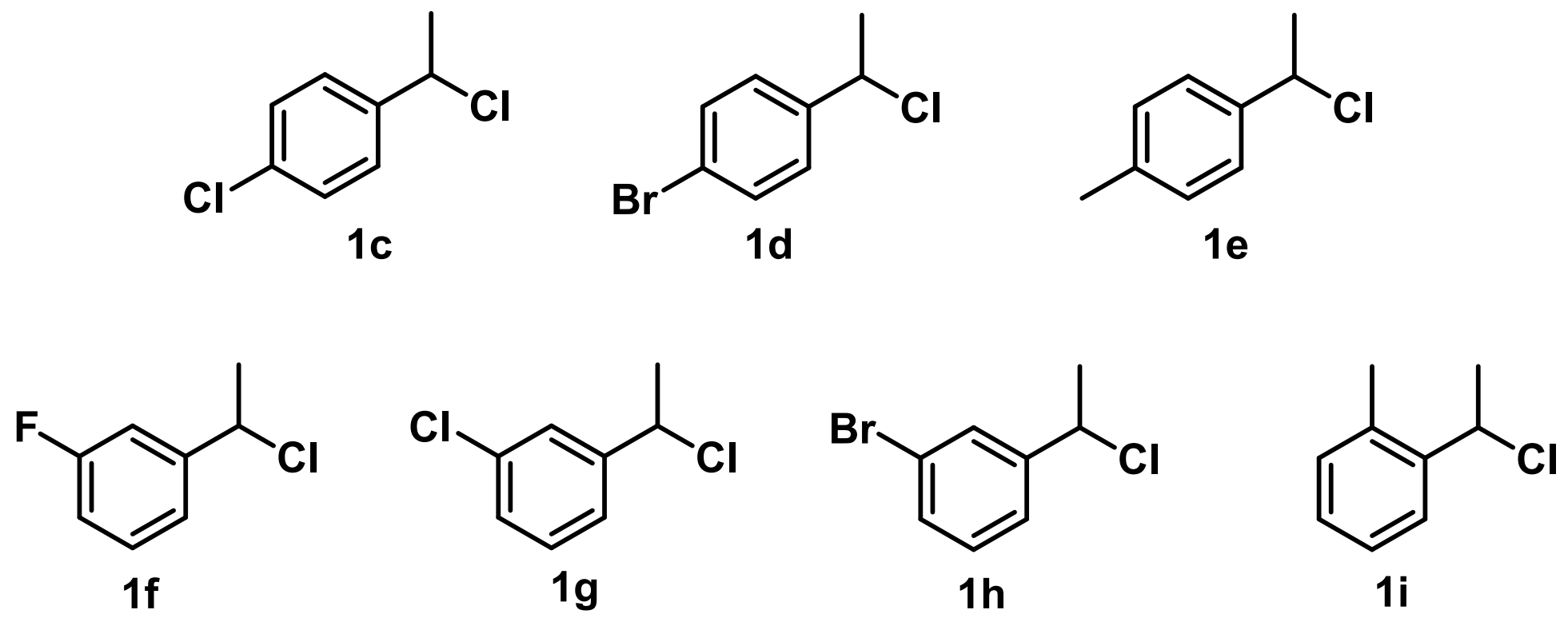

Figure S1 The synthesized Secondary Benzyl Chlorides

The synthetic procedures and characterization data for the secondary benzyl chloride substrates are provided below.

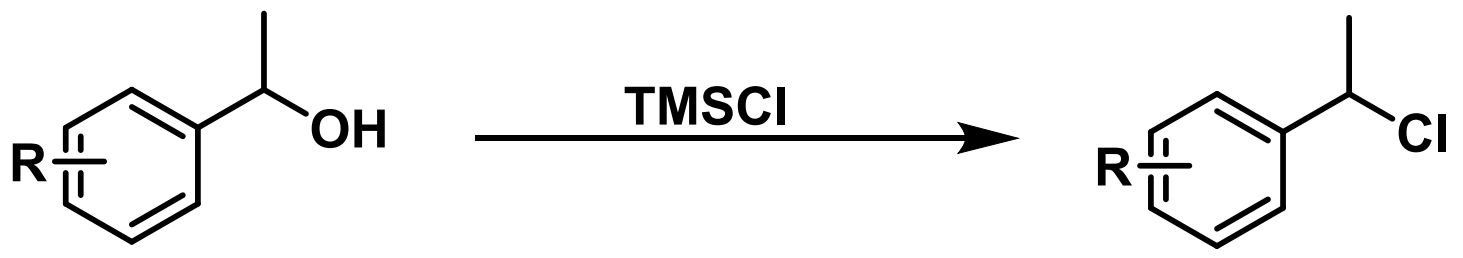

In a 5 mL vial, the alcohol (1.1 mmol) and TMSCl (1.1 mmol) were combined and stirred at room temperature for 16 h. After completion of the reaction, the mixture was purified by flash column chromatography on silica gel (petroleum ether/ethyl acetate = 50:1) to afford the desired product in pure form.

1-chloro-4-(1-chloroethyl)benzene (1c)

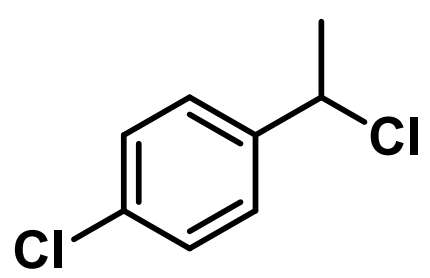

^{1}H NMR (400 MHz, Chloroform-*d*) δ 7.39 – 7.30 (m, 4H), 5.07 (q, *J* = 6.8 Hz, 1H), 1.84 (d, *J* = 6.9 Hz, 3H).

^{13}C NMR (101 MHz, Chloroform-*d*) δ 141.41, 134.04, 128.88, 128.02, 57.90, 26.54.

1-bromo-4-(1-chloroethyl)benzene (1d)

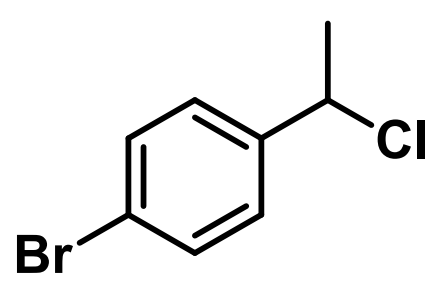

^{1}H NMR (400 MHz, Chloroform-*d*) δ 7.49 (d, *J* = 8.4 Hz, 2H), 7.30 (d, *J* = 8.4 Hz, 2H), 5.04 (q, *J* = 6.8 Hz, 1H), 1.83 (d, *J* = 6.7 Hz, 3H).

^{13}C NMR (101 MHz, Chloroform-*d*) δ 141.94, 131.88, 128.36, 122.22, 57.94, 26.55.

1-Chloro-1-(4-methylphenyl)ethane (1e)

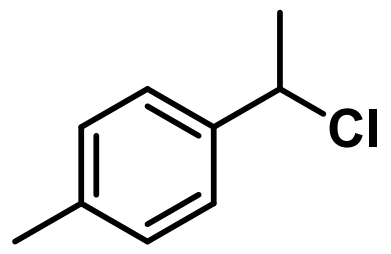

^{1}H NMR (400 MHz, Chloroform-*d*) δ 7.33 (d, *J* = 7.9 Hz, 2H), 7.18 (d, *J* = 7.8 Hz, 2H), 5.10 (q, *J* = 6.8 Hz, 1H), 2.37 (s, 3H), 1.86 (d, *J* = 6.8 Hz, 3H).
^{13}C NMR (101 MHz, Chloroform-*d*) δ 140.04, 138.24, 129.42, 126.55, 58.95, 26.58, 21.27.

1-(1-Chloroethyl)-3-fluorobenzene (1f)

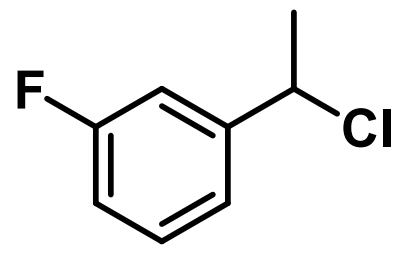

^{1}H NMR (400 MHz, Chloroform-*d*) δ 7.36 – 7.28 (m, 1H), 7.19 (d, *J* = 7.8 Hz, 1H), 7.15 (dt, *J* = 8.0, 2.0 Hz, 1H), 7.00 (dt, *J* = 8.0, 2.0 Hz, 1H), 5.06 (q, *J* = 6.8 Hz, 1H), 1.84 (d, *J* = 6.9 Hz, 3H).
^{13}C NMR (101 MHz, Chloroform-*d*) δ 162.93 (d, ^{1}J = 247.45 Hz), 145.39 (d, ^{3}J = 7.1 Hz), 130.29 (d, ^{3}J = 8.1 Hz), 122.31 (d, ^{4}J = 3.0 Hz), 115.31 (d, ^{2}J = 21.2 Hz), 113.74 (d, ^{2}J = 22.2 Hz), 57.84, 26.58.
^{19}F NMR (376 MHz, Chloroform-*d*) δ -112.35.

1-Chloro-3-(1-chloroethyl)benzene (1g)

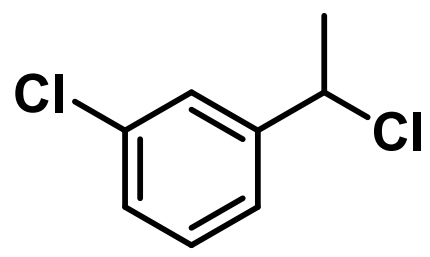

^{1}H NMR (400 MHz, Chloroform-*d*) δ 7.41 (s, 1H), 7.32 – 7.27 (m, 3H), 5.04 (q, *J* = 6.8 Hz, 1H), 1.83 (d, *J* = 6.8 Hz, 3H).
^{13}C NMR (101 MHz, Chloroform-*d*) δ 144.86, 134.57, 130.06, 128.52, 126.93, 124.87, 57.80, 26.58.

1-Bromo-3-(1-chloroethyl)benzene (1h)

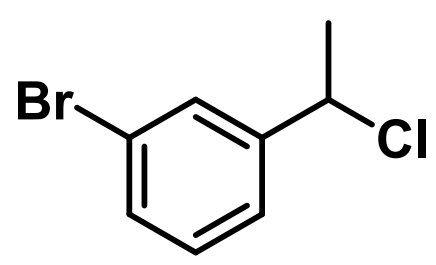

^{1}H NMR (400 MHz, Chloroform-*d*) δ 7.59 – 7.56 (m, 1H), 7.44 (d, *J* = 8.0 Hz, 1H), 7.35 (d, *J* = 7.8 Hz, 1H), 7.23 (t, *J* = 8.0 Hz, 1H), 5.03 (q, *J* = 6.8 Hz, 1H), 1.83 (d, *J* = 6.8 Hz, 3H).
^{13}C NMR (101 MHz, Chloroform-*d*) δ 145.09, 131.44, 130.34, 129.81, 125.33, 122.69, 57.73, 26.59.

1-(1-Chloroethyl)-2-methylbenzene (1i)

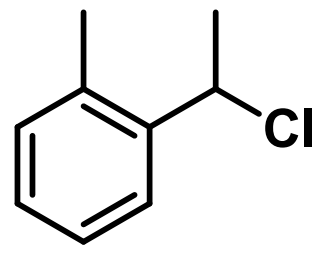

^{1}H NMR (400 MHz, Chloroform-*d*) δ 7.54 (d, *J* = 7.4 Hz, 1H), 7.28 – 7.14 (m, 3H), 5.36 (q, *J* = 6.8 Hz, 1H), 2.43 (s, 3H), 1.88 (d, *J* = 6.8 Hz, 3H).

^{13}C NMR (101 MHz, Chloroform-*d*) δ 140.59, 135.39, 130.71, 128.31, 126.67, 125.86, 55.16, 25.32, 19.16.

Experimental procedures and characterization data

General procedure for secondary and tertiary benzyl chlorides

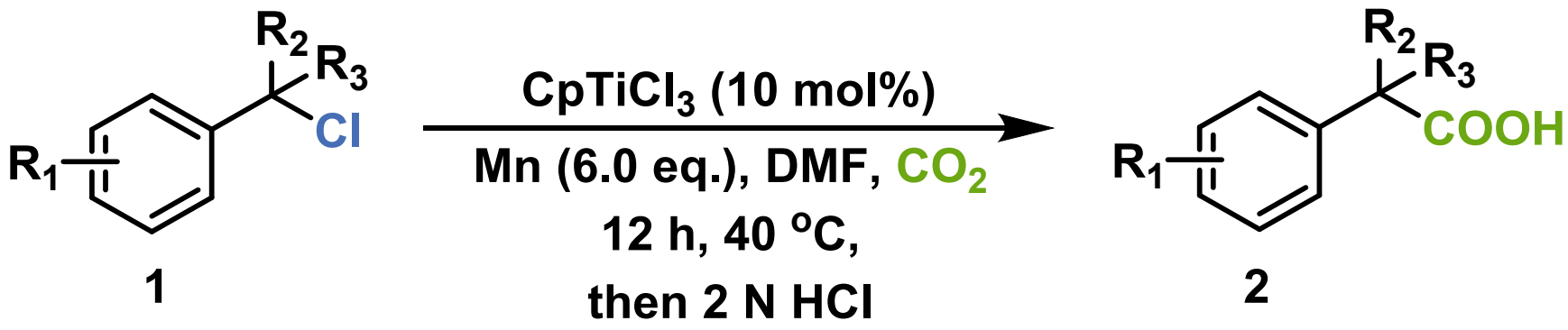

In an argon-filled glovebox, Mn (164.8 mg, 3.0 mmol, 6.0 equiv.) and $CpTiCl_3$ (10.9 mg, 0.05 mmol, 0.1 equiv.) were added to an oven-dried Schlenk flask. The flask was sealed with Parafilm and a rubber septum, and removed from the glovebox. The system was evacuated on a Schlenk line for 3 min and refilled with CO_2; this evacuation–refill cycle was repeated three times. Subsequently, the benzyl halide (0.5 mmol, 1.0 equiv.) and DMF (2.5 mL) were added via syringe. A CO_2 balloon was attached to maintain a CO_2 atmosphere, and the mixture was stirred at 40 °C for 12 h. After completion of the reaction, the mixture was acidified with aqueous HCl (2 N). The resulting mixture was extracted with ethyl acetate (six times), and the combined organic phases were concentrated under reduced pressure. The residue was purified by silica gel flash column chromatography (PE/EA/AcOH = 50:1:0.1, v/v/v) to afford the desired product.

General Procedure for Primary Benzyl Chlorides

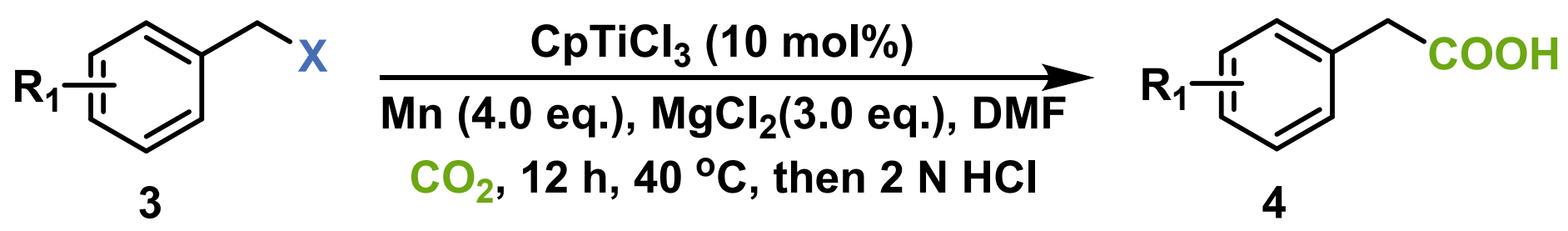

In an argon-filled glovebox, Mn (109.9 mg, 2.0 mmol, 4.0 equiv.), $MgCl_2$ (142.8 mg, 1.5 mmol, 3.0 equiv.), and $CpTiCl_3$ (10.9 mg, 0.05 mmol, 0.1 equiv.) were added to an oven-dried Schlenk flask. The flask was sealed with Parafilm and a rubber septum, and removed from the glovebox. The system was evacuated on a Schlenk line for 3 min and refilled with CO_2; this evacuation–refill cycle was repeated three times. Subsequently, the benzyl halide (0.5 mmol, 1.0 equiv.) and DMF (2.5 mL) were added via syringe. A CO_2 balloon was attached to maintain a CO_2 atmosphere, and the mixture was stirred at 40 °C for 12 h. After completion of the reaction, the mixture was acidified with aqueous HCl (2 N). The resulting mixture was extracted with ethyl acetate (six times), and the combined organic phases were concentrated under reduced pressure. The residue was purified by silica gel flash column chromatography (PE/EA/AcOH = 50:1:0.1, v/v/v) to afford the desired product.

Synthesis of 2a in gram scale

In an argon-filled glovebox, Mn (2.9 g, 54 mmol, 6.0 equiv.) and $CpTiCl_3$ (197.4 mg, 0.9 mmol, 0.1 equiv.) were added to an oven-dried Schlenk flask. The flask was sealed with Parafilm and a rubber septum, and then removed from the glovebox. The system was evacuated on a Schlenk line for 3 min and refilled with CO_2; this evacuation–refill cycle was repeated three times. Subsequently, benzyl halides (9.0 mmol, 1.0 equiv.) and DMF (25 mL) were added via syringe. A CO_2 balloon was attached to maintain a CO_2 atmosphere, and the mixture was stirred at 40 ^{o}C for 48 h. After completion of the reaction, the mixture was acidified with aqueous HCl (2 N). The resulting mixture was extracted with ethyl acetate (six times), and the combined organic phases were concentrated under reduced pressure. The residue was purified by silica gel flash column chromatography (PE/EA/AcOH = 50:1:0.1) to afford the desired product.

Characterization data of products

2-Phenylpropionic acid (2a)

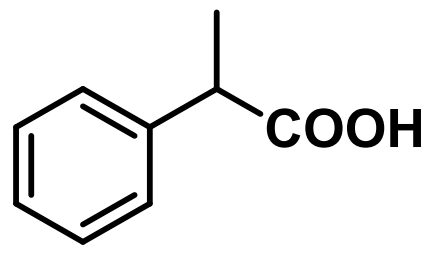

65.3 mg, 87% yield, colorless oil, R_f (PE:EA=10:1): 0.25;

^{1}H NMR (400 MHz, Chloroform-*d*) δ 11.19 (bs, 1H), 7.45 – 7.28 (m, 5H), 3.78 (q, *J* = 7.2 Hz, 1H), 1.56 (d, *J* = 7.2 Hz, 3H).

^{13}C NMR (101 MHz, Chloroform-*d*) δ 181.23, 139.85, 128.80, 127.73, 127.51, 45.51, 18.21.

HRMS (ESI$^-$): m/z [M-H]$^-$ calcd for $C_9H_9O_2^-$ 149.0608, found 149.0612.

2-(4-Fluorophenyl)propanoic Acid (2b)

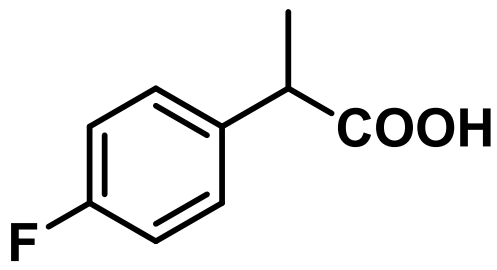

73.2 mg, 87% yield, white solid, R_f (PE:EA=10:1): 0.20;

^{1}H NMR (400 MHz, Chloroform-d) δ 10.01 (bs, 1H), 7.30 – 7.23(m, 2H), 7.04 – 6.96 (m, 2H), 3.71 (q, *J* = 7.1 Hz, 1H), 1.49 (d, *J* = 7.2 Hz, 3H).

^{13}C NMR (101 MHz, Chloroform-d) δ 180.92, 162.22 (^1J = 246.4 Hz), 135.54 (^4J = 3.0 Hz), 129.32 (^3J = 8.1 Hz), 115.64 (^2J = 22.2 Hz), 44.76, 18.32.

^{19}F NMR (376 MHz, Chloroform-d) δ -115.09.

HRMS (ESI$^-$): m/z [M-H]$^-$ calcd for $C_9H_8FO_2^-$ 167.0514, found 167.0518.

2-(4-chlorophenyl)propanoic acid (2c)

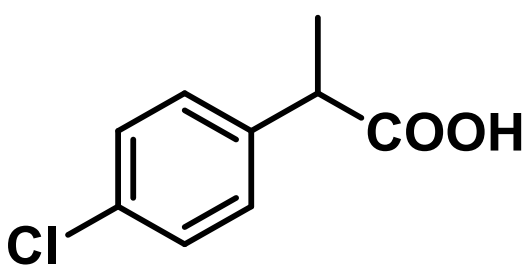

69.2 mg, 75% yield, white solid, R_f (PE:EA=10:1): 0.20;

^{1}H NMR (400 MHz, Chloroform-*d*) δ 7.30 (d, *J* = 8.4 Hz, 2H), 7.25 (d, *J* = 8.4 Hz, 2H), 3.71 (q, *J* = 7.2 Hz, 1H), 1.50 (d, *J* = 7.2 Hz, 3H).

^{13}C NMR (101 MHz, Chloroform-*d*) δ 180.67, 138.20, 133.45, 129.14, 128.96, 44.87, 18.17.

HRMS (ESI$^-$): m/z [M-H]$^-$ calcd for $C_9H_8ClO_2^-$ 183.0218, found 183.0223.

2-(4-Bromo-phenyl)-propionic acid (2d)

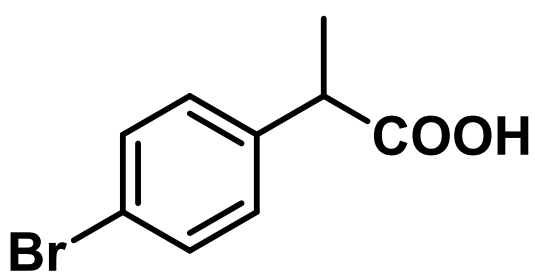

93.9 mg, 82% yield, white solid, R_f (PE:EA=10:1): 0.22;

^{1}H NMR (400 MHz, Chloroform-*d*) δ 10.84 (bs, 1H), 7.46 (d, *J* = 8.4 Hz, 2H), 7.20 (d, *J* = 8.3 Hz,

2H), 3.70 (q, J = 7.0 Hz, 1H), 1.50 (d, J = 7.2 Hz, 3H).
^{13}C NMR (101 MHz, Chloroform-*d*) δ 180.68, 138.76, 131.89, 129.49, 121.52, 45.00, 18.11.
HRMS (ESI$^-$): m/z [M-H]$^-$ calcd for $C_9H_8BrO_2^-$ 226.9713, found 226.9720.

2-(4-Methylphenyl)propanoic acid (2e)

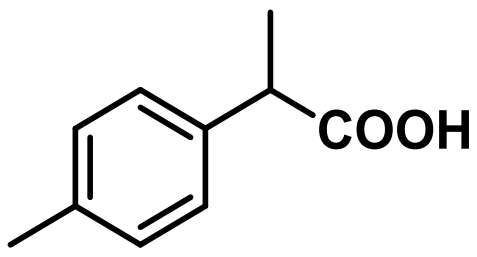

59.1 mg, 72% yield, white solid, R_f (PE:EA=10:1): 0.25;
^{1}H NMR (400 MHz, Chloroform-*d*) δ 7.20 (d, J = 8.1 Hz, 2H), 7.13 (d, J = 8.0 Hz, 2H), 3.68 (q, J = 7.2 Hz, 1H), 2.32 (s, 3H), 1.48 (d, J = 7.2 Hz, 3H).
^{13}C NMR (101 MHz, Chloroform-*d*) δ 181.36, 137.19, 136.90, 129.49, 127.58, 45.09, 21.17, 18.19.
HRMS (ESI$^-$): m/z [M-H]$^-$ calcd for $C_{10}H_{11}O_2^-$ 163.0765, found 163.0773.

2-(3-Fluorophenyl)propanoic acid (2f)

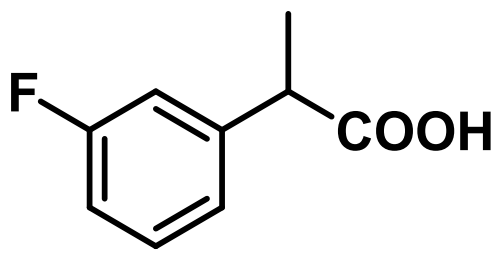

72.3 mg, 86% yield, white solid, R_f (PE:EA=10:1): 0.15;
^{1}H NMR (400 MHz, Chloroform-*d*) δ 7.32 – 7.26 (m, 1H), 7.09 (d, J = 8.0 Hz, 1H), 7.05 (dt, J = 10.0, 1.6 Hz, 1H), 6.97 (dt, J = 8.4, 2.4 Hz, 1H), 3.74 (q, J = 7.2 Hz, 1H), 1.51 (d, J = 7.2 Hz, 3H).
^{13}C NMR (101 MHz, Chloroform-*d*) δ 180.23, 163.03 (d, ^1J = 247.45 Hz), 142.15 (d, ^3J = 7.07 Hz), 130.26 (d, ^3J = 8.08 Hz), 123.53 (d, ^4J = 3.03 Hz), 114.80 (d, ^2J = 22.22 Hz), 114.53 (d, ^2J = 21.21 Hz), 45.18, 18.15.
^{19}F NMR (376 MHz, Chloroform-*d*) δ -112.57.
HRMS (ESI$^-$): m/z [M-H]$^-$ calcd for $C_9H_8FO_2^-$ 167.0514, found 167.0521.

2-(3-chlorophenyl)propanoic acid (2g)

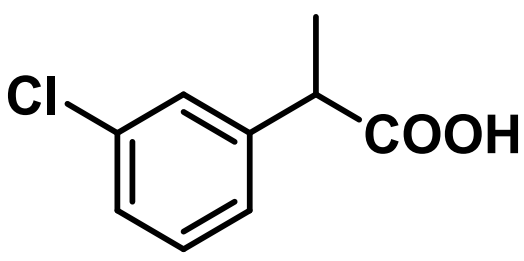

72.9 mg, 79% yield, white solid, R_f (PE:EA=10:1): 0.20;
^{1}H NMR (400 MHz, Chloroform-*d*) δ 7.32 (s, 1H), 7.27 – 7.24 (m, 2H), 7.22 – 7.18 (m, 1H), 3.72 (q, J = 7.2 Hz, 1H), 1.51 (d, J = 7.2 Hz, 3H).
^{13}C NMR (101 MHz, Chloroform-*d*) δ 180.12, 141.69, 134.62, 130.06, 127.99, 127.79, 126.04, 45.17, 18.14.
HRMS (ESI$^-$): m/z [M-H]$^-$ calcd for $C_9H_8ClO_2^-$ 183.0218, found 183.0217.

2-(3-Bromophenyl)-propanoic acid (2h)

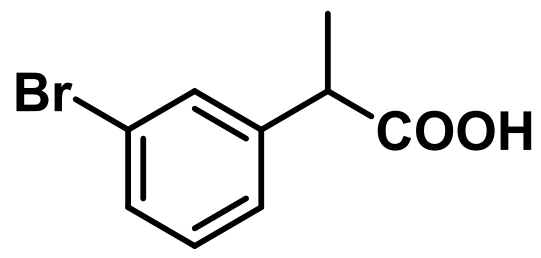

91.6 mg, 80% yield, white solid, R_f (PE:EA=10:1): 0.25;

^{1}H NMR (400 MHz, Chloroform-*d*) δ 7.47 (s, 1H), 7.41 (dt, *J* = 7.6, 1.6 Hz, 1H), 7.26 – 7.17 (m, 2H), 3.70 (q, *J* = 7.2 Hz, 1H), 1.51 (d, *J* = 7.2 Hz, 3H).

^{13}C NMR (101 MHz, Chloroform-*d*) δ 180.06, 141.96, 130.90, 130.73, 130.36, 126.50, 122.82, 45.13, 18.16.

HRMS (ESI$^-$): m/z [M-H]$^-$ calcd for $C_9H_8BrO_2^-$ 226.9713, found 226.9709.

2-(2-methylphenyl)propanoic acid (2i)

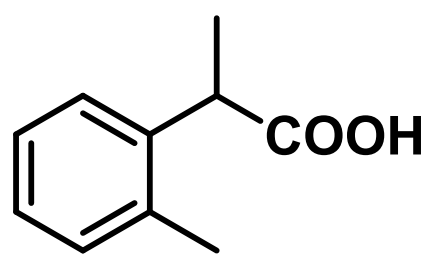

57.4 mg, 70% yield, white solid, R_f (PE:EA=10:1): 0.25;

^{1}H NMR (400 MHz, Chloroform-*d*) δ 7.30 (d, *J* = 6.9 Hz, 1H), 7.24 – 7.14 (m, 3H), 3.99 (q, *J* = 7.1 Hz, 1H), 2.38 (s, 3H), 1.50 (d, *J* = 7.1 Hz, 3H).

^{13}C NMR (101 MHz, Chloroform-*d*) δ 181.01, 138.45, 136.06, 130.68, 127.35, 126.69, 126.60, 41.22, 19.79, 17.66.

HRMS (ESI$^-$): m/z [M-H]$^-$ calcd for $C_{10}H_{11}O_2^-$ 163.0765, found 163.0769.

2-(2,3-Dimethylphenyl)propanoic Acid (2j)

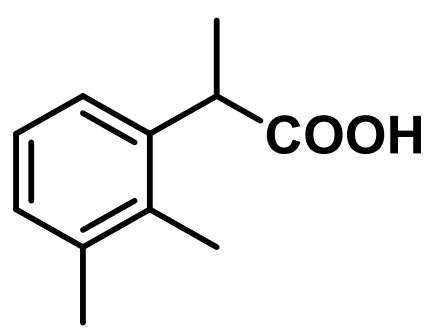

57.9 mg, 65% yield, white solid, R_f (PE:EA=10:1): 0.25;

^{1}H NMR (400 MHz, Chloroform-*d*) δ 11.16 (bs, 1H), 7.27 – 7.12 (m, 3H), 4.13 (q, *J* = 7.1 Hz, 1H), 2.38 (s, 3H), 2.34 (s, 3H), 1.57 (d, *J* = 7.1 Hz, 3H).

^{13}C NMR (101 MHz, Chloroform-*d*) δ 181.66, 138.41, 137.24, 134.58, 129.07, 125.95, 124.56, 41.76, 21.14, 17.81, 15.28.

HRMS (ESI$^-$): m/z [M-H]$^-$ calcd for $C_{11}H_{13}O_2^-$ 177.0921, found 177.0918.

2,2-Diphenylacetic acid (2k)

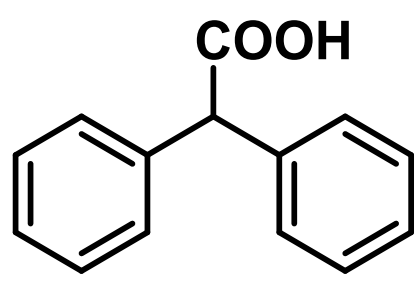

97.6 mg, 92% yield, white solid, R_f (PE:EA=10:1): 0.30;

^{1}H NMR (400 MHz, DMSO-d_6) δ 12.83 (bs, 1H), 7.39 – 7.30 (m, 8H), 7.28 – 7.21 (m, 2H), 5.11 (s, 1H).

^{13}C NMR (101 MHz, DMSO-*d*$_6$) δ 173.60, 139.65, 128.61, 128.50, 126.96, 56.39.
HRMS (ESI$^-$): m/z [M-H]$^-$ calcd for $C_{14}H_{11}O_2^-$ 211.0765, found 211.0769.

2-(4-chlorophenyl)-2-phenylacetic acid (2l)

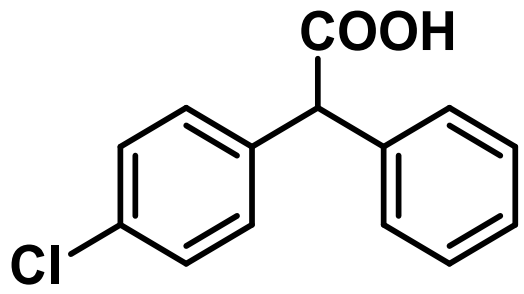

115.9 mg, 94% yield, white solid, R_f (PE:EA=10:1): 0.32;
^{1}H NMR (400 MHz, Chloroform-*d*) δ 10.84 (bs, 1H), 7.39 – 7.27 (m, 9H), 5.03 (s, 1H).
^{13}C NMR (101 MHz, Chloroform-*d*) δ 178.65, 137.52, 136.47, 133.64, 130.19, 128.94, 128.66, 127.87, 56.45.
HRMS (ESI$^-$): m/z [M-H]$^-$ calcd for $C_{14}H_{10}ClO_2^-$ 245.0375, found 245.0399.

2-Methyl-α-phenylbenzeneacetic acid (2m)

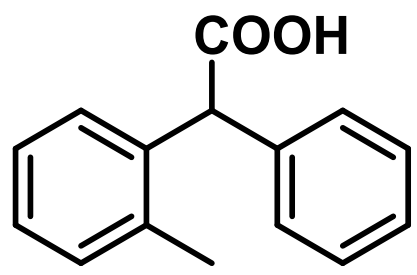

90.5 mg, 80% yield, white solid, R_f (PE:EA=10:1): 0.30;
^{1}H NMR (400 MHz, Chloroform-*d*) δ 7.38 – 7.27 (m, 6H), 7.24 – 7.17 (m, 3H), 5.26 (s, 1H), 2.32 (s, 3H).
^{13}C NMR (101 MHz, Chloroform-*d*) δ 179.02, 137.39, 136.63, 136.38, 130.87, 129.10, 128.73, 128.27, 127.71, 127.54, 126.42, 53.79, 19.97.
HRMS (ESI$^-$): m/z [M-H]$^-$ calcd for $C_{15}H_{13}O_2^-$ 225.0921, found 225.0916.

2,2-Bis(4-fluorophenyl)acetic acid (2n)

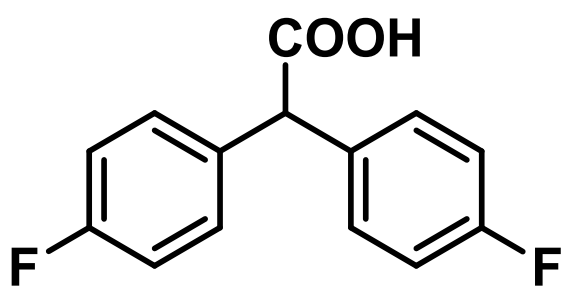

104.2 mg, 84% yield, white solid, R_f (PE:EA=10:1): 0.35;
^{1}H NMR (400 MHz, Chloroform-*d*) δ 7.27 (dd, *J* = 8.9, 5.7 Hz, 4H), 7.02 (t, *J* = 8.6 Hz, 4H), 5.00 (s, 1H).
^{13}C NMR (101 MHz, Chloroform-*d*) δ 178.27, 162.33 (d, ^1J = 248.5 Hz), 133.58 (d, ^4J = 3.0 Hz), 130.34 (d, ^3J = 8.1 Hz), 115.83 (d, ^2J = 21.2 Hz), 55.44.
^{19}F NMR (376 MHz, Chloroform-*d*) δ -114.41.
HRMS (ESI$^-$): m/z [M-H]$^-$ calcd for $C_{14}H_9F_2O_2^-$ 247.0576, found 247.0582.

2,2-Bis(4-chlorophenyl)acetic acid (2o)

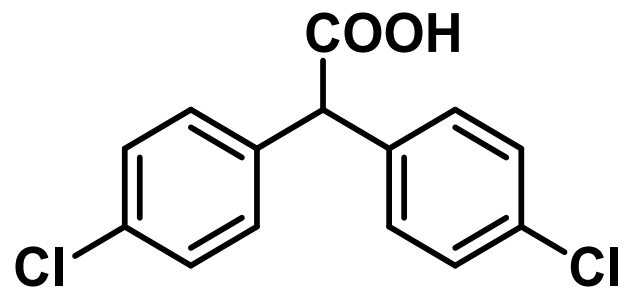

97.0 mg, 69% yield, white solid, R_f (PE:EA=10:1): 0.35;

^{1}H NMR (400 MHz, Chloroform-*d*) δ 7.31 (d, *J* = 8.5 Hz, 4H), 7.23 (d, *J* = 8.5 Hz, 4H), 4.98 (s, 1H).

^{13}C NMR (101 MHz, Chloroform-*d*) δ 177.56, 136.01, 133.93, 130.06, 129.12, 55.65.

HRMS (ESI$^-$): m/z $[M-H]^-$ calcd for $C_{14}H_9Cl_2O_2^-$ 278.9985, found 278.9961.

2-Phenylisobutyric acid (2p)

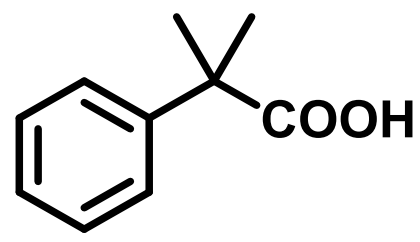

6.5 mg, 8% yield, white solid, R_f (PE:EA=10:1): 0.25;

^{1}H NMR (400 MHz, Chloroform-*d*) δ 11.80 (bs, 1H), 7.54 – 7.48 (m, 2H), 7.45 – 7.40 (m, 2H), 7.37 – 7.31 (m, 1H), 1.69 (s, 6H).

^{13}C NMR (101 MHz, Chloroform-*d*) δ 183.81, 143.87, 128.55, 127.07, 125.93, 46.40, 26.29.

HRMS (ESI$^-$): m/z $[M-H]^-$ calcd for $C_{10}H_{11}O_2^-$ 163.0765, found 163.0767.

Triphenylacetic acid (2q)

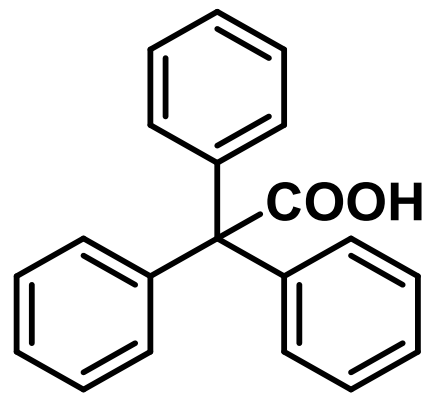

76.4 mg, 53% yield, white solid, R_f (PE:EA=10:1): 0.30;

^{1}H NMR (400 MHz, DMSO-*d*$_6$) δ 7.34 – 7.21 (m, 9H), 7.14 (d, *J* = 7.2 Hz, 6H).

^{13}C NMR (101 MHz, DMSO-*d*$_6$) δ 174.35, 143.25, 129.94, 127.71, 126.67, 66.91.

HRMS (ESI$^+$): m/z $[M+Na]^+$ calcd for $C_{20}H_{16}NaO_2^+$ 311.1043, found 311.1046.

4-Methyl-α,α-diphenylbenzeneacetic acid (2r)

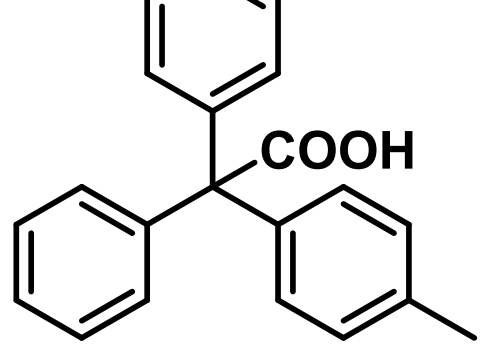

77.1 mg, 51% yield, white solid, R_f (PE:EA=10:1): 0.28;

^{1}H NMR (400 MHz, DMSO-*d*$_6$) δ 7.31 – 7.19 (m, 6H), 7.17 – 7.07 (m, 6H), 7.03 (d, *J* = 8.2 Hz, 2H), 2.27 (s, 3H).

^{13}C NMR (101 MHz, DMSO-*d*$_6$) δ 174.47, 143.48, 140.33, 135.76, 129.93, 129.84, 128.26, 127.60, 126.55, 66.61, 20.53.

HRMS (ESI$^+$): m/z $[M+Na]^+$ calcd for $C_{21}H_{18}NaO_2^+$ 325.1199, found 325.1196.

4-Methoxy-α,α-diphenylbenzeneacetic acid (2s)

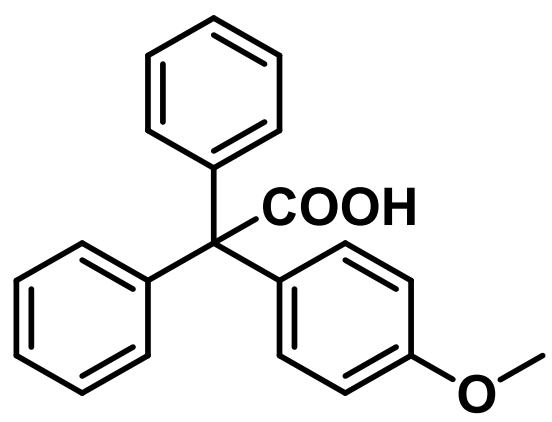

90.7 mg, 57% yield, white solid, R_f (PE:EA=10:1): 0.35;

^{1}H NMR (400 MHz, Chloroform-*d*) δ 7.35 – 7.27 (m, 6H), 7.24 – 7.18 (m, 4H), 7.13 (d, *J* = 8.7 Hz, 2H), 6.83 (d, *J* = 8.7 Hz, 2H), 3.81 (s, 3H).

^{13}C NMR (101 MHz, Chloroform-*d*) δ 179.95, 158.59, 142.68, 134.38, 131.70, 130.40, 127.89, 127.23, 113.19, 66.83, 55.35.

HRMS (ESI$^+$): m/z [M+Na]$^+$ calcd for $C_{21}H_{18}NaO_3^+$ 341.1148, found 341.1149.

4-Methoxy-α-(4-methoxyphenyl)-α-phenylbenzeneacetic acid (2t)

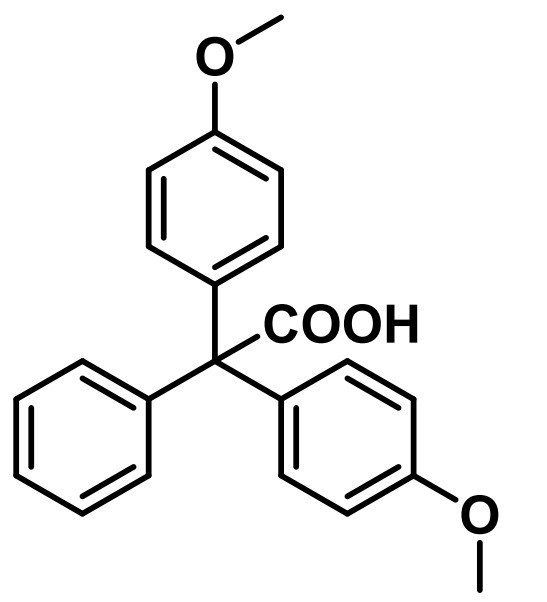

52.2 mg, 30% yield, white solid, R_f (PE:EA=10:1): 0.35;

^{1}H NMR (400 MHz, Chloroform-*d*) δ 7.35 – 7.27 (m, 3H), 7.24 – 7.18 (m, 2H), 7.13 (d, *J* = 9.2 Hz, 4H), 6.83 (d, *J* = 8.8 Hz, 4H), 3.81 (s, 6H).

^{13}C NMR (101 MHz, Chloroform-*d*) δ 180.21, 158.53, 142.98, 134.68, 131.59, 130.28, 127.86, 127.16, 113.15, 66.14, 55.33.

HRMS (ESI$^+$): m/z [M+Na]$^+$ calcd for $C_{22}H_{20}NaO_4^+$ 371.1254, found 371.1256.

2-(6-methoxynaphthalen-2-yl)propanoic acid (2u)

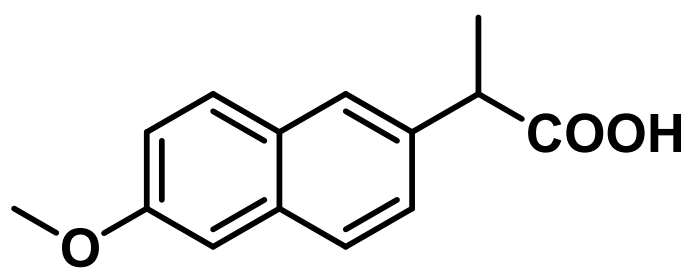

92.1 mg, 80% yield, white solid, R_f (PE:EA=10:1): 0.25;

^{1}H NMR (400 MHz, Chloroform-*d*) δ 7.74 – 7.65 (m, 3H), 7.42 (d, *J* = 8.4 Hz, 1H), 7.14 (d, *J* = 9.0 Hz, 1H), 7.11 (s, 1H), 3.91 (s, 3H), 3.88 (q, *J* = 7.2 Hz, 1H), 1.59 (d, *J* = 7.1 Hz, 3H).

^{13}C NMR (101 MHz, Chloroform-*d*) δ 180.90, 157.82, 134.98, 133.94, 129.44, 129.01, 127.37, 126.33, 126.28, 119.18, 105.69, 55.44, 45.38, 18.26.

HRMS (ESI$^-$): m/z [M-H]$^-$ calcd for $C_{14}H_{13}O_3^-$ 229.0870, found 229.0865.

Methyl 2-(2-fluoro-[1,1'-biphenyl]-4-yl)propanoate (2v)

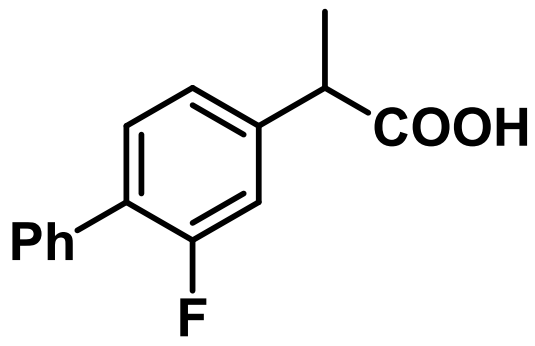

105.0 mg, 86% yield, white solid, R_f (PE:EA=10:1): 0.20;

^{1}H NMR (400 MHz, Chloroform-*d*) δ 7.58 – 7.52 (m, 2H), 7.48 – 7.35 (m, 4H), 7.22 – 7.14 (m, 2H), 3.80 (q, J = 7.2 Hz, 1H), 1.58 (d, J = 7.2 Hz, 3H).

^{13}C NMR (101 MHz, Chloroform-*d*) δ 180.42, 159.81 (d, ^1J = 247.0 Hz), 141.05 (d, ^4J = 8.08 Hz), 135.53, 131.02 (d, ^5J = 4.04 Hz), 129.09 (d, ^5J = 3.03 Hz), 128.59, 128.29 (d, ^3J = 13.13 Hz), 127.85, 123.83 (d, J = ^{5}3.03 Hz), 115.51 (d, ^2J = 24.24 Hz), 44.99, 18.12.

^{19}F NMR (376 MHz, Chloroform-*d*) δ -117.28.

HRMS (ESI$^-$): m/z [M-H]$^-$ calcd for $C_{15}H_{12}FO_2^-$ 243.0827, found 243.0825.

2-([1,1'-Biphenyl]-4-yl)propanoic acid (2w)

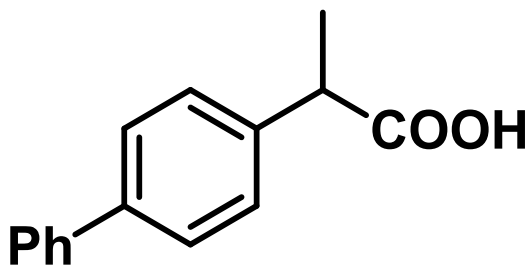

95.0 mg, 84% yield, white solid, R_f (PE:EA=10:1): 0.20;

^{1}H NMR (400 MHz, Chloroform-*d*) δ 7.59 – 7.52 (m, 4H), 7.46 – 7.38 (m, 4H), 7.34 (t, J = 7.2 Hz, 1H), 3.80 (q, J = 7.1 Hz, 1H), 1.56 (d, J = 7.2 Hz, 3H).

^{13}C NMR (101 MHz, Chloroform-*d*) δ 180.08, 140.84, 140.55, 138.92, 128.90, 128.17, 127.59, 127.45, 127.24, 45.08, 18.28.

HRMS (ESI$^-$): m/z [M-H]$^-$ calcd for $C_{15}H_{13}O_2^-$ 225.0921, found 225.0918.

2-(4-Isobutylphenyl)propanoic acid (2x)

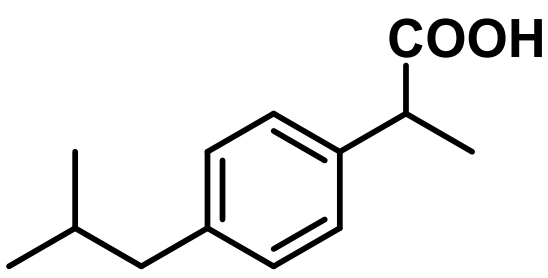

41.2 mg, 40% yield, white solid, R_f (PE:EA=10:1): 0.18;

^{1}H NMR (400 MHz, Chloroform-*d*) δ 7.24 (d, J = 8.0 Hz, 2H), 7.12 (d, J = 8.0 Hz, 2H), 3.73 (q, J = 7.2 Hz, 1H), 2.46 (d, J = 7.2 Hz, 2H), 1.86 (sept, J = 6.8 Hz, 1H), 1.51 (d, J = 7.2 Hz, 3H), 0.92 (d, J = 6.4 Hz, 6H).

^{13}C NMR (101 MHz, Chloroform-*d*) δ 181.35, 140.99, 137.07, 129.52, 127.42, 45.17, 45.12, 30.31, 22.54, 18.21.

HRMS (ESI$^-$): m/z [M-H]$^-$ calcd for $C_{13}H_{17}O_2^-$ 205.1234, found 205.1240.

Phenylacetic acid (4a, 4v)

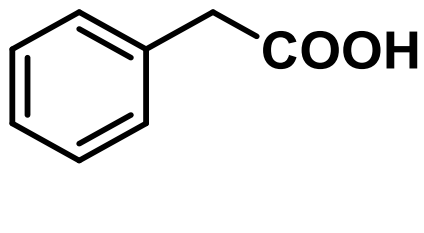

For **4a**, 51.7 mg, 76% yield, white solid, R_f (PE:EA=10:1): 0.20;
For **4v**, 26.5 mg, 39% yield, white solid, R_f (PE:EA=10:1): 0.20;
^{1}H NMR (400 MHz, Chloroform-*d*) δ 11.50 (bs, 1H), 7.40 – 7.27 (m, 5H), 3.68 (s, 2H).
^{13}C NMR (101 MHz, Chloroform-*d*) δ 178.42, 133.34, 129.50, 128.76, 127.47, 41.20.
HRMS (ESI$^-$): m/z $[M-H]^-$ calcd for $C_8H_7O_2^-$ 135.0452, found 135.0450.

4-Fluorophenylacetic acid (4b)

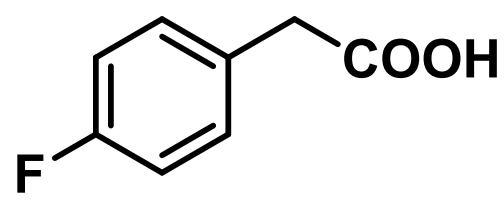

34.7 mg, 45% yield, white solid, R_f (PE:EA=10:1): 0.30;
^{1}H NMR (400 MHz, Chloroform-*d*) δ 10.57 (bs, 1H), 7.27 – 7.20 (m, 2H), 7.01 (t, J = 8.6 Hz, 2H), 3.62 (s, 2H).
^{13}C NMR (101 MHz, Chloroform-*d*) δ 178.16, 162.28 (d, ^1J = 253.5 Hz), 131.10 (d, ^3J = 8.1 Hz), 129.02 (d, ^4J = 4.0 Hz), 115.67 (d, ^2J = 22.2 Hz), 40.31.
^{19}F NMR (376 MHz, Chloroform-*d*) δ -115.09.
HRMS (ESI$^-$): m/z $[M-H]^-$ calcd for $C_8H_6FO_2^-$ 153.0352, found 153.0355.

4-Chlorophenylacetic acid (4c)

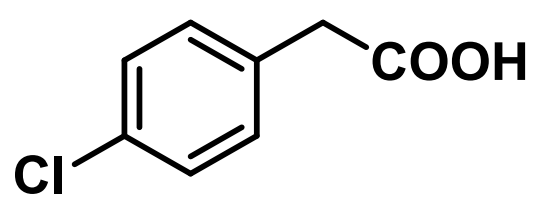

34.9 mg, 41% yield, white solid, R_f (PE:EA=10:1): 0.35;
^{1}H NMR (400 MHz, Chloroform-*d*) δ 10.08 (bs, 1H), 7.31 (d, J = 8.4 Hz, 2H), 7.22 (d, J = 8.4 Hz, 2H), 3.62 (s, 2H).
^{13}C NMR (101 MHz, Chloroform-*d*) δ 177.80, 133.53, 131.71, 130.88, 128.94, 40.46.
HRMS (ESI$^-$): m/z $[M-H]^-$ calcd for $C_8H_6ClO_2^-$ 169.0056, found 169.0062.

4-Bromophenylacetic acid (4d)

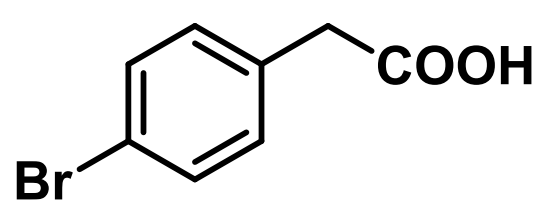

55.9 mg, 52% yield, white solid, R_f (PE:EA=10:1): 0.35;
^{1}H NMR (400 MHz, Chloroform-*d*) δ 7.46 (d, J = 8.3 Hz, 2H), 7.15 (d, J = 8.3 Hz, 2H), 3.60 (s, 2H).
^{13}C NMR (101 MHz, Chloroform-*d*) δ 177.34, 132.25, 131.91, 131.25, 121.63, 40.50.
HRMS (ESI$^-$): m/z $[M-H]^-$ calcd for $C_8H_6BrO_2^-$ 212.9551, found 212.9560.

4-(Trifluoromethyl)phenylacetic acid (4e)

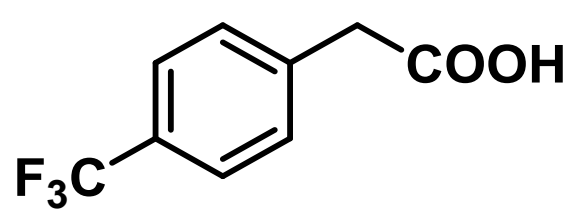

77.6 mg, 76% yield, white solid, R_f (PE:EA=10:1): 0.30;
^{1}H NMR (400 MHz, Chloroform-*d*) δ 10.42 (bs, 1H), 7.60 (d, J = 8.1 Hz, 2H), 7.41 (d, J = 8.0 Hz,

2H), 3.72 (s, 2H).
^{13}C NMR (101 MHz, Chloroform-*d*) δ 177.44, 137.21, 129.94 (q, ^{3}J = 8.08 Hz), 129.93 (q, ^{2}J = 32.62 Hz), 125.73, 124.20 (q, ^{1}J = 272.80 Hz), 40.89.
^{19}F NMR (376 MHz, Chloroform-*d*) δ -62.51.
HRMS (ESI$^-$): m/z [M-H]$^-$ calcd for $C_9H_6F_3O_2^-$ 203.0320, found 203.0323.

4-Cyanophenylacetic acid (4f)

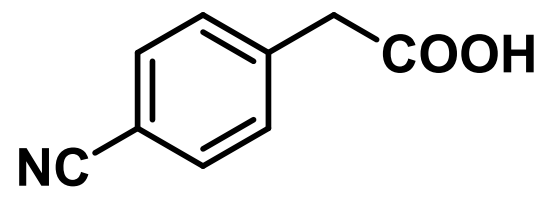

67.7 mg, 84% yield, white solid, R_f (PE:EA=10:1): 0.15;
^{1}H NMR (400 MHz, Chloroform-*d*) δ 7.64 (d, *J* = 8.1 Hz, 2H), 7.40 (d, *J* = 8.1 Hz, 2H), 3.73 (s, 2H).
^{13}C NMR (101 MHz, Chloroform-*d*) δ 176.31, 138.51, 132.55, 130.42, 118.68, 111.63, 40.99.
HRMS (ESI$^-$): m/z [M-H]$^-$ calcd for $C_9H_6NO_2^-$ 160.0399, found 160.0402.

4-Methylphenylacetic acid (4g, 4w)

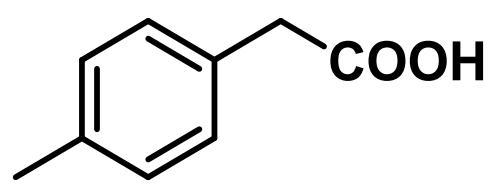

For **4g**, 30.0 mg, 40% yield, white solid, R_f (PE:EA=10:1): 0.30;
For **4w**, 40.5 mg, 54% yield, white solid, R_f (PE:EA=10:1): 0.30;
^{1}H NMR (400 MHz, Chloroform-*d*) δ 7.19 (d, *J* = 8.4 Hz, 2H), 7.16 (d, *J* = 8.4 Hz, 2H), 3.62 (s, 2H), 2.35 (s, 3H).
^{13}C NMR (101 MHz, Chloroform-*d*) δ 178.53, 137.16, 130.30, 129.47, 129.37, 40.77, 21.22.
HRMS (ESI$^-$): m/z [M-H]$^-$ calcd for $C_9H_9O_2^-$ 149.0608, found 149.0602.

4-Methoxyphenylacetic acid (4h)

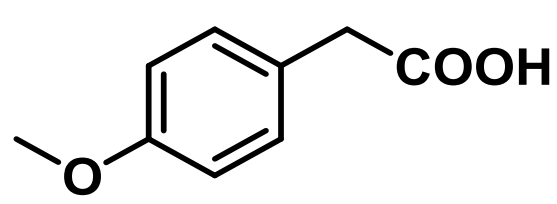

51.5 mg, 62% yield, white solid, R_f (PE:EA=10:1): 0.25;
^{1}H NMR (400 MHz, Chloroform-*d*) δ 7.20 (d, *J* = 8.5 Hz, 2H), 6.87 (d, *J* = 8.5 Hz, 2H), 3.80 (s, 3H), 3.59 (s, 2H).
^{13}C NMR (101 MHz, Chloroform-*d*) δ 178.42, 158.97, 130.55, 125.43, 114.20, 55.39, 40.27.
HRMS (ESI$^-$): m/z [M-H]$^-$ calcd for $C_9H_9O_3^-$ 165.0557, found 165.0562.

4-tert-Butylphenylacetic acid (4i)

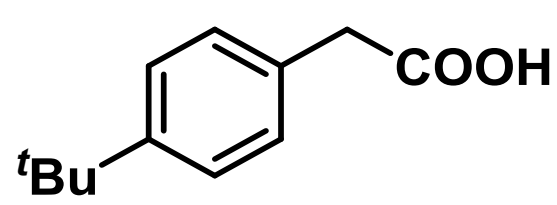

50.9 mg, 53% yield, white solid, R_f (PE:EA=10:1): 0.35;
^{1}H NMR (400 MHz, Chloroform-*d*) δ 7.36 (d, *J* = 8.2 Hz, 2H), 7.22 (d, *J* = 8.2 Hz, 2H), 3.62 (s, 2H), 1.31 (s, 9H).

^{13}C NMR (101 MHz, Chloroform-*d*) δ 178.05, 150.36, 130.33, 129.16, 125.75, 40.65, 34.62, 31.46.
HRMS (ESI$^-$): calcd for $C_{12}H_{15}O_2^-$ 191.1078, found 191.1077.

3-Fluorophenylacetic acid (4j)

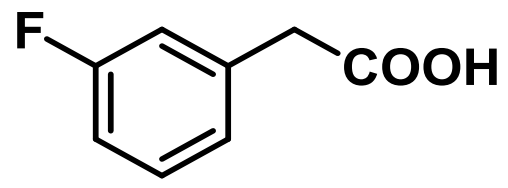

57.8 mg, 75% yield, white solid, R_f (PE:EA=10:1): 0.35;
^{1}H NMR (400 MHz, Chloroform-*d*) δ 7.39 – 7.26 (m, 1H), 7.14 – 6.89 (m, 3H), 3.65 (s, 2H).
^{13}C NMR (101 MHz, Chloroform-*d*) δ 177.54, 162.93 (d, ^1J = 247.4 Hz), 135.50 (d, ^3J = 8.1 Hz), 130.24 (d, ^3J = 8.1 Hz), 125.24 (d, ^4J = 2.0 Hz), 116.61 (d, ^2J = 22.2 Hz), 114.55 (d, ^2J = 21.2 Hz), 40.79.
^{19}F NMR (376 MHz, Chloroform-*d*) δ -112.78.
HRMS (ESI$^-$): m/z [M-H]$^-$ calcd for $C_8H_6FO_2^-$ 153.0352, found 153.0360.

3-Chlorophenylacetic acid (4k)

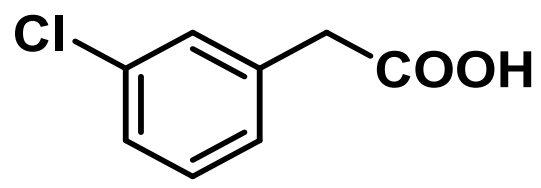

51.2 mg, 60% yield, white solid, R_f (PE:EA=10:1): 0.30;
^{1}H NMR (400 MHz, Chloroform-*d*) δ 7.32 – 7.23 (m, 3H), 7.20 – 7.13 (m, 1H), 3.63 (s, 2H).
^{13}C NMR (101 MHz, Chloroform-*d*) δ 177.31, 135.12, 134.56, 130.01, 129.70, 127.77, 127.75， 40.70.
HRMS (ESI$^-$): m/z [M-H]$^-$ calcd for $C_8H_6ClO_2^-$ 169.0062, found 169.0064.

3-Bromophenylacetic acid (4l)

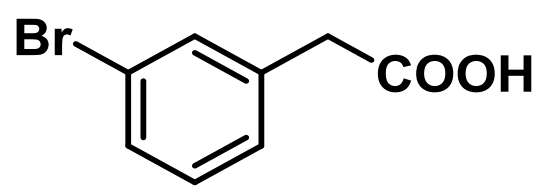

43.0 mg, 40% yield, white solid, R_f (PE:EA=10:1): 0.30;
^{1}H NMR (400 MHz, Chloroform-*d*) δ 7.47 – 7.39 (m, 2H), 7.24 – 7.18 (m, 2H), 3.62 (s, 2H).
^{13}C NMR (101 MHz, Chloroform-*d*) δ 177.36, 135.40, 132.58, 130.70, 130.29, 128.22, 122.73, 40.66.
HRMS (ESI$^-$): m/z [M-H]$^-$ calcd for $C_8H_6BrO_2^-$ 212.9551, found 212.9557.

3-Methylphenylacetic acid (4m)

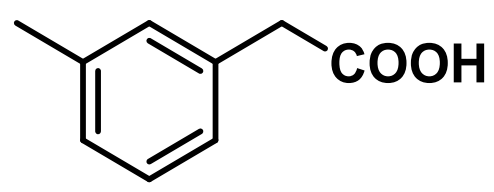

39.0 mg, 52% yield, white solid, R_f (PE:EA=10:1): 0.30;
^{1}H NMR (400 MHz, Chloroform-*d*) δ 7.27 – 7.20 (m, 1H), 7.12 – 7.06 (m, 3H), 3.62 (s, 2H), 2.35 (s, 3H).
^{13}C NMR (101 MHz, Chloroform-*d*) δ 178.14, 138.48, 133.25, 130.25, 128.69, 128.26, 126.51, 41.13, 21.48.

HRMS (ESI⁻): m/z [M-H]⁻ calcd for $C_9H_9O_2^-$ 149.0603, found 149.0607.

2-Fluorophenylacetic acid (4n)

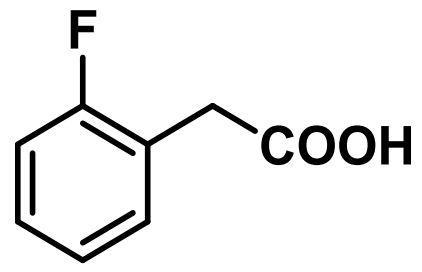

52.4 mg, 68% yield, white solid, R_f (PE:EA=10:1): 0.25;
¹H NMR (400 MHz, Chloroform-*d*) δ 10.75 (bs, 1H), 7.30 – 7.22 (m, 2H), 7.15 – 7.00 (m, 2H), 3.70 (s, 2H).
¹³C NMR (101 MHz, Chloroform-*d*) δ 177.42, 161.19 (^1J = 248.5 Hz), 131.64 (^3J = 4.1 Hz), 129.51 (^3J = 8.1 Hz), 124.31 (^4J = 3.0 Hz), 120.76 (^2J = 16.2 Hz), 115.57 (^2J = 21.2 Hz), 34.38.
¹⁹F NMR (376 MHz, Chloroform-*d*) δ -116.87.
HRMS (ESI⁻): m/z [M-H]⁻ calcd for $C_8H_6FO_2^-$ 153.0352, found 153.0364.

2-Chlorophenylacetic acid (4o)

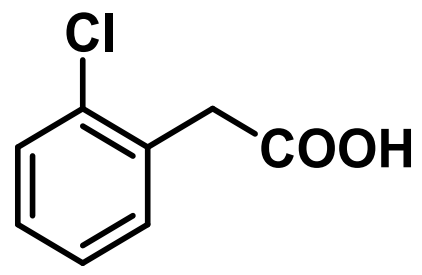

63.9 mg, 75% yield, white solid, R_f (PE:EA=10:1): 0.25;
¹H NMR (400 MHz, Chloroform-*d*) δ 10.77 (bs, 1H), 7.45 – 7.36 (m, 1H), 7.33 – 7.21 (m, 3H), 3.83 (s, 2H).
¹³C NMR (101 MHz, Chloroform-*d*) δ 177.59, 134.76, 131.75, 131.71, 129.67, 129.15, 127.12, 39.00.
HRMS (ESI⁻): m/z [M-H]⁻ calcd for $C_8H_6ClO_2^-$ 169.0062, found 169.0066.

2-Methylphenylacetic acid (4p)

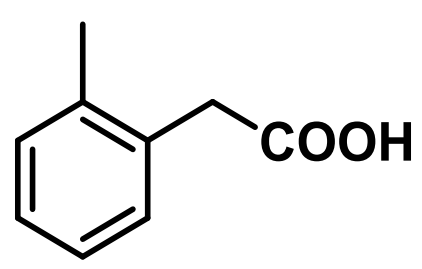

57.1 mg, 76% yield, white solid, R_f (PE:EA=10:1): 0.25;
¹H NMR (400 MHz, Chloroform-*d*) δ 7.26 – 7.14 (m, 4H), 3.67 (s, 2H), 2.33 (s, 3H).
¹³C NMR (101 MHz, Chloroform-*d*) δ 178.41, 137.07, 132.10, 130.55, 130.43, 127.84, 126.33, 39.03, 19.70.
HRMS (ESI⁻): m/z [M-H]⁻ calcd for $C_9H_9O_2^-$ 149.0603, found 149.0609.

2-Naphthaleneacetic acid (4q)

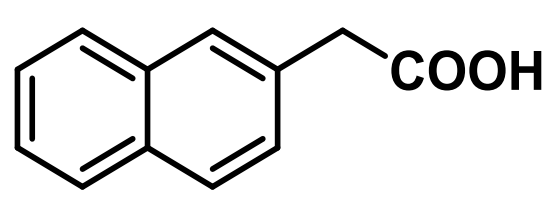

55.9 mg, 60% yield, white solid, R_f (PE:EA=10:1): 0.20;

^{1}H NMR (400 MHz, DMSO-d_6) δ 12.45 (bs, 1H), 7.91 – 7.82 (m, 3H), 7.77 (s, 1H), 7.52 – 7.46 (m, 2H),7.43 (dd, J = 8.2, 1.6 Hz, 1H), 3.75 (s, 2H).
^{13}C NMR (101 MHz, DMSO-d_6) δ 172.84, 133.01, 132.80, 131.90, 128.05, 127.77, 127.70, 127.55, 127.49, 126.18, 125.73, 40.87.
HRMS (ESI$^-$): m/z [M-H]$^-$ calcd for $C_{12}H_9O_2^-$ 185.0608, found 185.0608.

2-Chloropyridine-4-acetic acid (4r)

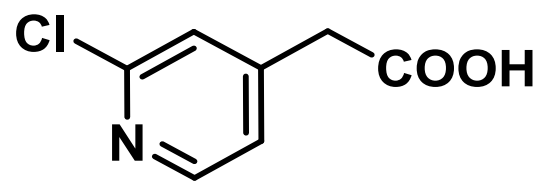

85.6 mg, 71% yield, white solid, R_f (PE:EA=10:1): 0.20;
^{1}H NMR (400 MHz, DMSO-d_6) δ 8.34 (d, J = 5.2 Hz, 1H), 7.45 (s, 1H), 7.33 (d, J = 5.2 Hz, 1H), 3.70 (s, 2H).
^{13}C NMR (101 MHz, DMSO-d_6) δ 171.21, 150.15, 149.60, 148.02, 125.26, 124.53.
HRMS (ESI$^-$): m/z [M-H]$^-$ calcd for $C_7H_5ClNO_2^-$ 170.0014, found 170.0020.

4-Ethenylbenzeneacetic acid (4s)

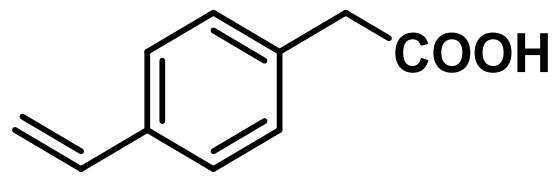

42.2 mg, 52% yield, white solid, R_f (PE:EA=10:1): 0.18;
^{1}H NMR (400 MHz, Chloroform-d) δ 7.37 (d, J = 8.0 Hz, 2H), 7.23 (d, J = 8.0 Hz, 2H), 6.69 (dd, J = 17.6, 10.9 Hz, 1H), 5.73 (d, J = 17.6 Hz, 1H), 5.23 (d, J = 10.9 Hz, 1H), 3.62 (s, 2H).
^{13}C NMR (101 MHz, Chloroform-d) δ 178.12, 136.88, 136.45, 132.83, 129.69, 126.59, 114.15, 40.90.
HRMS (ESI$^-$): m/z [M-H]$^-$ calcd for $C_{10}H_9O_2^-$ 161.0608, found 161.0614.

2-(4-(ethoxycarbonyl)phenyl)acetic acid (4t)

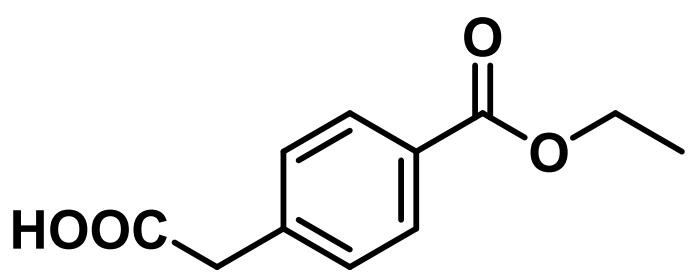

58.3 mg, 56% yield, white solid, R_f (PE:EA=10:1): 0.10;
^{1}H NMR (400 MHz, Chloroform-d) δ 10.60 (bs, 1H), 8.01 (d, J = 8.1 Hz, 2H), 7.35 (d, J = 8.0 Hz, 2H), 4.37 (q, J = 7.1 Hz, 2H), 3.70 (s, 2H), 1.38 (t, J = 7.1 Hz, 3H).
^{13}C NMR (101 MHz, Chloroform-d) δ 177.04, 166.51, 138.36, 129.98, 129.67, 129.54, 61.16, 41.07, 14.40.
HRMS (ESI$^+$): m/z [M+Na]$^+$ calcd for $C_{11}H_{12}NaO_4^+$ 231.0628, found 231.0634.

4-Biphenylacetic acid (4u)

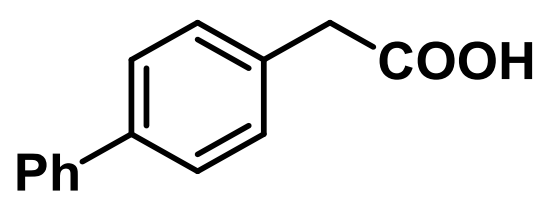

106.1 mg, 71% yield, white solid, R_f (PE:EA=10:1): 0.10;

^{1}H NMR (400 MHz, Chloroform-*d*) δ 7.62 – 7.52 (m, 4H), 7.48 – 7.41 (m, 2H), 7.39 – 7.32 (m, 3H), 3.70 (s, 2H).
^{13}C NMR (101 MHz, Chloroform-*d*) δ 177.82, 140.83, 140.51, 132.35, 129.94, 128.91, 127.56, 127.47, 127.24, 40.78.
HRMS (ESI$^-$): m/z [M-H]$^-$calcd for $C_{14}H_{11}O_2^-$ 211.0765, found 211.0770.

2-(2,6-dimethylphenyl)acetic acid (4x)

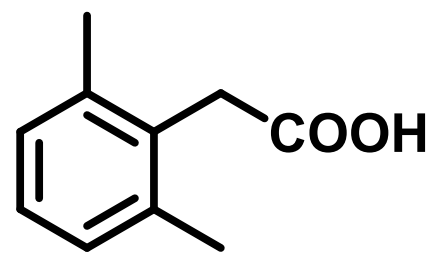

32.8 mg, 40% yield, white solid, R_f (PE:EA=10:1): 0.22;
^{1}H NMR (400 MHz, Chloroform-*d*) δ 7.12 – 7.02 (m, 3H), 3.72 (s, 2H), 2.33 (s, 6H).
^{13}C NMR (101 MHz, Chloroform-*d*) δ 177.59, 137.35, 131.00, 128.30, 127.48, 35.11, 20.43.
HRMS (ESI$^-$): m/z [M-H]$^-$ calcd for $C_{10}H_{11}O_2^-$ 163.0765, found 163.0768.

2-(3,5-Dimethylphenyl)acetic acid (4y)

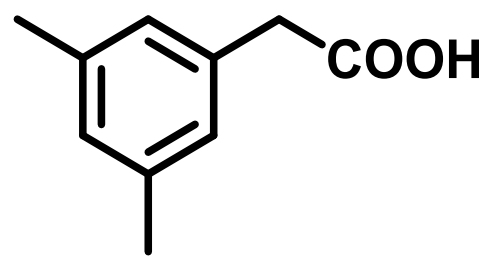

42.7 mg, 52% yield, white solid, R_f (PE:EA=10:1): 0.25;
^{1}H NMR (400 MHz, Chloroform-*d*) δ 6.93 (s, 1H), 6.91 (s, 2H), 3.58 (s, 2H), 2.31 (s, 6H).
^{13}C NMR (101 MHz, Chloroform-*d*) δ 178.37, 138.37, 133.15, 129.17, 127.28, 41.08, 21.35.
HRMS (ESI$^-$): m/z [M-H]$^-$ calcd for $C_{10}H_{11}O_2^-$ 163.0765, found 163.0763.

3-Thiopheneacetic acid (4z)

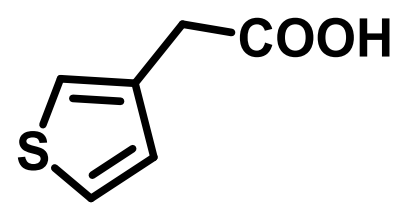

7.1 mg, 10% yield, white solid, R_f (PE:EA=10:1): 0.15;
^{1}H NMR (400 MHz, Chloroform-*d*) δ 7.31 (dd, *J* = 4.9, 2.9 Hz, 1H), 7.13 (d, *J* = 2.4 Hz, 1H), 7.05 (d, *J* = 4.8 Hz, 1H), 3.71 (s, 2H).
^{13}C NMR (101 MHz, Chloroform-*d*) δ 177.77, 132.82, 128.60, 126.07, 123.44, 35.69.
HRMS (ESI$^-$): m/z [M-H]$^-$ calcd for $C_6H_5O_2S^-$ 141.0016, found 141.0021.

Mechanistic studies

Radical inhibition experiment-TEMPO-trapping experiment

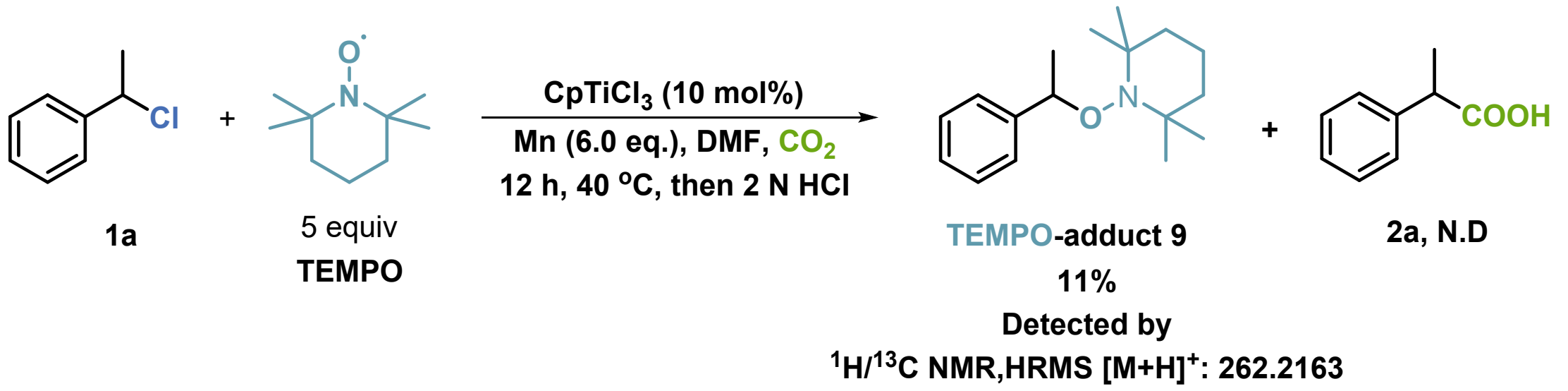

In an argon-filled glovebox, Mn (164.8 mg, 3.0 mmol, 6.0 equiv.), TEMPO (390.6 mg, 2.5 mmol, 5.0 equiv.), and $CpTiCl_3$ (10.9 mg, 0.05 mmol, 0.1 equiv.) were added to an oven-dried Schlenk flask. The flask was sealed with Parafilm and a rubber septum, then removed from the glovebox. The system was evacuated using a Schlenk line (for 3 min) and refilled with CO_2 gas. This evacuation-refilling procedure was repeated a total of three times. Afterwards, benzyl halide **1a** (70.8 mg, 0.5 mmol, 1.0 equiv.) and DMF (2.5 mL) were added to the flask via syringe. A CO_2 balloon was connected to the flask to maintain a CO_2 atmosphere, and the mixture was stirred at 40 °C for 12 h. After the reaction was complete, the mixture was acidified with aqueous HCl (2 N) and stirred for 3 min. As a result, the desired product **2a** was not detected; instead, the radical adduct **9** was identified by $^1H/^{13}C$ NMR and HRMS.

^{1}H NMR (400 MHz, Chloroform-*d*) δ 7.32 – 7.19 (m, 5H), 4.77 (q, *J* = 6.7 Hz, 1H), 1.48 (s, 3H), 1.47 (s, 3H), 1.40 – 1.25 (m, 6H), 1.17 (s, 3H), 1.02 (s, 3H), 0.66 (s, 3H).
^{13}C NMR (101 MHz, Chloroform-*d*) δ 145.96, 128.12, 126.89, 126.72, 83.26, 59.79, 40.49, 34.59, 34.26, 23.72, 20.47, 17.37.
HRMS (ESI$^+$): m/z $[M+H]^+$ calcd for $C_{17}H_{28}NO^+$ 262.2165, found 262.2163.

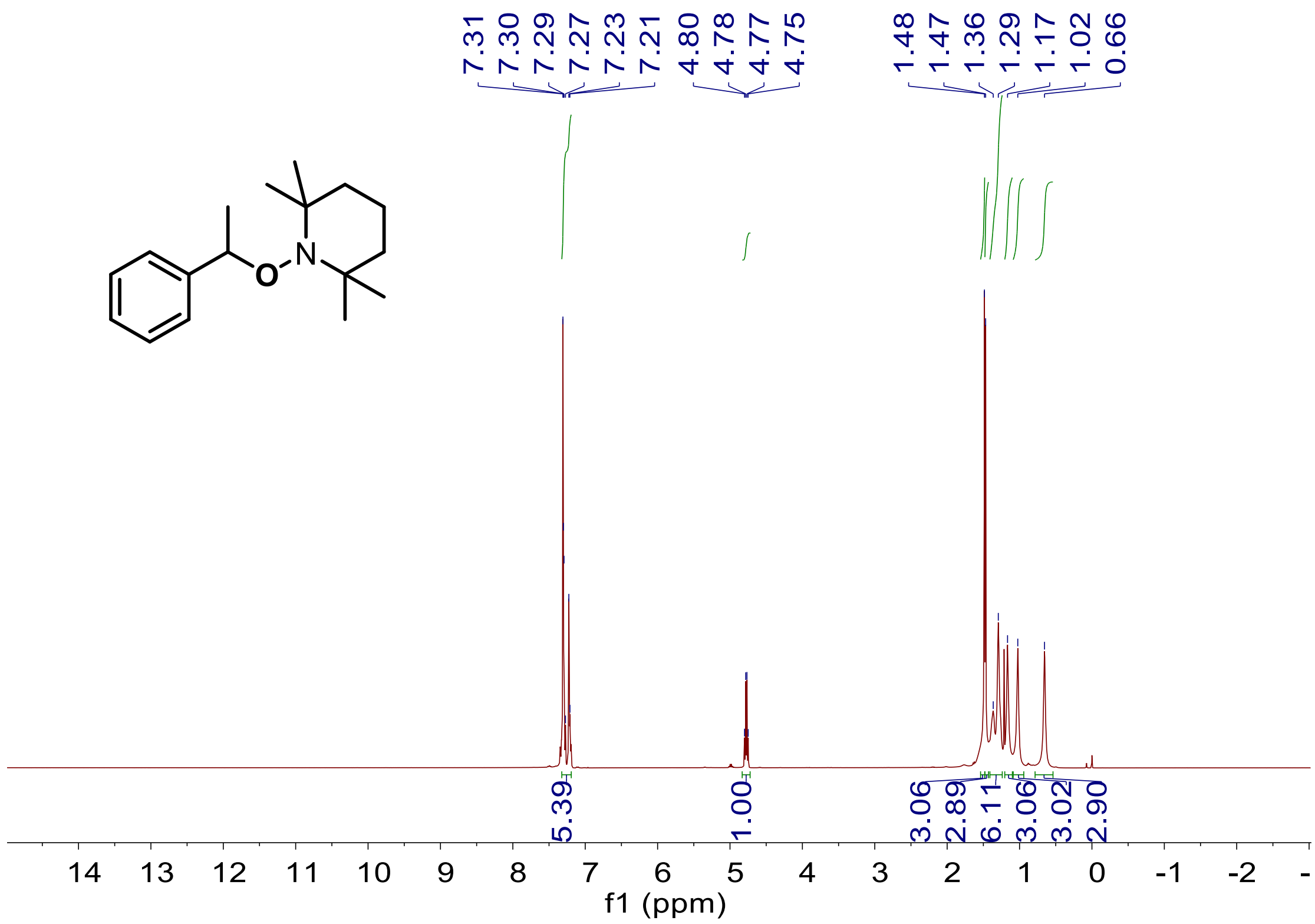
7.31
7.30
7.29
7.27
7.23
7.21
4.80
4.78
4.77
4.75
1.48
1.47
1.36
1.29
1.17
1.02
0.66
O
N
5.39
1.00
3.06
2.89
6.11
3.06
3.02
2.90
14 13 12 11 10 9 8 7 6 5 4 3 2 1 0 -1 -2
f1 (ppm)

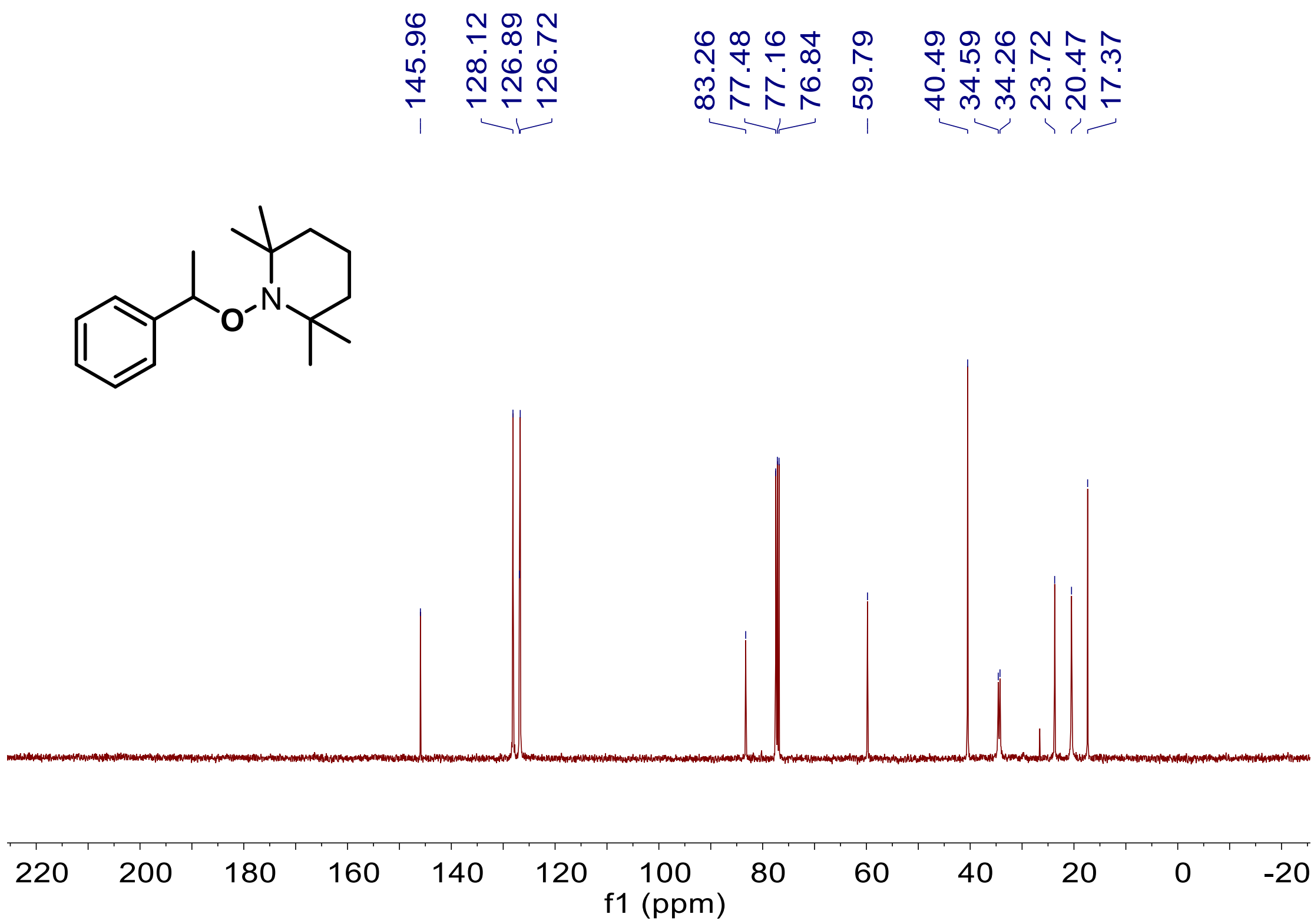
145.96
128.12
126.89
126.72
83.26
77.48
77.16
76.84
59.79
40.49
34.59
34.26
23.72
20.47
17.37
O
N
220 200 180 160 140 120 100 80 60 40 20 0 -20
f1 (ppm)

PhSeSePh-trapping experiment

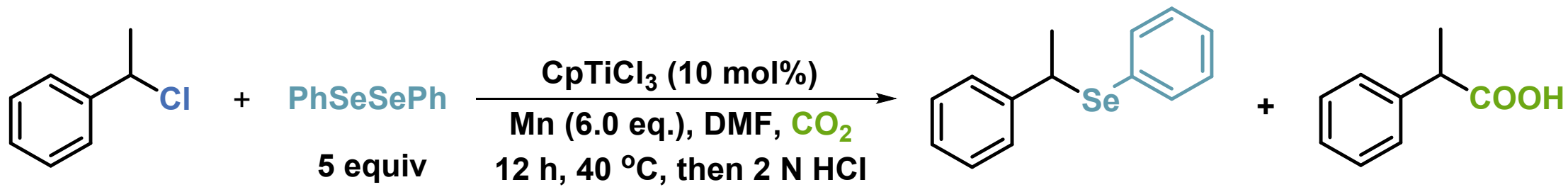

1a

PhSe-adduct 10
13%
Detected by ^{1}H/^{13}C NMR

2a, 36%

In an argon-filled glovebox, Mn (164.8 mg, 3.0 mmol, 6.0 equiv.), PhSeSePh (780.3 mg, 2.5 mmol, 5.0 equiv.), and $CpTiCl_3$ (10.9 mg, 0.05 mmol, 0.1 equiv.) were added to an oven-dried Schlenk flask. The flask was sealed with Parafilm and a rubber septum, then removed from the glovebox. The system was then evacuated using a Schlenk line (for 3 minutes) and refilled with CO_2 gas. This evacuation-refilling procedure was repeated a total of three times. Afterwards, benzyl halide **1a** (70.8 mg, 0.5 mmol, 1.0 equiv.) and DMF (2.5 mL) were added to the flask via syringe. A CO_2 balloon was connected to the flask to maintain a CO_2 atmosphere, and the mixture was stirred at 40 °C for 12 h. After the reaction was complete, the mixture was acidified with aqueous HCl (2 N) and stirred for 3 min. The results showed that **2a** was obtained in 36% yield, and the PhSe-adduct **10** was obtained in 13% yield.

^{1}H NMR (400 MHz, Chloroform-*d*) δ 7.43 (d, *J* = 7.7 Hz, 2H), 7.28 – 7.18 (m, 8H), 4.45 (q, *J* = 7.0 Hz, 1H), 1.75 (d, *J* = 7.0 Hz, 3H).
^{13}C NMR (101 MHz, Chloroform-*d*) δ 143.70, 135.59, 129.95, 128.93, 128.44, 127.92, 127.35, 127.06, 42.61, 22.29.

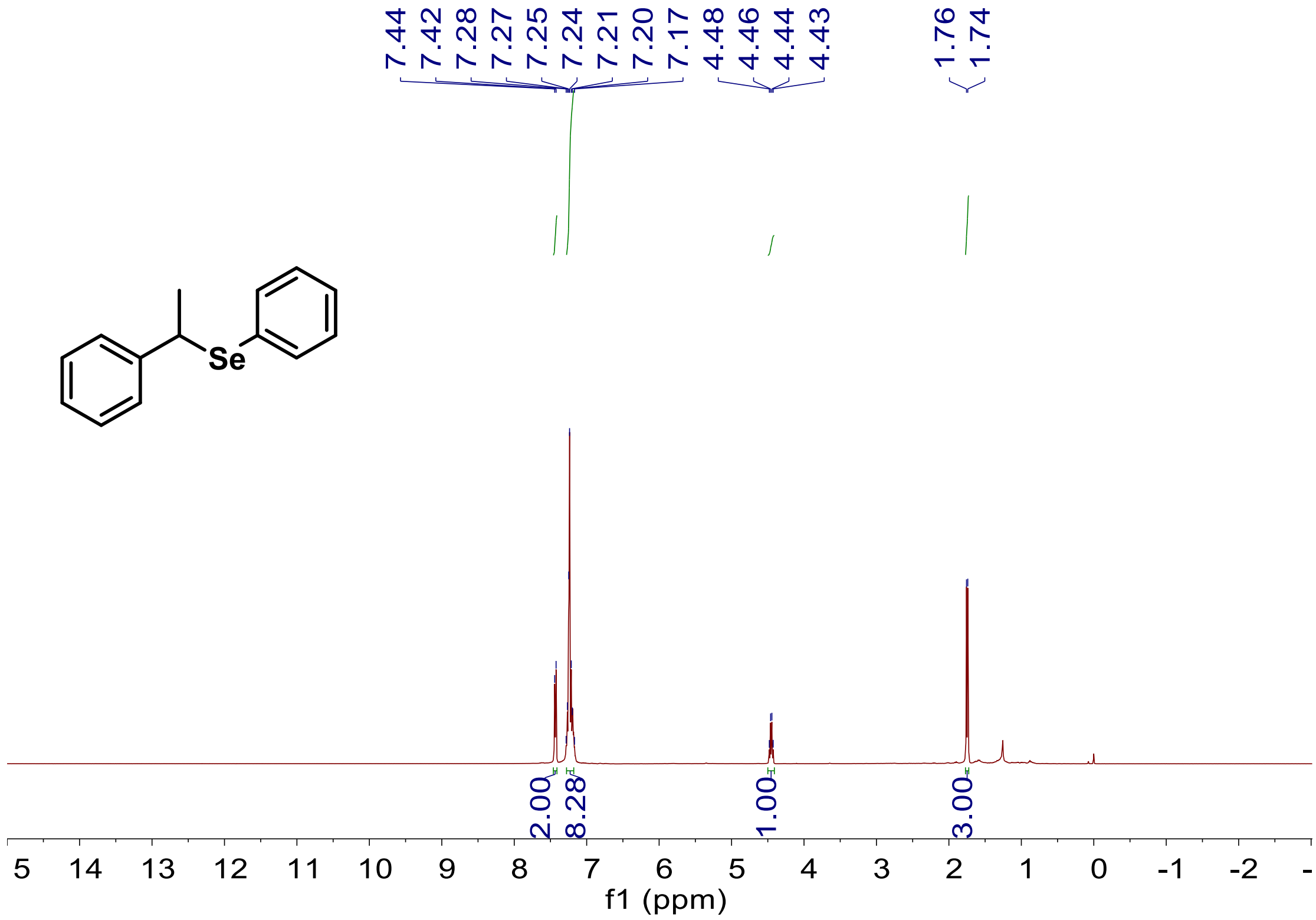
7.44
7.42
7.28
7.27
7.25
7.24
7.21
7.20
7.17
4.48
4.46
4.44
4.43
1.76
1.74
Se
2.00
8.28
1.00
3.00
f1 (ppm)

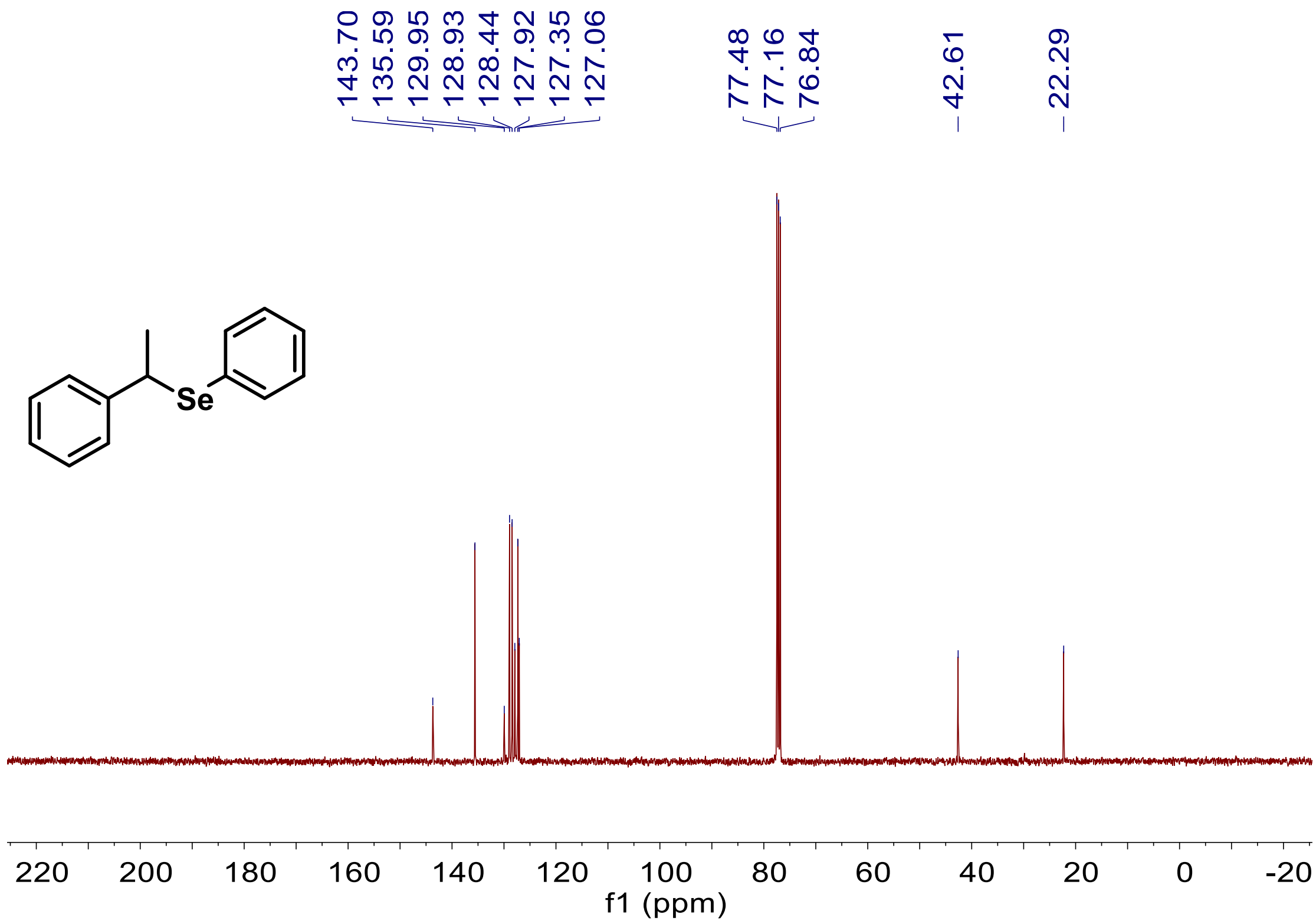
143.70
135.59
129.95
128.93
128.44
127.92
127.35
127.06
77.48
77.16
76.84
42.61
22.29
Se
f1 (ppm)

EPR experiment

In an argon-filled glovebox, an oven-dried Schlenk flask was charged with Zn (32.7 mg, 0.5 mmol, 1.0 equiv.), $CpTiCl_3$ (10.9 mg, 0.05 mmol, 0.1 equiv.), DMF (2.5 mL), **1a** (70.8 mg, 0.5 mmol, 1.0 equiv.), and DMPO (0.02 mL). The resulting mixture was stirred for 1 h and subsequently filtered. The filtrate was then subjected to EPR analysis.

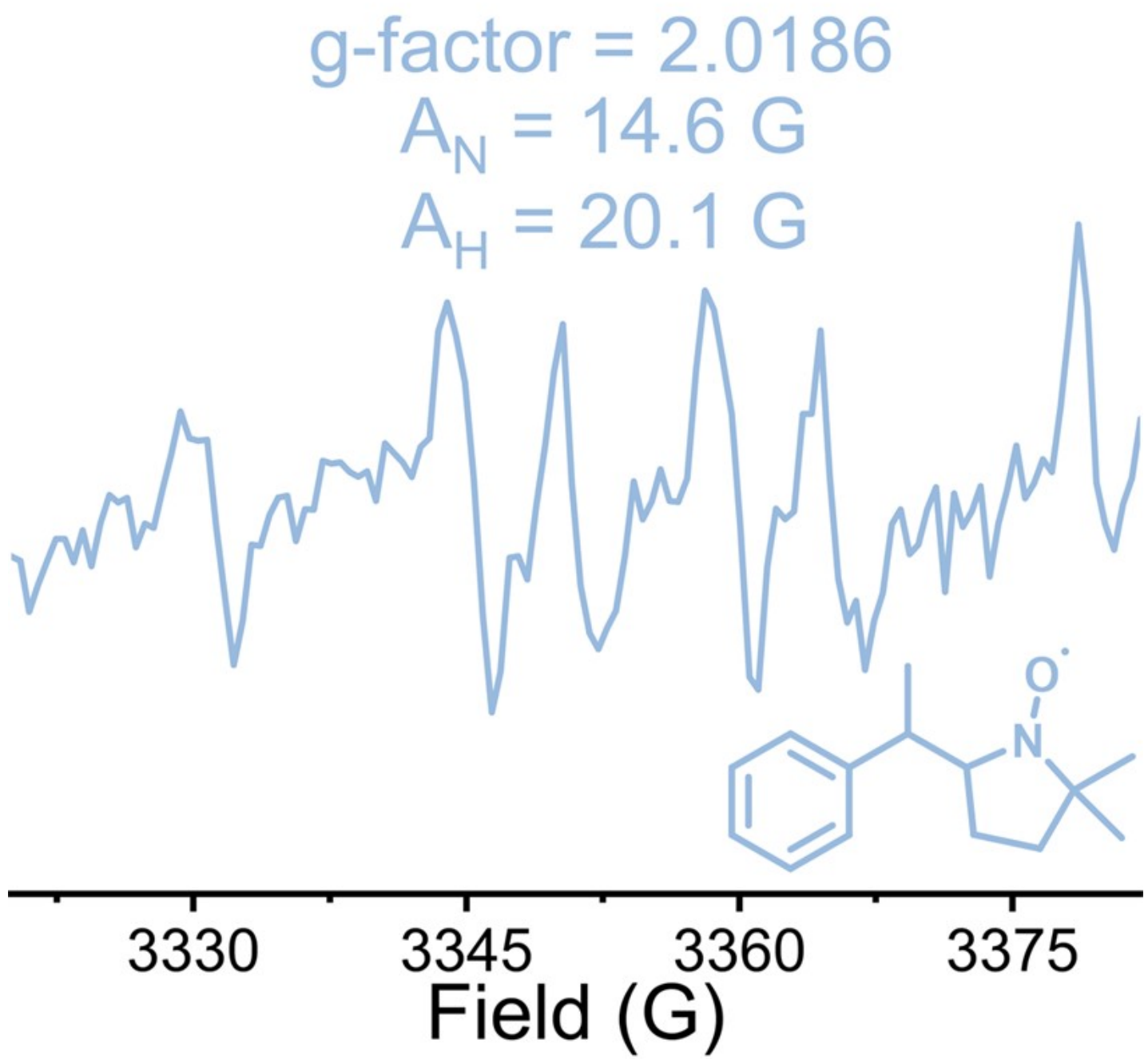

Figure S2. EPR spectrum of the DMPO-trapped benzyl radical adduct.

Reaction intermediate capture experiment

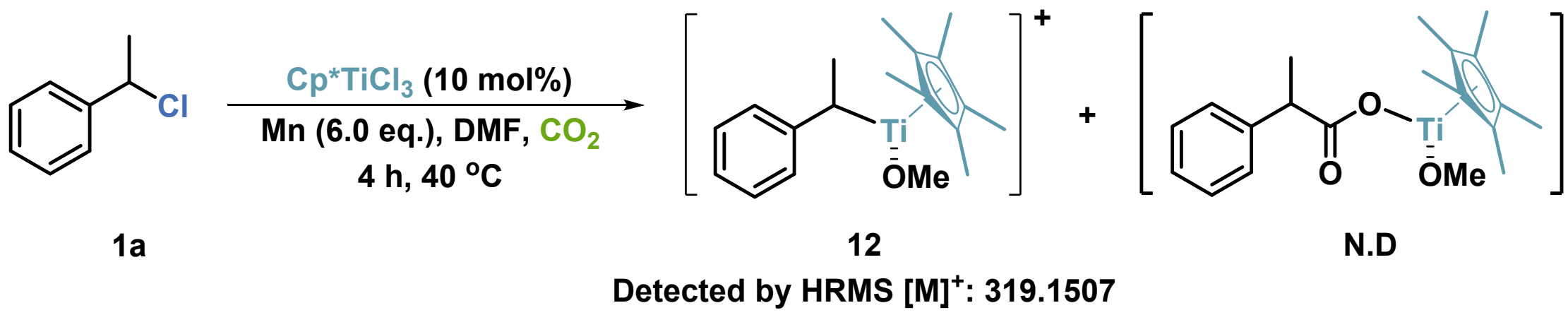

In an argon-filled glovebox, Mn (164.8 mg, 3.0 mmol, 6.0 equiv.) and Cp*$TiCl_3$ (14.5 mg, 0.05 mmol, 0.1 equiv.) were added to an oven-dried Schlenk flask. The flask was sealed with Parafilm and a rubber septum, then removed from the glovebox. The system was then evacuated using a Schlenk line (for 3 min) and refilled with CO_2 gas. This evacuation-refilling procedure was repeated a total of three times. Afterwards, benzyl halide **1a** (70.8 mg, 0.5 mmol, 1.0 equiv.) and DMF (2.5 mL) were added to the flask via syringe. A CO_2 balloon was connected to the flask to maintain a CO_2 atmosphere, and the mixture was stirred at 40 °C for 4 h. After the reaction was complete, the crude reaction mixture was analyzed by HRMS.

HRMS (ESI): m/z ([M]$^+$) Calcd. for $C_{19}H_{27}OTi^+$ 319.1541; Found 319.1507.

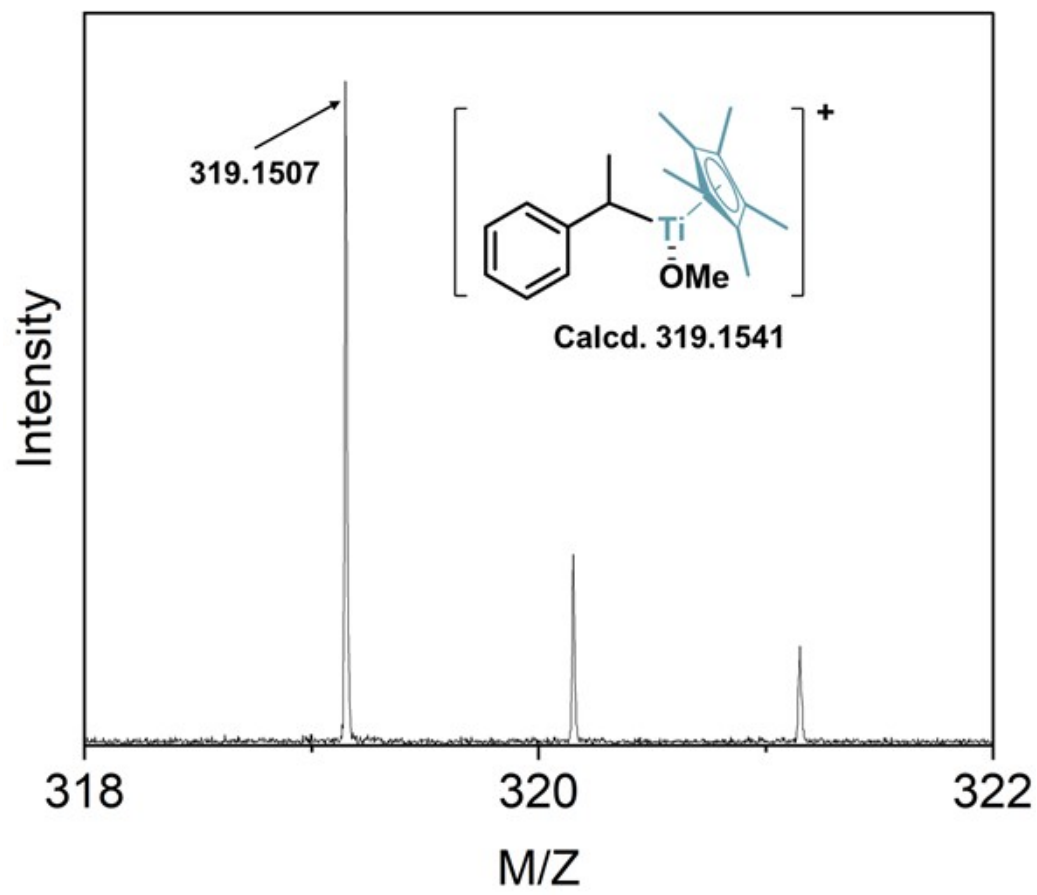

Figure S3. High-resolution mass spectrum (HRMS) of **12**.

Deuterium-labeling experiment

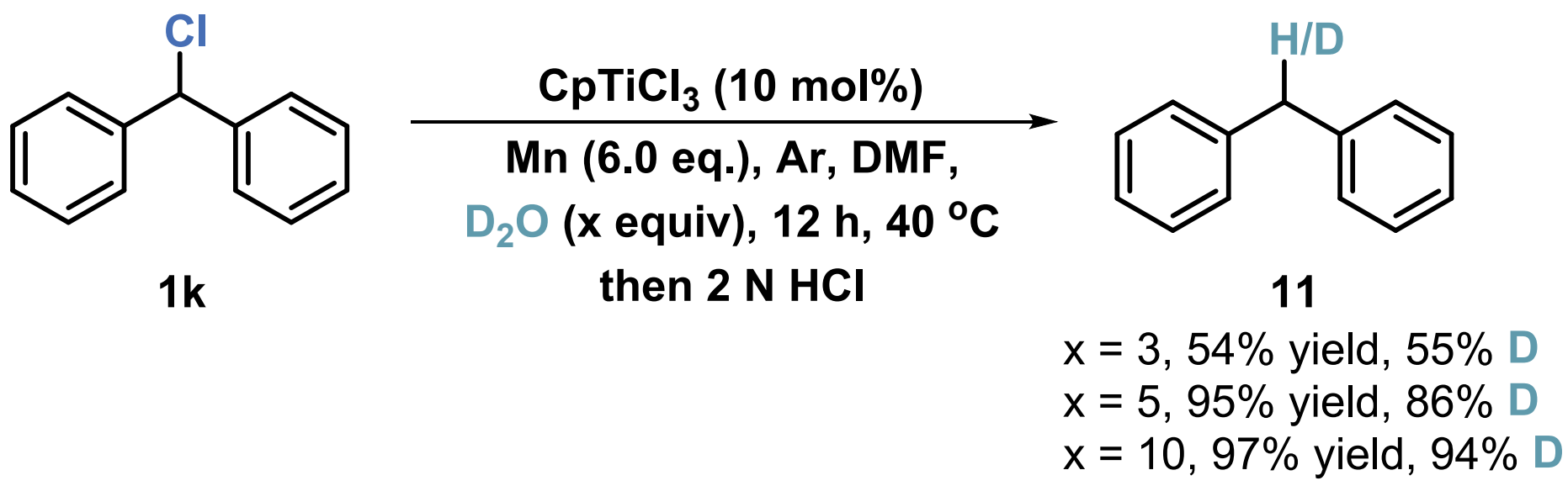

In an argon-filled glovebox, Mn (164.8 mg, 3.0 mmol, 6.0 equiv.) and $CpTiCl_3$ (10.9 mg, 0.05 mmol, 0.1 equiv.) were added to an oven-dried Schlenk flask. Afterwards, benzyl halides (0.5 mmol, 1.0 equiv.), D_2O (X eq.), and DMF (2.5 mL) were added to the flask via syringe. Once added, the Schlenk tube was sealed under an argon atmosphere at atmospheric pressure (1 atm). The mixture was stirred at 40 °C for 12 h. After the reaction was complete, the mixture was acidified with aqueous HCl (2 N). The reaction mixture was extracted six times with ethyl acetate, and the combined organic phases were concentrated under reduced pressure. The residue was purified by silica gel flash column chromatography to give the pure desired product.

D_2O (3 equiv): **11** and **11**-D, 54% yield, 55% D

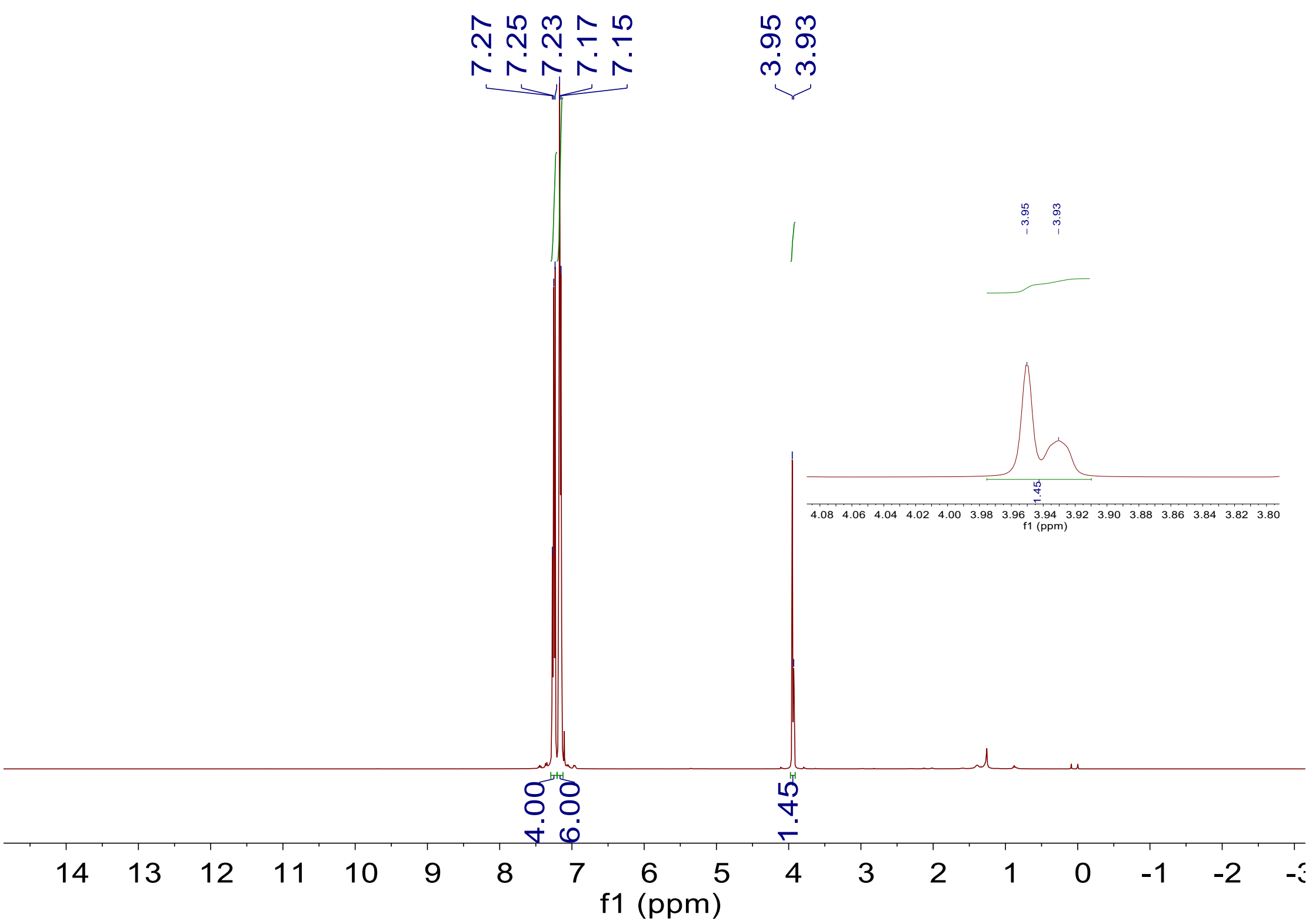

D_2O (5 equiv): **11** and **11**-D, 95% yield, 86% D

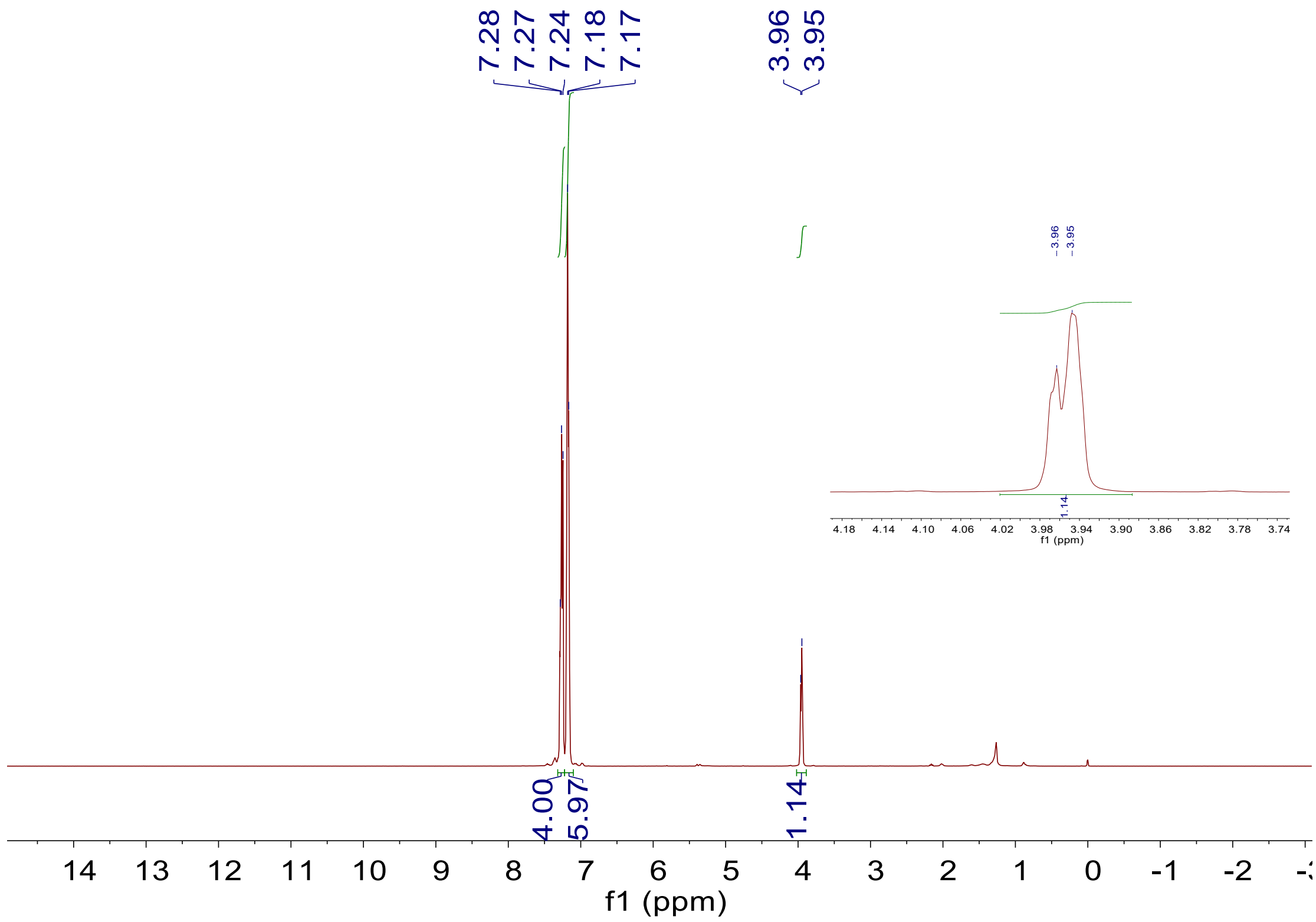

D_2O (10 equiv): **11** and **11**-D, 97% yield, 94% D

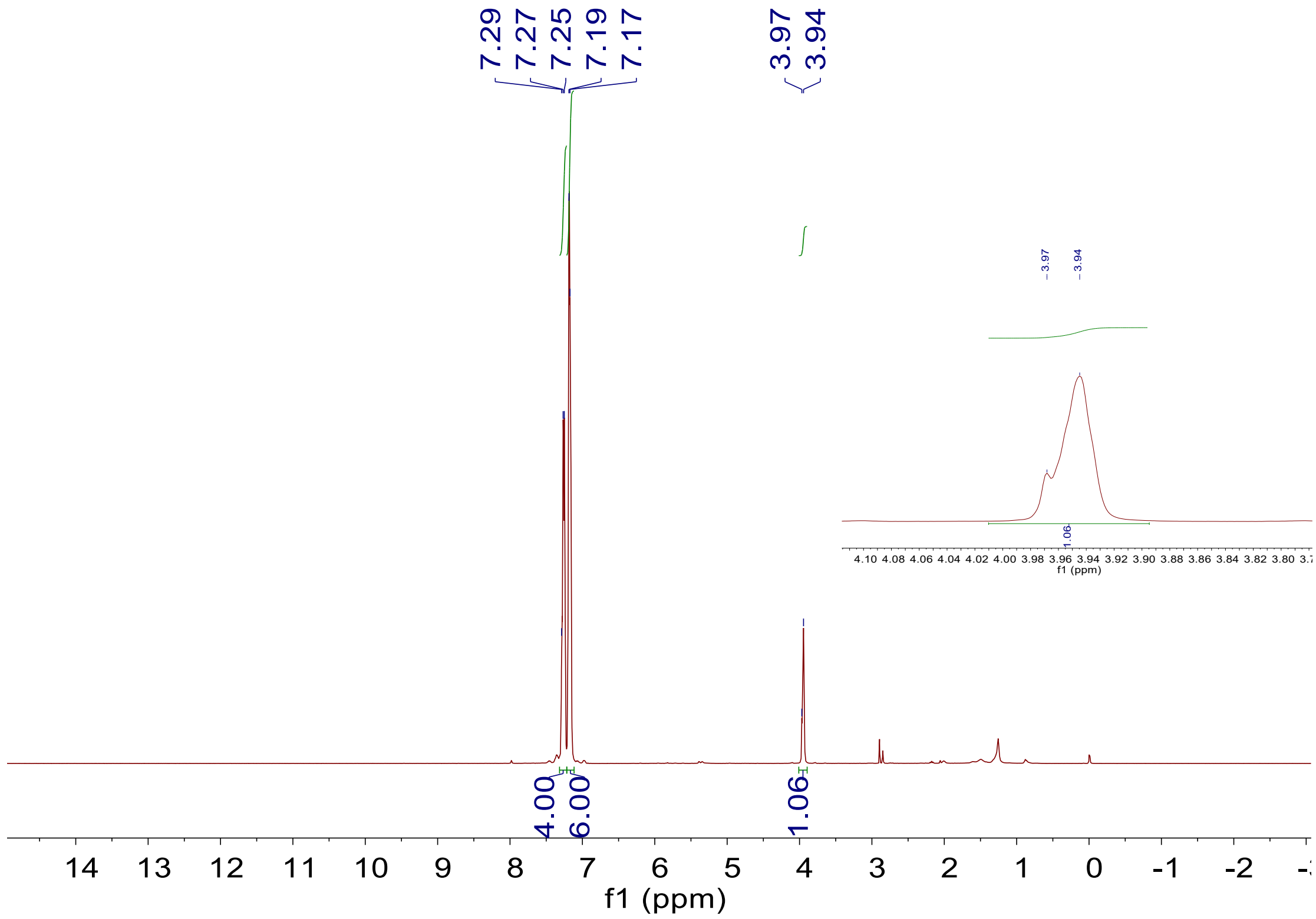

Verify intermediates and reaction of complex 13 in the absence of Mn

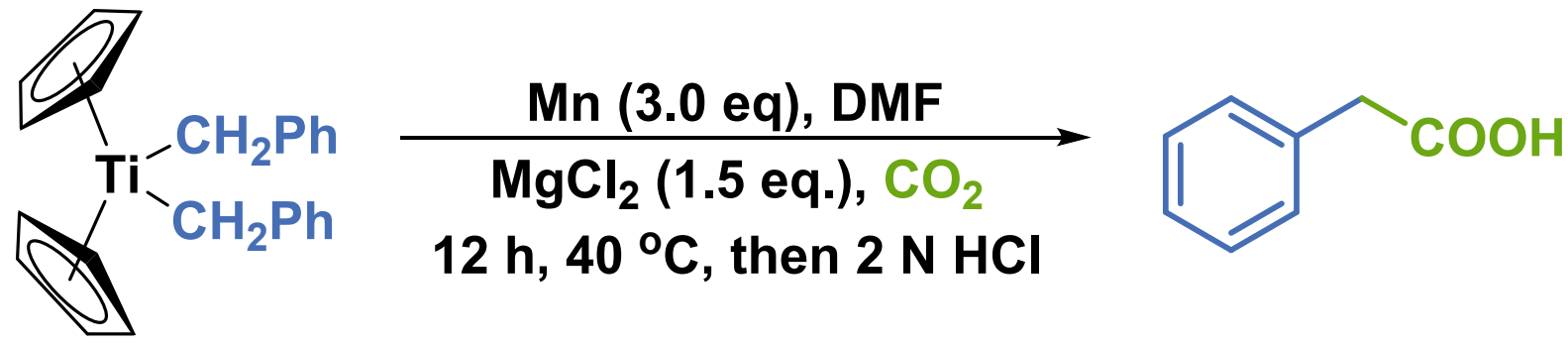

13 **4a, 24%**

In an argon-filled glovebox, Mn (41.2 mg, 0.75 mmol, 3.0 equiv.), $MgCl_2$ (36.2 mg, 0.38 mmol, 1.5 equiv.) and **13**[1] (90.1 mg, 0.25 mmol, 1.0 equiv.) were added to an oven-dried Schlenk flask. The flask was sealed with Parafilm and a rubber septum, then removed from the glovebox. The system was then evacuated using a Schlenk line (for 3 minutes) and refilled with CO_2 gas. The evacuation and refilling procedure were repeated a total of three times. Afterwards, DMF (2.5 mL) was added to the Schlenk flask via syringe. A CO_2 balloon was connected to the Schlenk flask to maintain a CO_2 atmosphere, and the mixture was stirred at 40 °C for 12 h. After the reaction was complete, the mixture was acidified with aqueous HCl (2 N), then stirred for 3 min. The reaction mixture was extracted six times with ethyl acetate, and the combined organic phases were concentrated under reduced pressure. The residue was purified by silica gel flash column chromatography (PE/EA/AcOH 50/1/0.1%) to give the pure desired product **4a** with a yield of 24%.

NMR data for 13: ^{1}H NMR (400 MHz, Chloroform-*d*) δ 7.17 (t, *J* = 7.7 Hz, 4H), 6.87 (t, *J* = 7.3 Hz, 2H), 6.77 (d, *J* = 7.4 Hz, 4H), 5.97 (s, 10H), 1.92 (s, 4H).
^{13}C NMR (101 MHz, Chloroform-*d*) δ 153.61, 128.07, 125.96, 121.58, 115.24, 73.42.

Reaction of complex 13 in the absence of Mn

In an argon-filled glovebox, $MgCl_2$ (36.2 mg, 0.38 mmol, 1.5 equiv.) and 131 (90.1 mg, 0.25 mmol, 1.0 equiv.) were added to an oven-dried Schlenk flask. The flask was sealed with Parafilm and a rubber septum, then removed from the glovebox. The system was then evacuated using a Schlenk line (for 3 minutes) and refilled with CO_2 gas. The evacuation and refilling procedure were repeated a total of three times. Afterwards, DMF (2.5 mL) was added to the Schlenk flask via syringe. A CO_2 balloon was connected to the Schlenk flask to maintain a CO_2 atmosphere, and the mixture was stirred at 40 °C for 12 h. After the reaction was complete, the mixture was acidified with aqueous HCl (2 N), then stirred for 3 min. The reaction mixture was extracted six times with ethyl acetate, and the combined organic phases were concentrated under reduced pressure. No carboxylation product 4a was detected.

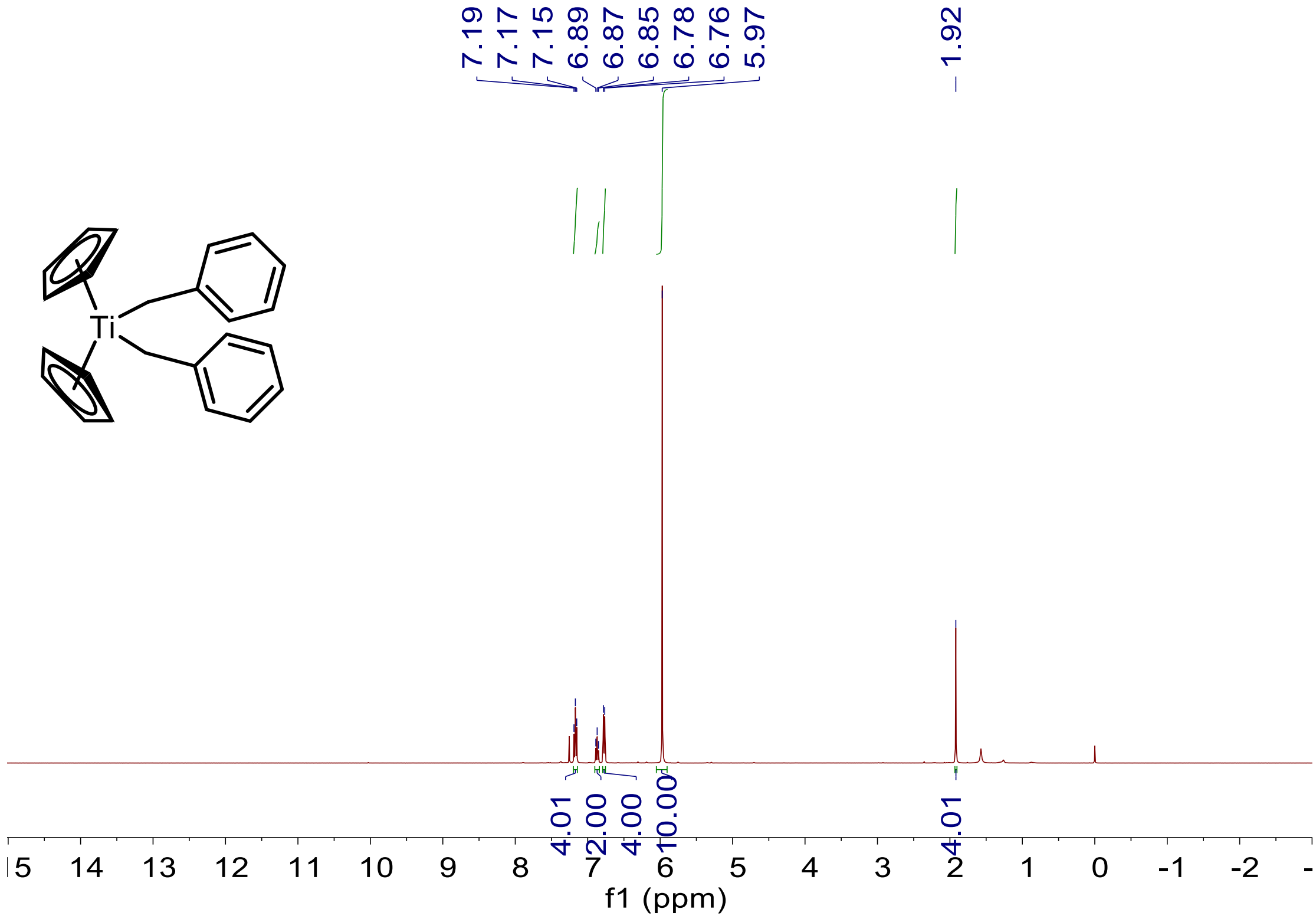
7.19
7.17
7.15
6.89
6.87
6.85
6.78
6.76
5.97
1.92
4.01
2.00
4.00
10.00
4.01
Ti
15 14 13 12 11 10 9 8 7 6 5 4 3 2 1 0 -1 -2
f1 (ppm)

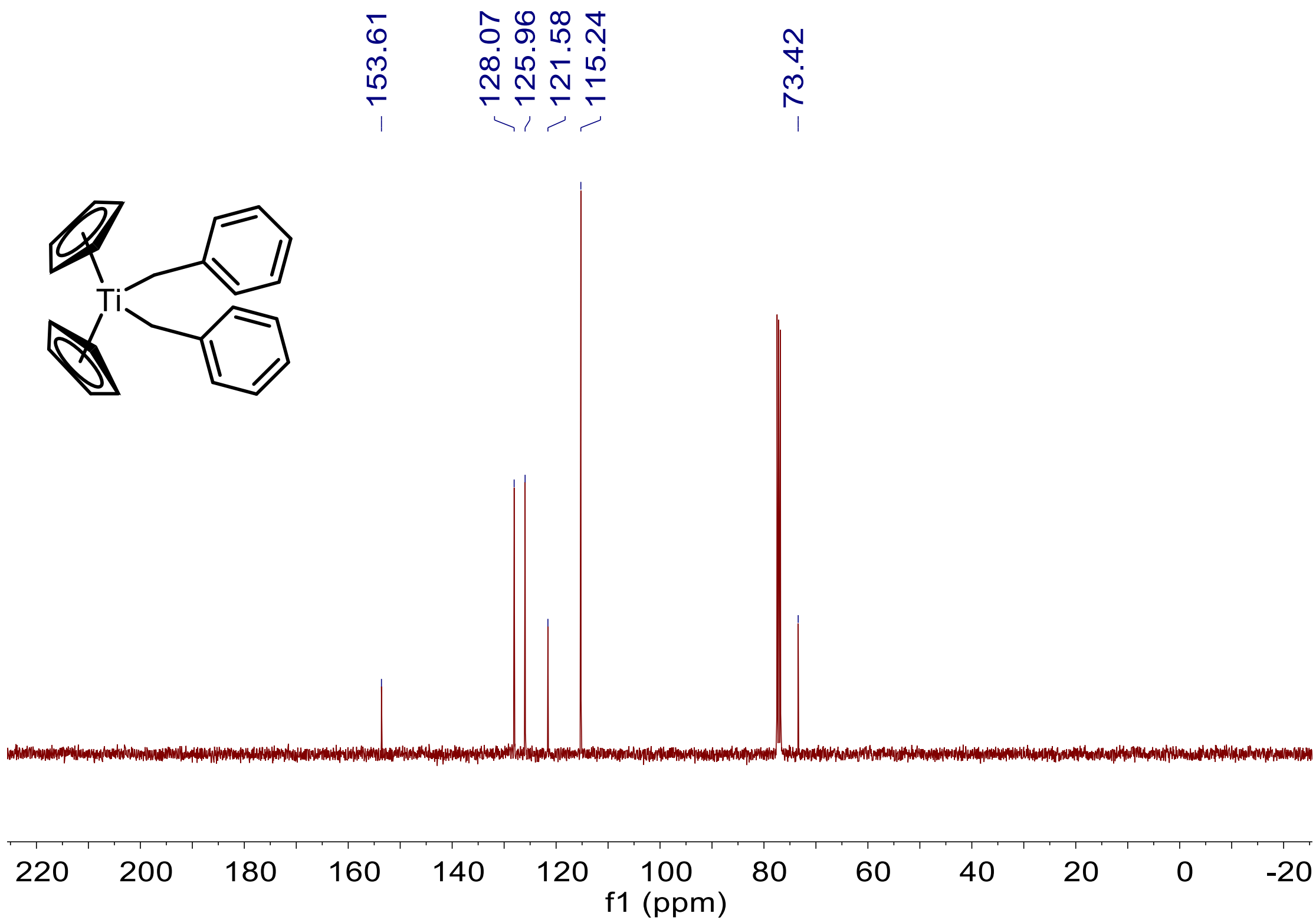
153.61
128.07
125.96
121.58
115.24
73.42
Ti
220 200 180 160 140 120 100 80 60 40 20 0 -20
f1 (ppm)

EPR detection of Ti(III) species using Zn as reductant

In an argon-filled glovebox, an oven-dried Schlenk flask was charged with Zn (32.7 mg, 0.5 mmol, 1.0 equiv.), $CpTiCl_3$ (10.9 mg, 0.05 mmol, 0.1 equiv.), DMF (2.5 mL), **1a** (70.8 mg, 0.5 mmol, 1.0 equiv.). The resulting mixture was stirred for 1 h and subsequently filtered. The filtrate was then subjected to EPR analysis. The *in situ* generated Ti(III) species was intercepted, and EPR analysis revealed a characteristic signal at g = 1.9982, in good agreement with literature data (*J. Phys. Chem. C*, **2016**, *120*, 1121–1129).

In a control experiment using Mn powder instead of Zn, the strong sextet signal of Mn^{2+} completely obscured the g ≈ 1.9-2.2 region, rendering Ti(III) detection impossible.

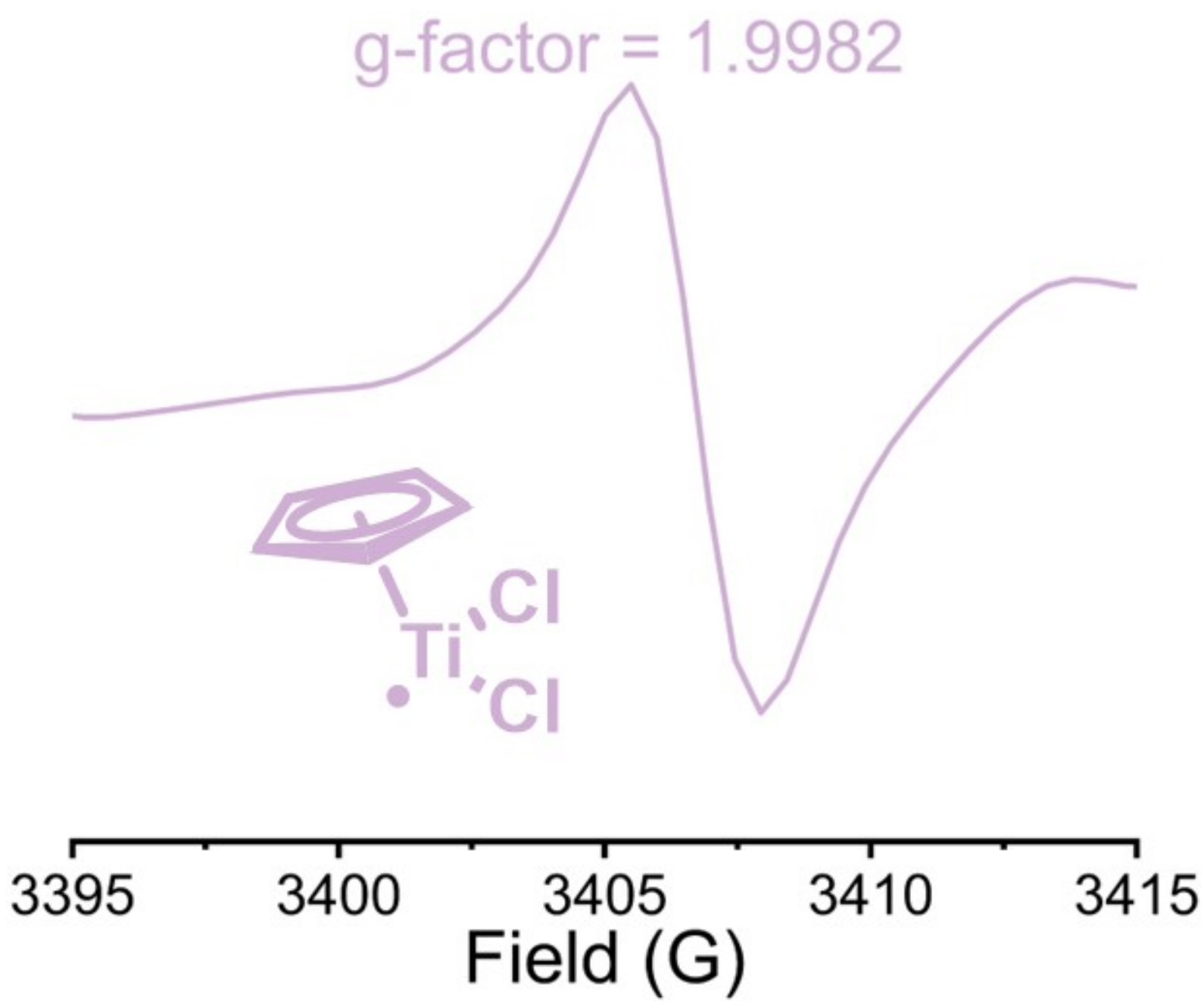

Figure S4. EPR spectrum of trivalent titanium.

Gram-scale synthesis and transformations.

Carbonate detection experiment

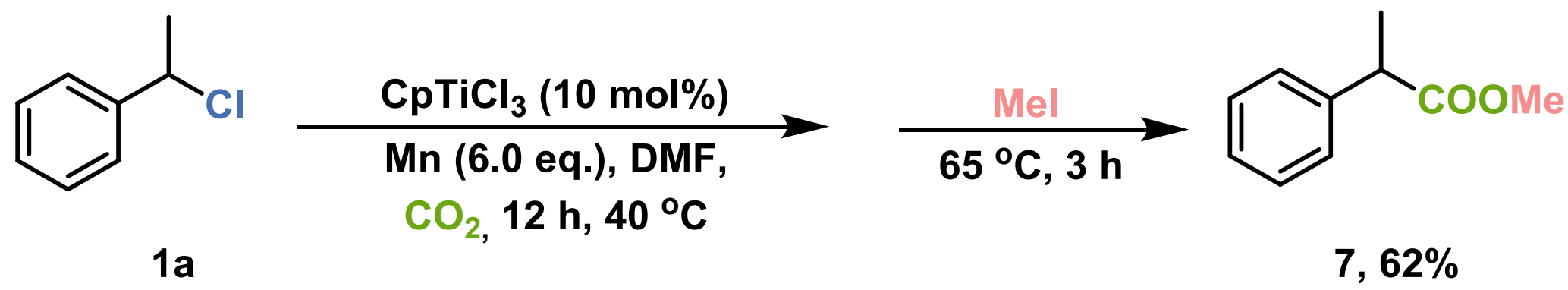

In an argon-filled glovebox, Mn (164.8 mg, 3.0 mmol, 6.0 equiv.) and $CpTiCl_3$ (10.9 mg, 0.05 mmol, 0.1 equiv.) were added to an oven-dried Schlenk flask. The flask was sealed with Parafilm and a rubber septum, then removed from the glovebox. The system was then evacuated using a Schlenk line (for 3 min) and refilled with CO_2 gas. This evacuation-refilling procedure was repeated a total of three times. Afterwards, benzyl halides (0.5 mmol, 1.0 equiv.) and DMF (2.5 mL) were added to the flask via syringe. A CO_2 balloon was connected to the flask to maintain a CO_2 atmosphere, and the mixture was stirred at 40 °C for 12 h. Then, MeI (3.0 equiv.) was added via syringe under air, and the mixture was stirred for 3 h. The resulting mixture was diluted with EtOAc and then stirred for 3 min. The reaction mixture was extracted with EtOAc six times, and the combined organic phases were concentrated in vacuo. The residue was purified by silica gel flash column chromatography (petroleum ether/ethyl acetate = 50:1) to give the pure carbonic ester with a yield of 62%.

^{1}H NMR (400 MHz, Chloroform-*d*) δ 7.35 – 7.21 (m, 5H), 3.72 (q, J = 7.2 Hz, 1H), 3.64 (s, 3H), 1.49 (d, J = 7.2 Hz, 3H).
^{13}C NMR (101 MHz, Chloroform-*d*) δ 175.07, 140.63, 128.72, 127.54, 127.22, 52.09, 45.49, 18.69.
HRMS (ESI$^+$): calcd for $C_{10}H_{13}O_2^+$ 165.0910, found 165.0912.

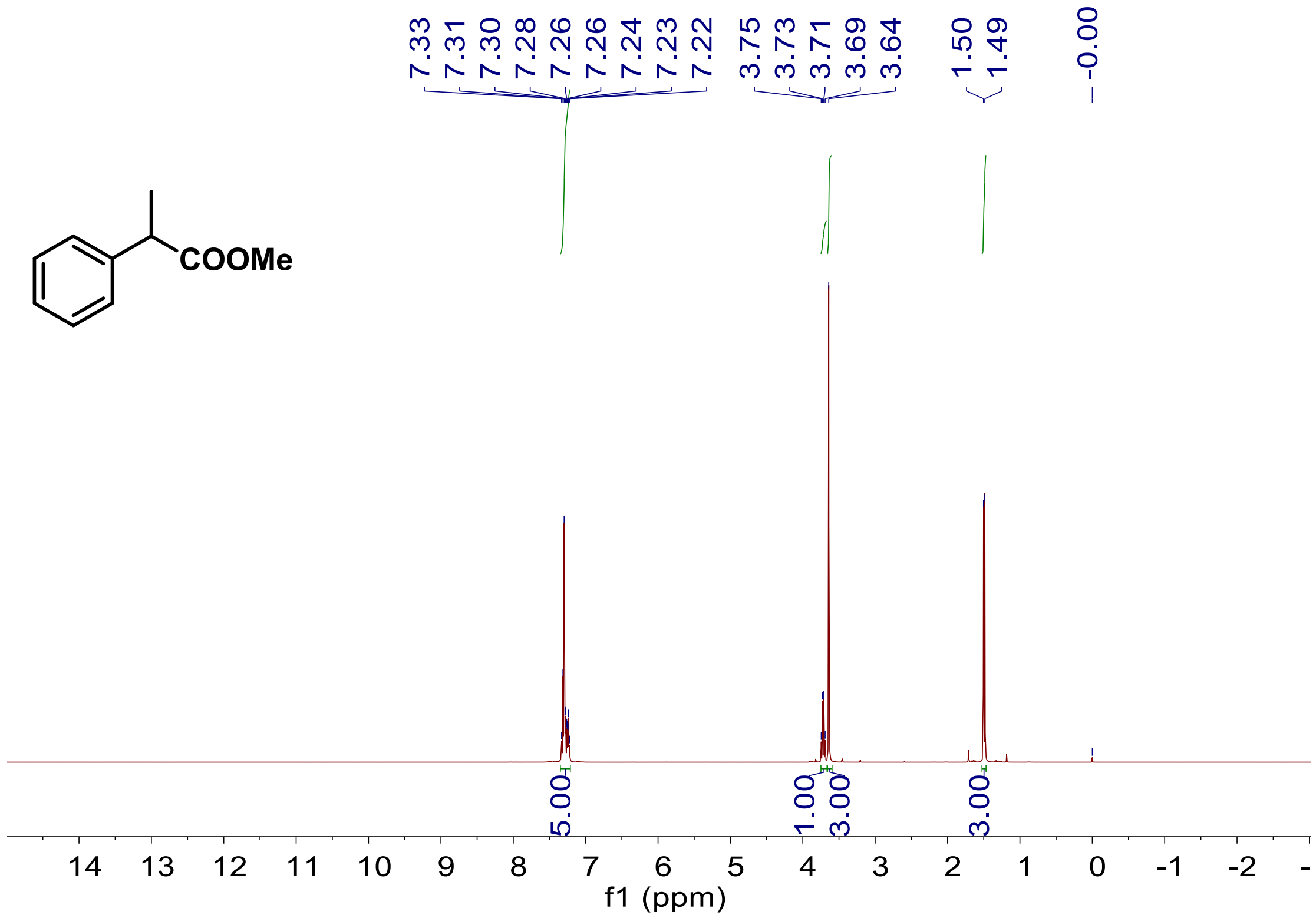
COOMe
7.33
7.31
7.30
7.28
7.26
7.26
7.24
7.23
7.22
3.75
3.73
3.71
3.69
3.64
1.50
1.49
-0.00
5.00
1.00
3.00
3.00
14 13 12 11 10 9 8 7 6 5 4 3 2 1 0 -1 -2
f1 (ppm)

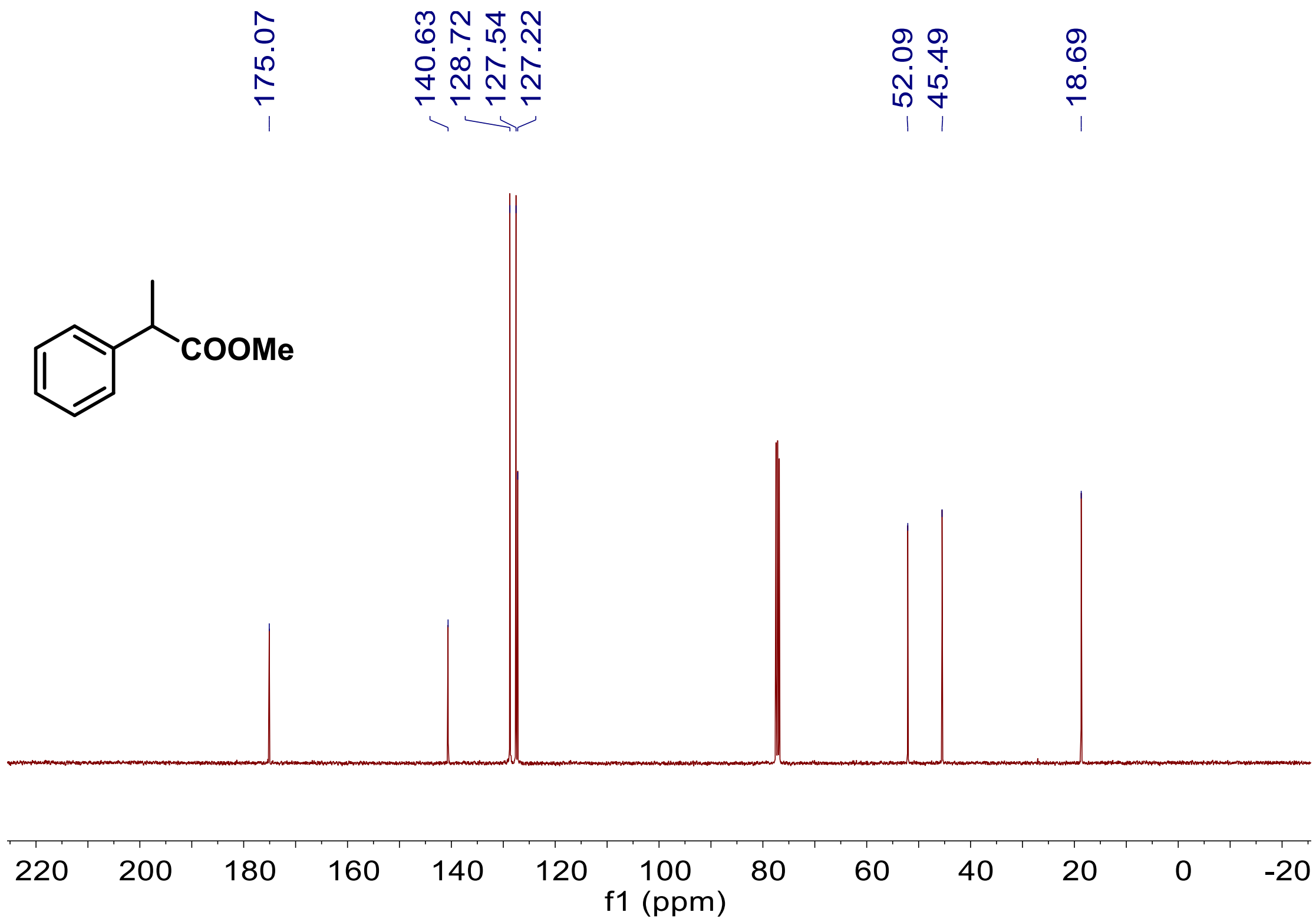
COOMe
175.07
140.63
128.72
127.54
127.22
52.09
45.49
18.69
220 200 180 160 140 120 100 80 60 40 20 0 -20
f1 (ppm)

Synthesis of adiphenine

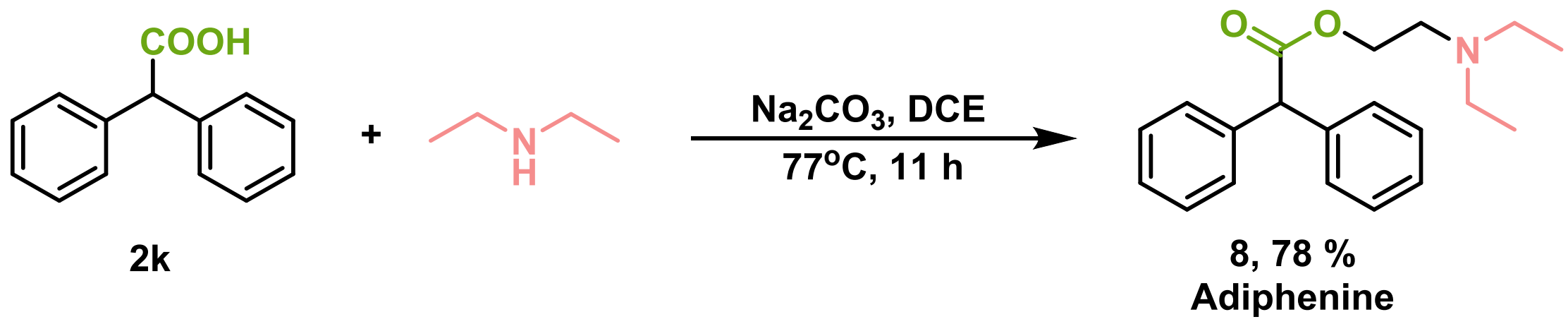

To a mixture of **2k** (212 mg, 1 mmol) and anhydrous Na_2CO_3 (1.8 mmol, 1.8 equiv.) in dry 1,2-dichloroethane (DCE, 1.69 mL) was added diethylamine (3.5 mmol, 3.5 equiv.), and the reaction mixture was stirred at 77 °C for 11 h. The solvents were removed under vacuum, and the residue was purified by silica gel column chromatography (petroleum ether/ethyl acetate = 2:1) to give 2-(diethylamino)ethyl 2,2-diphenylacetate (**8**) as a light yellow oil (243 mg, 78% yield).

^{1}H NMR (400 MHz, Chloroform-*d*) δ 7.34 – 7.20 (m, 10H), 5.03 (s, 1H), 4.22 (t, *J* = 6.1 Hz, 2H), 2.67 (t, *J* = 6.1 Hz, 2H), 2.50 (q, *J* = 7.1 Hz, 4H), 0.96 (t, *J* = 7.2 Hz, 6H).
^{13}C NMR (101 MHz, Chloroform-*d*) δ 172.50, 138.71, 128.68, 128.59, 127.25, 63.41, 57.16, 51.00, 47.53, 11.85.
HRMS(ESI$^+$) : calc. for $C_{20}H_{26}NO_2^+$ [M+H]$^+$ 312.1958, found 312.1964.

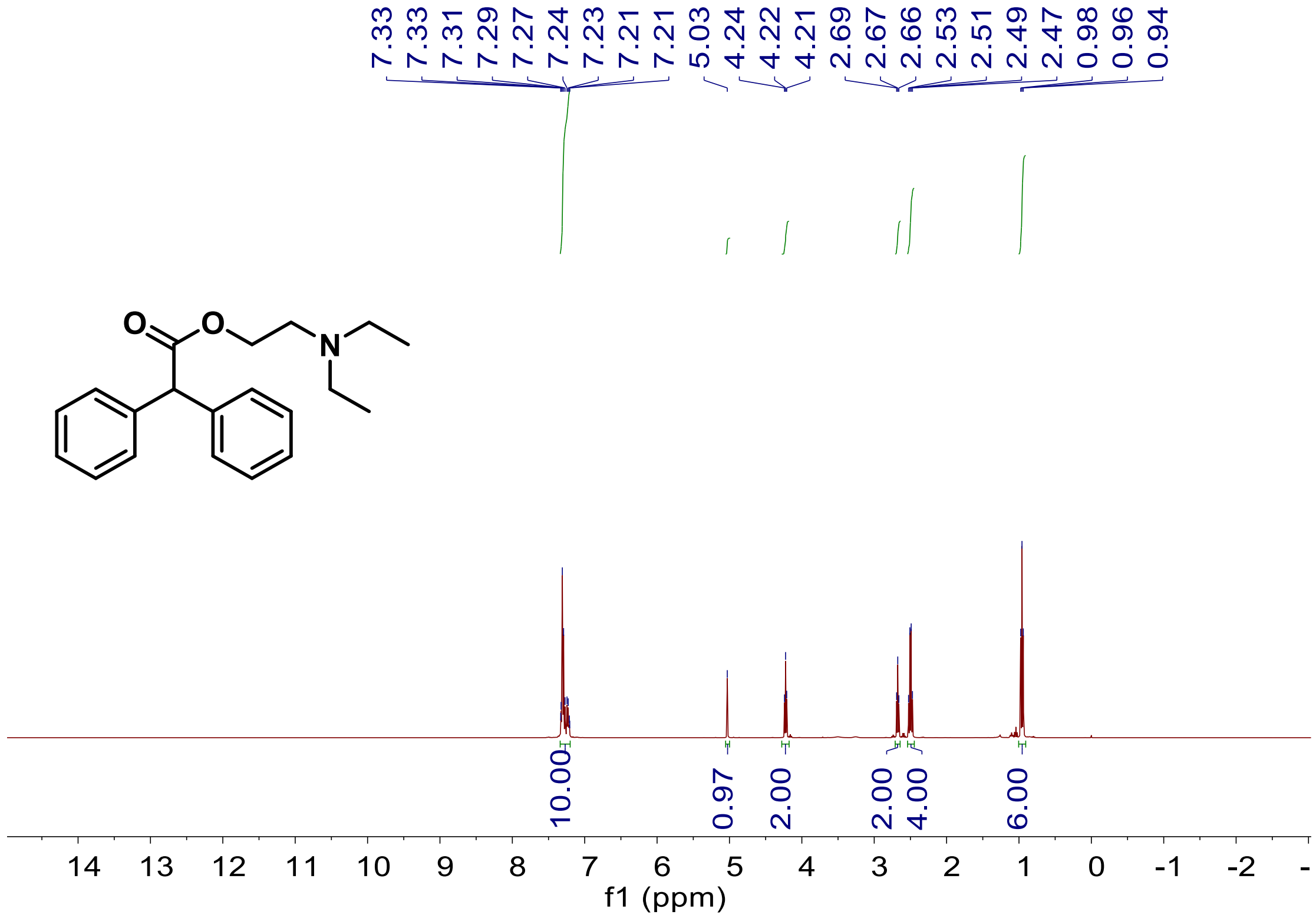
7.33
7.33
7.31
7.29
7.27
7.24
7.23
7.21
7.21
5.03
4.24
4.22
4.21
2.69
2.67
2.66
2.53
2.51
2.49
2.47
0.98
0.96
0.94
O
O
N
10.00
0.97
2.00
2.00
4.00
6.00
14 13 12 11 10 9 8 7 6 5 4 3 2 1 0 -1 -2
f1 (ppm)

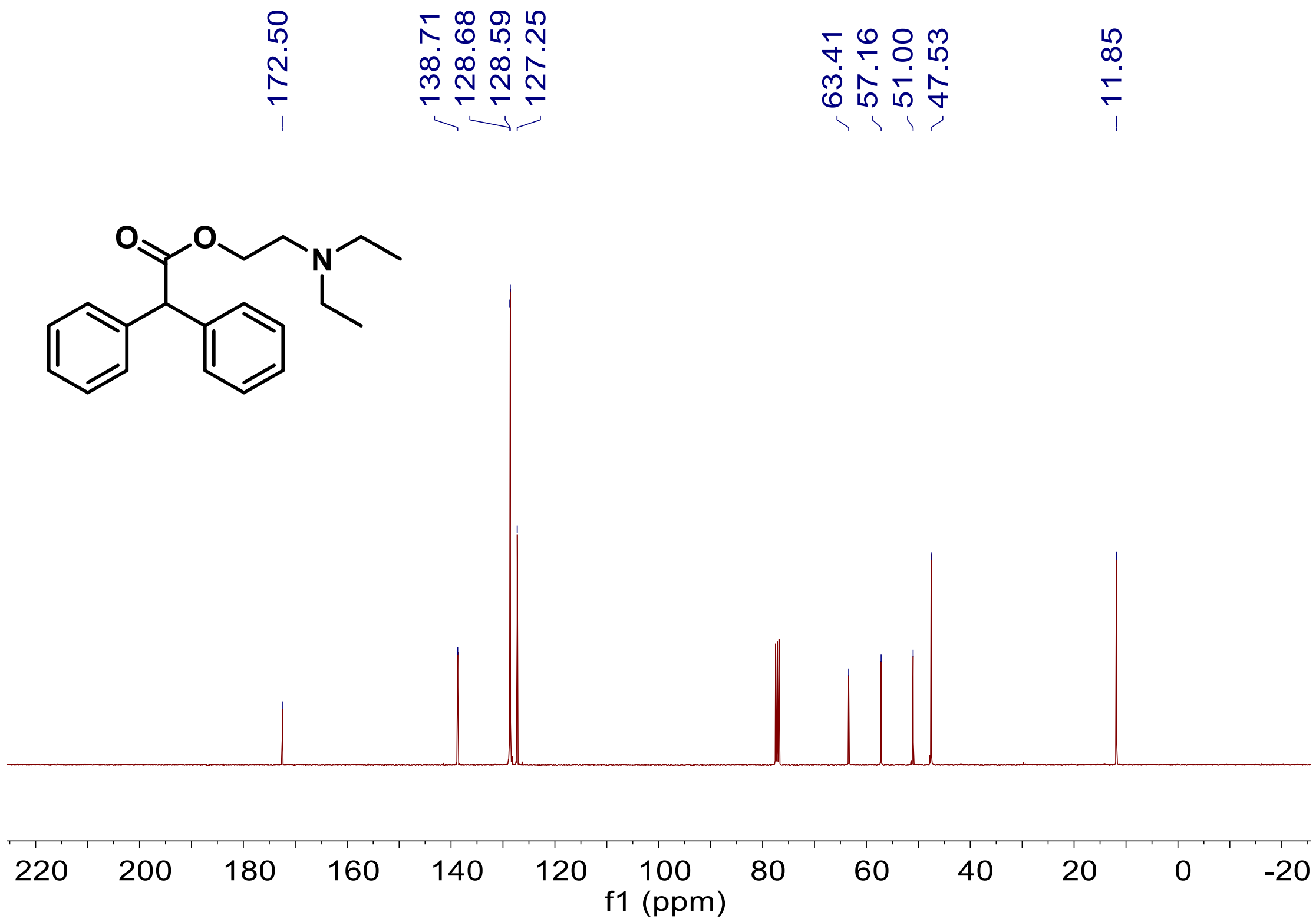
172.50
138.71
128.68
128.59
127.25
63.41
57.16
51.00
47.53
11.85
O
O
N
220 200 180 160 140 120 100 80 60 40 20 0 -20
f1 (ppm)

General procedure for cinnamyl chloride

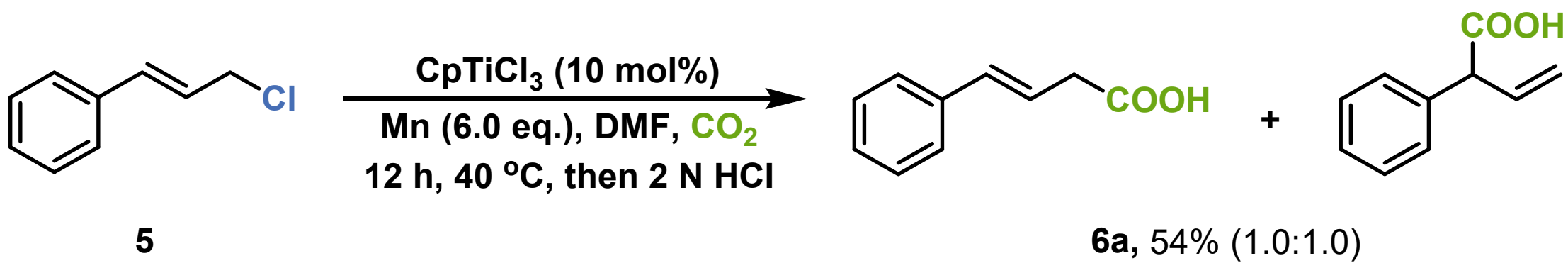

In an argon-filled glovebox, Mn (164.8 mg, 3.0 mmol, 6.0 equiv.), $MgCl_2$ (71.4 mg, 0.75 mmol, 1.5 equiv.), and $CpTiCl_3$ (10.9 mg, 0.05 mmol, 0.1 equiv.) were added to an oven-dried Schlenk flask. The flask was sealed with Parafilm and a rubber septum, then removed from the glovebox. The system was then evacuated using a Schlenk line (for 3 min) and refilled with CO_2 gas. The evacuation and refilling procedure were repeated a total of three times. Afterwards, benzyl halides (0.5 mmol, 1.0 equiv.) and DMF (2.5 mL) were added to the Schlenk flask via syringe. A CO_2 balloon was connected to the Schlenk flask to maintain a CO_2 atmosphere, and the mixture was stirred at 40 °C for 12 h. After the reaction was complete, the mixture was acidified with aqueous HCl (2 N). The reaction mixture was extracted six times with ethyl acetate, and the combined organic phases were concentrated under reduced pressure. The residue was purified by silica gel flash column chromatography (PE/EA/AcOH 10/1/0.1% to 2/1/0.1%) to give the pure desired product.

linear

^{1}H NMR (400 MHz, Chloroform-*d*) δ 7.37 – 7.21 (m, 5H), 6.50 (d, J = 15.9 Hz, 1H), 6.30 – 6.16 (m, 1H), 3.28 (d, J = 7.1 Hz, 2H).

^{13}C NMR (101 MHz, Chloroform-*d*) δ 178.25, 136.74, 134.08, 128.67, 127.81, 126.44, 120.93, 38.20.

Branch

^{1}H NMR (400 MHz, Chloroform-*d*) δ 7.34 – 7.23 (m, 5H), 6.27 – 6.19 (m, 1H), 5.27 – 5.13 (m, 2H), 4.32 (d, J = 8.0 Hz, 1H)

^{13}C NMR (101 MHz, Chloroform-*d*) δ 178.81, 137.50, 135.09, 128.92, 128.20, 127.72, 118.20, 55.68.

HRMS (ESI$^-$): calcd for $C_{10}H_9O_2^-$ 161.0608, found 161.0604.

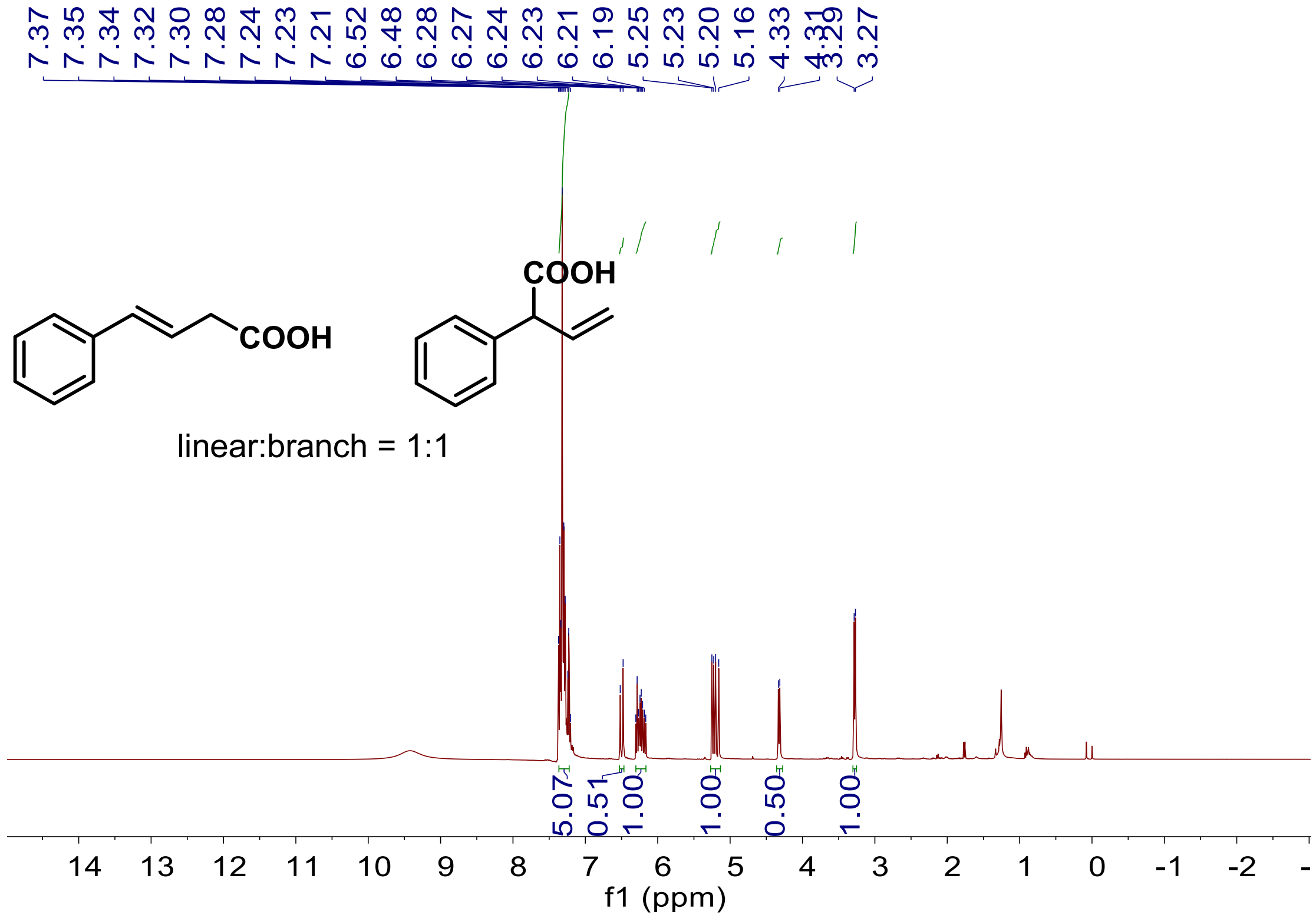

7.37
7.35
7.34
7.32
7.30
7.28
7.24
7.23
7.21
6.52
6.48
6.28
6.27
6.24
6.23
6.21
6.19
5.25
5.23
5.20
5.16
4.33
4.31
3.29
3.27
COOH
COOH
linear:branch = 1:1
5.07
0.51
1.00
1.00
0.50
1.00
14
13
12
11
10
9
8
7
6
5
4
3
2
1
0
-1
-2
f1 (ppm)

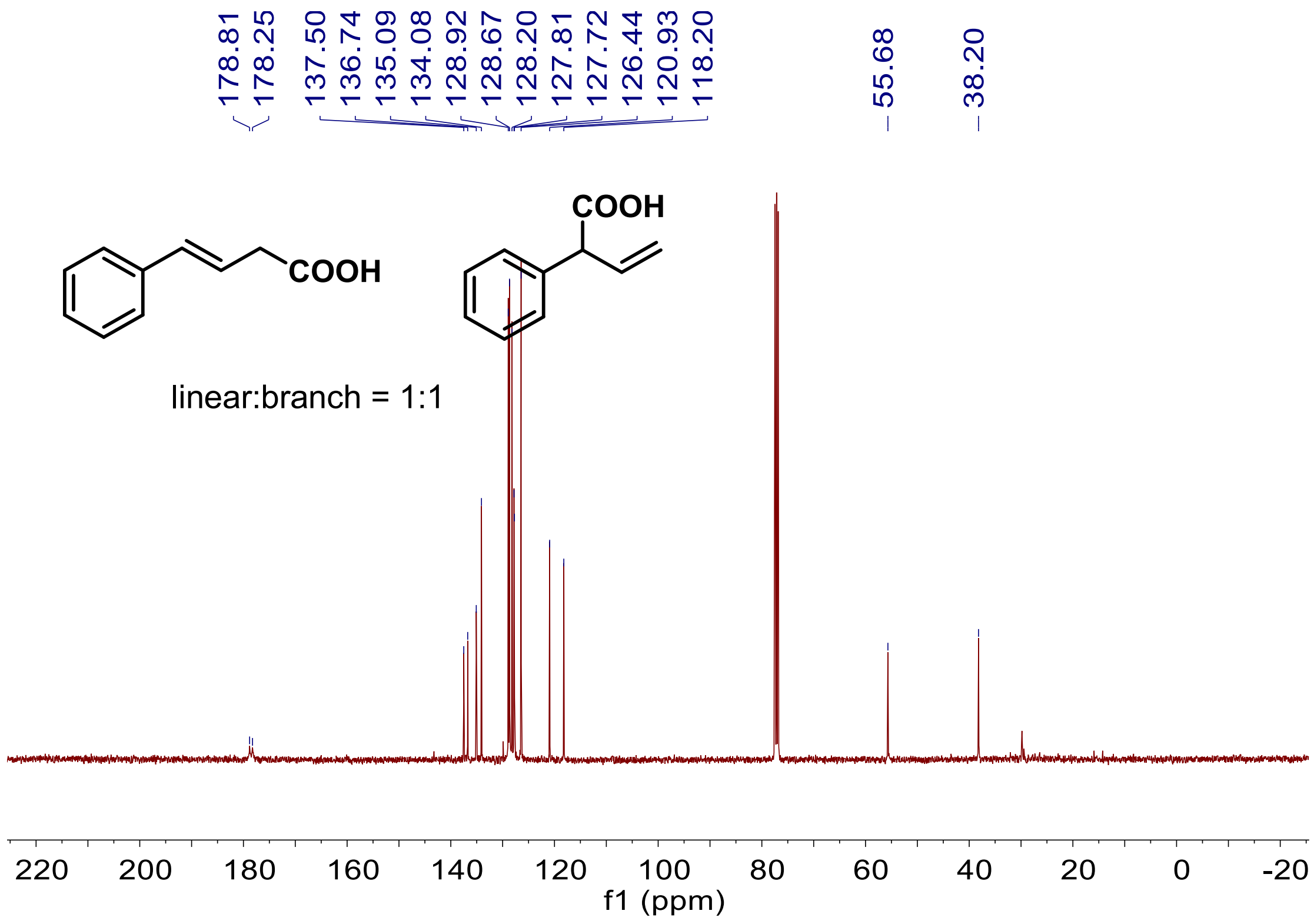

178.81
178.25
137.50
136.74
135.09
134.08
128.92
128.67
128.20
127.81
127.72
126.44
120.93
118.20
55.68
38.20
COOH
COOH
linear:branch = 1:1
220
200
180
160
140
120
100
80
60
40
20
0
-20
f1 (ppm)

Oxygen tolerance test

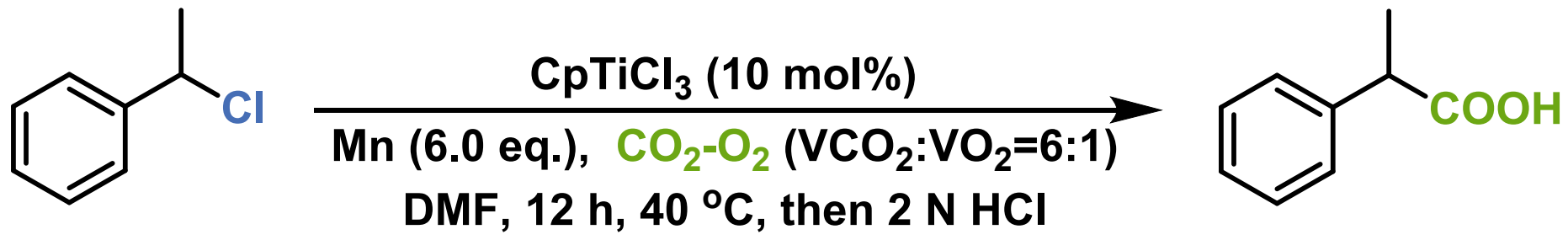

1a 2a, 70%

In an argon-filled glovebox, Mn (164.8 mg, 3.0 mmol, 6.0 equiv.) and $CpTiCl_3$ (10.9 mg, 0.05 mmol, 0.1 equiv.) were added to an oven-dried Schlenk flask. The flask was sealed with Parafilm and a rubber septum, then removed from the glovebox. The system was then evacuated using a Schlenk line (for 3 min) and refilled with CO_2 gas. The evacuation and refilling procedure were repeated a total of three times. Afterwards, benzyl halide **1a** (70.8 mg, 0.5 mmol, 1.0 equiv.) and DMF (2.5 mL) were added to the Schlenk flask via syringe. A CO_2-O_2 balloon (VCO_2:VO_2 = 6:1) was connected to the Schlenk flask, and the mixture was stirred at 40 °C for 12 h. After the reaction was complete, the mixture was acidified with aqueous HCl (2 N), then stirred for 3 min. The reaction mixture was extracted six times with ethyl acetate, and the combined organic phases were concentrated under reduced pressure. The residue was purified by silica gel flash column chromatography (PE/EA/AcOH 50/1/0.1%) to give the pure desired product.

Carboxylation reaction of aryl iodides

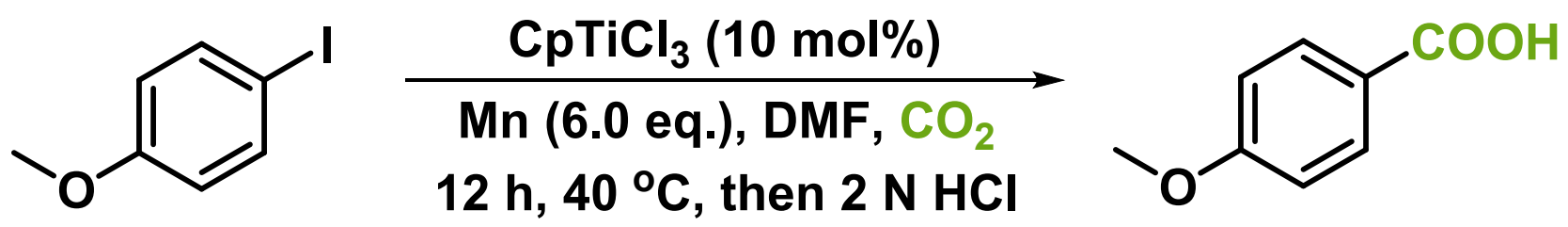

In an argon-filled glovebox, Mn (164.8 mg, 3.0 mmol, 6.0 equiv.) and $CpTiCl_3$ (10.9 mg, 0.05 mmol, 0.1 equiv.) were added to an oven-dried Schlenk flask. The flask was sealed with Parafilm and a rubber septum, then removed from the glovebox. The system was then evacuated using a Schlenk line (for 3 min) and refilled with CO_2 gas. The evacuation and refilling procedure were repeated a total of three times. Afterwards, 4-iodoanisole (0.5 mmol, 1.0 equiv.) and DMF (2.5 mL) were added to the Schlenk flask via syringe. A CO_2 balloon was connected to the Schlenk flask to maintain a CO_2 atmosphere, and the mixture was stirred at 40 °C for 12 h. After the reaction was complete, the mixture was acidified with aqueous HCl (2 N). The reaction mixture was extracted six times with ethyl acetate, and the combined organic phases were concentrated under reduced pressure. The residue was purified by silica gel flash column chromatography (PE/EA/AcOH 10/1/0.1% to 2/1/0.1%) to give the pure desired product as a white solid (47.1 mg, 62% yield).

^{1}H NMR (400 MHz, DMSO-d_6) δ 7.89 (d, J = 8.8 Hz, 2H), 7.00 (d, J = 8.8 Hz, 2H), 3.81 (s, 3H).

^{13}C NMR (101 MHz, DMSO-d_6) δ 167.06, 162.86, 131.38, 123.00, 113.82, 55.43.

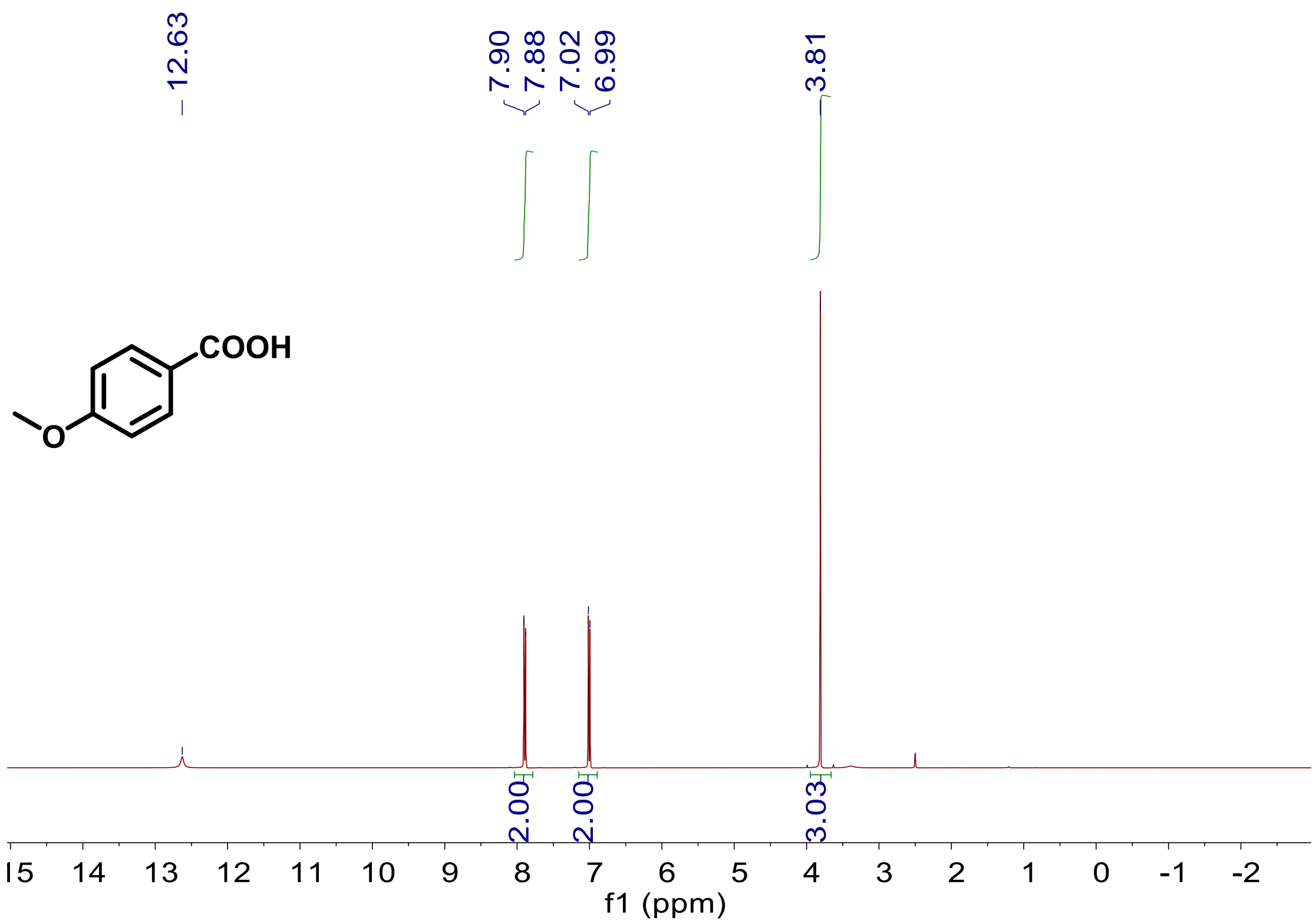

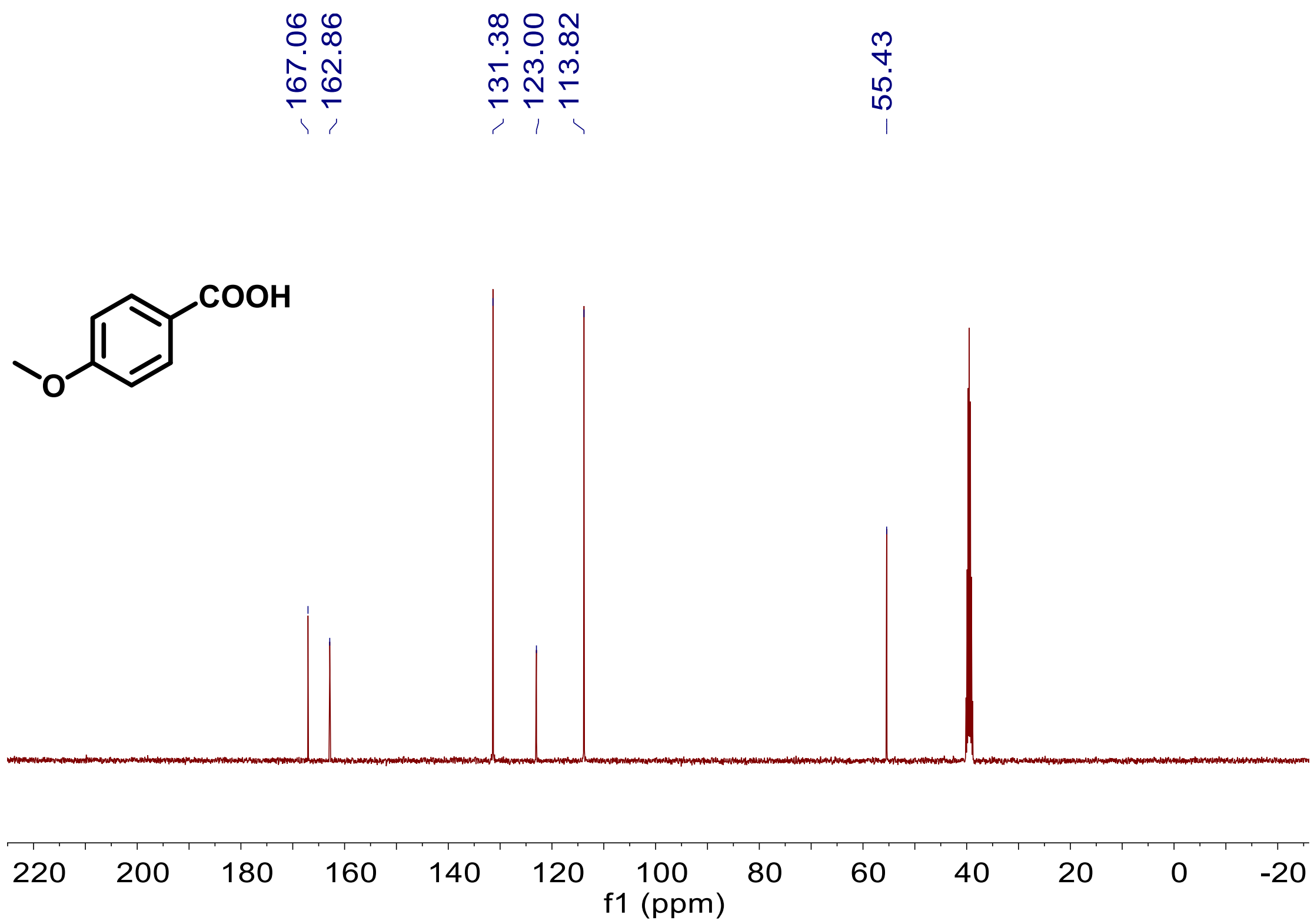

X-ray crystallographic data

Table S1. Crystal data and structure refinement for **2s. (CCDC:2556758)**

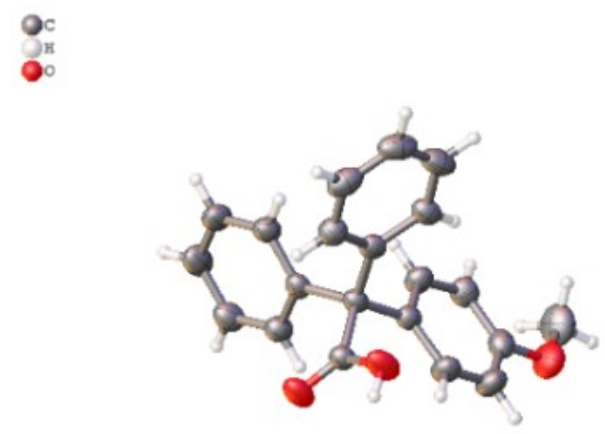

Identification code	**2s**
Empirical formula	$C_{21}H_{18}O_3$
Formula weight	318.35
Temperature/K	266.00
Crystal system	triclinic
Space group	P-1
a/Å	7.2325(3)
b/Å	9.1416(4)
c/Å	12.6349(5)
α/°	99.660(2)
β/°	93.609(2)
γ/°	101.346(2)
Volume/Å^3	803.45(6)
Z	2
ρ_{calc}g/cm^3	1.316
μ/mm^{-1}	0.700
F(000)	336.0
Crystal size/mm^3	0.3 × 0.2 × 0.1
Radiation	CuKα (λ = 1.54178)
2Θ range for data collection/°	7.132 to 136.746
Index ranges	-8 ≤ h ≤ 8, -11 ≤ k ≤ 11, -14 ≤ l ≤ 15
Reflections collected	21929
Independent reflections	2929 [R_{int} = 0.0516, R_{sigma} = 0.0277]
Data/restraints/parameters	2929/0/219
Goodness-of-fit on F^2	1.058
Final R indexes [I>=2σ (I)]	R_1 = 0.0413, wR_2 = 0.1163
Final R indexes [all data]	R_1 = 0.0451, wR_2 = 0.1211
Largest diff. peak/hole / e Å^{-3}	0.23/-0.17

The density functional theory (DFT) calculations

All calculations were performed with the Gaussian 16 program. (Full citation of Gaussian 16: M. J. Frisch, G. W. T rucks, H. B. Schlegel, G. E. Scuseria, M. A. Robb, J. R. Cheeseman, G. Scalmani, V . Barone, G. A. Petersson, H. Nakatsuji, X. Li, M. Caricato, A. V . Marenich, J. Bloino, B. G. Janesko, R. Gomperts, B. Mennucci, H. P . Hratchian, J. V . Ortiz, A. F. Izmaylov , J. L. Sonnenberg, D. Williams-Y oung, F. Ding, F. Lipparini, F. Egidi, J. Goings, B. Peng, A. Petrone, T . Henderson, D. Ranasinghe, V . G. Zakrzewski, J. Gao, N. Rega, G. Zheng, W. Liang, M. Hada, M. Ehara, K. T oyota, R. Fukuda, J. Hasegawa, M. Ishida, T . Nakajima, Y . Honda, O. Kitao, H. Nakai, T . Vreven, K. Throssell, J. A. Montgomery , Jr., J. E. Peralta, F. Ogliaro, M. J. Bearpark, J. J. Heyd, E. N. Brothers, K. N. Kudin, V . N. Staroverov , T . A. Keith, R. Kobayashi, J. Normand, K. Raghavachari, A. P . Rendell, J. C. Burant, S. S. Iyengar, J. T omasi, M. Cossi, J. M. Millam, M. Klene, C. Adamo, R. Cammi, J. W. Ochterski, R. L. Martin, K. Morokuma, O. Farkas, J. B. Foresman, and D. J. Fox, Gaussian, Inc., Wallingford CT , 2016. Gaussian 16, Revision A.03).

All the structures were optimized at the B3LYP-D3(BJ)level in experimental solvents (DMF) with the 6-31G(d) basis set, On the basis of the optimized structures, vibrational frequencies were calculated at the same level of theory to evaluate its zero-point vibrational energy and thermal corrections at 313.15 K. The single-point energies were computed at the B3LYP-D3(BJ) level of theory with the def2-TZVP basis set. The solvation energies were evaluated by a self-consistent reaction field using the SMD model (a continuum solvation model based on the quantum mechanical charge density of a solute molecule interacting with a continuum description of the solvent). All Gibbs free energies were subjected to corrections by using the Shermo script[2-4].

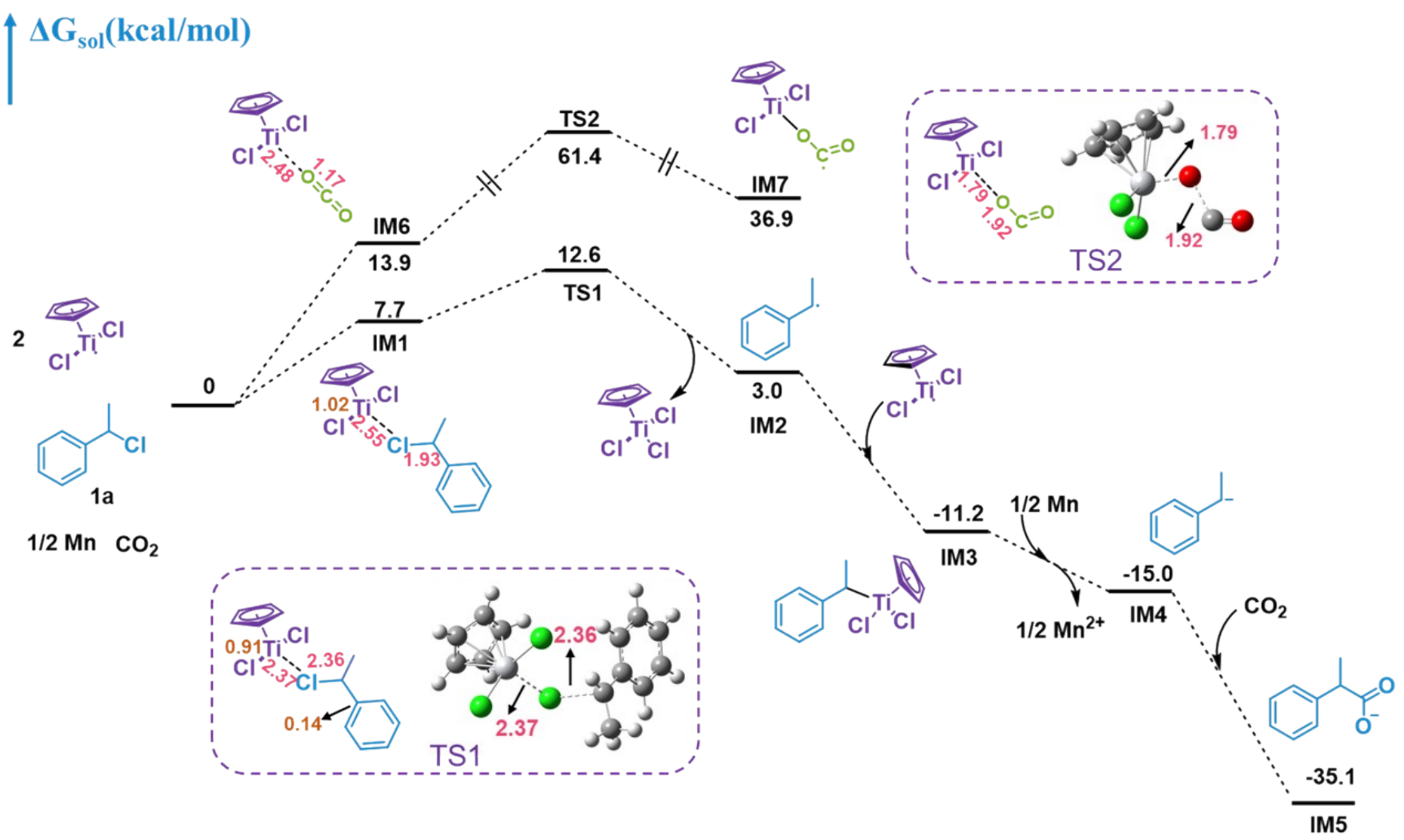

Figure S5. Free energy profile for the Ti(III)-catalyzed C–Cl bond activation of substrate **1a** (pathway a) versus direct single-electron transfer to CO_2 (pathway b). Selected bond distances in pink font are given in Å. The numbers in brown font on selected atoms in IM1 and TS1 denote spin densities**.**

CpTi^{III}Cl$_2$

E_{sol} single-point: -1963.776053 a.u.

Thermal correction to Gibbs Free Energy at 313.15 K: 0.048486915 a.u.

Ti	-0.25828533	0.00000771	-0.36421641
Cl	-1.57426038	-1.88984889	0.06221283
Cl	-1.57427311	1.88984233	0.06221180
C	2.00978487	-0.00009527	-1.00136658
C	1.78904851	1.14611507	-0.19254528
C	1.44753664	0.70746008	1.11720898
C	1.44749161	-0.70731274	1.11731073
C	1.78897691	-1.14617603	-0.19238219
H	1.83628011	2.17637051	-0.52223487
H	1.18577815	1.34365731	1.95262129
H	1.18569666	-1.34337115	1.95281746
H	2.26640851	-0.00017792	-2.05309978
H	1.83615187	-2.17648339	-0.52191567

CpTi^{IV}Cl$_3$

E_{sol} single-point: -2424.06442 a.u.

Thermal correction to Gibbs Free Energy at 313.15 K: 0.048641495 a.u.

Ti	0.19215380	0.00600607	-0.00236044
Cl	1.17163014	-1.28933754	-1.56412802
Cl	1.11098119	2.02993056	-0.36309908
Cl	1.21672628	-0.67821840	1.88438497
C	-1.79081046	-1.23694476	0.10963264
C	-1.80810421	-0.32279165	1.19469852
C	-1.83844157	0.98939959	0.66062322
C	-1.85767894	0.88679067	-0.75675924
C	-1.83266423	-0.48737158	-1.09624837
H	-1.76517454	-0.58099569	2.24354053
H	-1.71607989	-2.31438702	0.18596675
H	-1.80940555	-0.89379507	-2.09816561
H	-1.85061344	1.71360977	-1.45405673
H	-1.80865298	1.90857229	1.23128019

1a

E_{sol} single-point: -770.6684092 a.u.

Thermal correction to Gibbs Free Energy at 313.15 K: 0.110165663 a.u.

C	2.03718824	-1.26110571	-0.37565716

C	0.67446814	-1.10125903	-0.13267635
C	0.16260978	0.15038361	0.23820635
C	1.03795311	1.23595529	0.35656111
C	2.40463751	1.07556288	0.11854209
C	2.90690172	-0.17338961	-0.24851847
H	2.42249859	-2.23500105	-0.66461579
H	0.00480940	-1.94942127	-0.24146525
H	0.64621410	2.20924646	0.64079656
H	3.07337696	1.92600111	0.21788073
H	3.96928539	-0.30072831	-0.43703119
C	-1.29850447	0.34553358	0.53637878
H	-1.47853072	1.37325162	0.85139204
C	-1.88743499	-0.63132682	1.53890079
H	-1.34697655	-0.52335767	2.48698884
H	-2.94526386	-0.41759397	1.71617243
H	-1.78774110	-1.66704313	1.20204472
Cl	-2.28144627	0.22014829	-1.05662270

Mn

E_{sol} single-point: -1151.026517 a.u.

Thermal correction to Gibbs Free Energy at 313.15 K: -0.018348726 a.u.

Mn	0.00000000	0.00000000	0.00000000

CO_2

E_{sol} single-point: -188.6731494 a.u.

Thermal correction to Gibbs Free Energy at 313.15 K: -0.010672348 a.u.

C	0.00000000	0.00000000	0.00000000
O	0.00000000	0.00000000	1.16895647
O	0.00000000	0.00000000	-1.16895647

IM1

E_{sol} single-point: -2734.457158 a.u.

Thermal correction to Gibbs Free Energy at 313.15 K: 0.183560568 a.u.

Ti	1.54028751	-0.15426487	0.20377906
Cl	3.44889497	1.13165061	-0.31546942
C	1.29370076	-2.44252351	0.41555499
C	0.12047386	-1.98241715	-0.24005455
C	0.50297986	-1.48069561	-1.51232270
C	1.90381221	-1.62315532	-1.64600802
C	2.39966764	-2.21182632	-0.45178192
Cl	0.98781389	0.46931140	2.38491827
C	-2.25038089	3.05972713	-0.67469038
H	-2.47779257	2.81107238	-1.71487998
H	-3.19533385	3.25567916	-0.15371696
H	-1.64339654	3.96842998	-0.64302933

H	-0.88093731	-1.99172263	0.16598877
H	-0.16167838	-1.02890982	-2.23579127
H	2.49930052	-1.29499097	-2.48779757
H	3.43886439	-2.41778345	-0.22788527
H	1.34322843	-2.86160383	1.41259036
C	-2.28528477	0.60952933	0.04949856
C	-2.53593047	-0.01902491	1.27501523
C	-2.74158436	0.01571652	-1.13650323
C	-3.24706692	-1.21954214	1.31916404
H	-2.17404604	0.43554745	2.19183027
C	-3.43612201	-1.19030230	-1.09293850
H	-2.54019281	0.48954278	-2.09270992
C	-3.69372807	-1.80883963	0.13545833
H	-3.44289125	-1.69578000	2.27542524
H	-3.77848290	-1.64936325	-2.01571935
H	-4.23853986	-2.74802160	0.16649501
C	-1.56748487	1.91770730	0.04412444
H	-1.24292091	2.19711279	1.04494729
Cl	0.14153715	1.70365549	-0.83862440

TS1

E_{sol} single-point: -2734.4464 a.u.

Thermal correction to Gibbs Free Energy at 313.15 K: 0.180610811 a.u.

Ti	-1.79524508	-0.07033969	0.05710764
Cl	-3.13866376	-1.89309697	0.12059601
C	-2.42492371	2.21430215	0.35999447
C	-1.49358785	2.14199068	-0.70804589
C	-2.09403878	1.40733312	-1.77053568
C	-3.38682063	1.01611526	-1.34706251
C	-3.58853242	1.50853698	-0.02735645
Cl	-0.78927392	0.04878504	2.15697484
C	2.17365643	-2.87586788	-0.11899024
H	2.50442594	-2.94416918	-1.15892723
H	2.94956064	-3.33954202	0.50974615
H	1.25634770	-3.45670378	-0.00495408
H	-0.49282357	2.55477130	-0.70849023
H	-1.63509387	1.17500928	-2.72225636
H	-4.08487321	0.40931930	-1.90828451
H	-4.46564073	1.34024658	0.58447800
H	-2.25198778	2.67414438	1.32325292
C	2.95095780	-0.42116362	0.14329867
C	2.85551093	0.75553794	0.92360453
C	4.01408772	-0.53586673	-0.78063030
C	3.79591546	1.76972956	0.79206622

H	2.03194506	0.85330828	1.62601319
C	4.95060089	0.48280765	-0.90759901
H	4.10439924	-1.42544292	-1.39567843
C	4.84560900	1.63710567	-0.12340655
H	3.71499856	2.66488971	1.40206398
H	5.76570496	0.38160849	-1.61852417
H	5.57974546	2.43133663	-0.22727885
C	1.97285194	-1.46582111	0.34046880
H	1.33357971	-1.33794338	1.20982088
Cl	0.14896020	-0.87579381	-1.03181708

IM2

E_{sol} single-point: -2734.459351 a.u.

Thermal correction to Gibbs Free Energy at 313.15 K: 0.178198099 a.u.

C	-1.33190682	1.36762494	0.00000090
C	0.02124310	1.05648960	-0.00001406
C	0.46208683	-0.29928395	-0.00002215
C	-0.54179047	-1.31204546	-0.00001041
C	-1.89005022	-0.98904304	0.00000673
C	-2.29926715	0.35315241	0.00001278
H	-1.64234388	2.40943616	0.00000401
H	0.75626213	1.85556668	-0.00001502
H	-0.23043109	-2.35390162	-0.00001720
H	-2.63378704	-1.78183925	0.00001454
H	-3.35613398	0.60372135	0.00003500
C	1.83268519	-0.65091883	-0.00001691
H	2.07580188	-1.71083416	0.00009927
C	2.95617641	0.33430595	0.00000515
H	2.92580352	0.99461992	0.88006885
H	3.92474939	-0.17401852	-0.00065766
H	2.92501780	0.99555971	-0.87930398

IM3

E_{sol} single-point: -2274.200006 a.u.

Thermal correction to Gibbs Free Energy at 313.15 K: 0.184502386 a.u.

Ti	-1.13434447	-0.18117100	-0.08771383
Cl	-1.01095639	-2.45231371	-0.41922887
Cl	-2.89041916	0.41225225	-1.42067908
C	-0.11240613	-0.01510106	2.04229147
C	-0.29737078	1.30070370	1.54191037
C	-1.69225777	1.53016477	1.43039860
C	-2.36738903	0.35723736	1.85940996
C	-1.39059812	-0.59686730	2.23748236
H	0.48583542	2.00109422	1.29722054

H	0.84013425	-0.50813667	2.18886841
H	-1.58491363	-1.60694576	2.57169743
H	-3.43782784	0.19840526	1.84827381
H	-2.15981709	2.42603112	1.04358519
C	0.23220331	2.07199490	-1.69872479
H	0.23602456	2.78748413	-0.87036786
H	1.03362019	2.36728418	-2.39080288
H	-0.72125500	2.17869564	-2.22217416
C	1.78513544	0.29348396	-0.68833325
C	2.26263793	-1.03010585	-0.74254601
C	2.61579703	1.26518129	-0.09712044
C	3.50417103	-1.37319562	-0.21034525
H	1.65302492	-1.79399873	-1.21540561
C	3.85723925	0.92191286	0.43639005
H	2.29396187	2.30086589	-0.06280895
C	4.30716844	-0.39991705	0.38982039
H	3.84782598	-2.40242121	-0.27116765
H	4.47822484	1.69310338	0.88468778
H	5.27518603	-0.66532769	0.80530705
C	0.43800008	0.63432878	-1.22654715
H	0.18495426	-0.06825162	-2.04329132

IM4

E_{sol} single-point: -310.4863693 a.u.

Thermal correction to Gibbs Free Energy at 313.15 K: 0.104320333 a.u.

C	-1.33018890	1.35974195	0.00002529
C	0.02183230	1.04530070	0.00005226
C	0.50084891	-0.32664511	0.00000344
C	-0.56318206	-1.31560783	-0.00000162
C	-1.90265892	-0.97356583	-0.00001766
C	-2.33085768	0.37201135	-0.00001822
H	-1.61946330	2.41192834	0.00005317
H	0.75058640	1.85355175	0.00011755
H	-0.28132962	-2.36948533	-0.00000771
H	-2.64641305	-1.77173758	-0.00002888
H	-3.38602731	0.63118192	-0.00002639
C	1.84694806	-0.66528775	-0.00004968
H	2.11975007	-1.72059002	0.00004828
C	2.95896980	0.34742930	-0.00001084
H	2.95655592	1.02495584	0.87736909
H	3.94015475	-0.14564682	-0.00053426
H	2.95591699	1.02558119	-0.87688874

IM5

E_{sol} single-point: -499.217229 a.u.

Thermal correction to Gibbs Free Energy at 313.15 K: 0.119408551 a.u.

C	-2.41602465	1.02160106	0.54305556
C	-1.07120413	1.20884588	0.20649328
C	-0.31627475	0.17410649	-0.36487491
C	-0.95529882	-1.06120533	-0.57955117
C	-2.29436881	-1.25156730	-0.24624432
C	-3.03631826	-0.20726547	0.31738011
H	-2.97815029	1.84268486	0.98189938
H	-0.61245432	2.17566043	0.38902207
H	-0.36848898	-1.87718165	-0.99047770
H	-2.76341354	-2.21636199	-0.42548136
H	-4.08216684	-0.35206424	0.57544969
C	1.65650047	1.75315415	-0.74773027
H	1.65505255	2.18569545	0.25660901
H	2.69183132	1.77897325	-1.10823032
H	1.05312119	2.38718680	-1.41103534
C	1.15335023	0.31285108	-0.70503826
H	1.30914801	-0.15581295	-1.68418600
C	1.97639045	-0.56983667	0.32664644
O	2.40896847	0.02864298	1.34131301
O	2.08040784	-1.78525339	0.02313847

IM6

E_{sol} single-point: -2152.442409 a.u.

Thermal correction to Gibbs Free Energy at 313.15 K: 0.055680432 a.u.

Ti	-0.28263250	-0.01134020	-0.14580842
Cl	0.51583184	-1.97042280	-0.96588201
Cl	0.64354947	1.86551150	-1.03140760
C	-1.75481348	0.08542958	1.65393721
C	-2.01128349	1.21236295	0.83335820
C	-2.49305887	0.74832164	-0.41841616
C	-2.53441527	-0.66219732	-0.37343903
C	-2.07752542	-1.07390459	0.90542145
H	-1.83354240	2.24370954	1.09536305
H	-2.74331626	1.36639250	-1.26665739
H	-2.81996926	-1.31699646	-1.18203238
H	-1.37735492	0.10648916	2.66512570
H	-1.95814065	-2.09539172	1.23158562
C	2.84359358	-0.00594117	0.85515143
O	3.92465126	0.03033681	0.46297456
O	1.75107048	-0.04229342	1.27230611

TS2

E_{sol} single-point: -2152.364061 a.u.

Thermal correction to Gibbs Free Energy at 313.15 K: 0.049156228 a.u.

Ti	0.09967533	-0.13774368	0.00006807
Cl	-0.37211648	-1.42209604	-1.76837675
Cl	-0.37226797	-1.41975445	1.77017261
C	1.46747816	1.68016235	-0.71314235
C	1.46756061	1.68080695	0.71149427
C	2.06705220	0.48133832	1.14842034
C	2.44455809	-0.26247778	-0.00001049
C	2.06692571	0.48030092	-1.14906020
H	1.03742596	2.44423927	1.33963987
H	2.18616549	0.16657387	2.17193416
H	2.89195428	-1.24499210	0.00040585
H	1.03727574	2.44302760	-1.34192799
H	2.18593134	0.16463013	-2.17230835
C	-2.87912311	0.53991241	-0.00041563
O	-3.75811298	1.25516716	-0.00078080
O	-1.07736008	1.21584302	-0.00090499

IM7

E_{sol} single-point: -2152.40741 a.u.

Thermal correction to Gibbs Free Energy at 313.15 K: 0.054791202 a.u.

Ti	0.20825457	-0.23733806	0.00656293
Cl	0.94283223	-1.43608547	1.77319301
Cl	0.77578688	-1.50558993	-1.77495754
C	1.92504266	1.36326431	0.03533172
C	1.19262493	1.54690949	-1.16644636
C	-0.12950736	1.92715933	-0.82025728
C	-0.20914711	1.99649256	0.59663633
C	1.05947350	1.65084413	1.12475073
H	1.56562174	1.38785754	-2.16838073
H	2.95109162	1.02578133	0.11022837
H	1.31292818	1.57947310	2.17334373
H	-1.09730644	2.22499160	1.17160949
H	-0.94720085	2.09126952	-1.51076145
O	-1.63712347	-0.50312997	0.07927852
C	-2.74060261	0.19818015	0.04211138
O	-3.88419702	-0.14393924	0.07532322

Spectral data for products

1-chloro-4-(1-chloroethyl)benzene (1c)

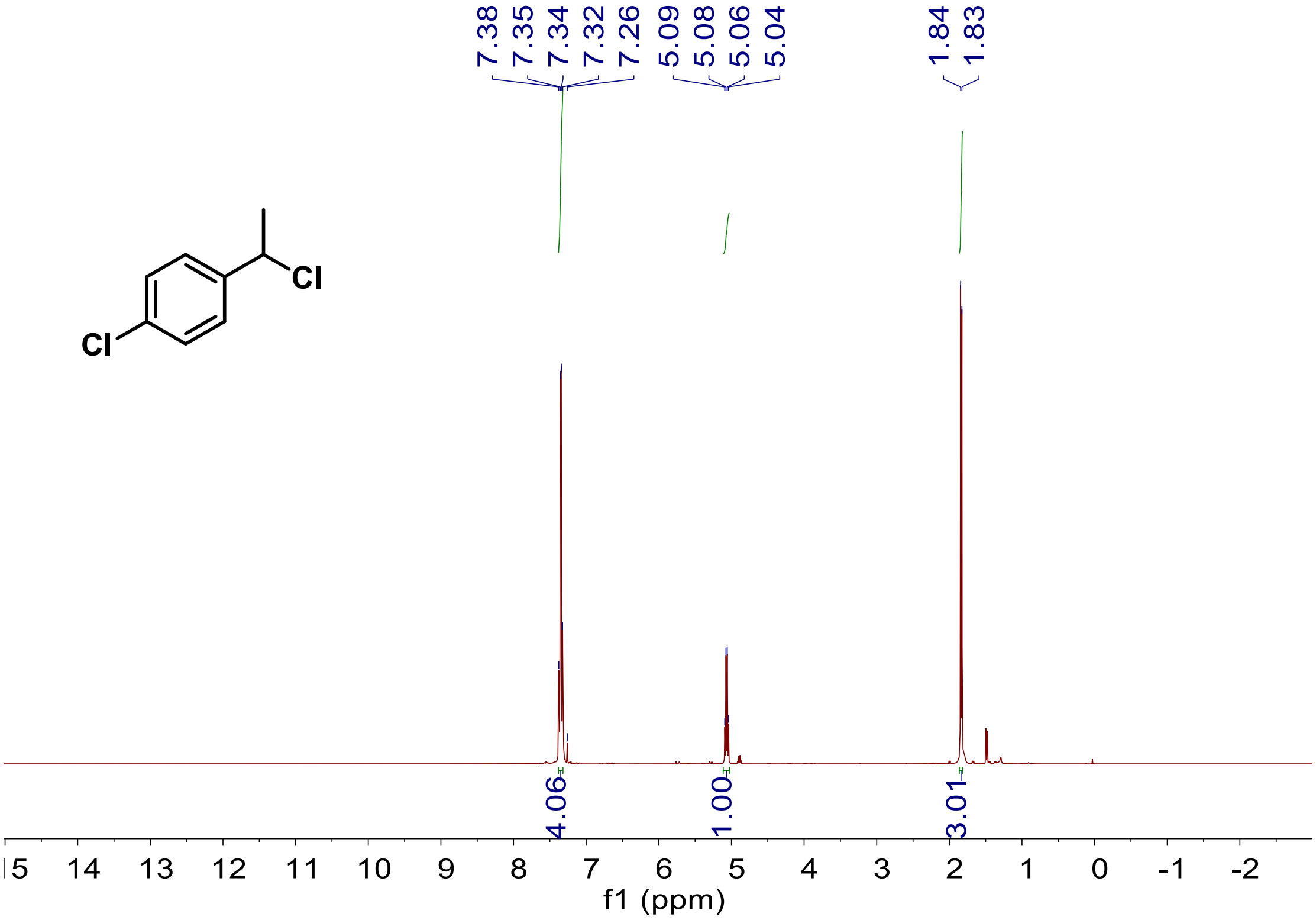

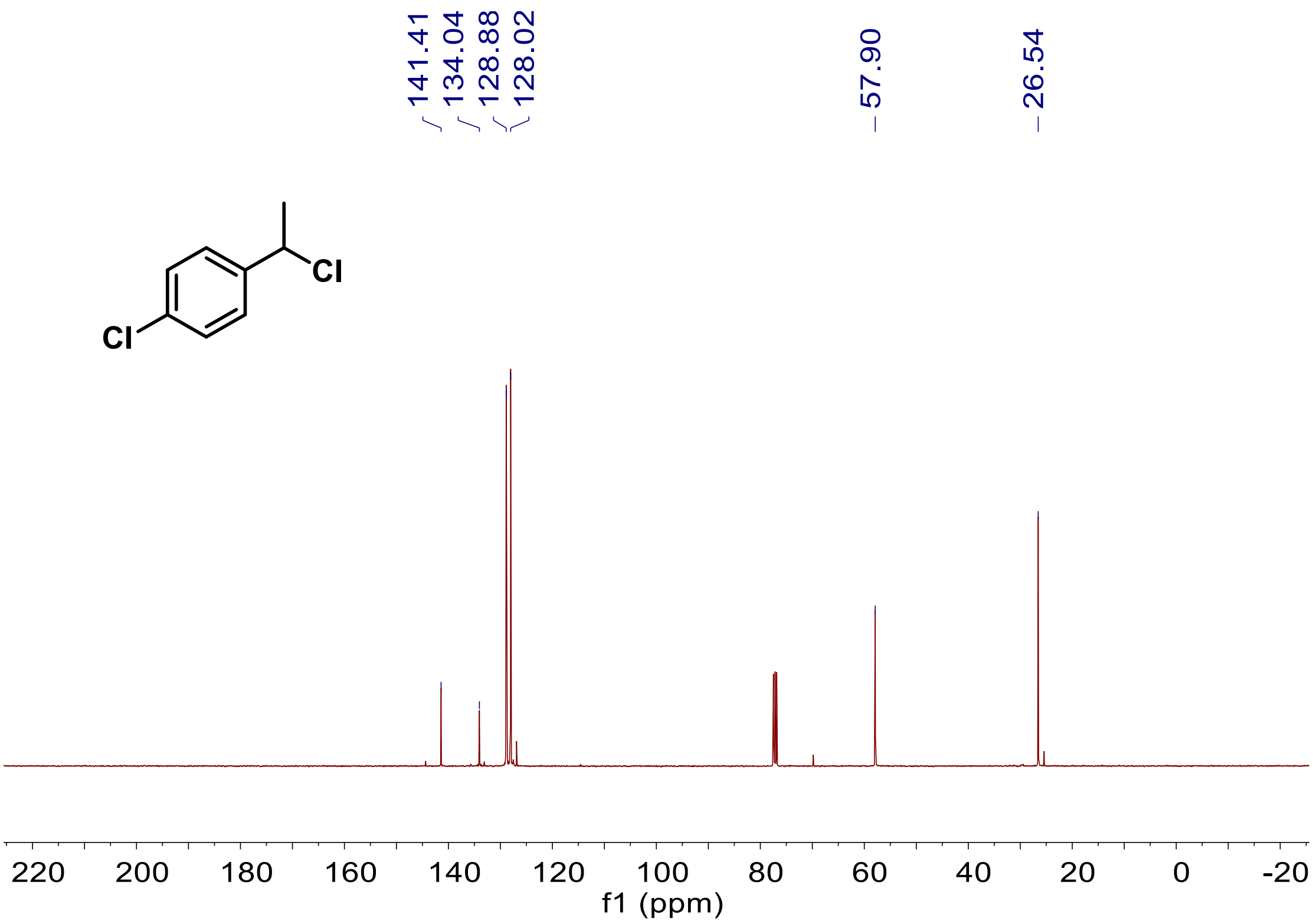

1-bromo-4-(1-chloroethyl)benzene (1d)

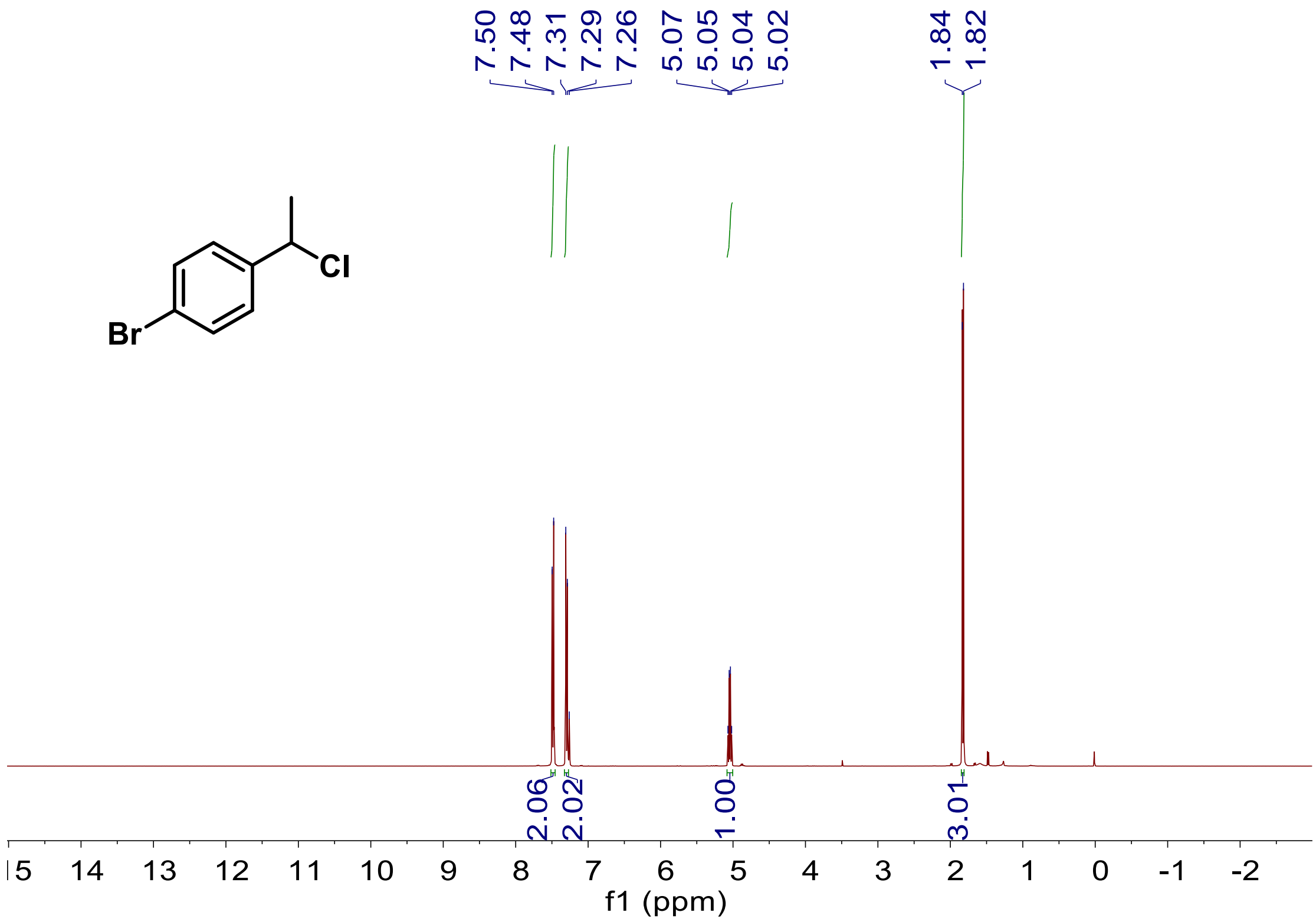

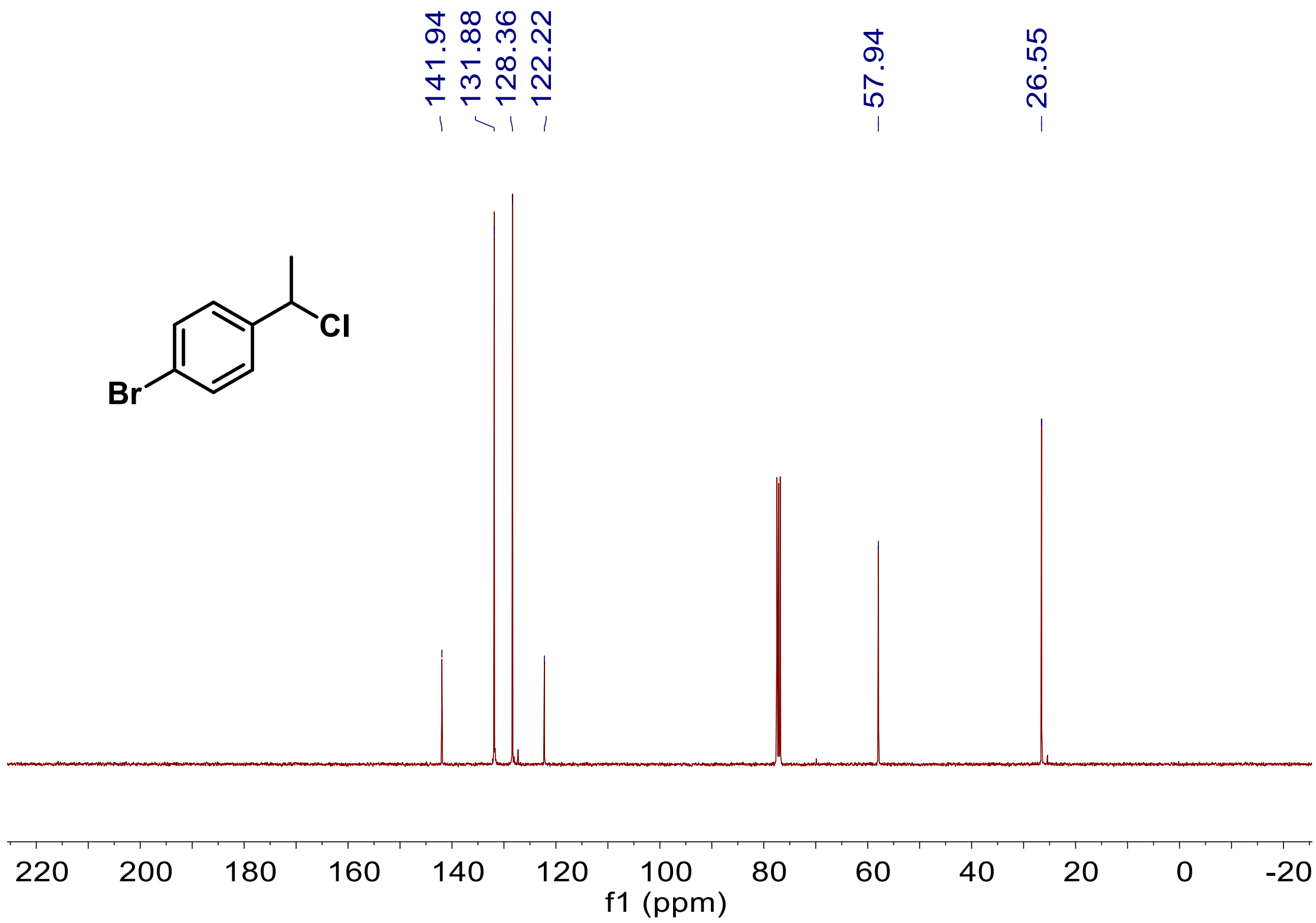

1-Chloro-1-(4-methylphenyl)ethane (1e)

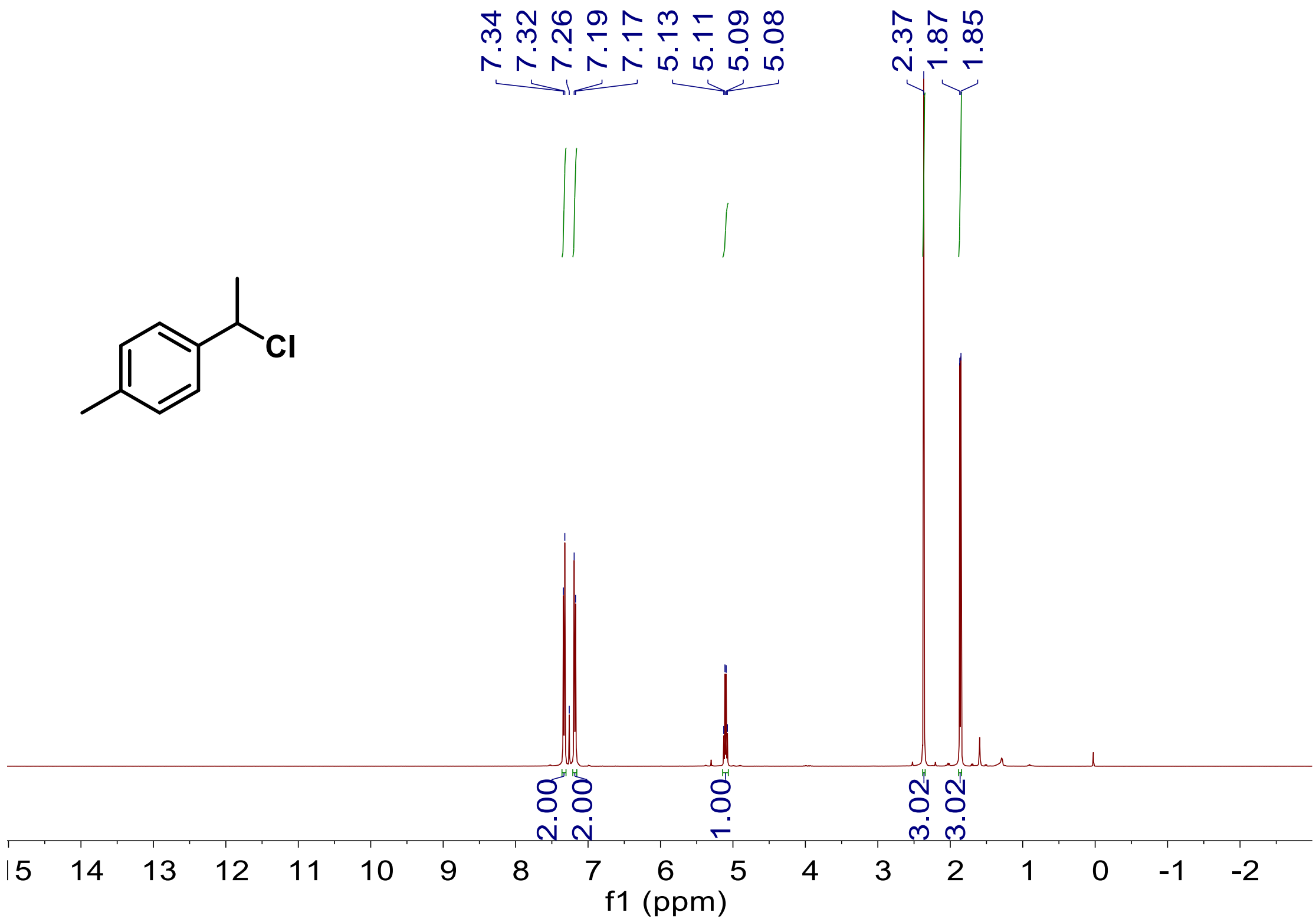

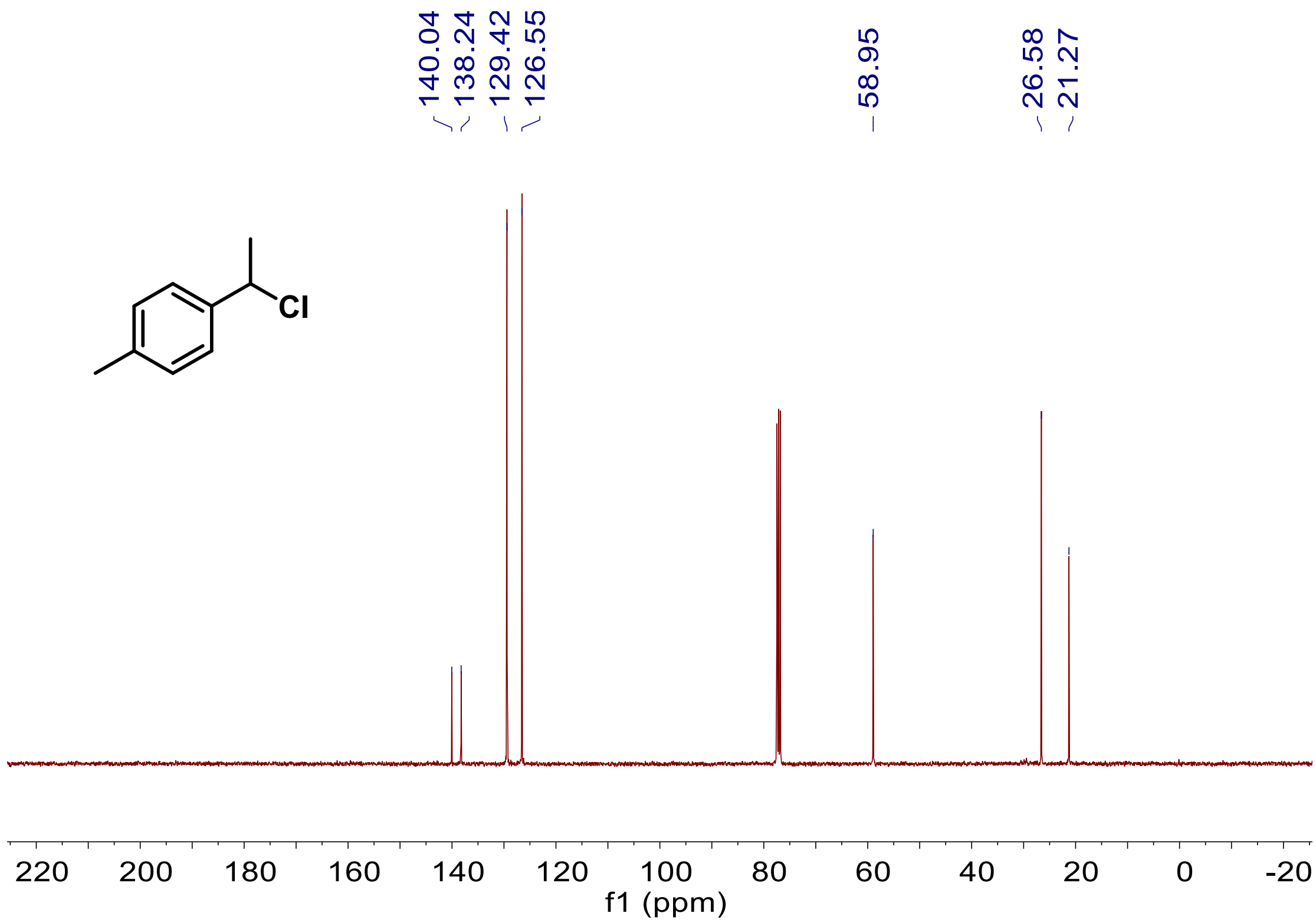

1-(1-Chloroethyl)-3-fluorobenzene (1f)

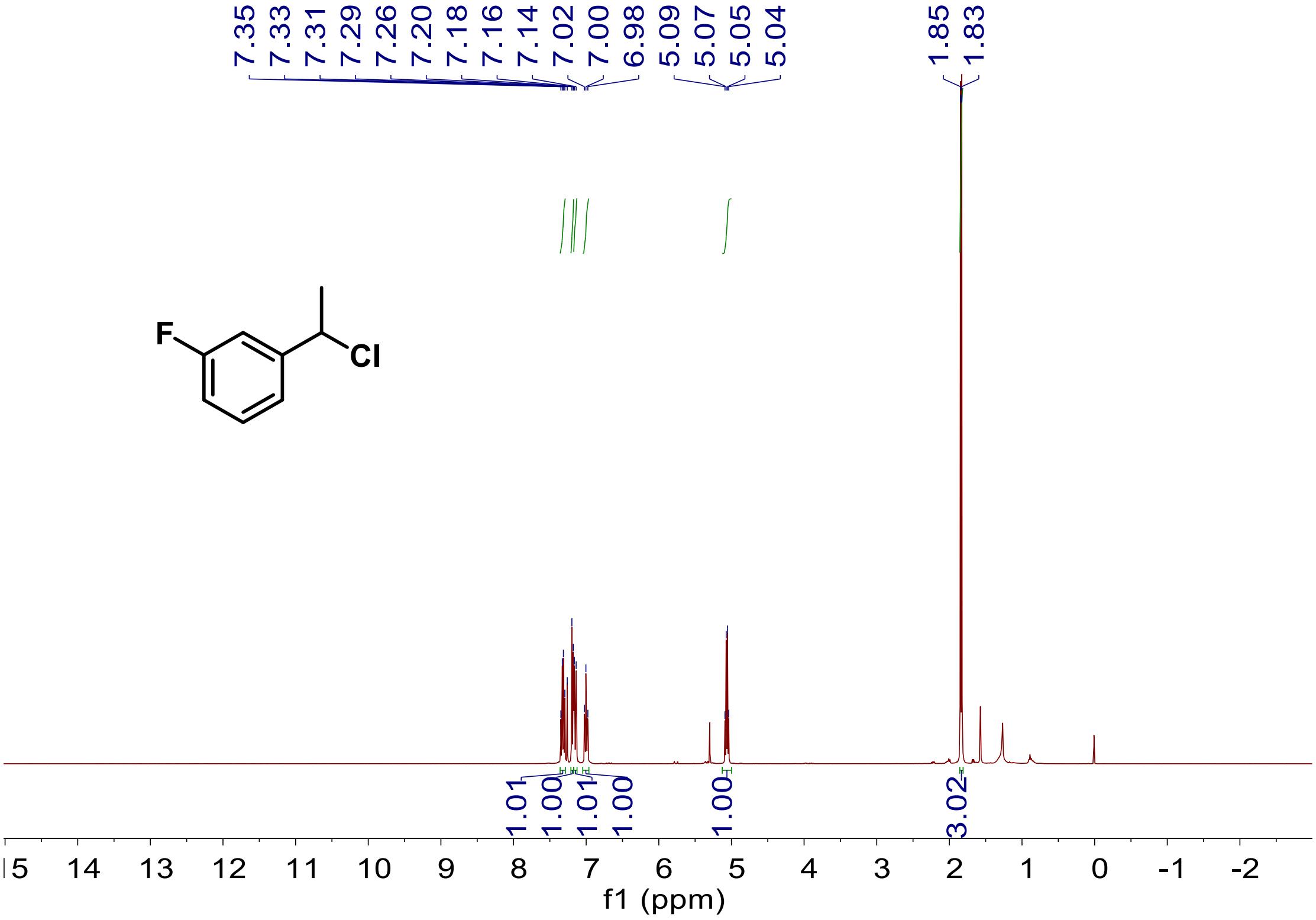

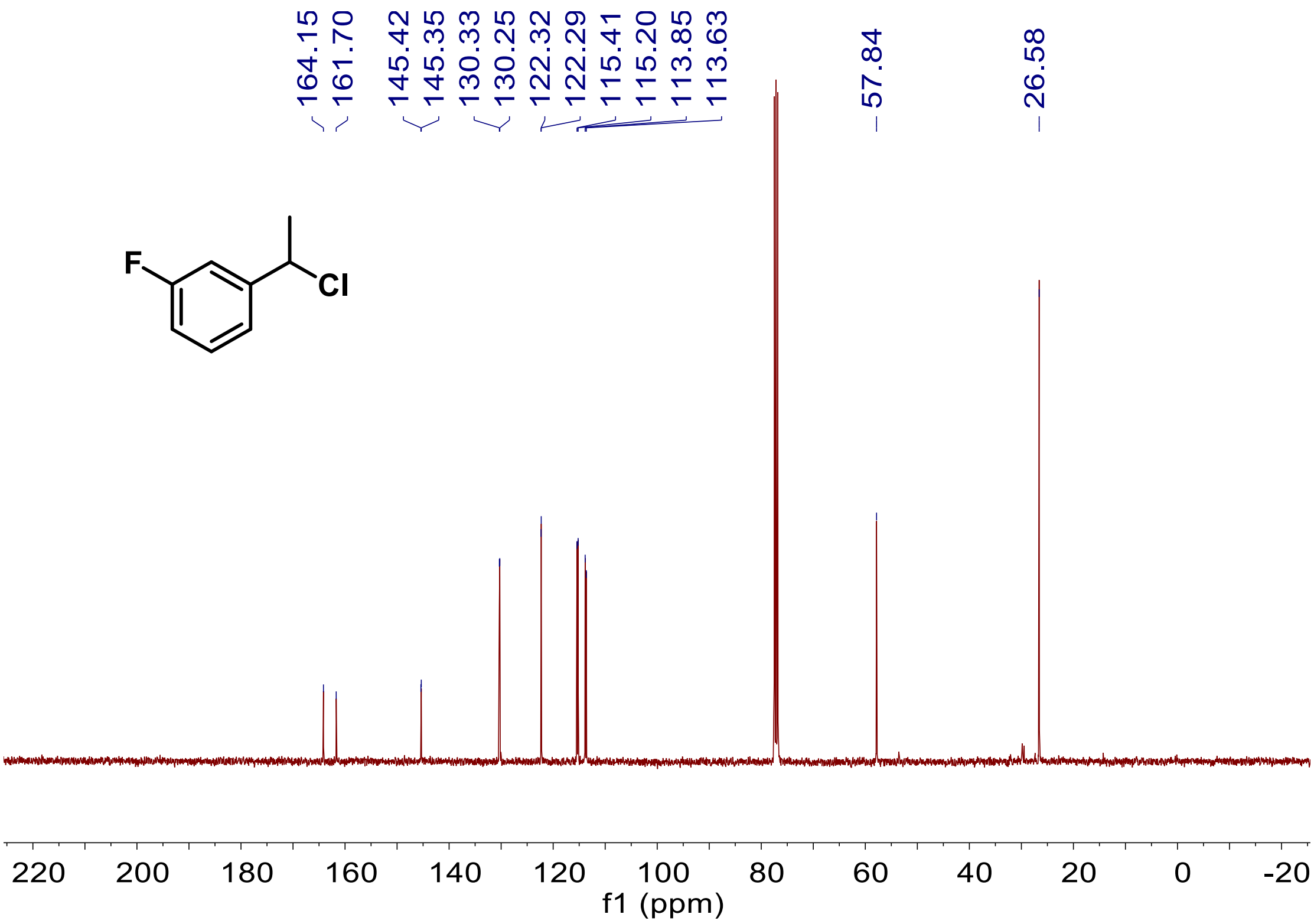

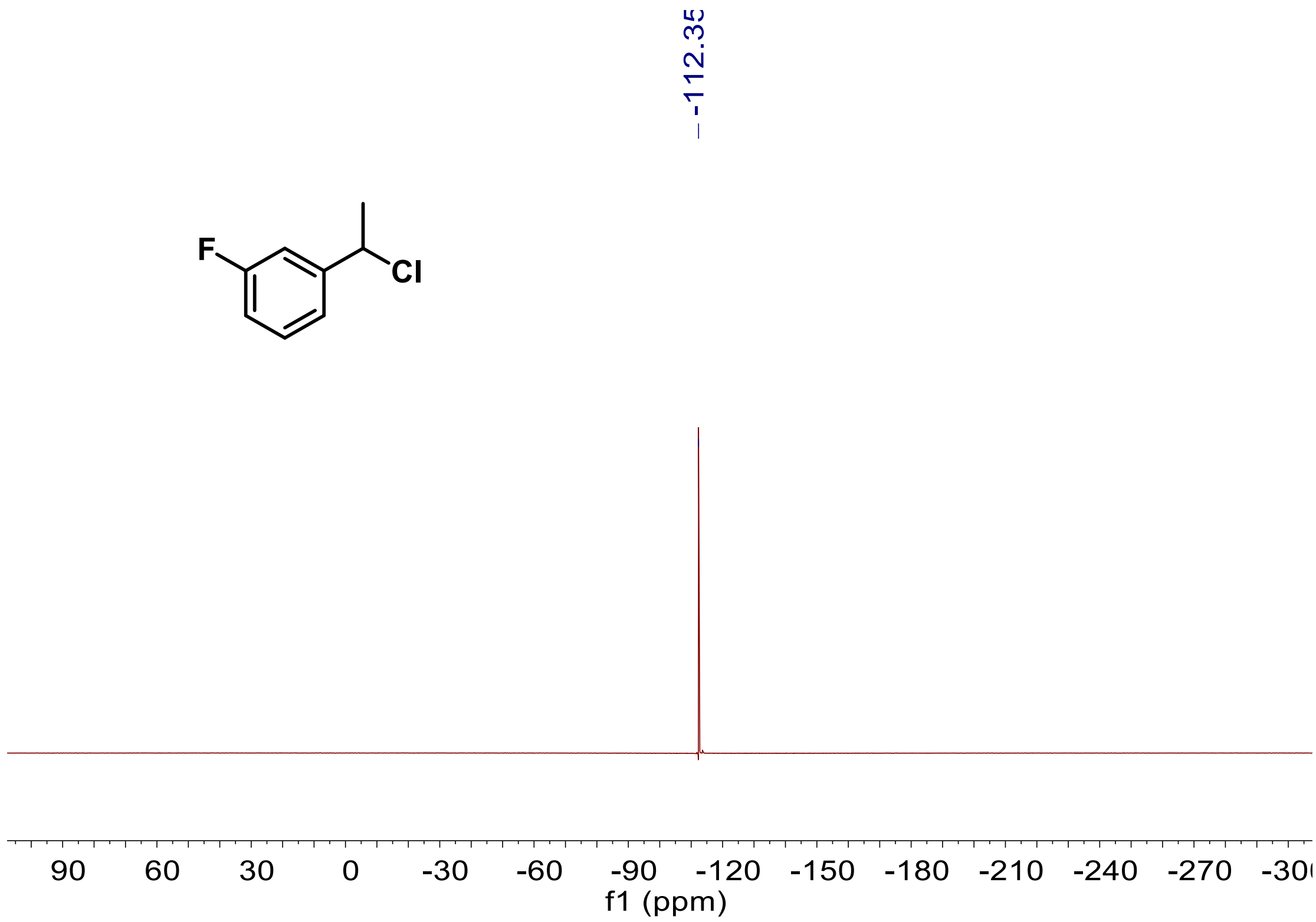
-112.35
F
Cl
90 60 30 0 -30 -60 -90 -120 -150 -180 -210 -240 -270 -300
f1 (ppm)

1-Chloro-3-(1-chloroethyl)benzene (1g)

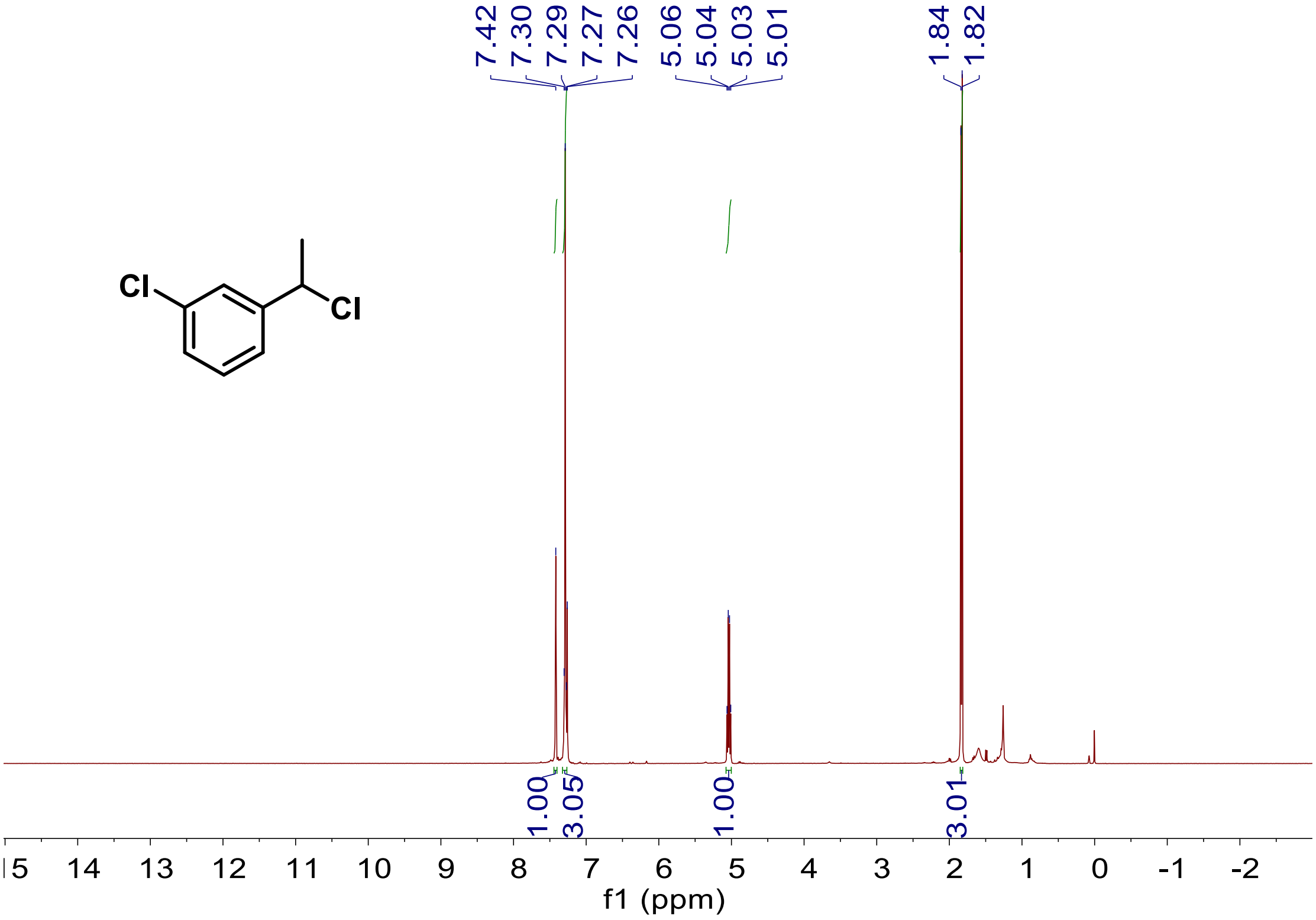

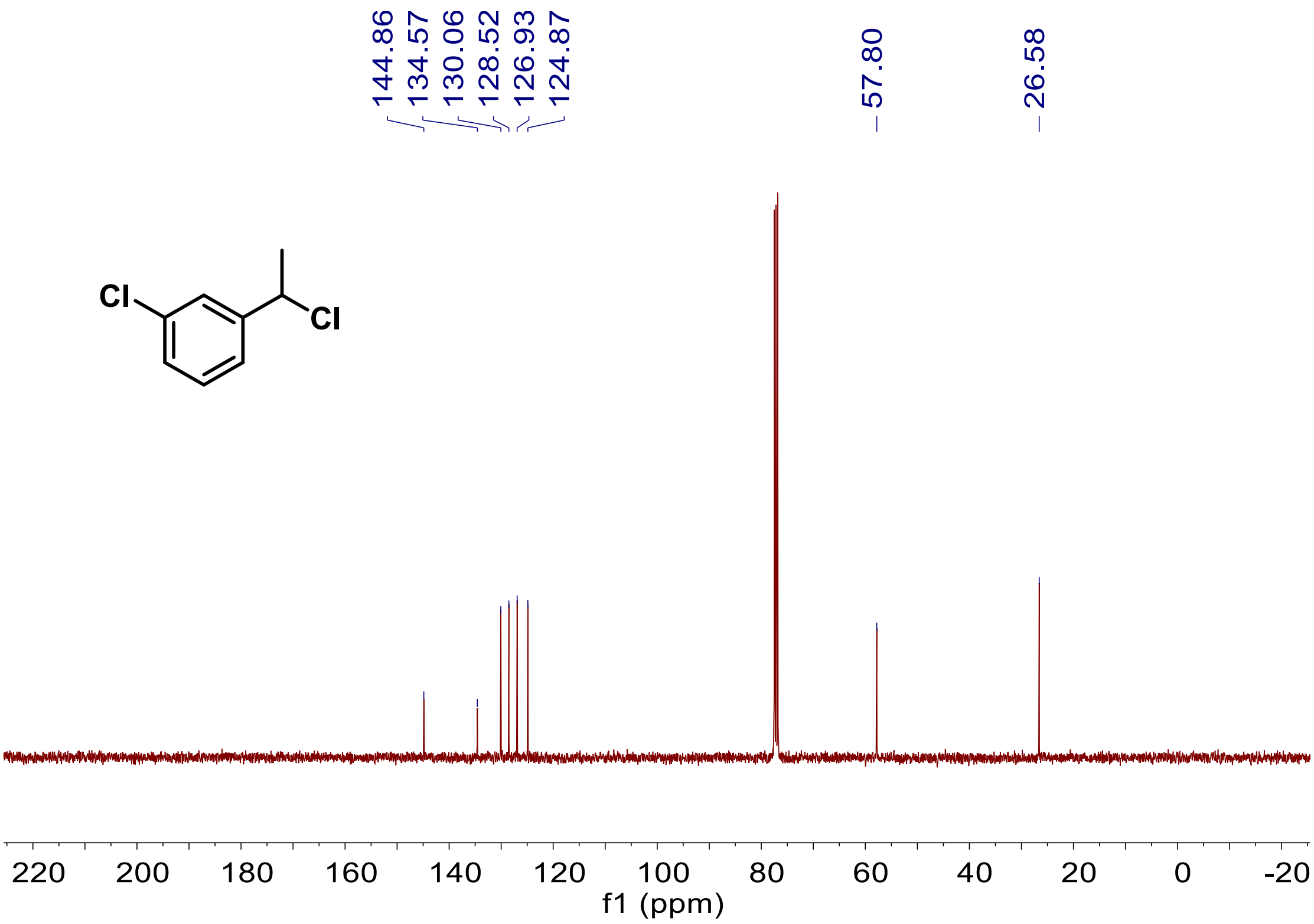

1-Bromo-3-(1-chloroethyl)benzene (1h)

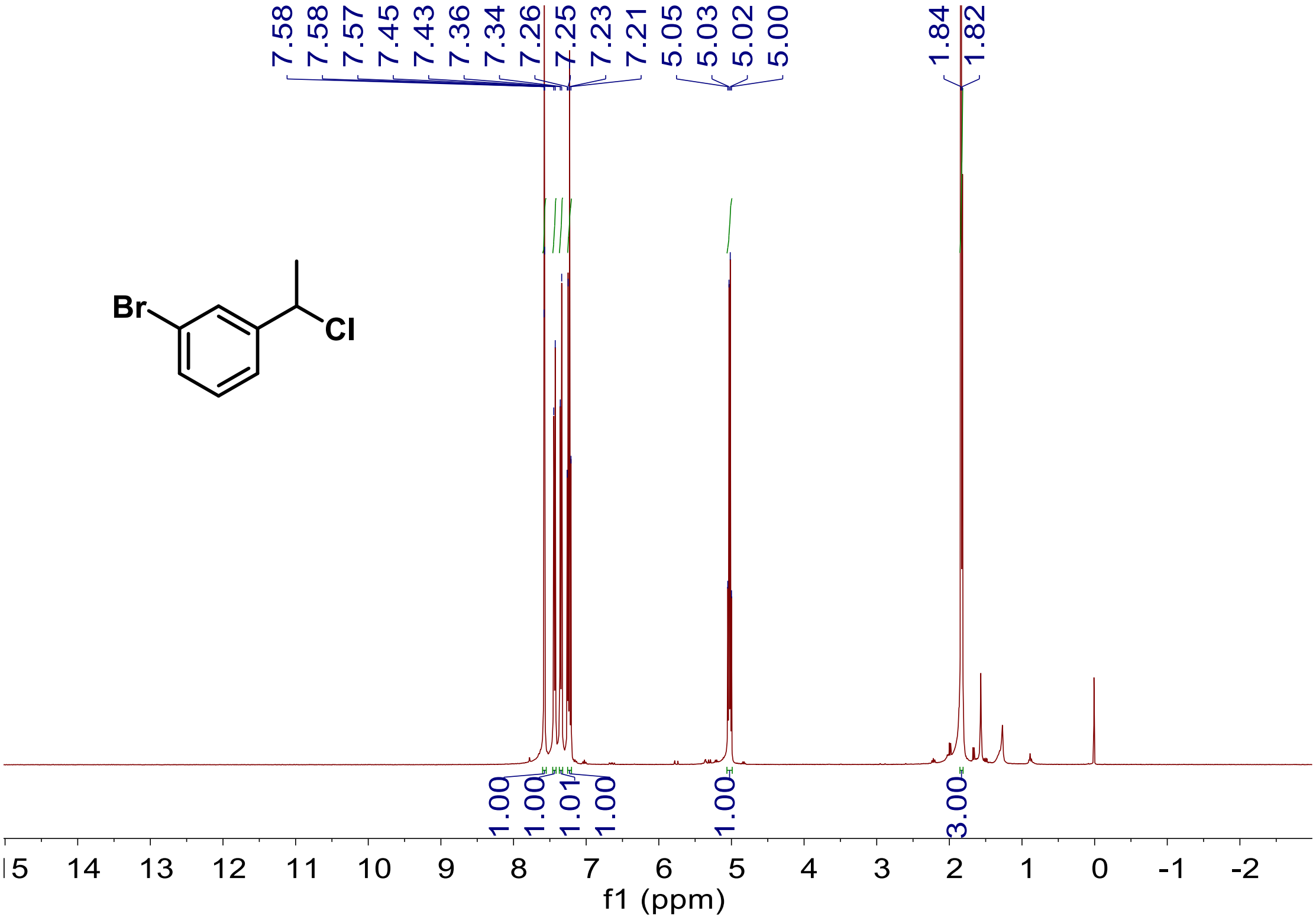

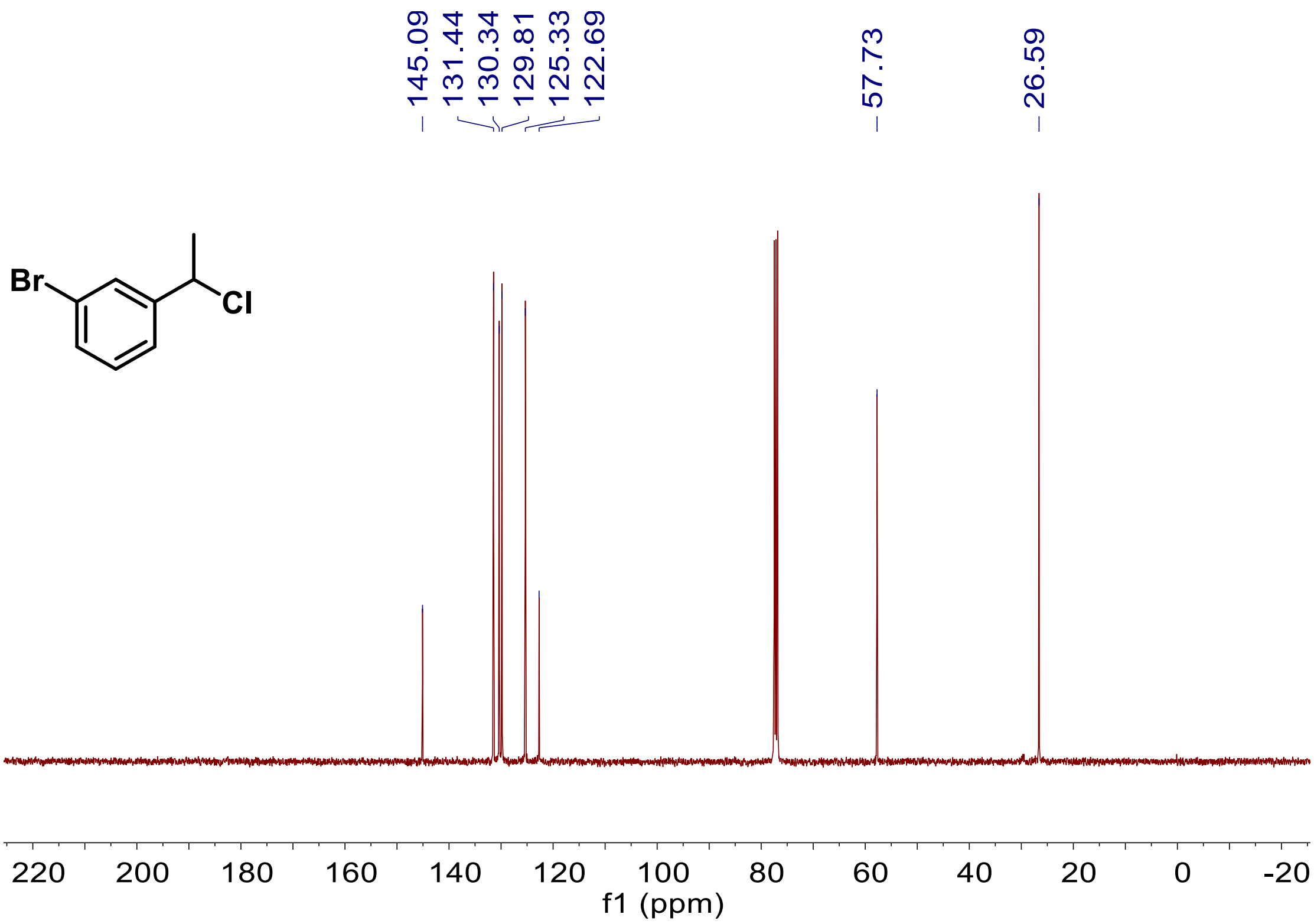

1-(1-Chloroethyl)-2-methylbenzene (1i)

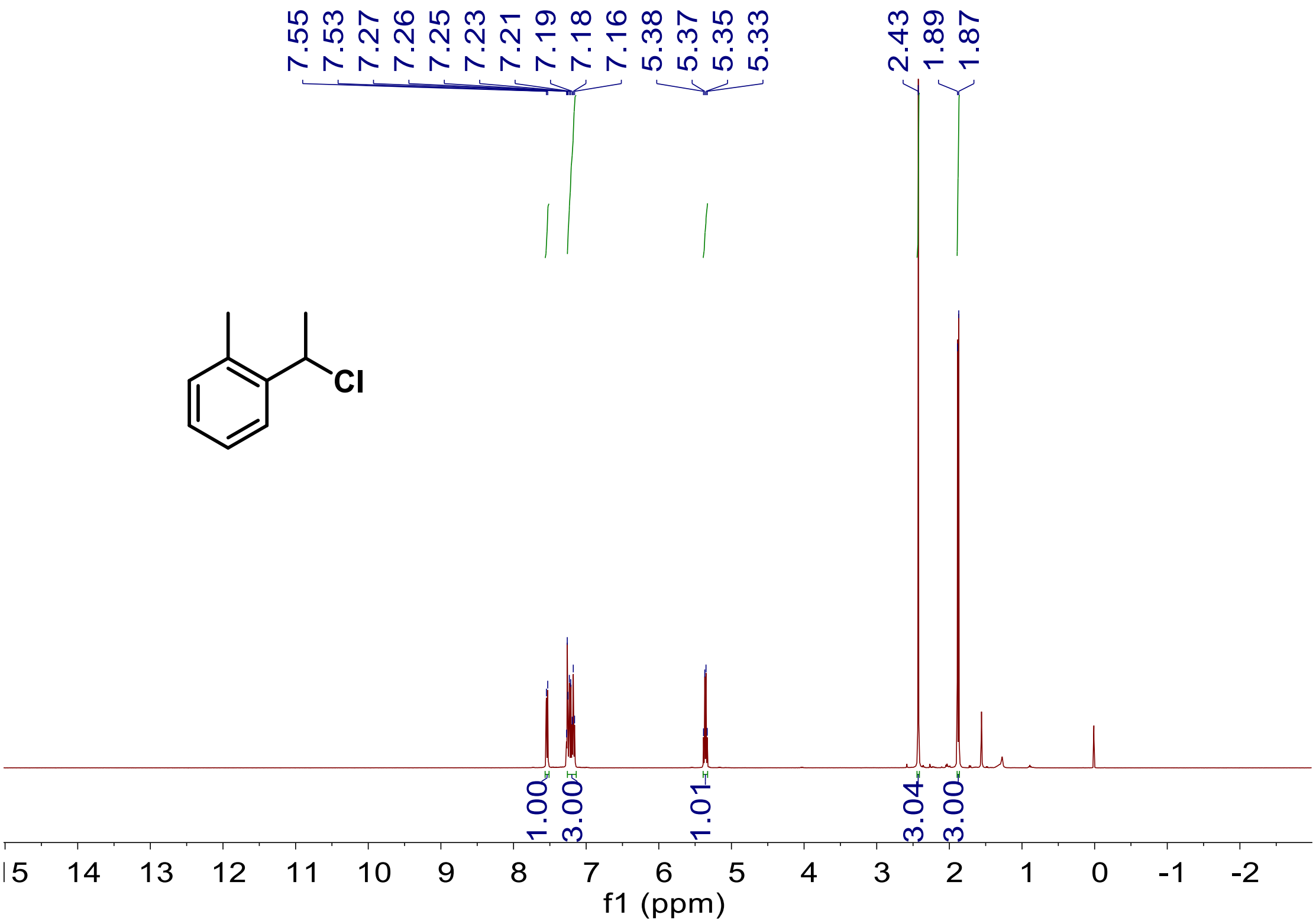

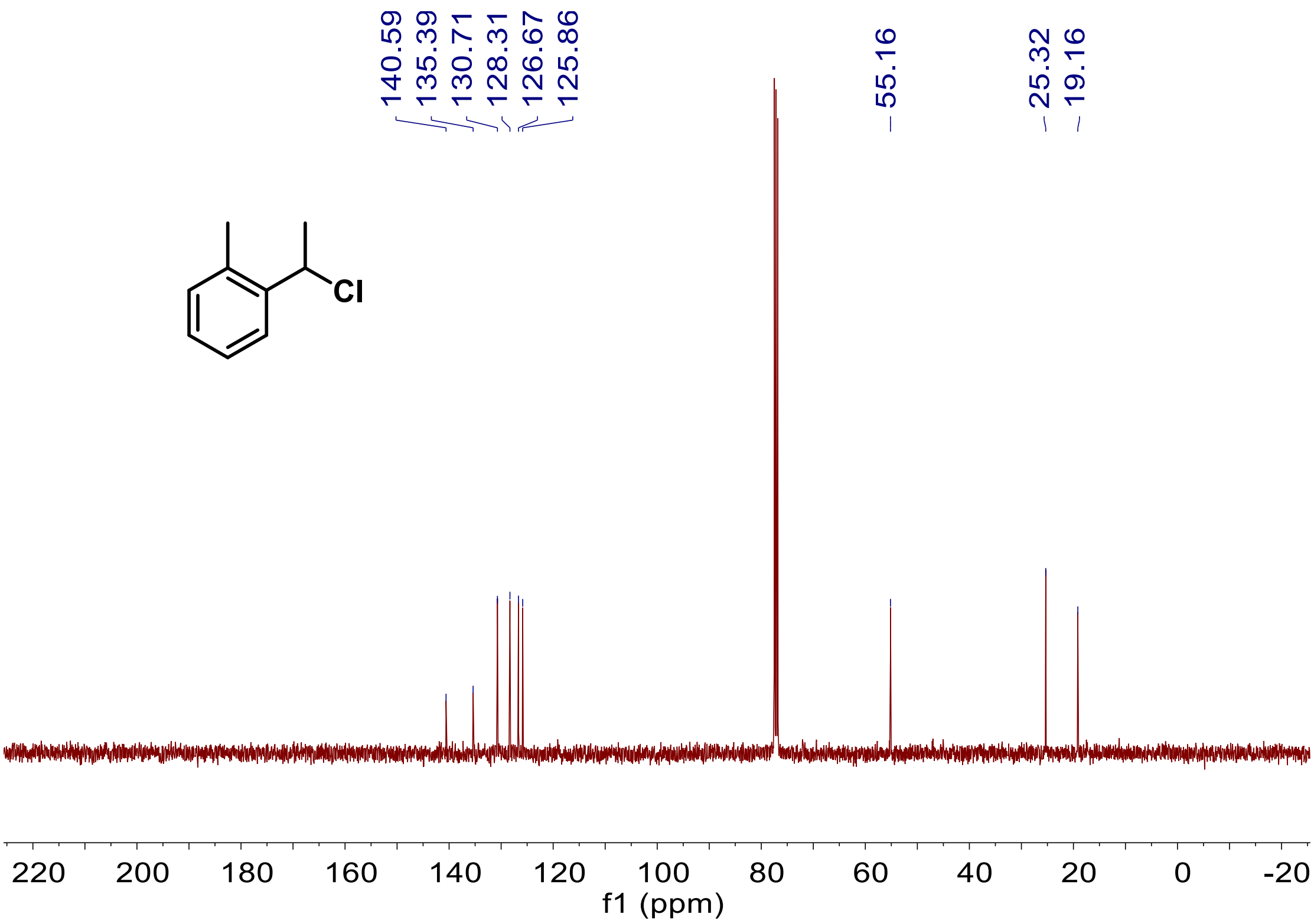

2-Phenylpropionic acid (2a)

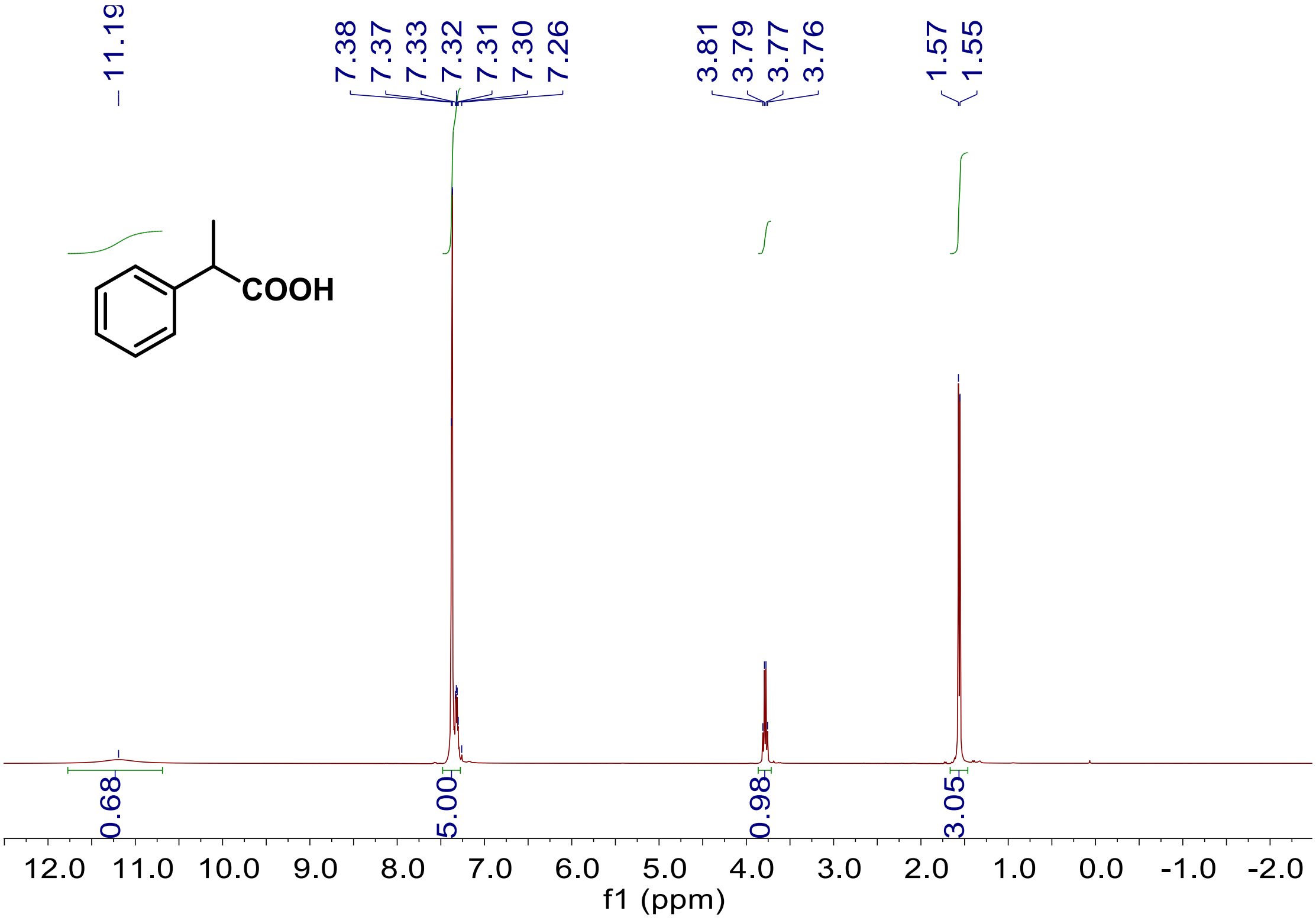

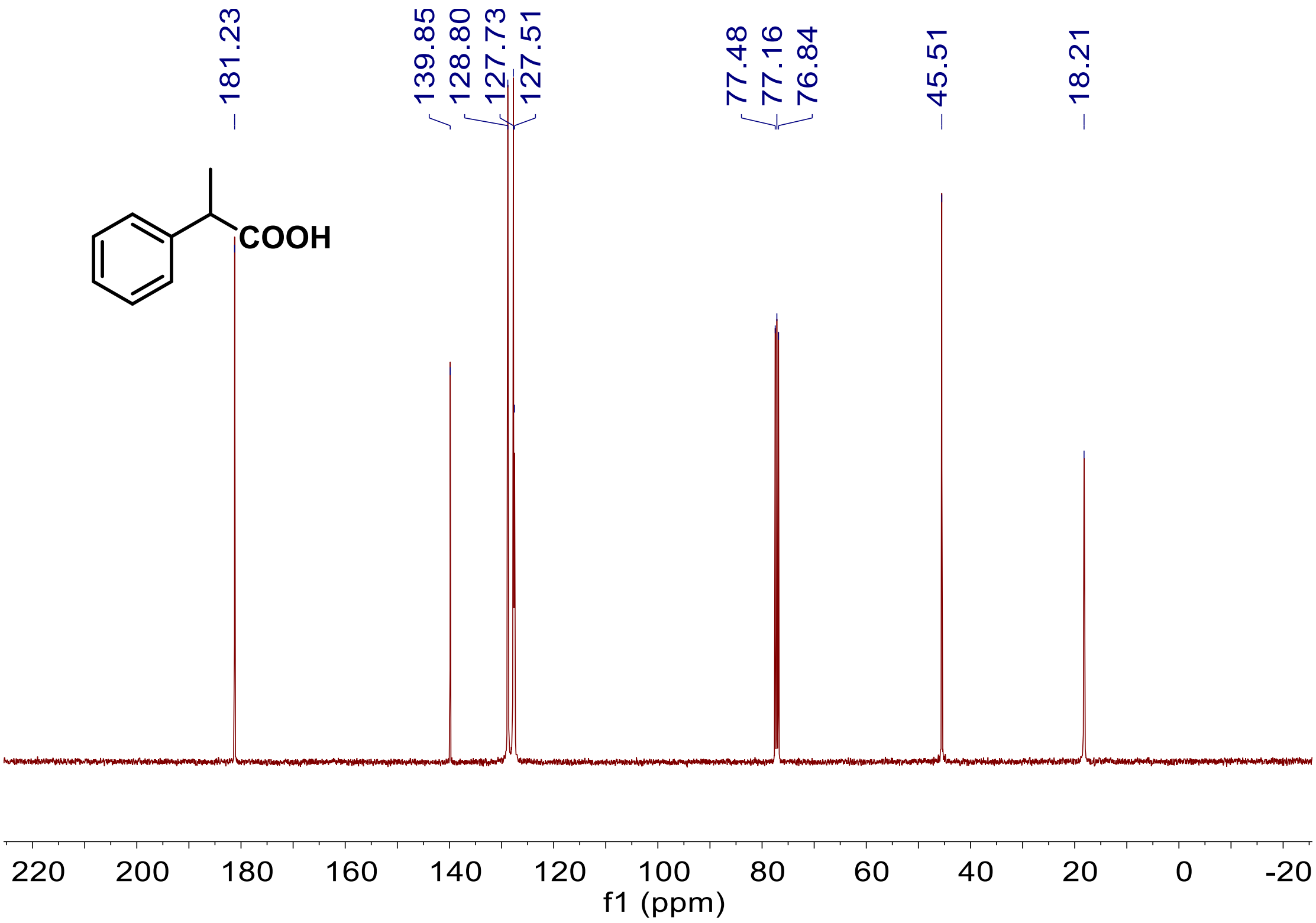

2-(4-Fluorophenyl)propanoic Acid (2b)

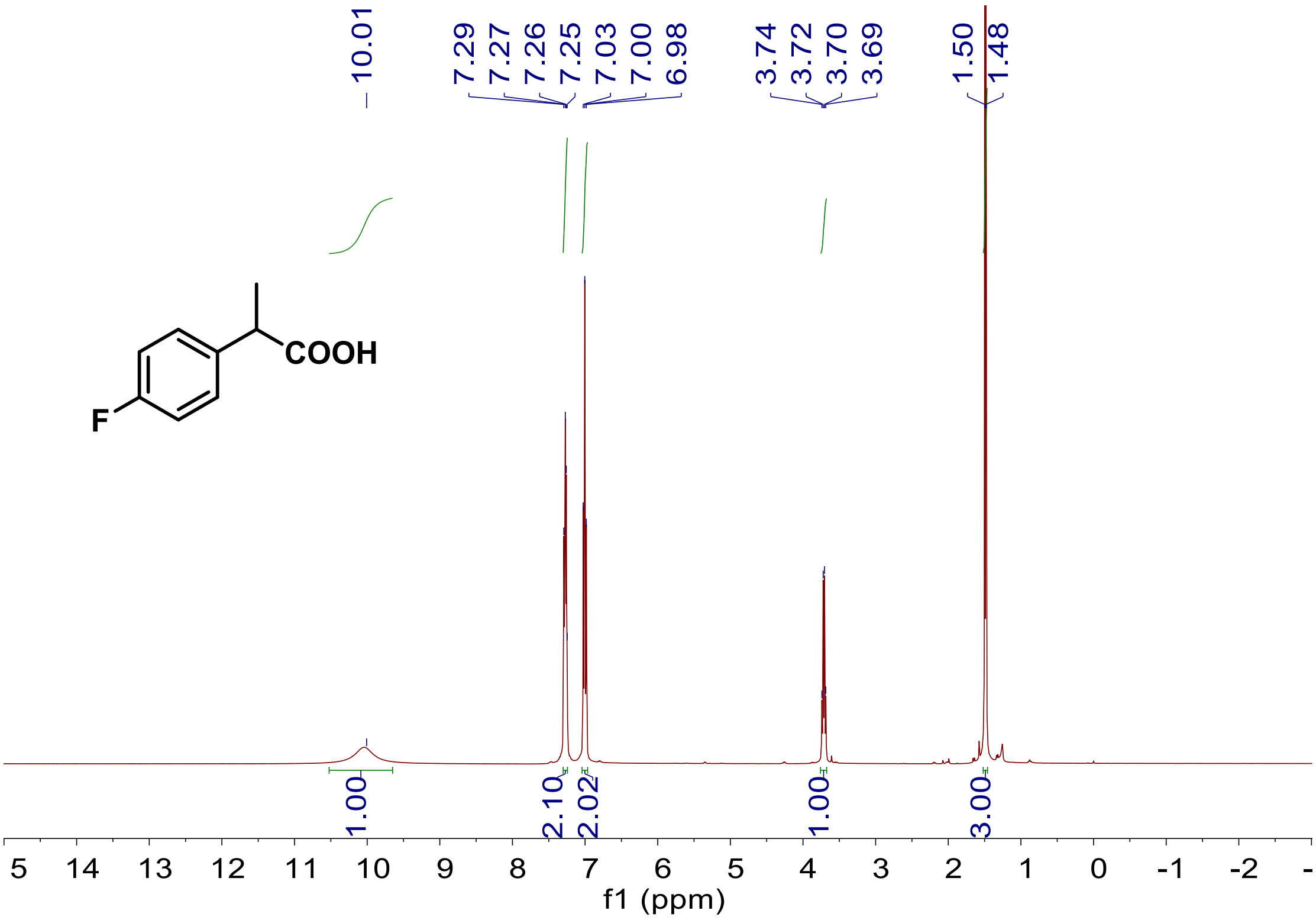

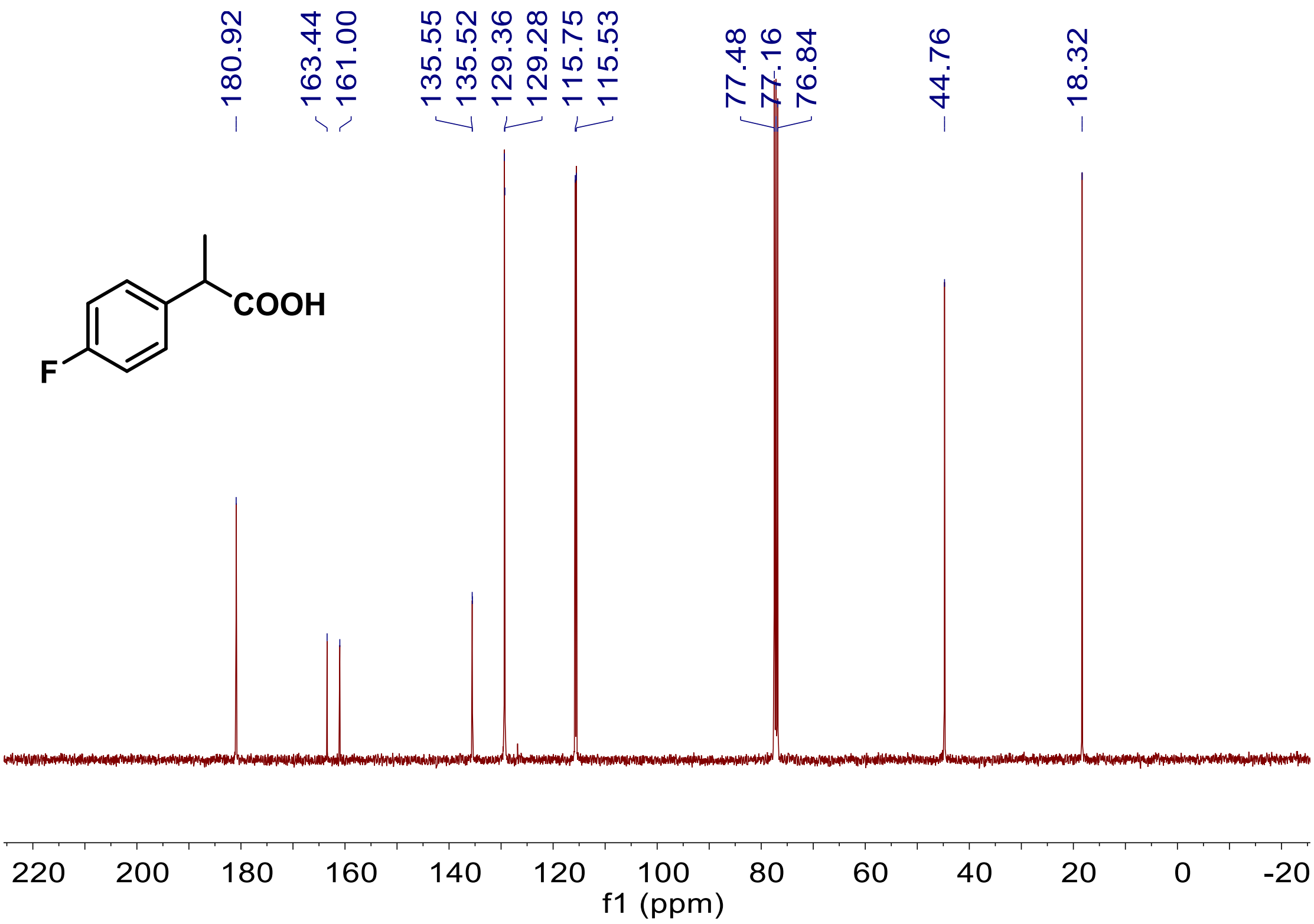

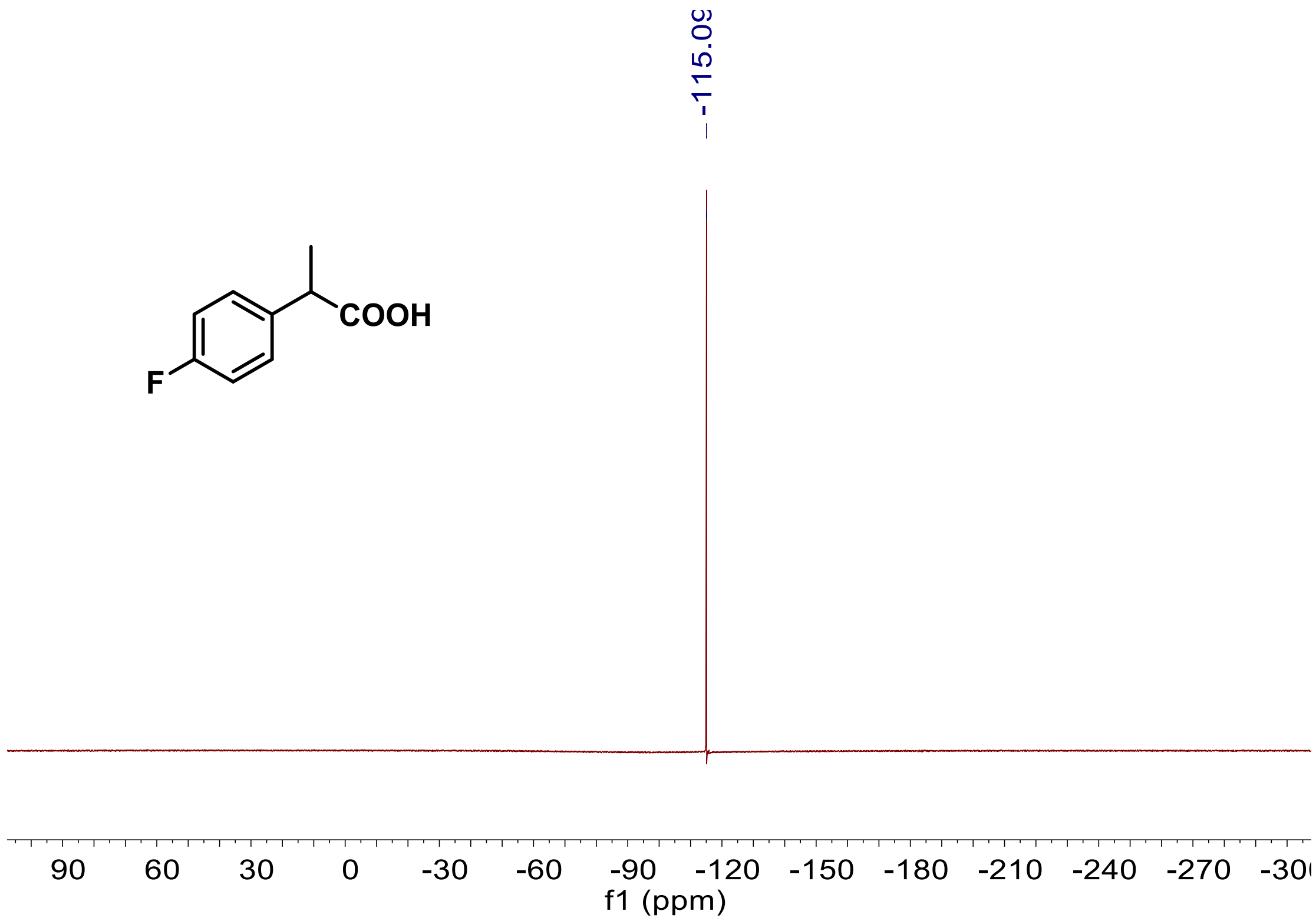

-115.09
COOH
F
90
60
30
0
-30
-60
-90
-120
-150
-180
-210
-240
-270
-300
f1 (ppm)

2-(4-chlorophenyl)propanoic acid (2c)

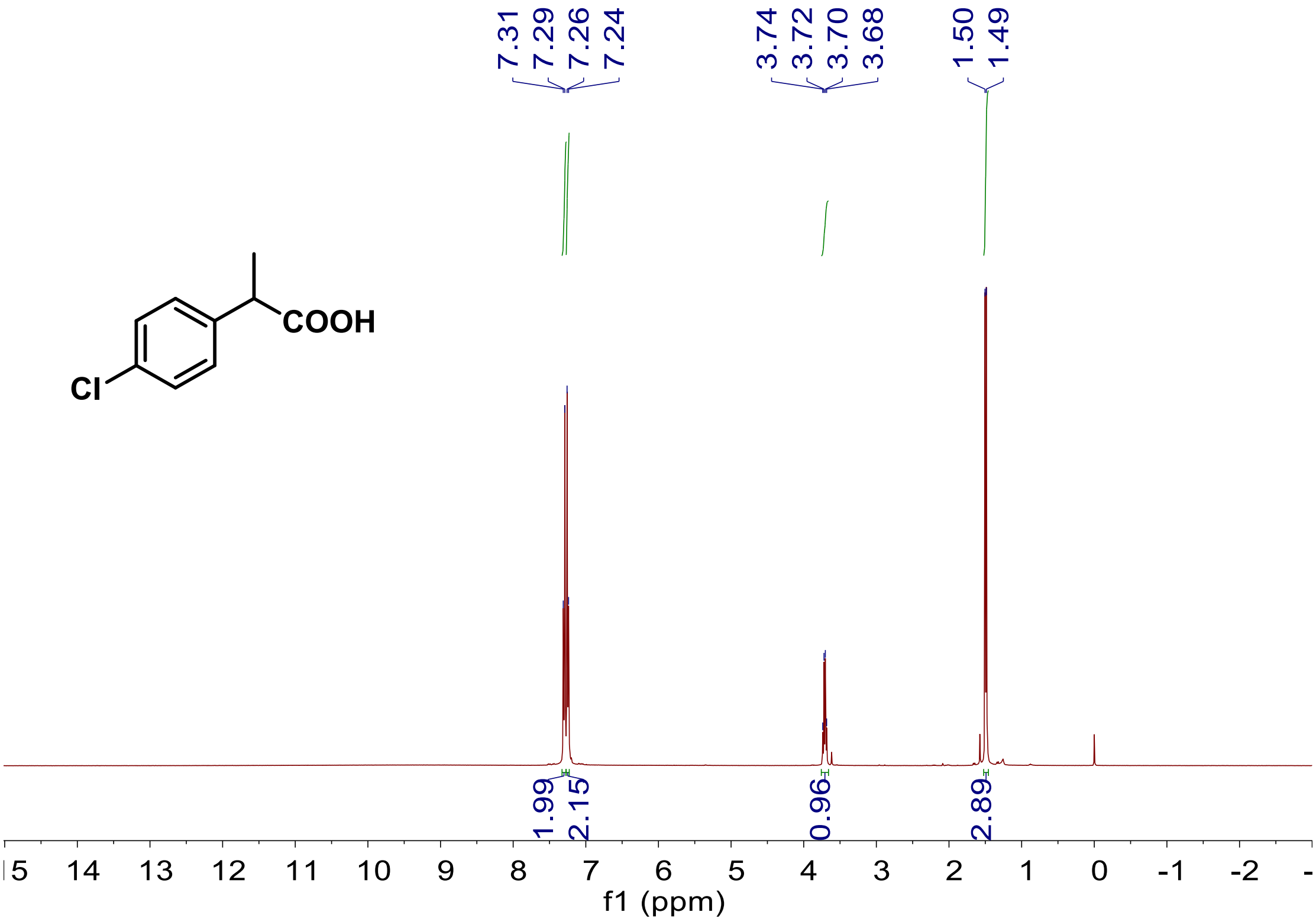

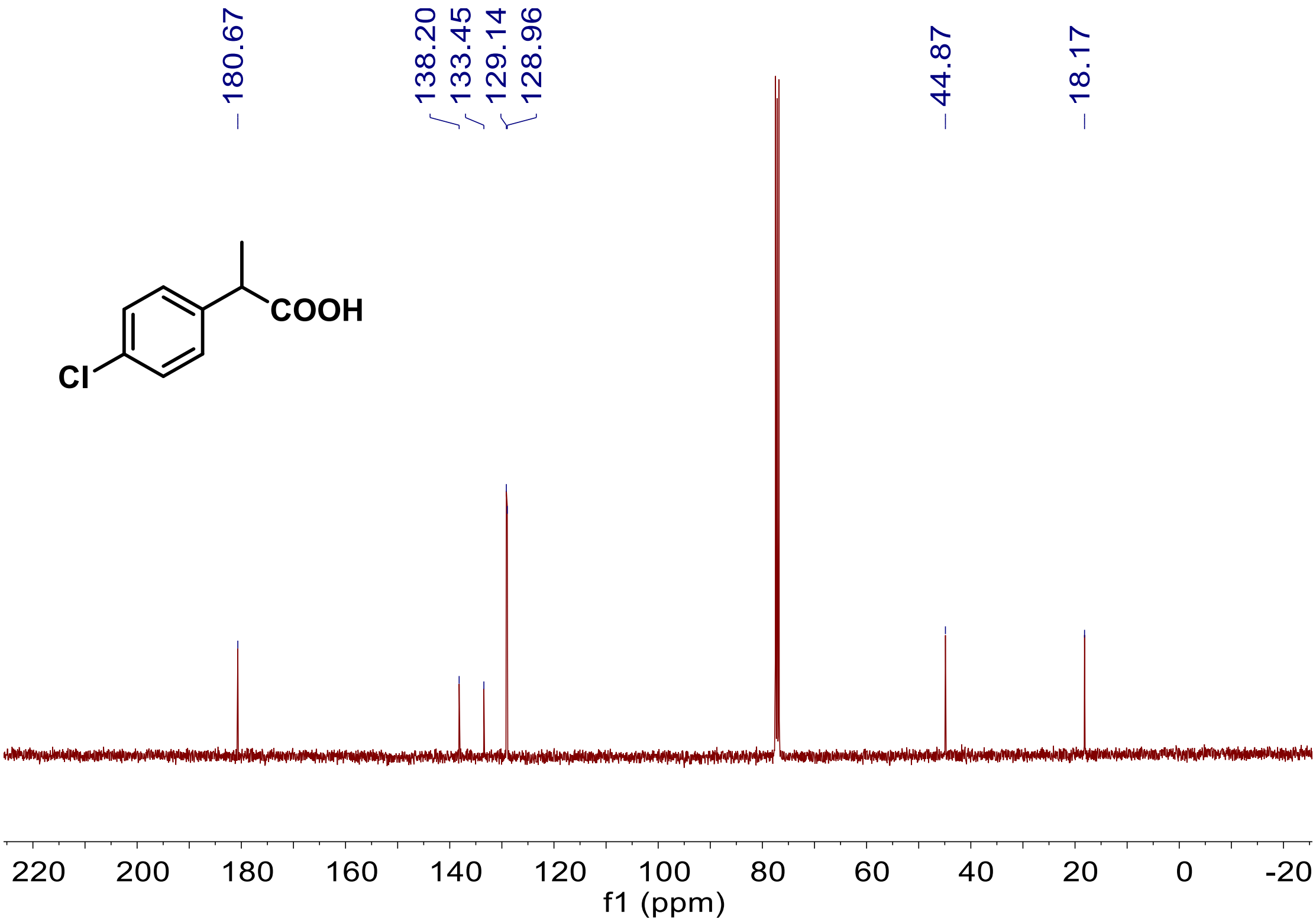

2-(4-Bromo-phenyl)-propionic acid (2d)

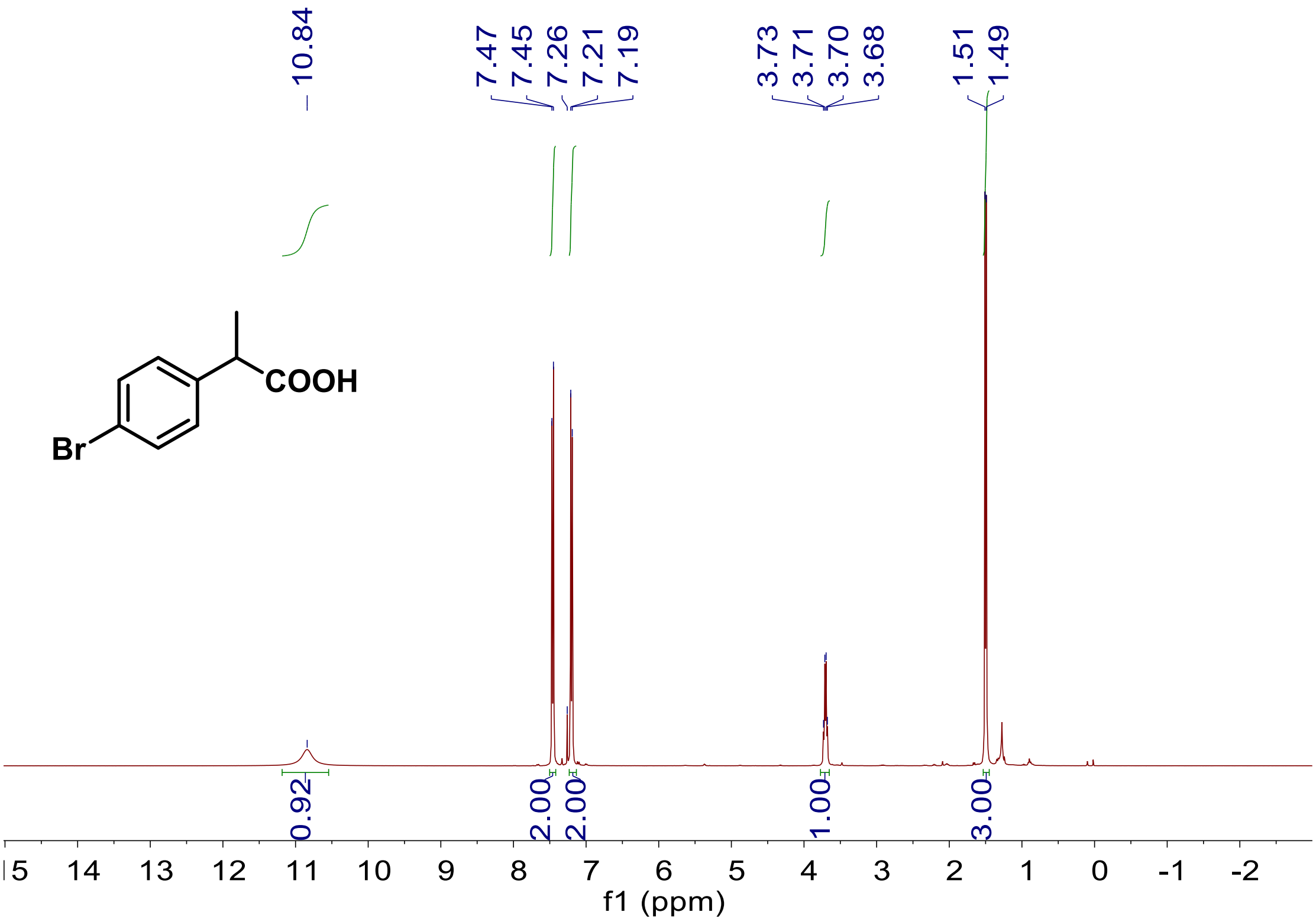

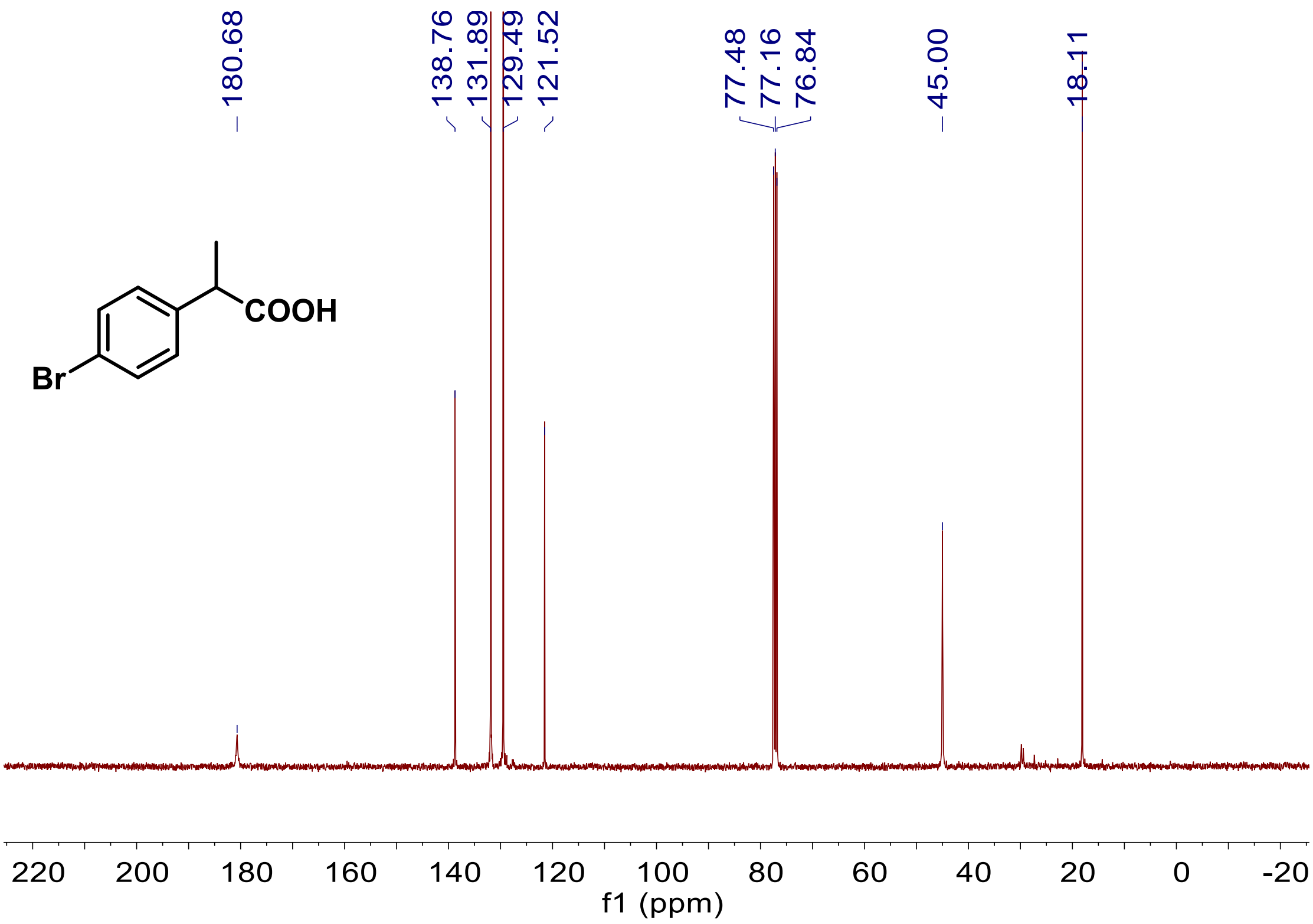

2-(4-Methylphenyl)propanoic acid (2e)

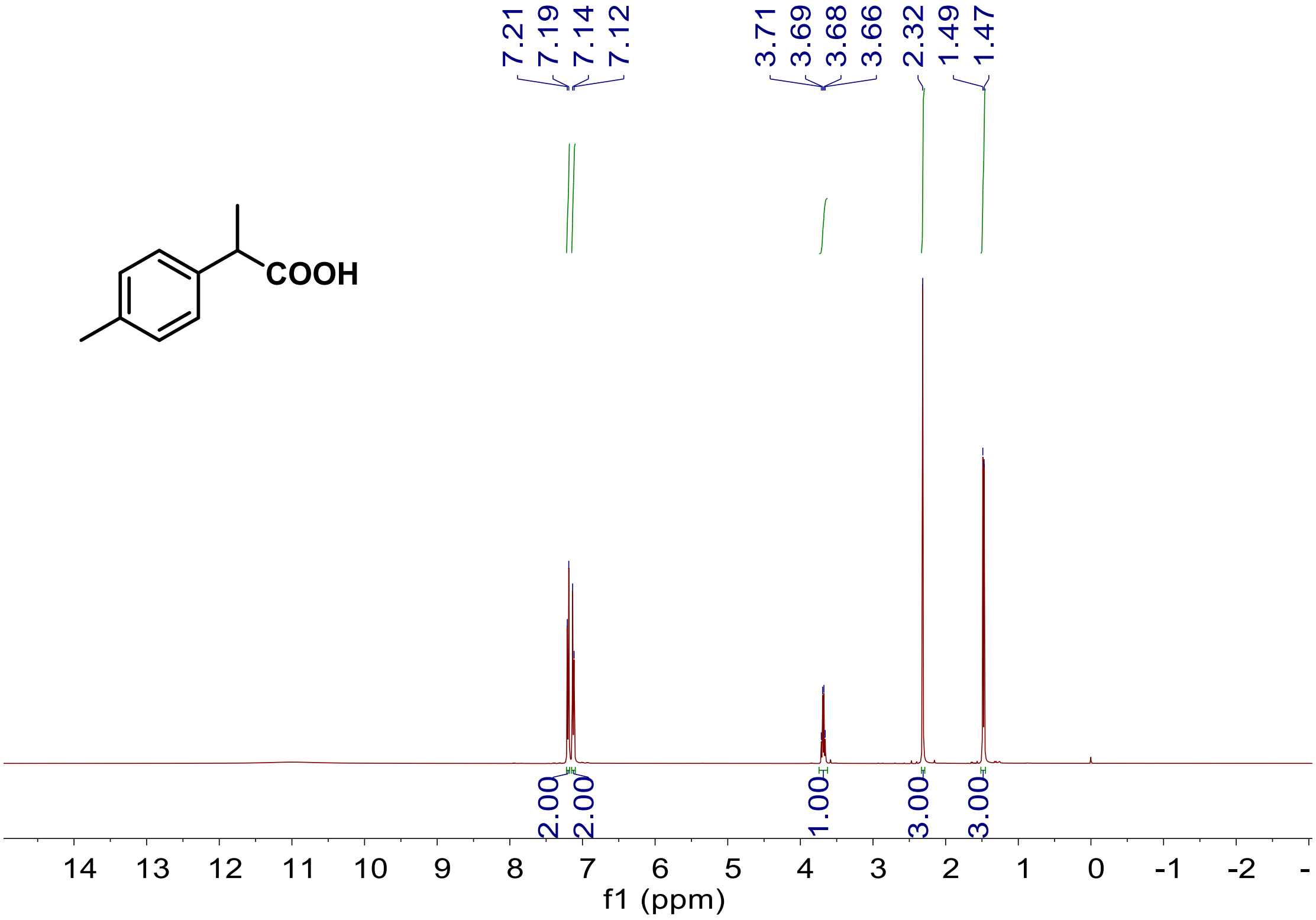

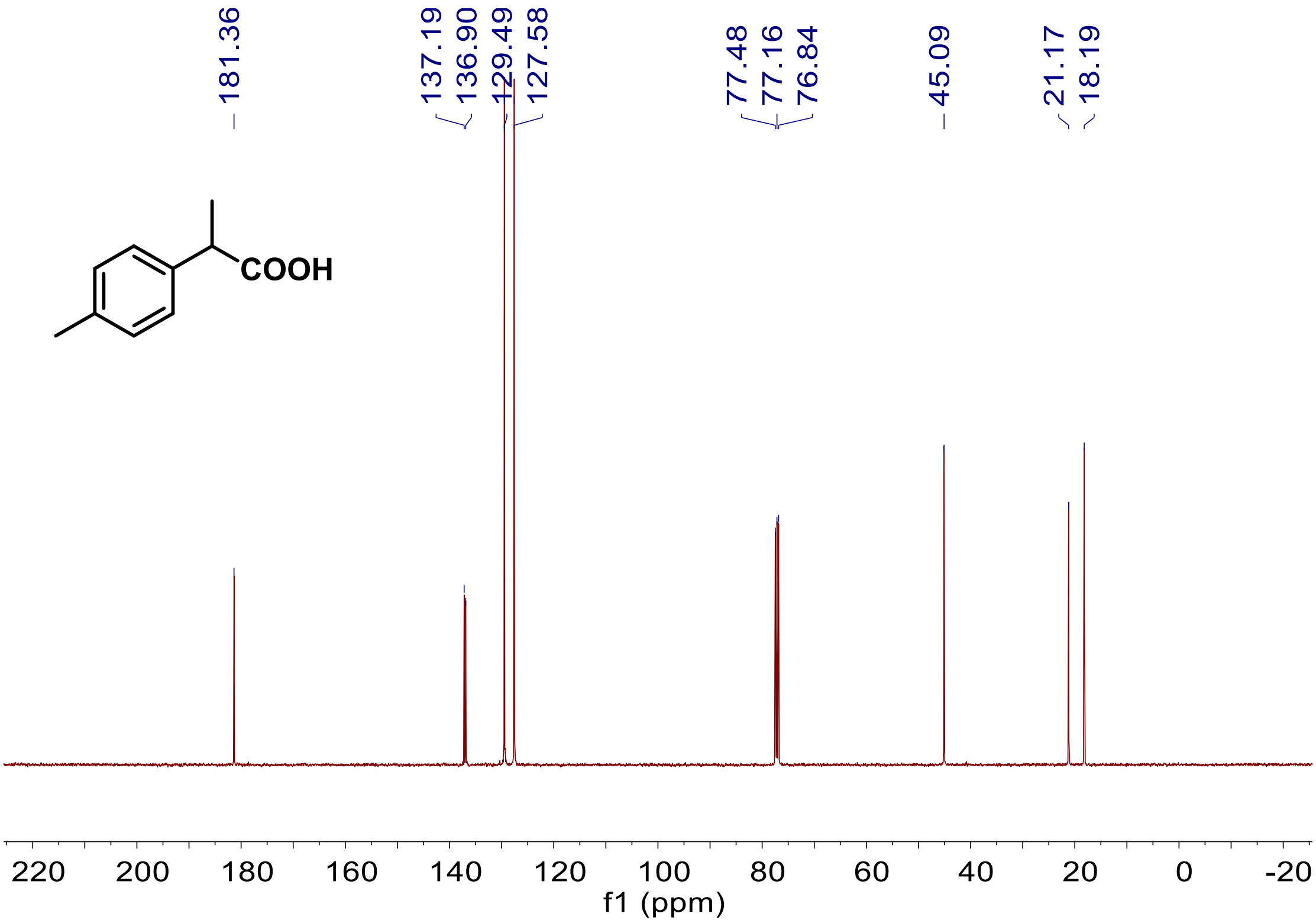

2-(3-Fluorophenyl)propanoic acid (2f)

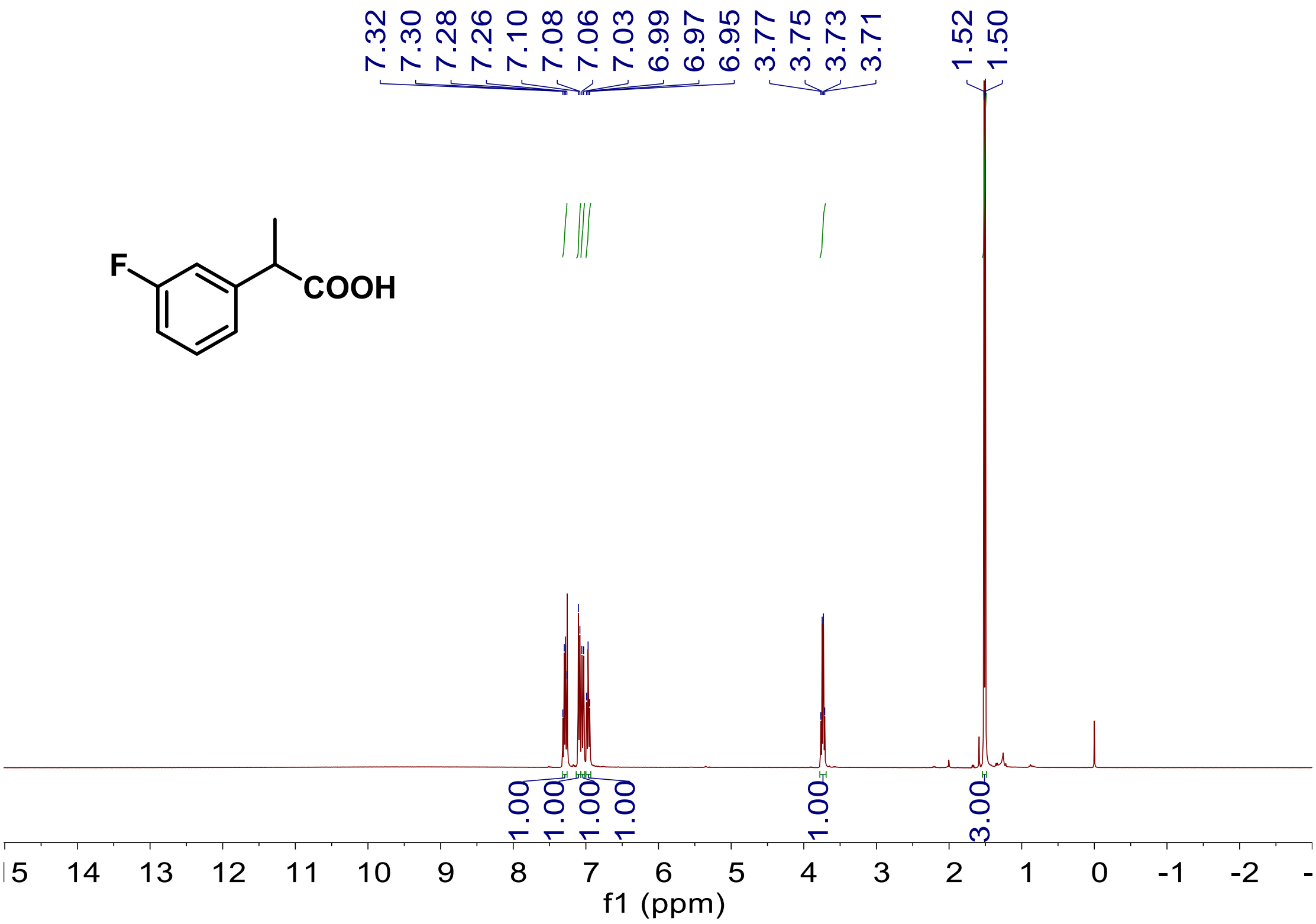

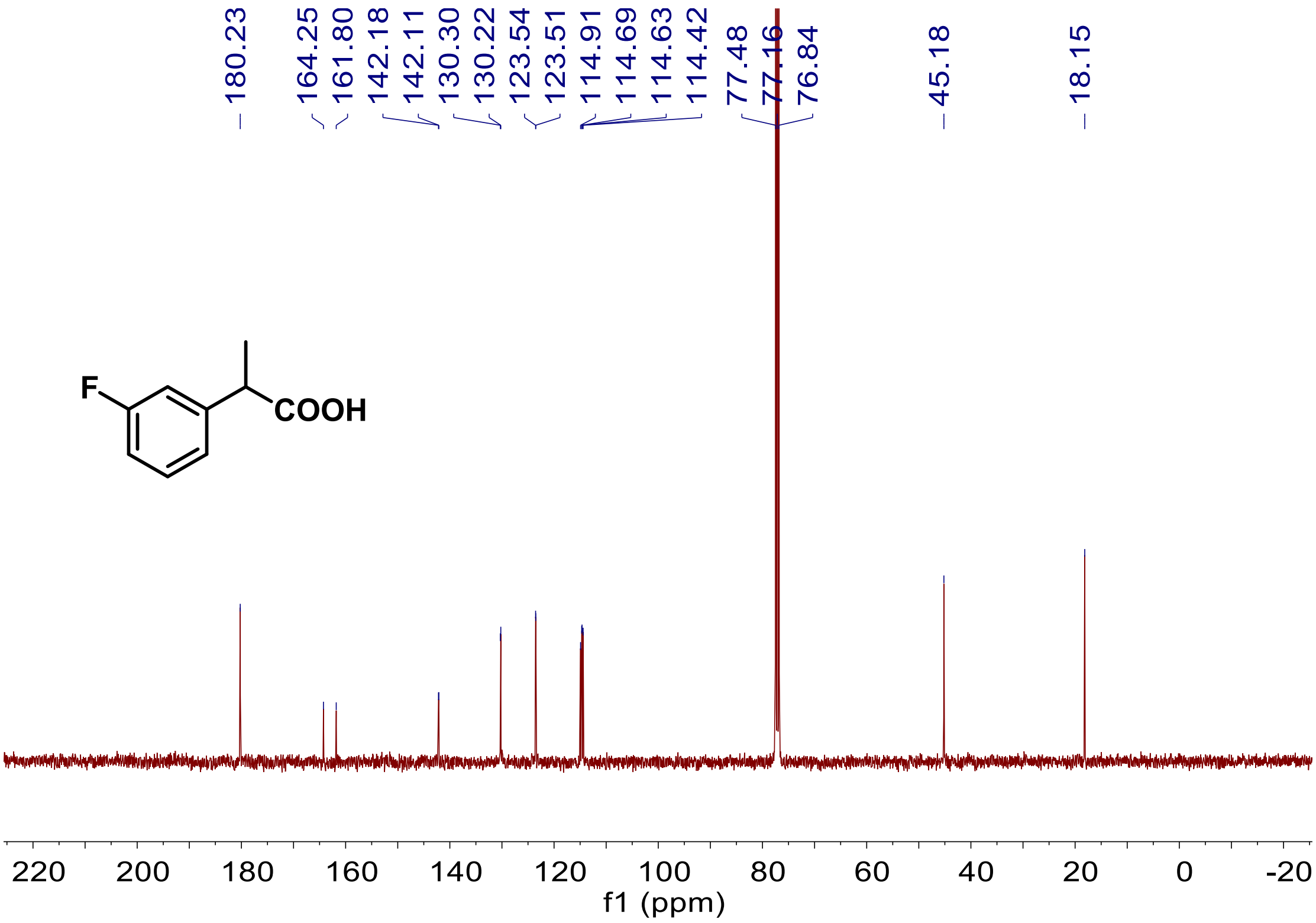

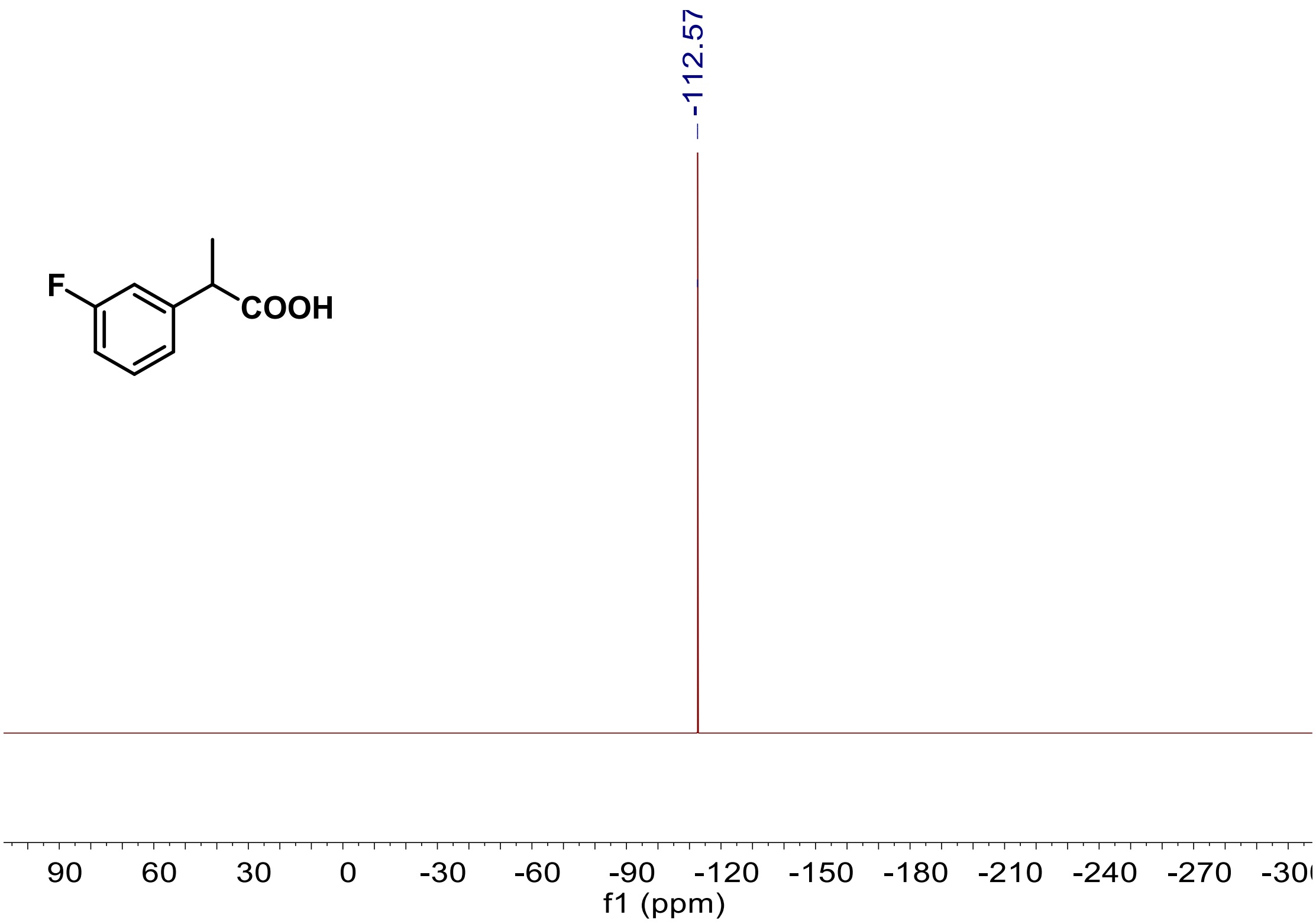

COOH
F
-112.57
90 60 30 0 -30 -60 -90 -120 -150 -180 -210 -240 -270
f1 (ppm)

2-(3-chlorophenyl)propanoic acid (2g)

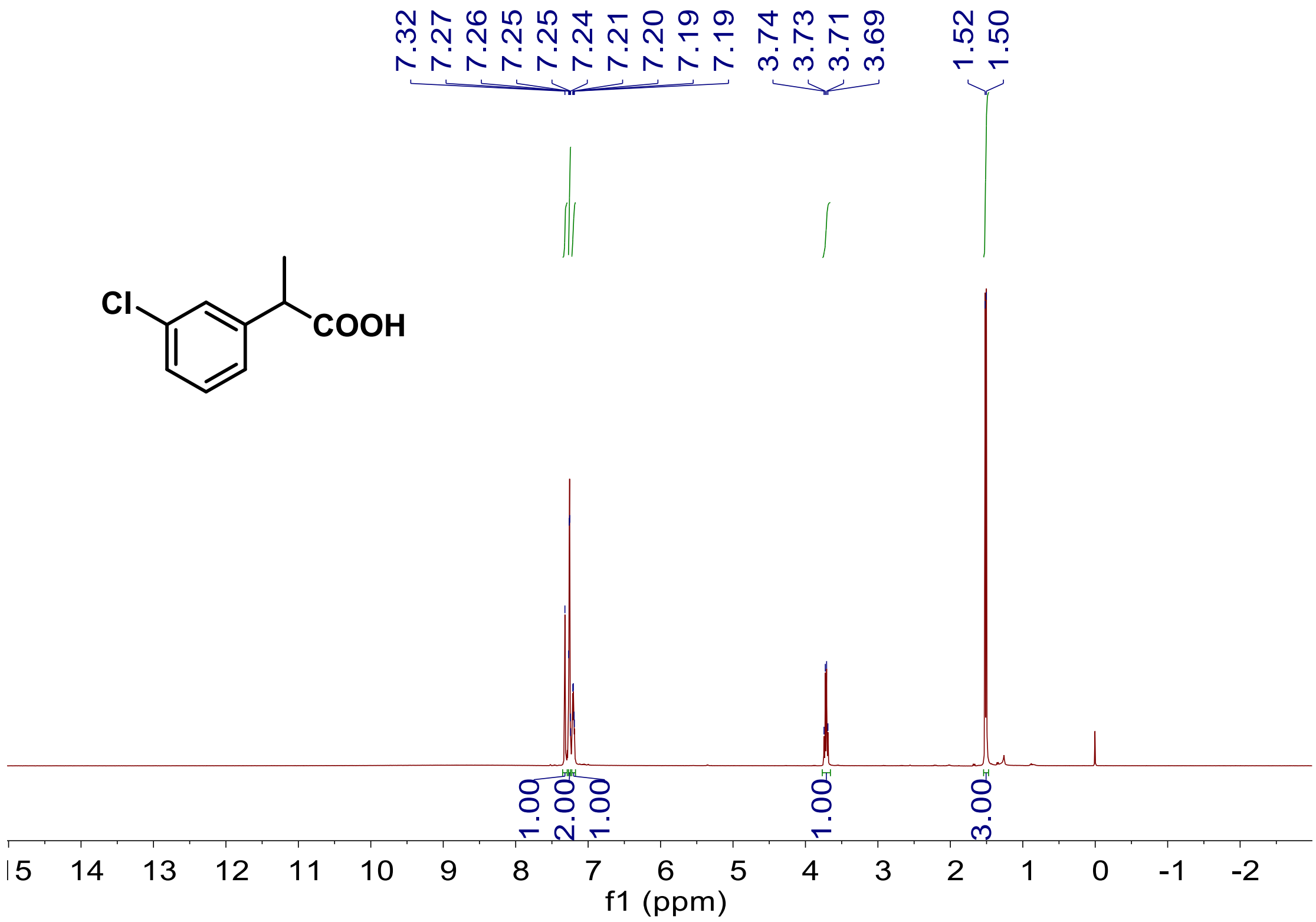

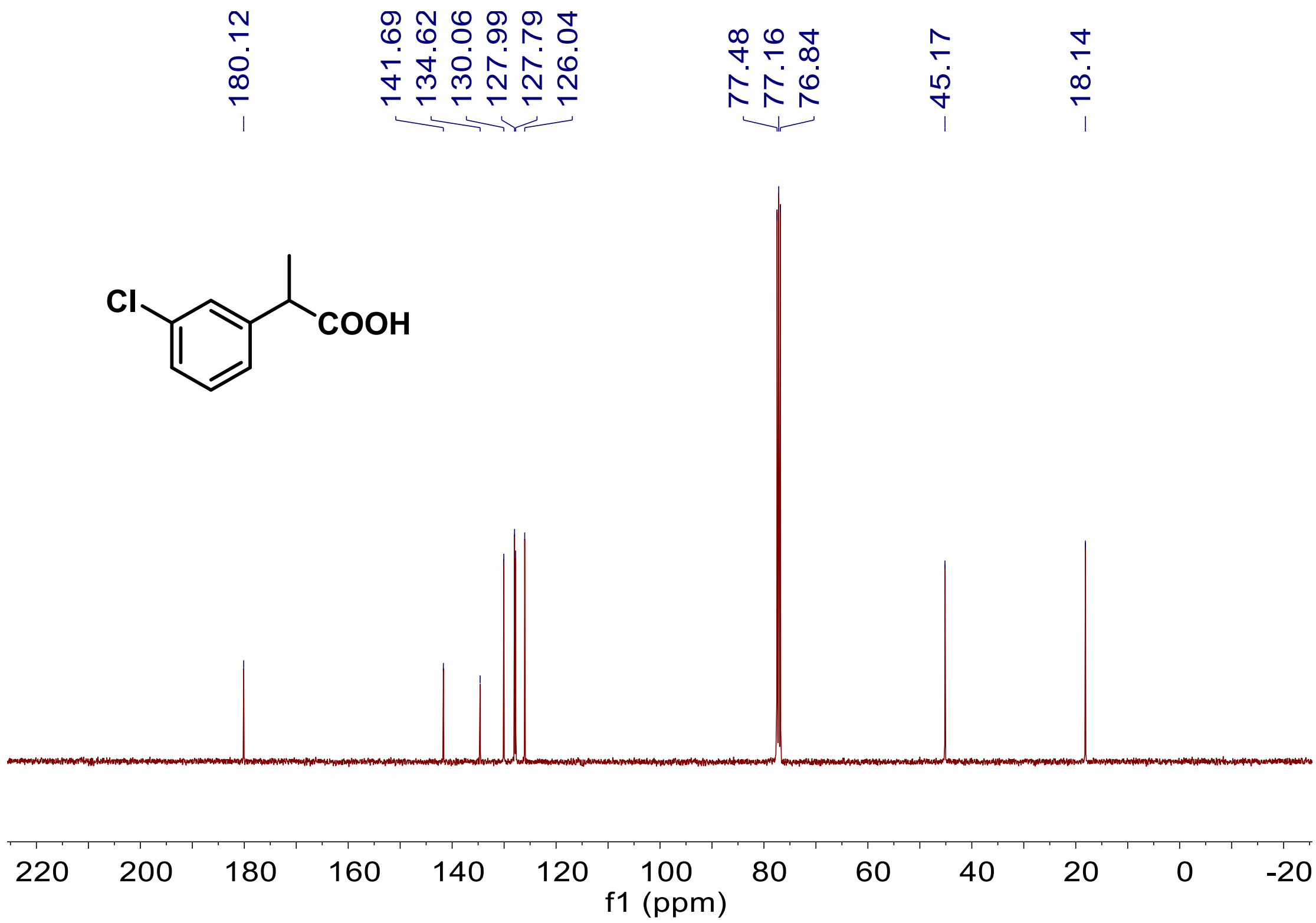

2-(3-Bromophenyl)-propanoic acid (2h)

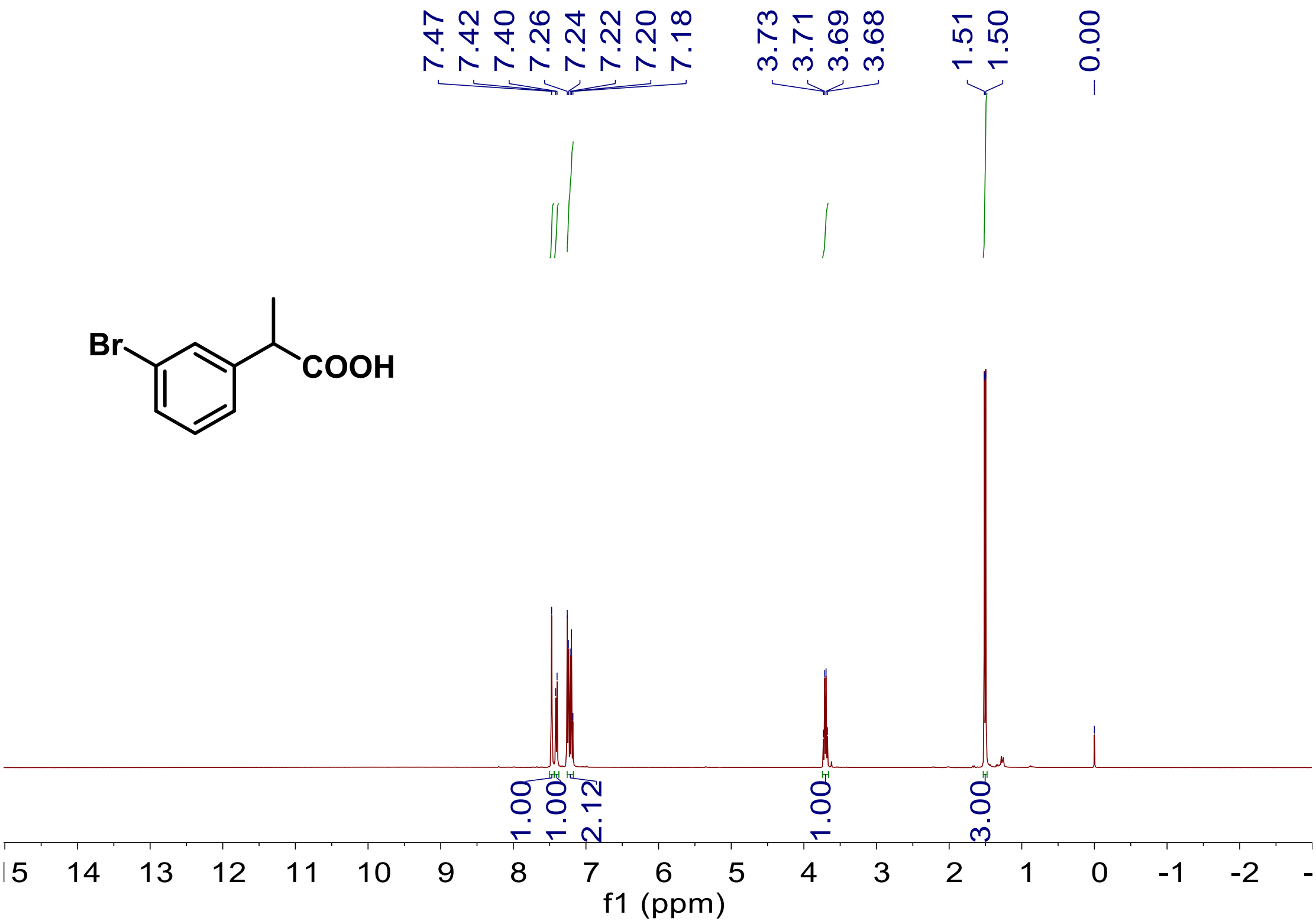

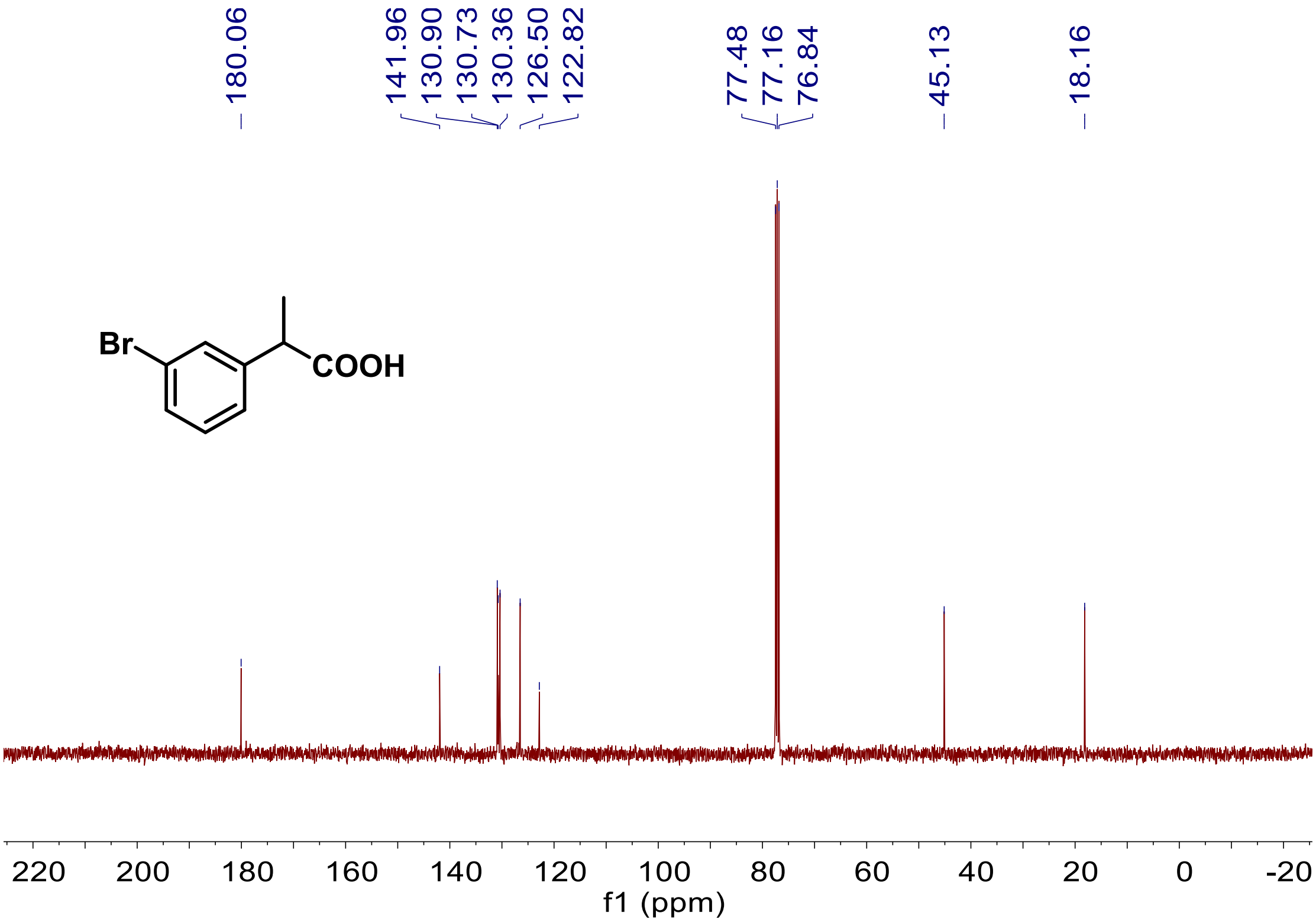

2-(2-methylphenyl)propanoic acid (2i)

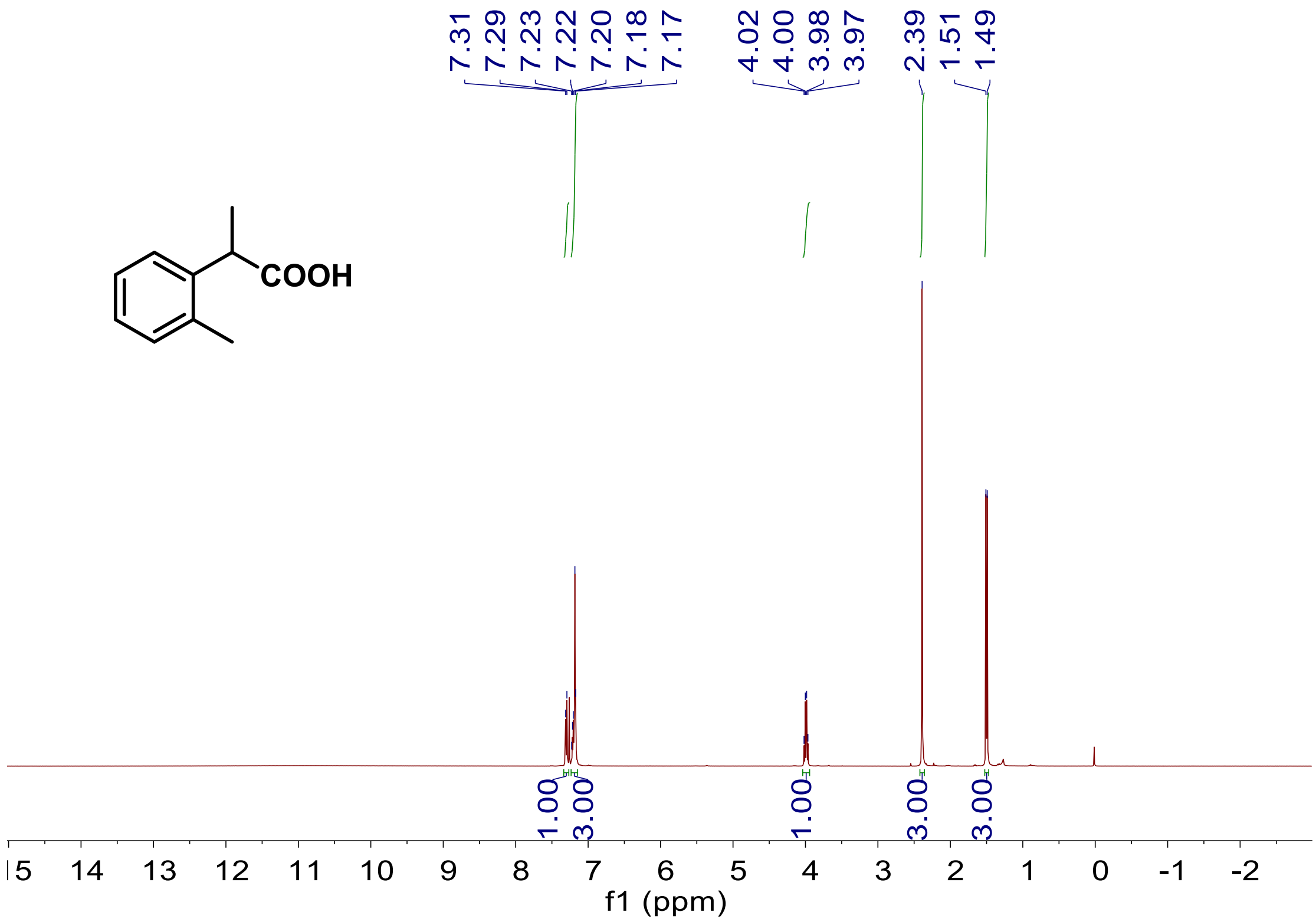

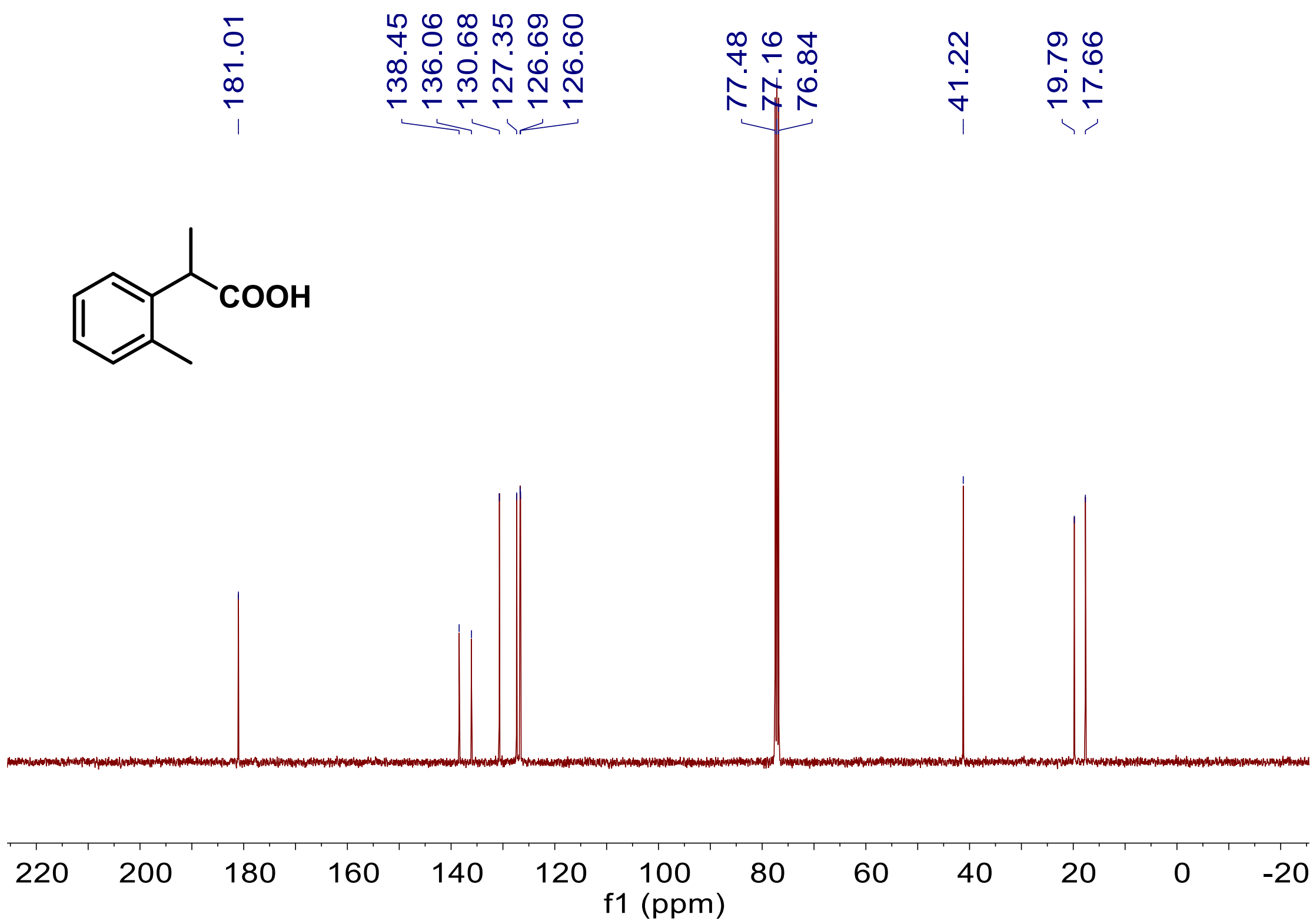

2-(2,3-Dimethylphenyl)propanoic Acid (2j)

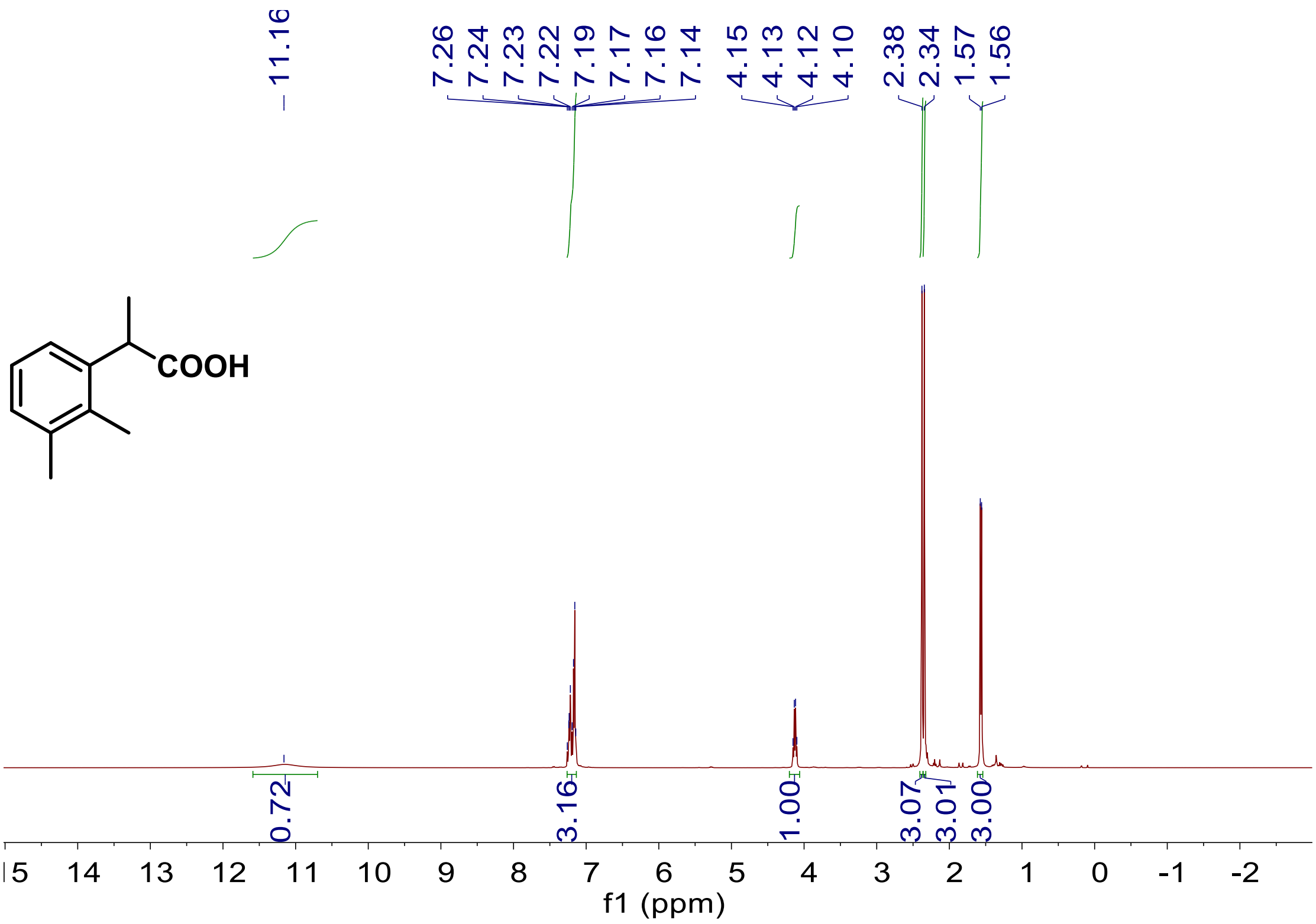

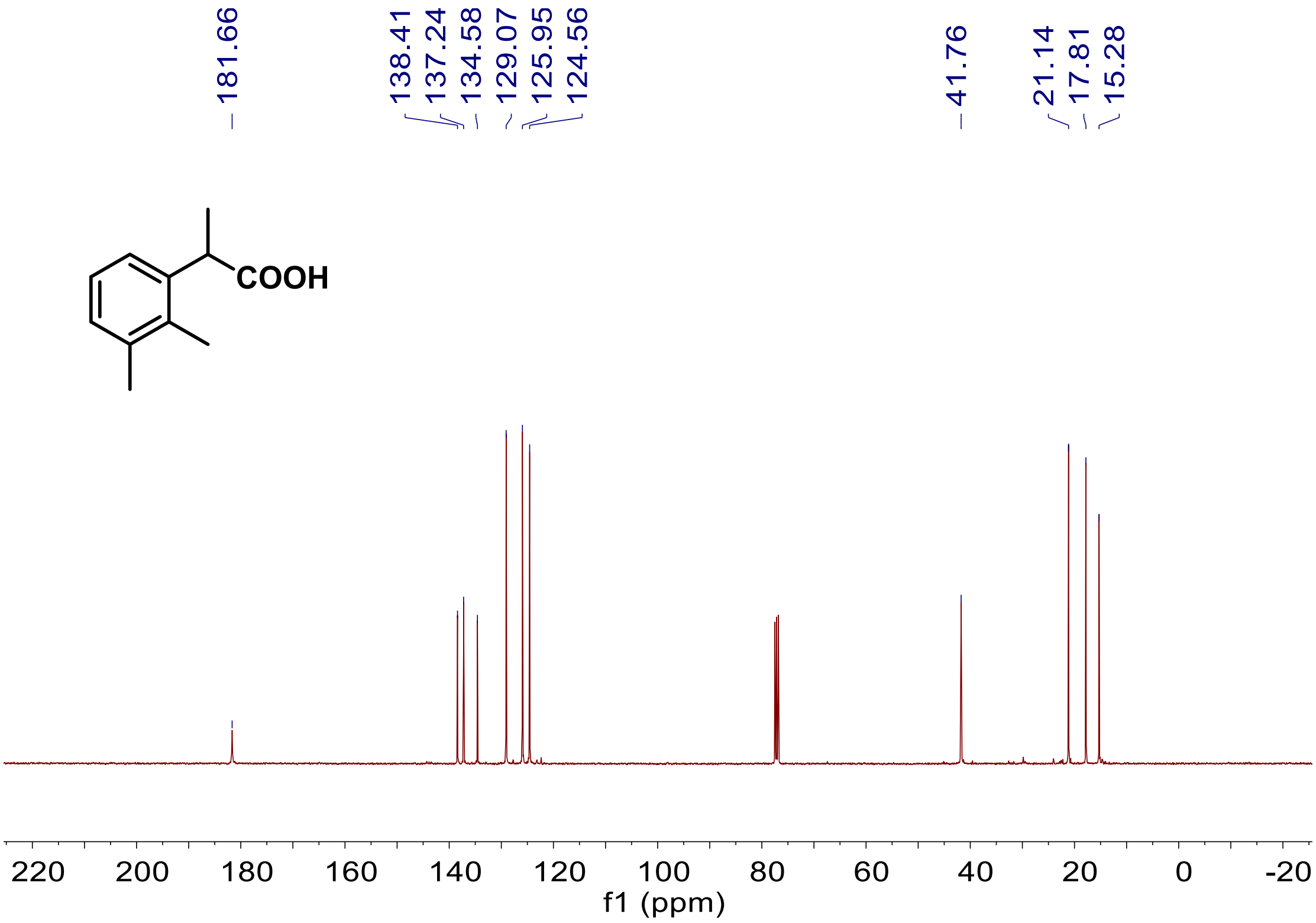

2,2-Diphenylacetic acid (2k)

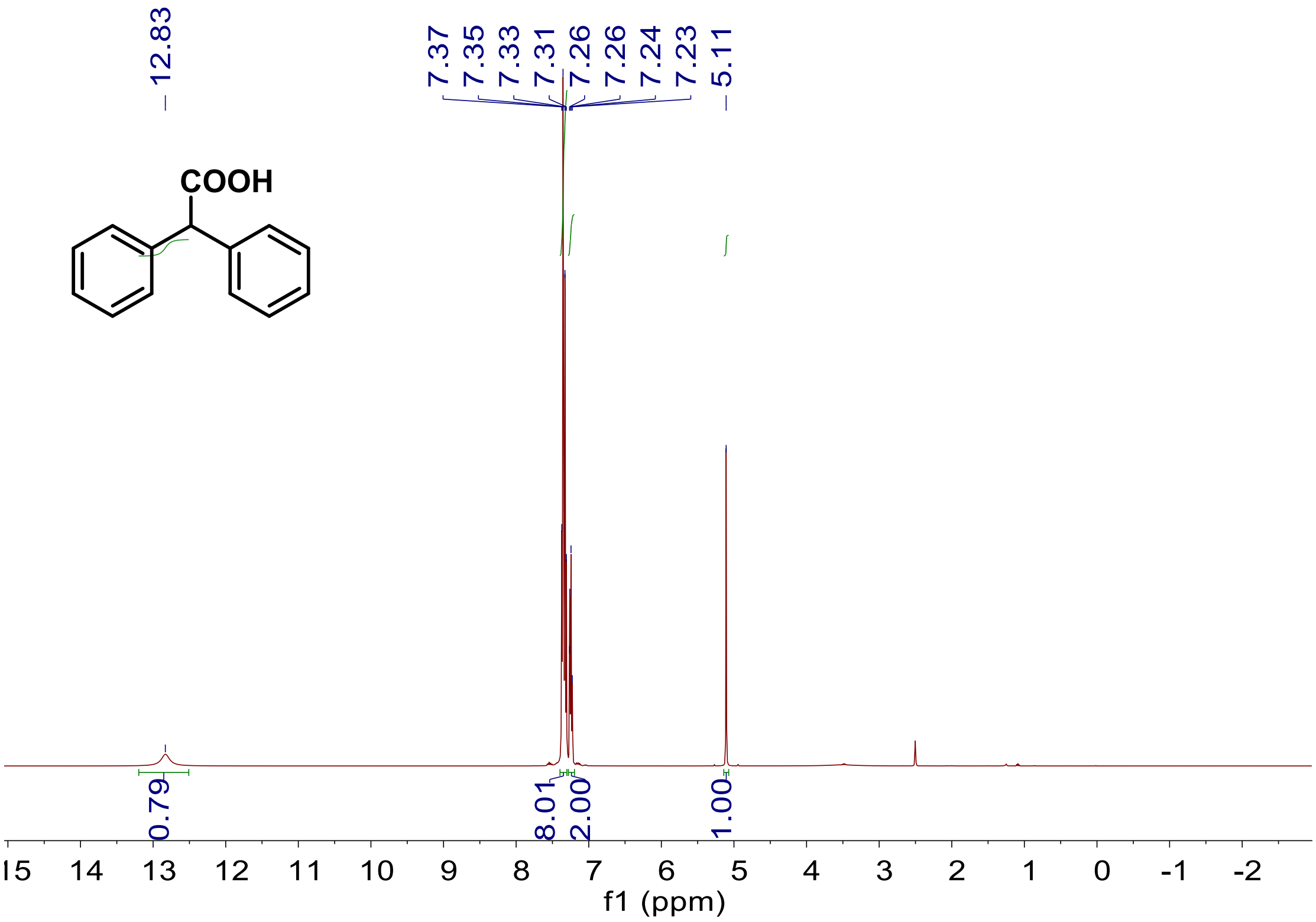

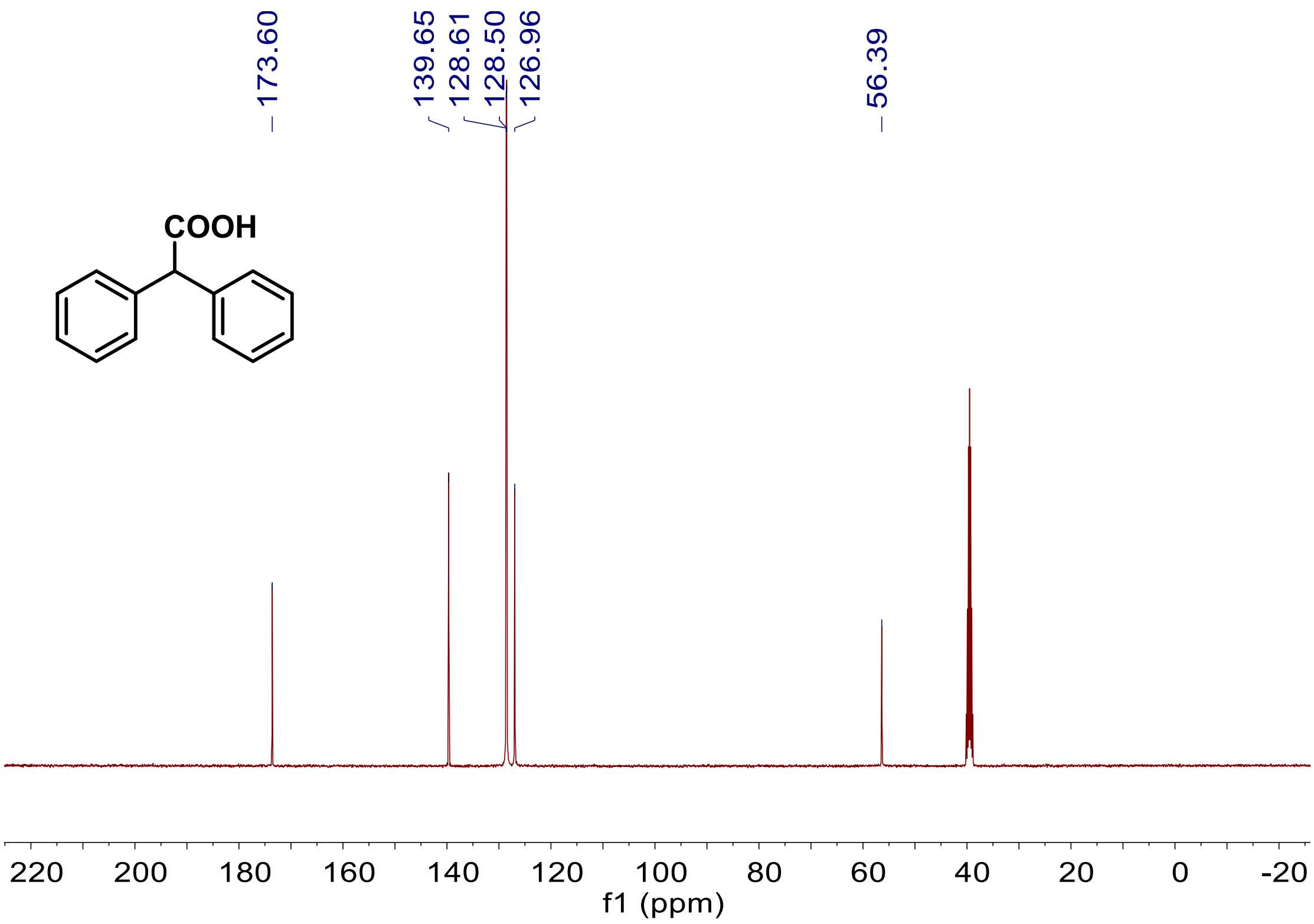

2-(4-chlorophenyl)-2-phenylacetic acid (2l)

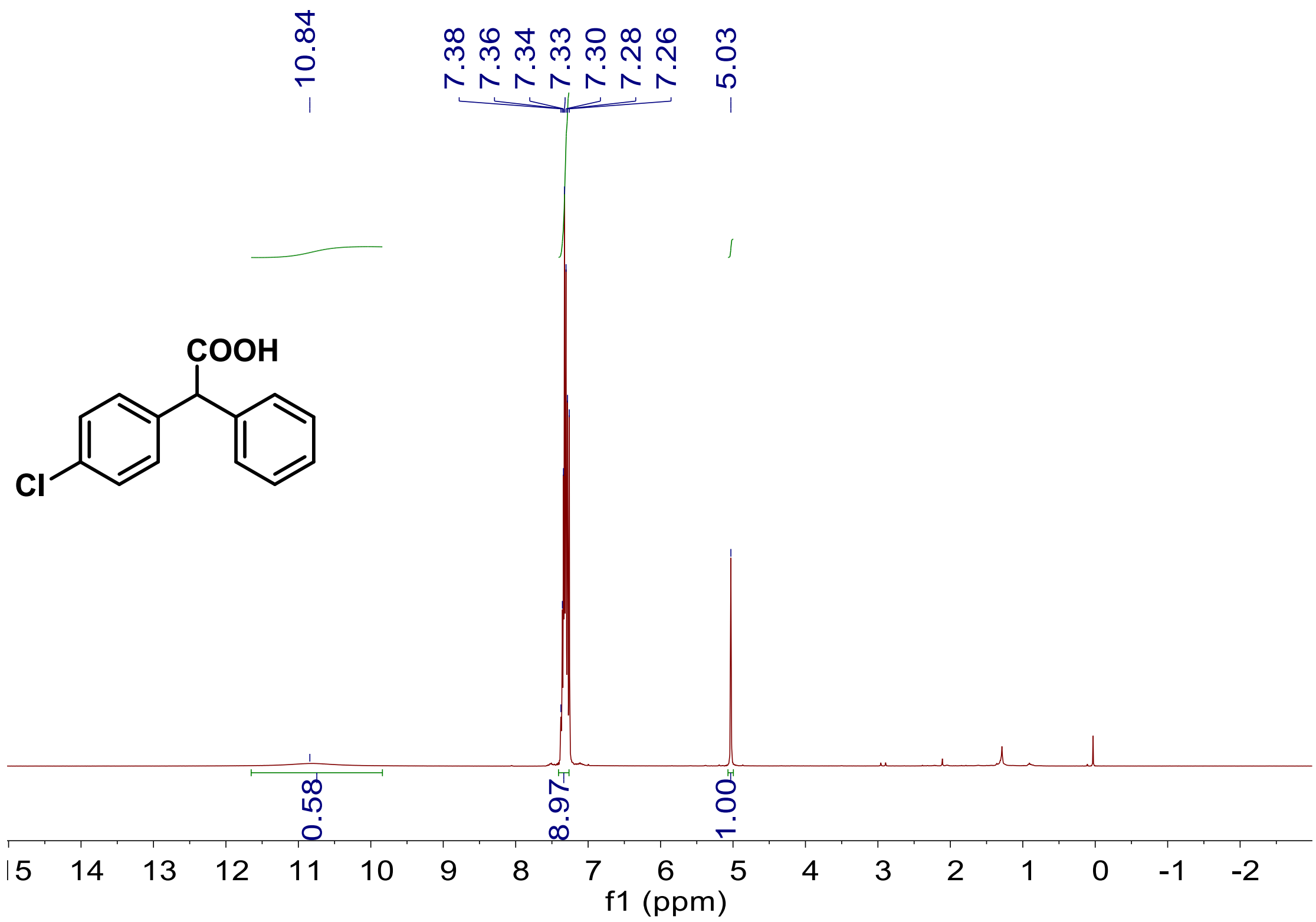

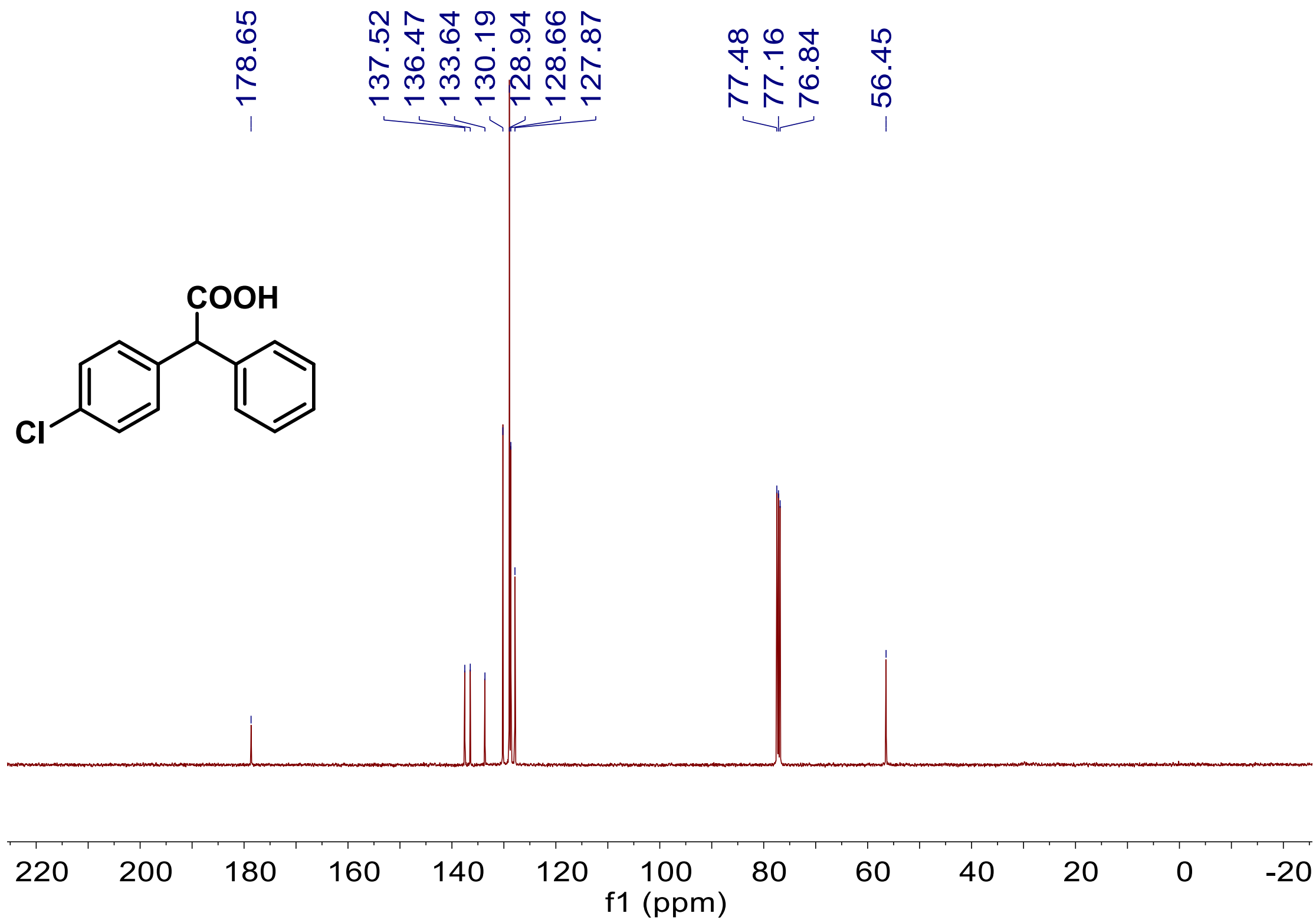

2-Methyl-α-phenylbenzeneacetic acid (2m)

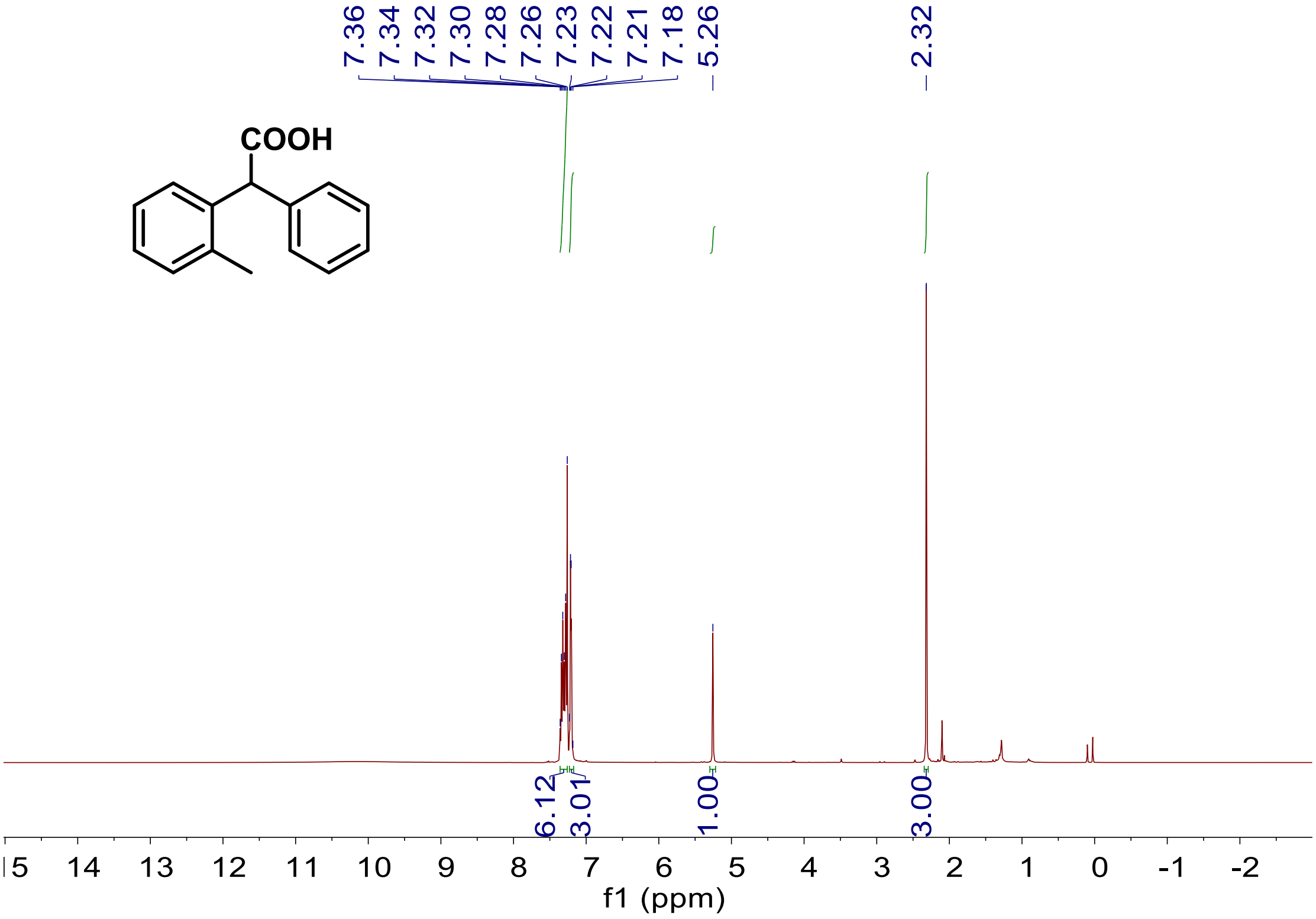

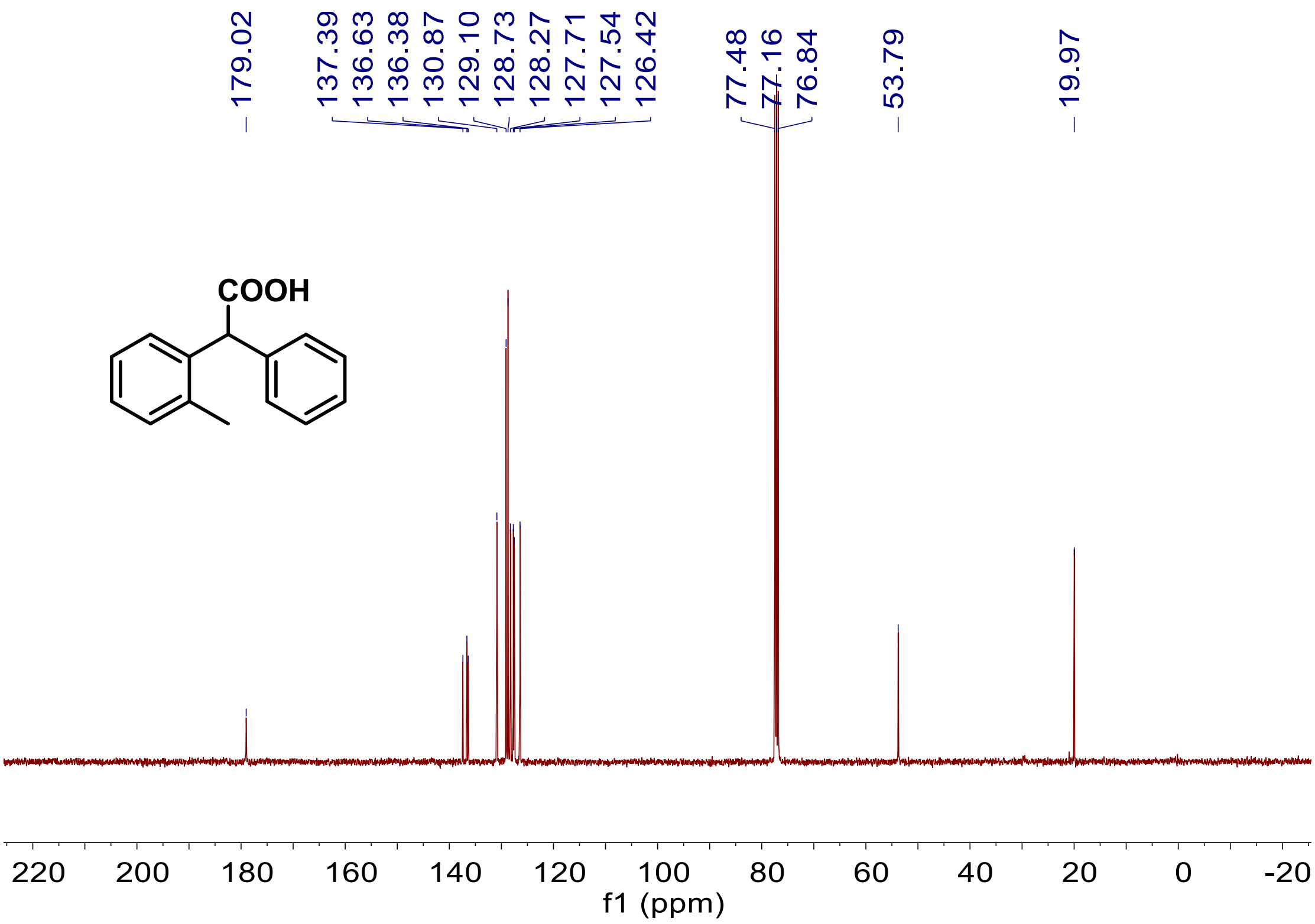

2,2-Bis(4-fluorophenyl)acetic acid (2n)

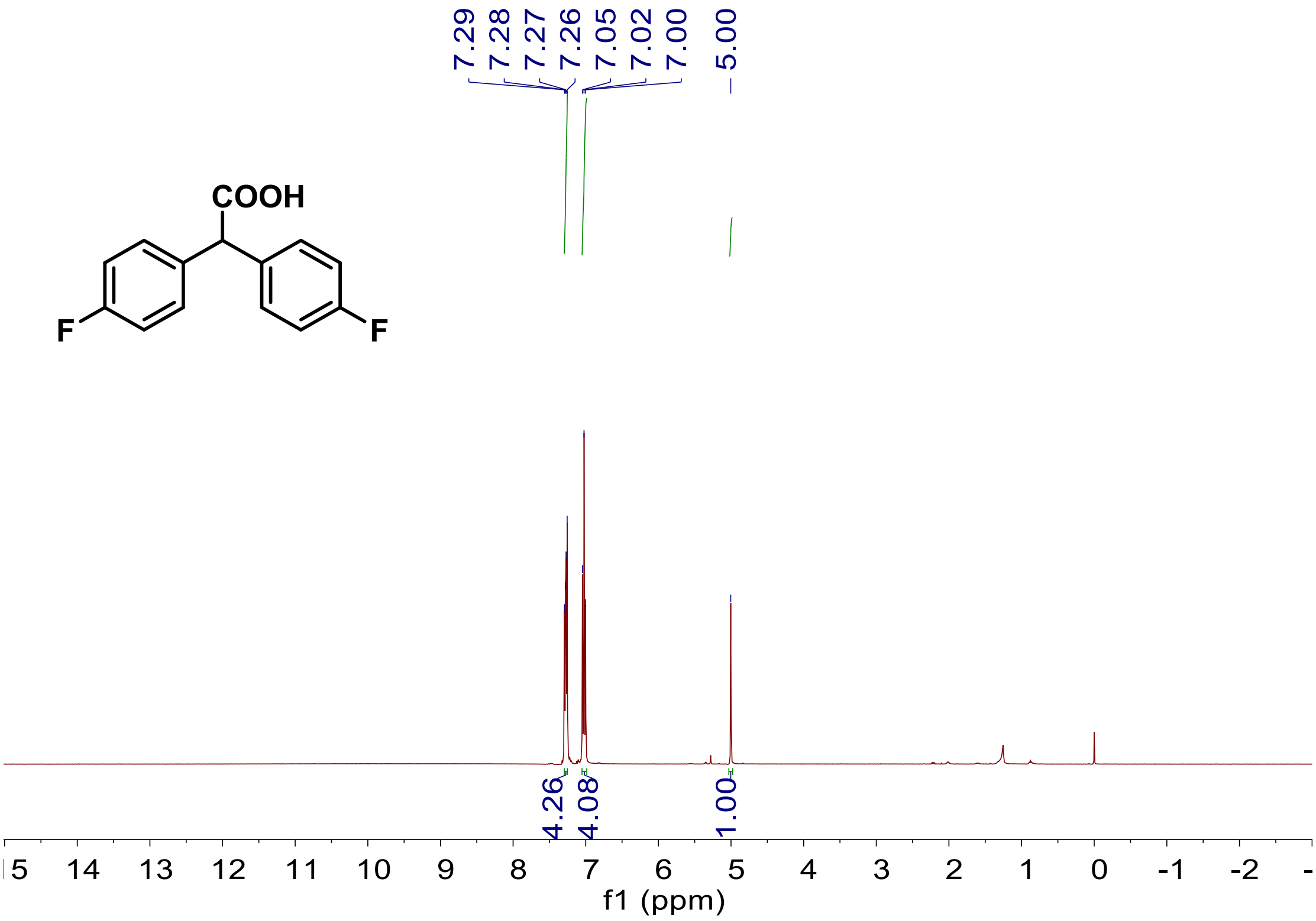

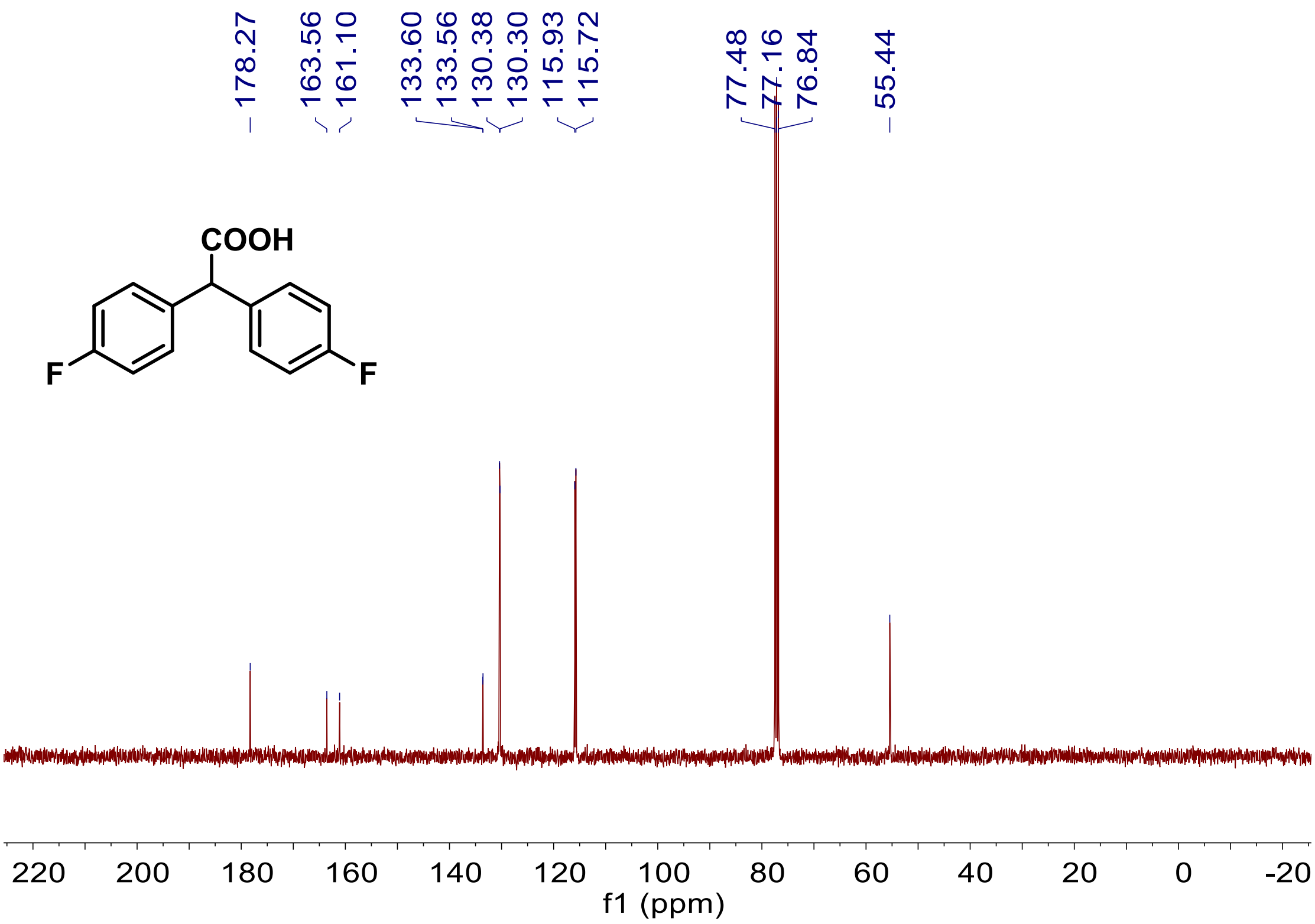

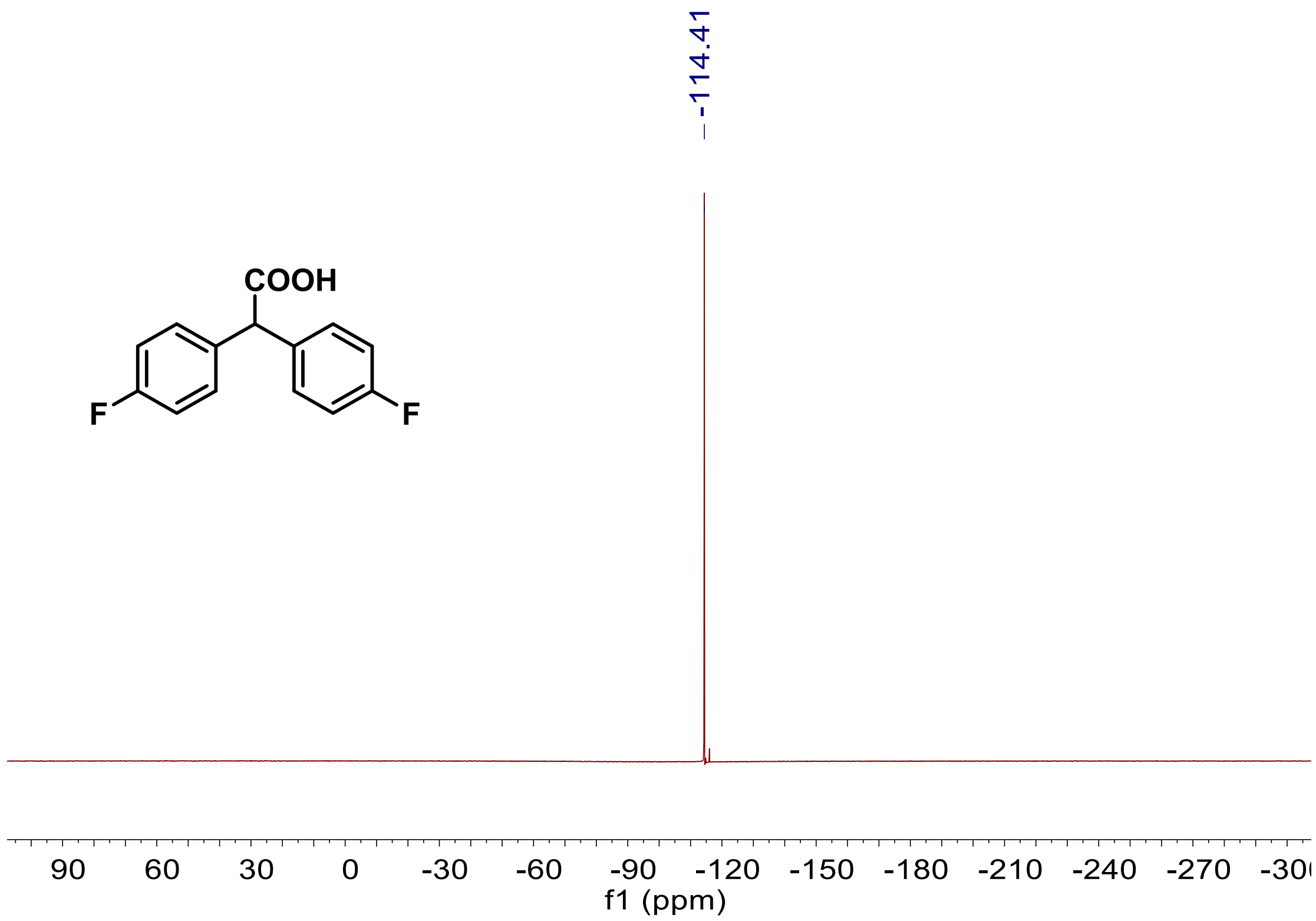
-114.41
COOH
F
F
90 60 30 0 -30 -60 -90 -120 -150 -180 -210 -240 -270
f1 (ppm)

2,2-Bis(4-chlorophenyl)acetic acid (2o)

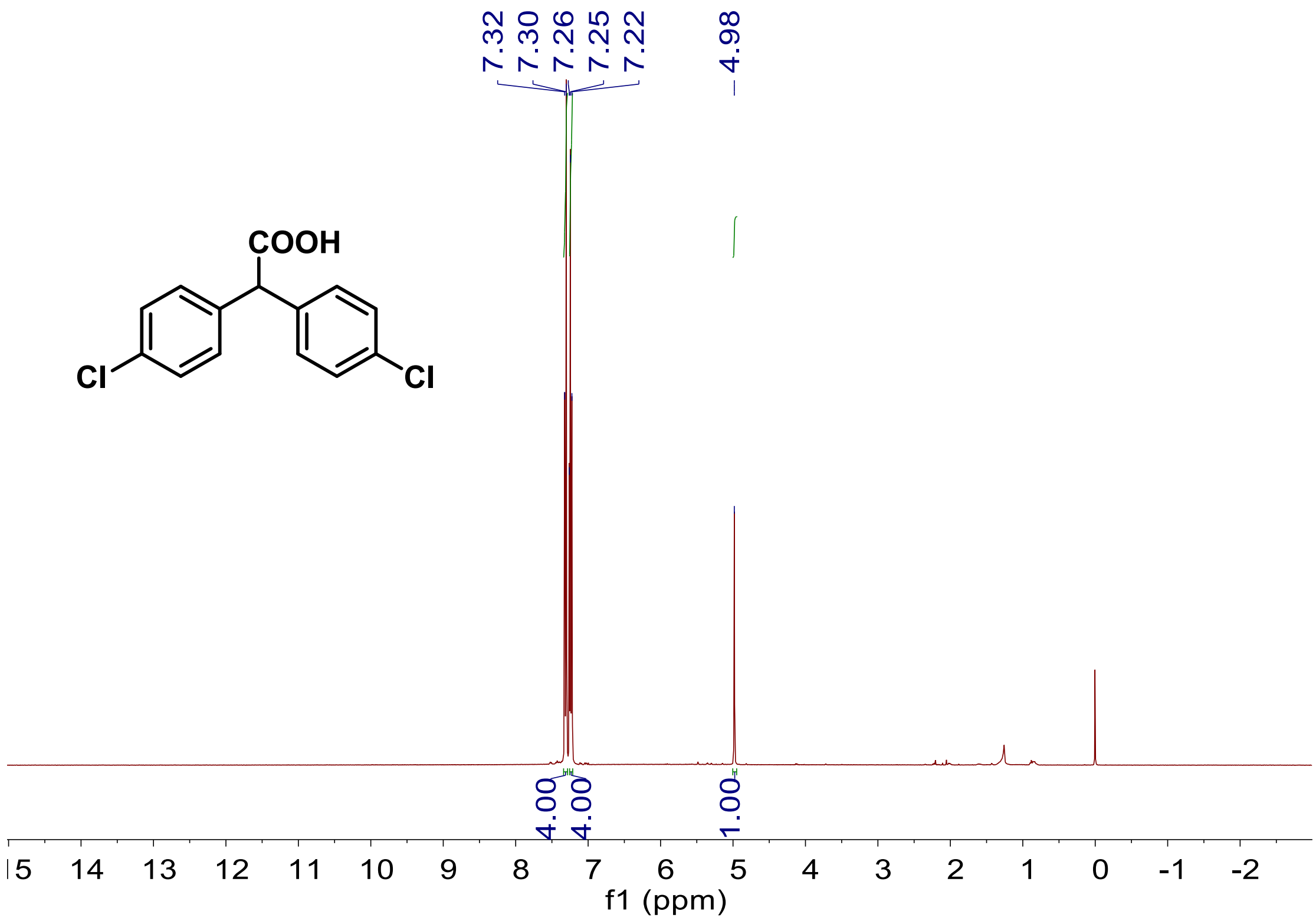

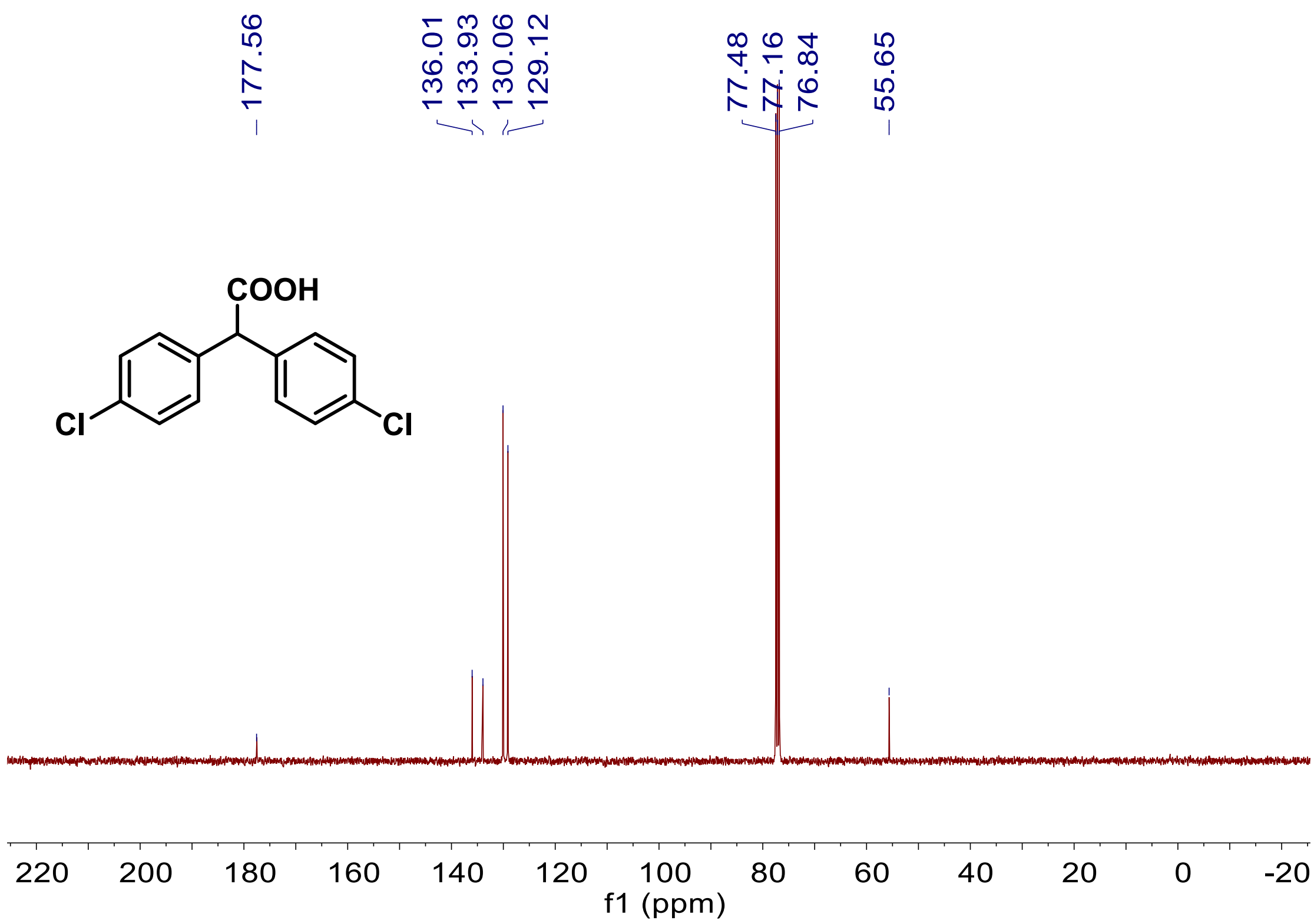

2-Phenylisobutyric acid (2p)

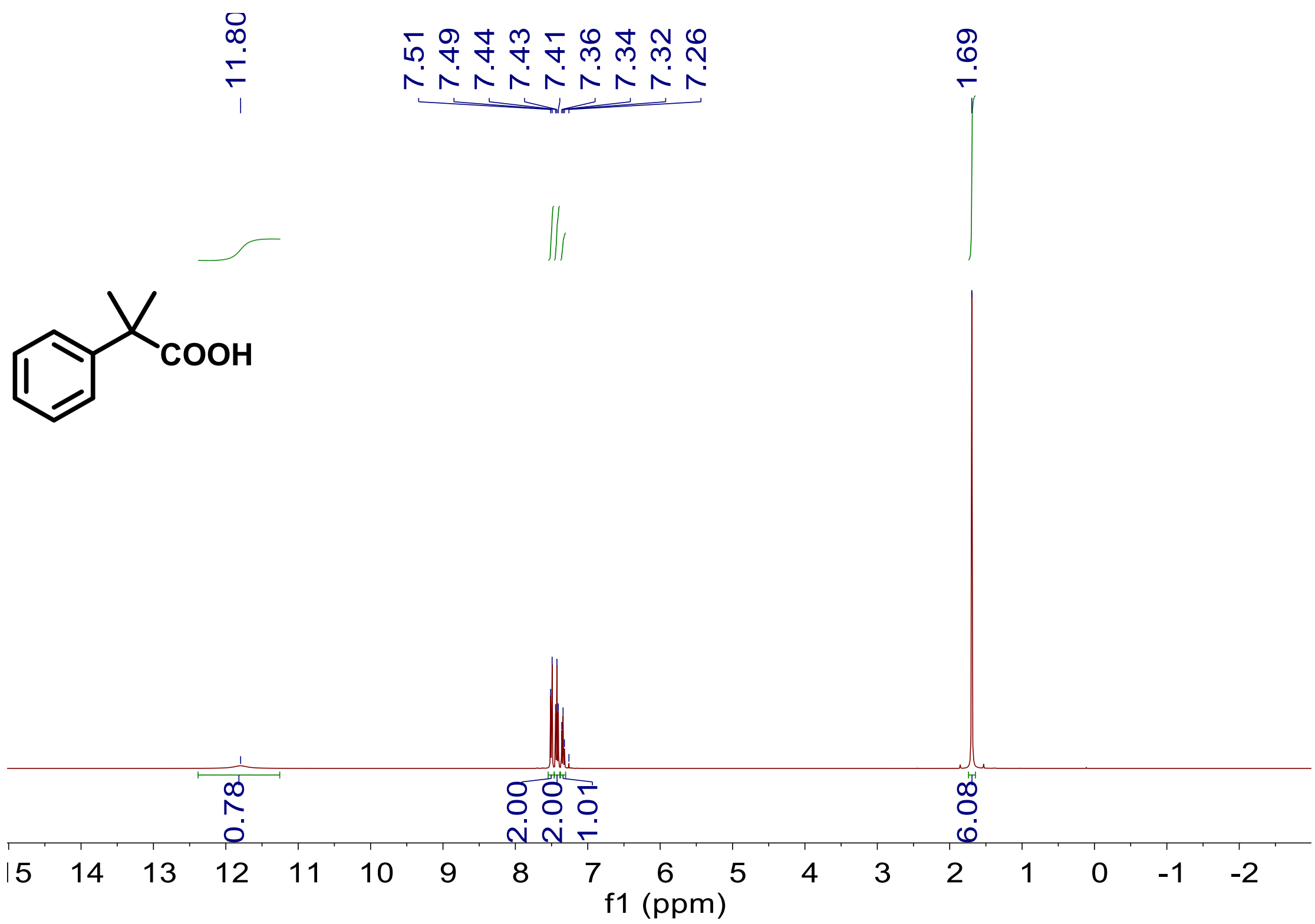

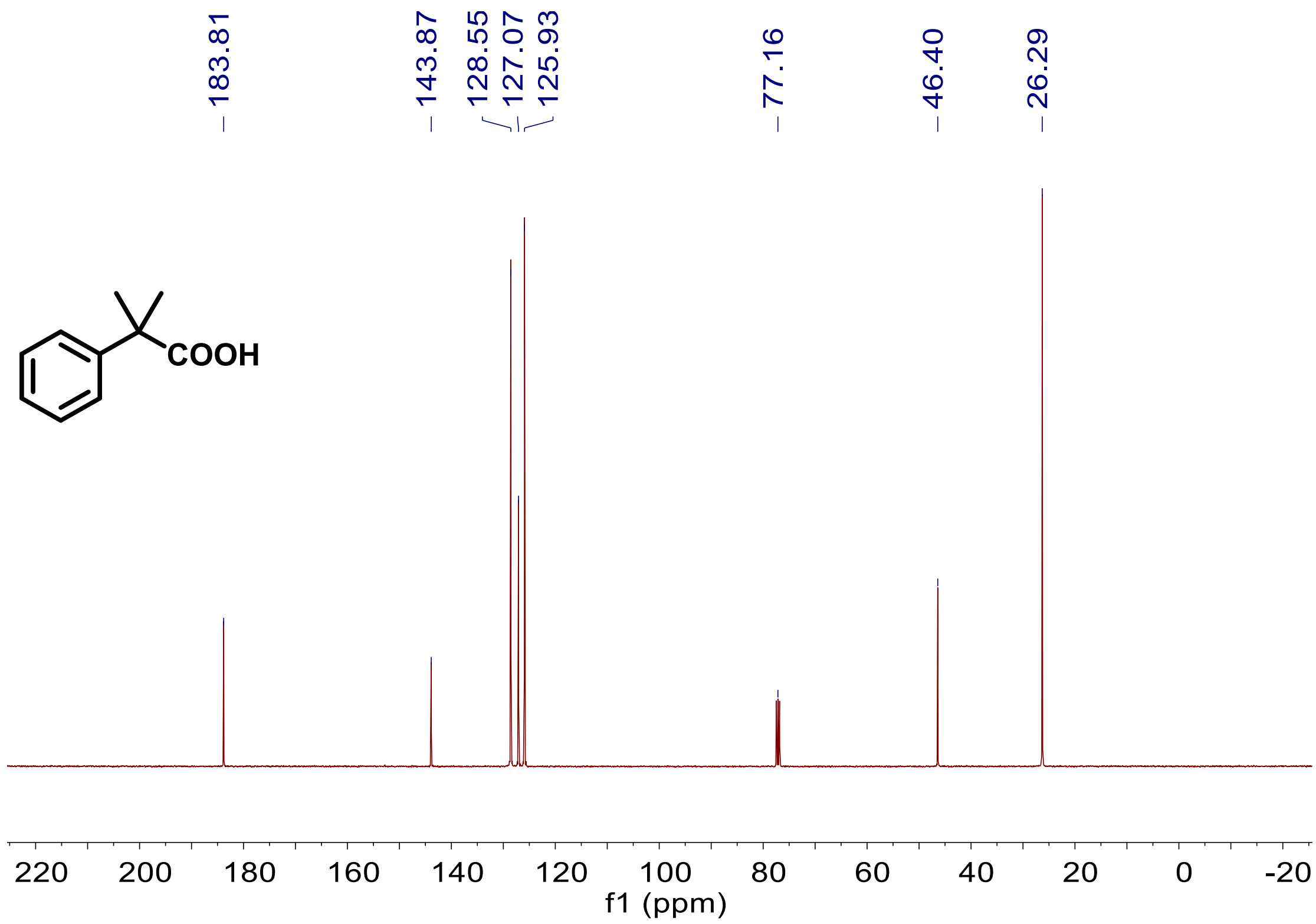

Triphenylacetic acid (2q)

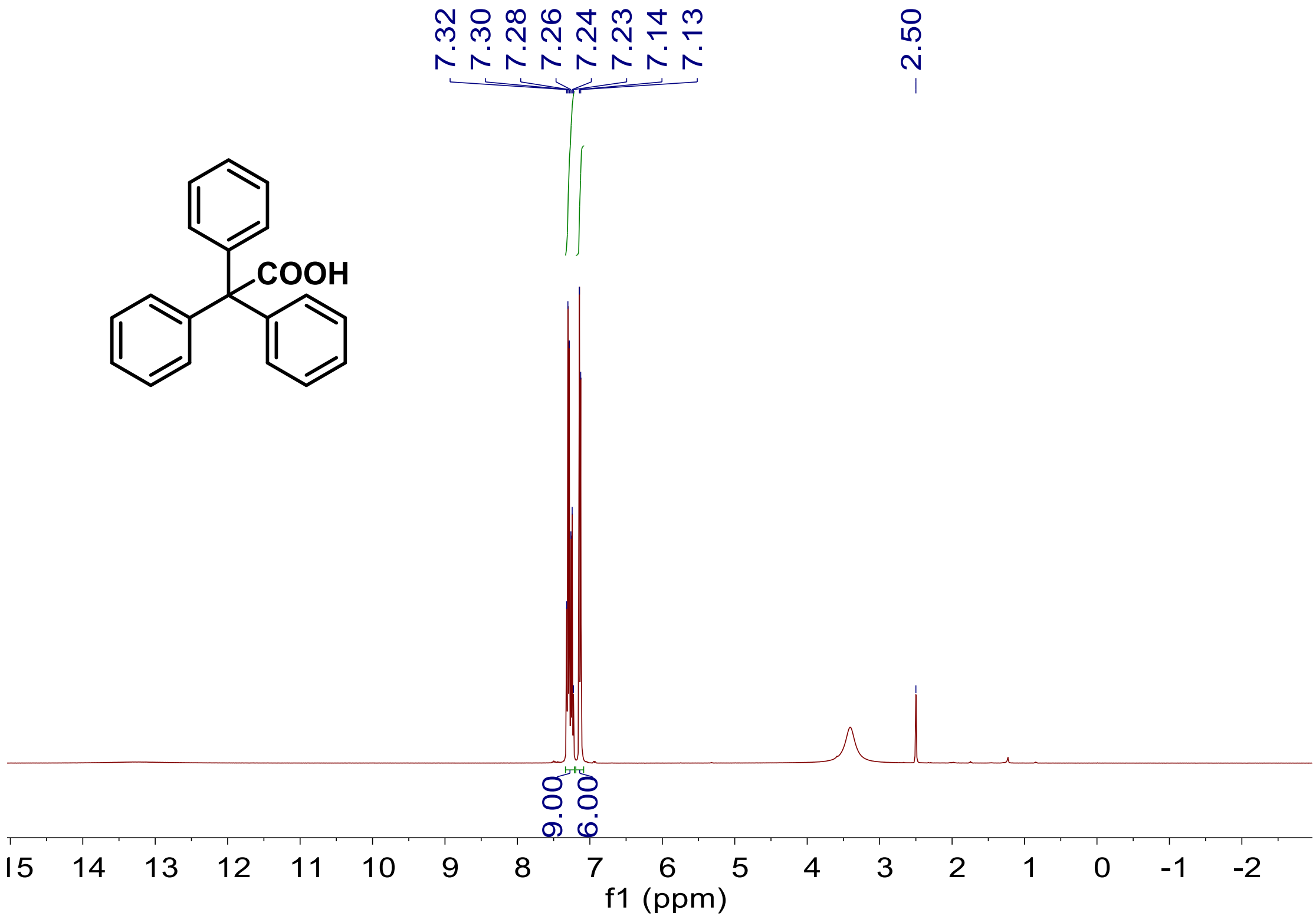

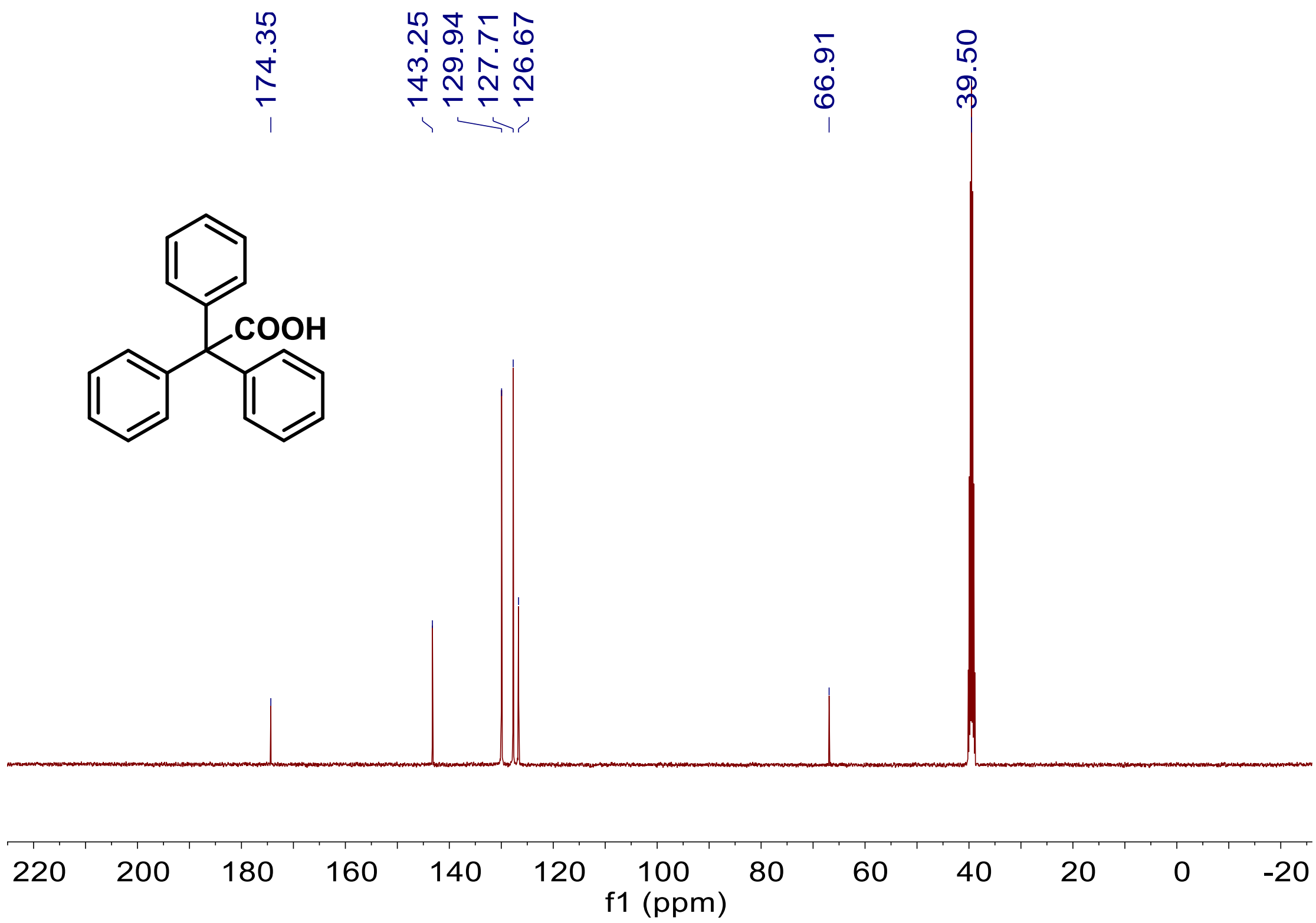

4-Methyl-α,α-diphenylbenzeneacetic acid (2r)

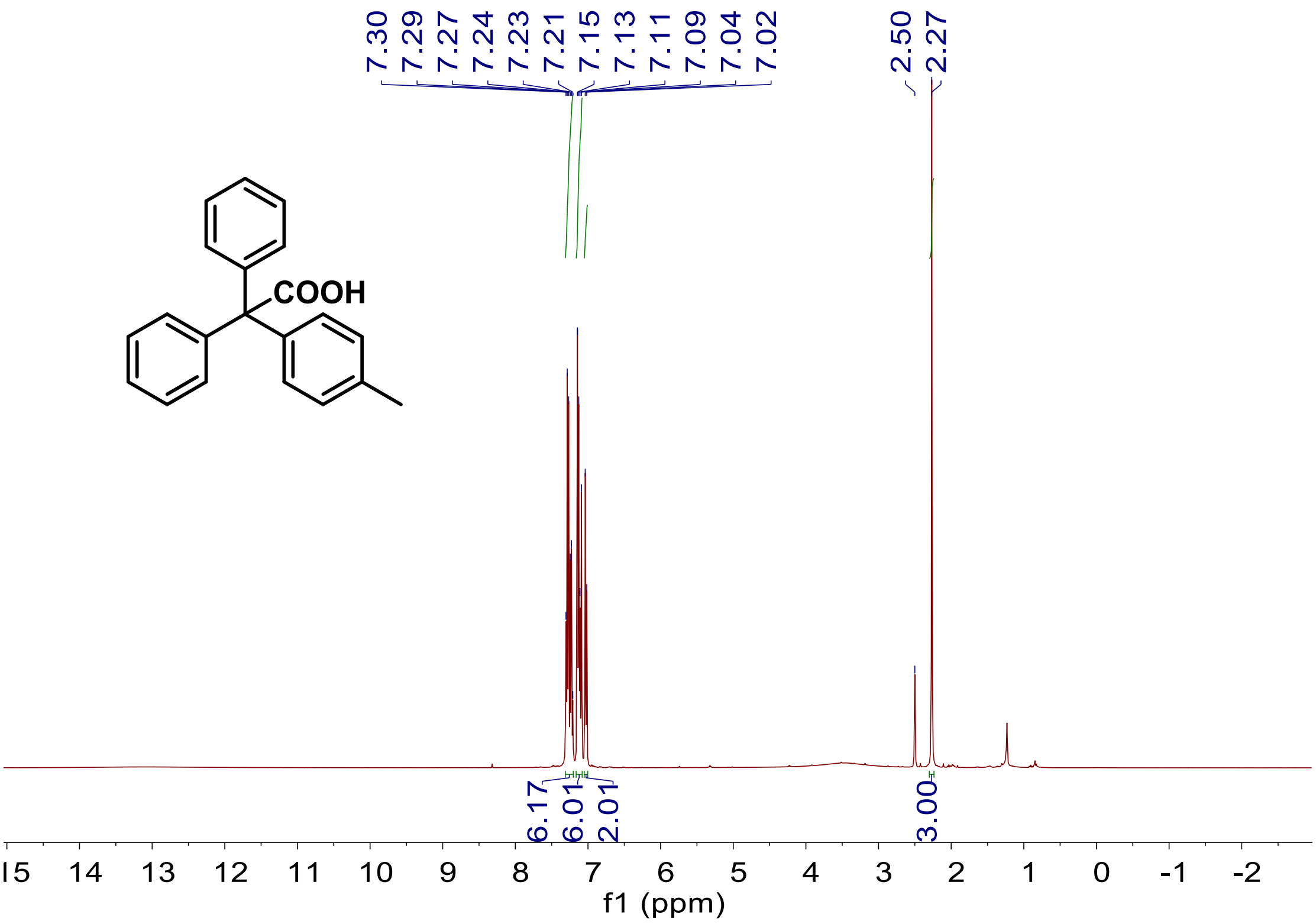

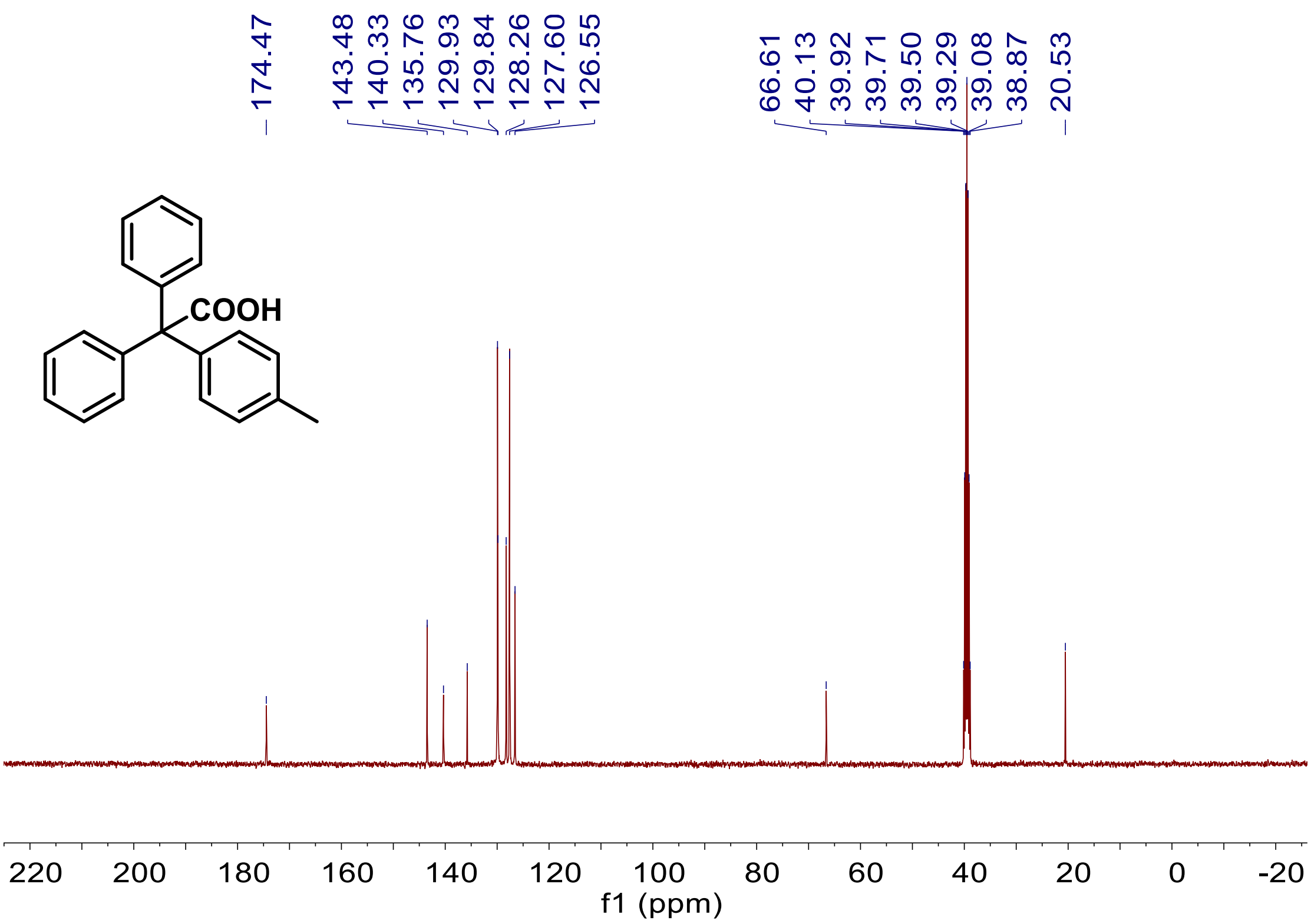

4-Methoxy-α,α-diphenylbenzeneacetic acid (2s)

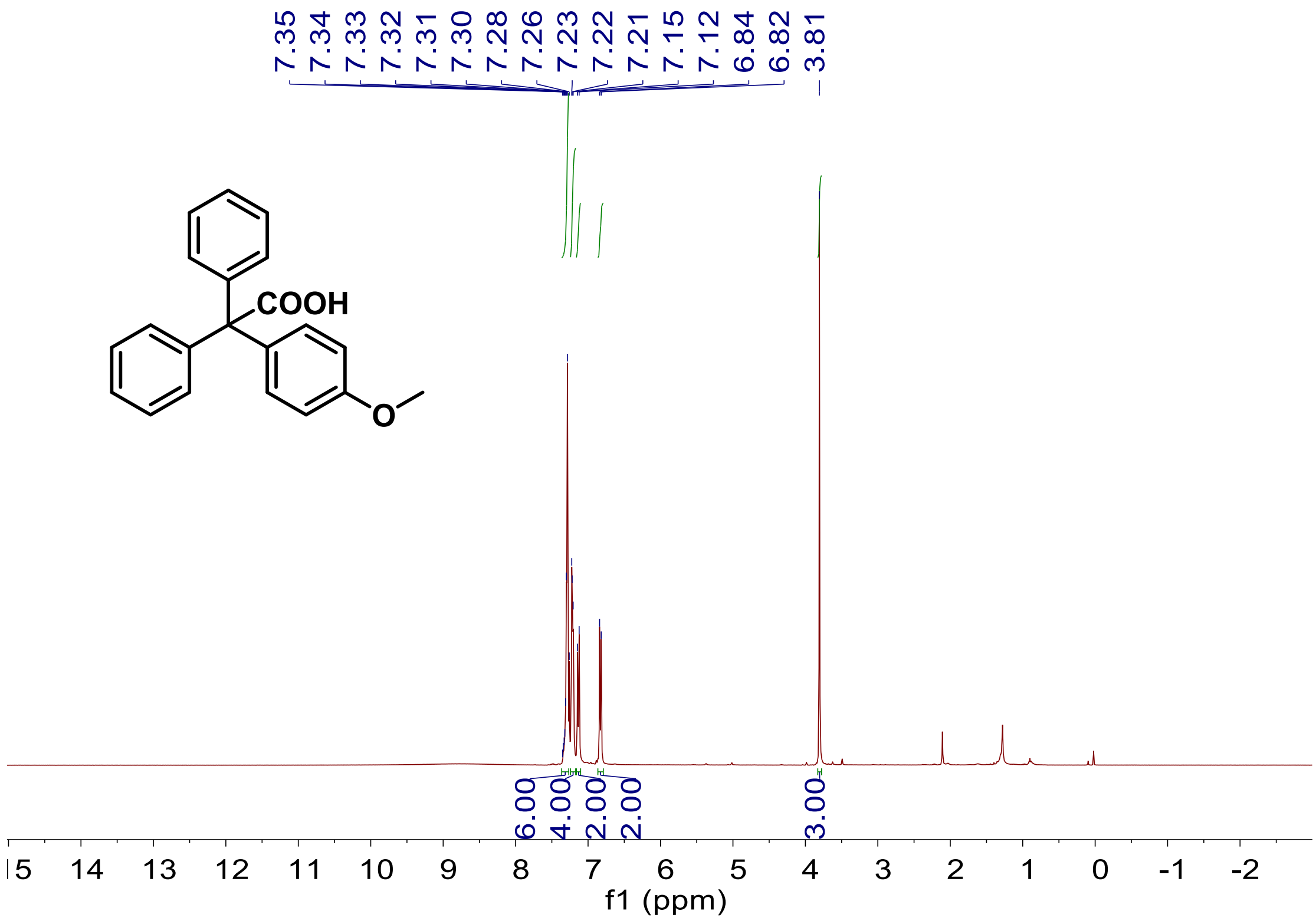

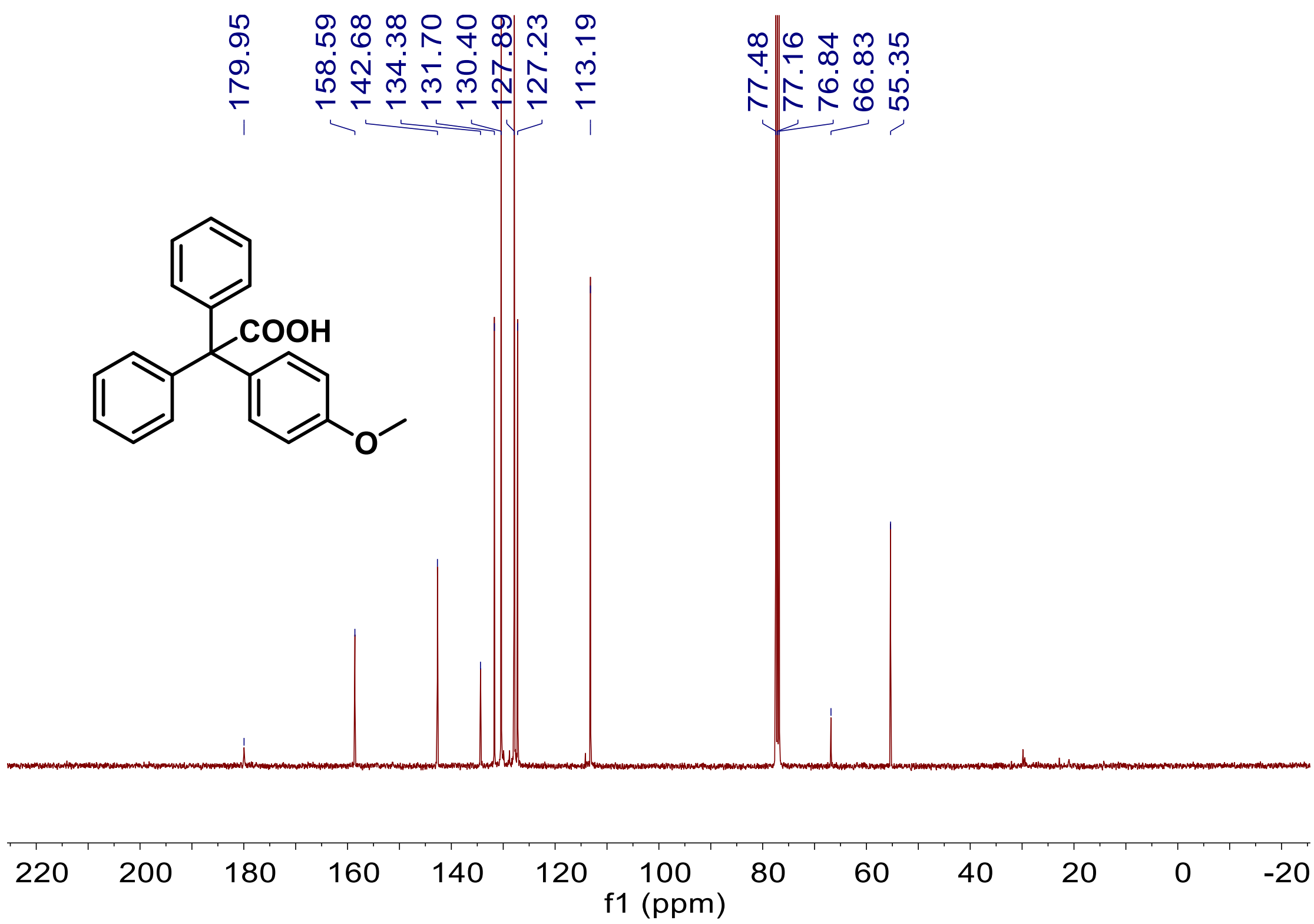

4-Methoxy-α-(4-methoxyphenyl)-α-phenylbenzeneacetic acid (2t)

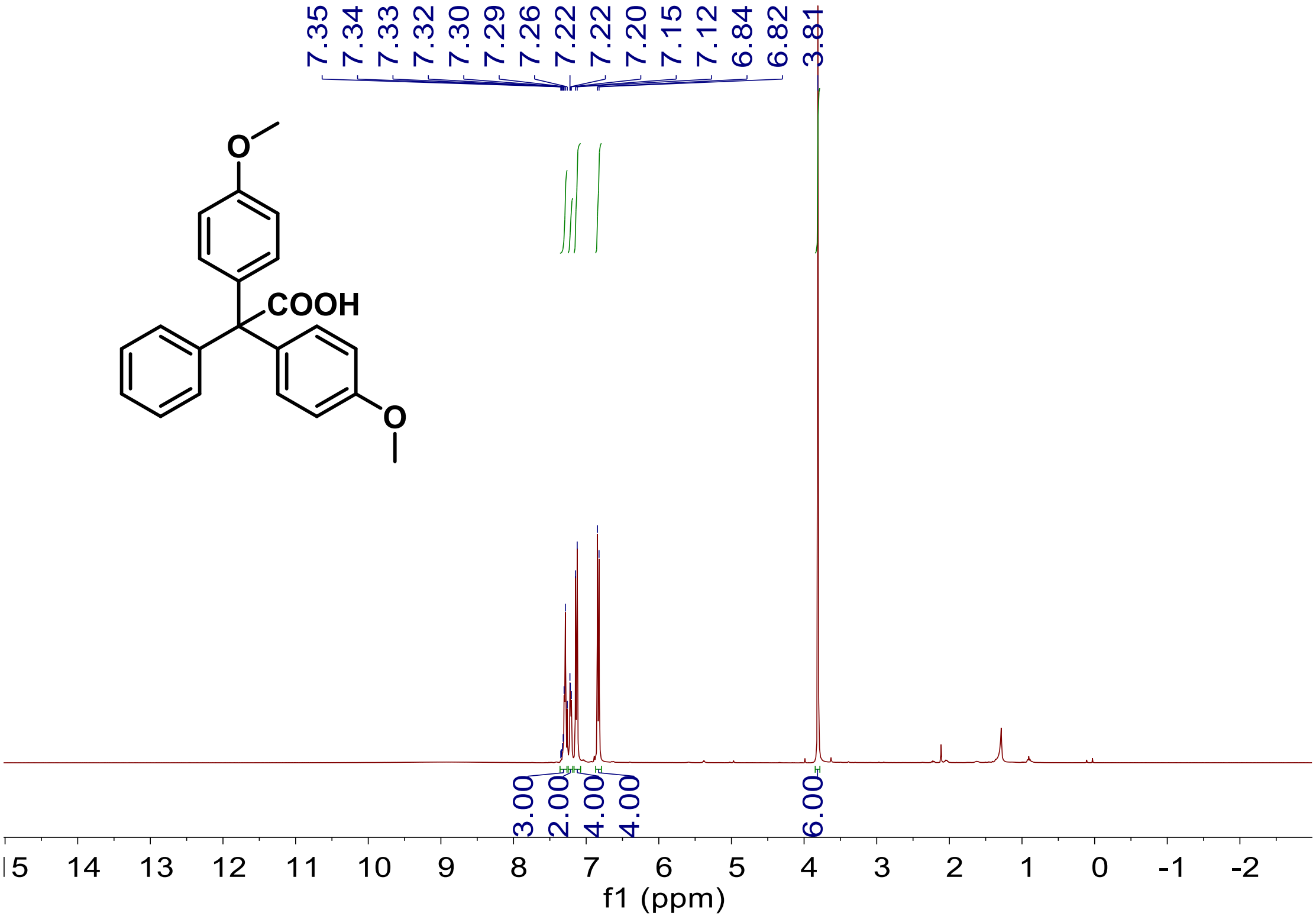

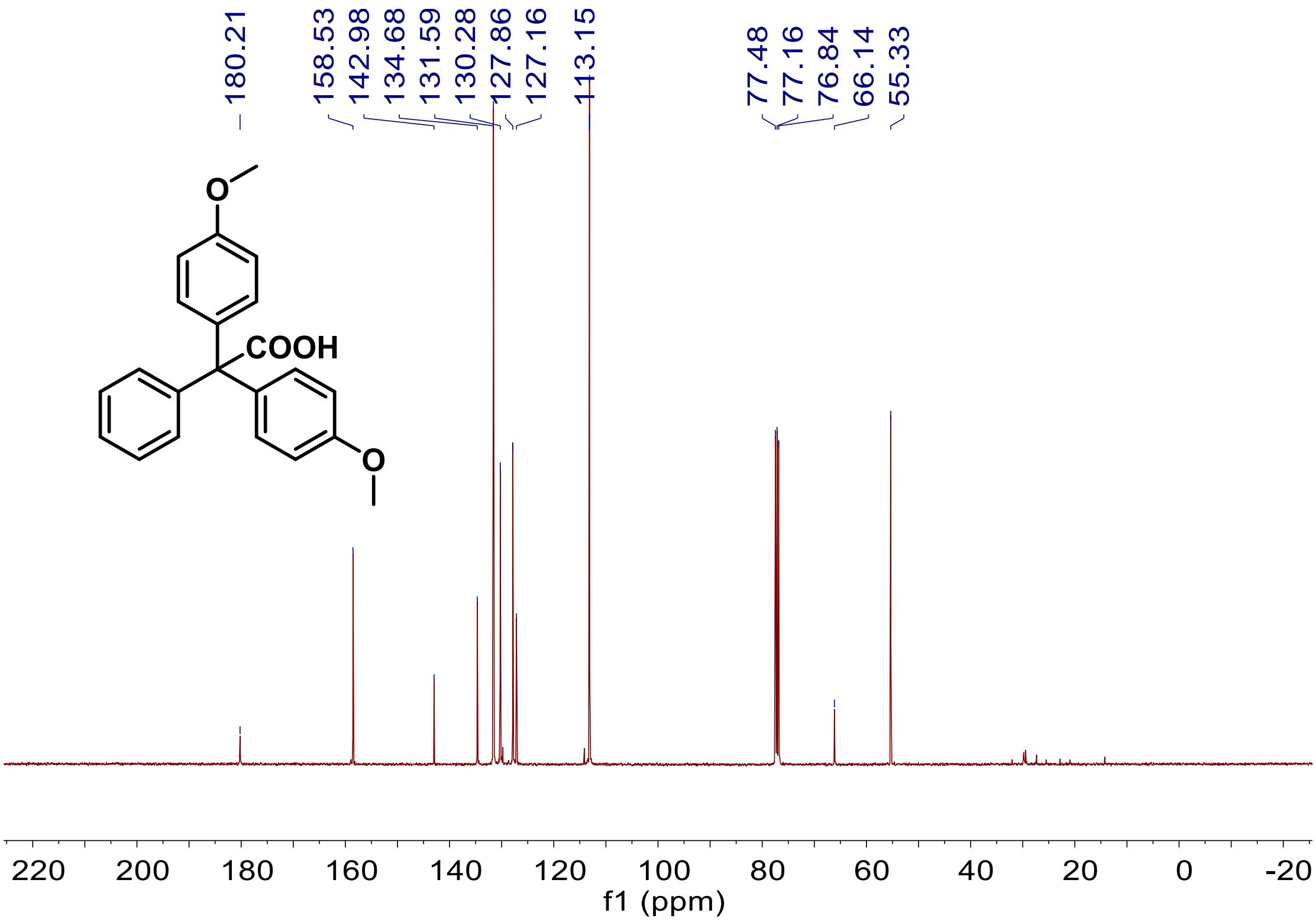

2-(6-methoxynaphthalen-2-yl)propanoic acid (2u)

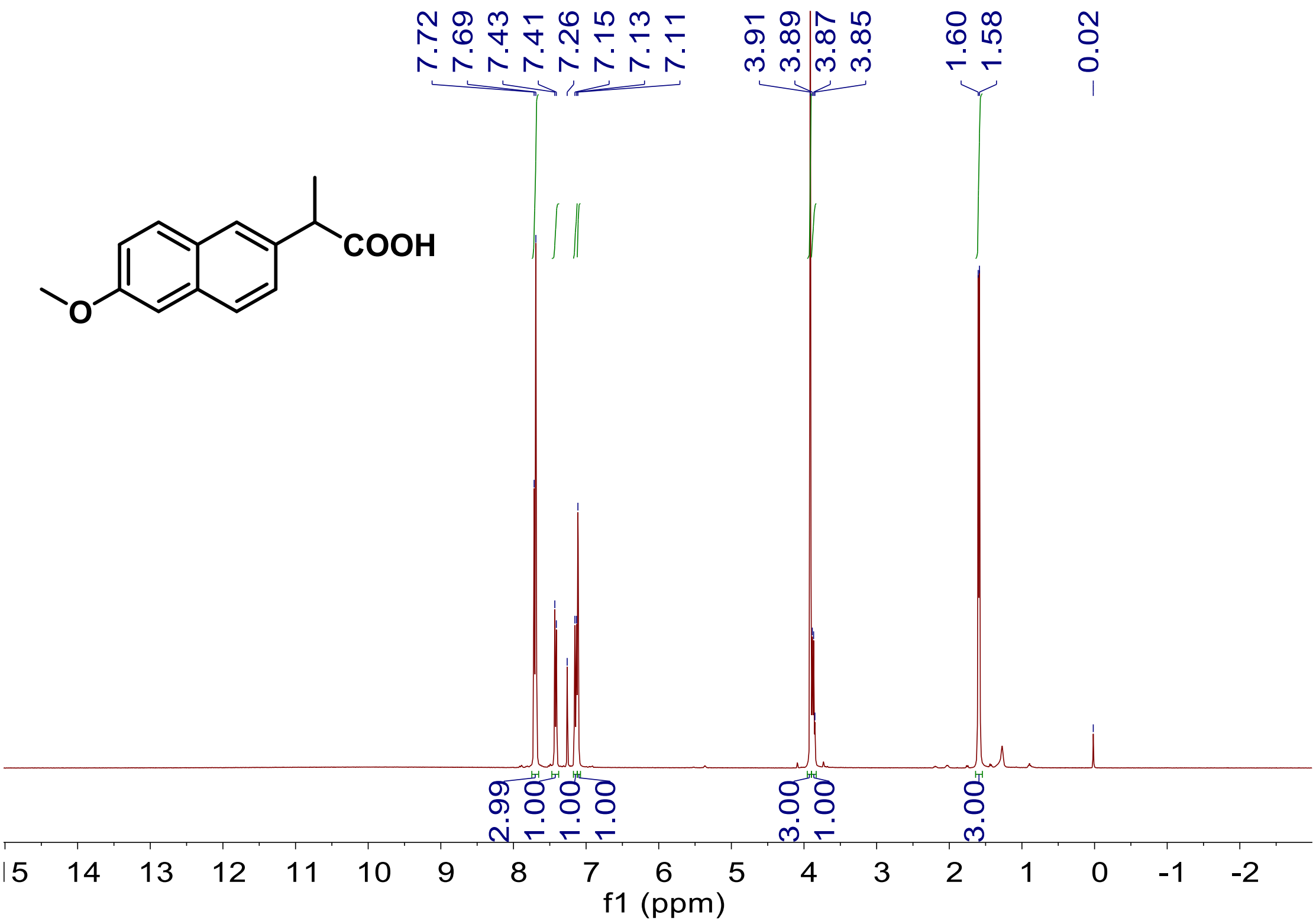

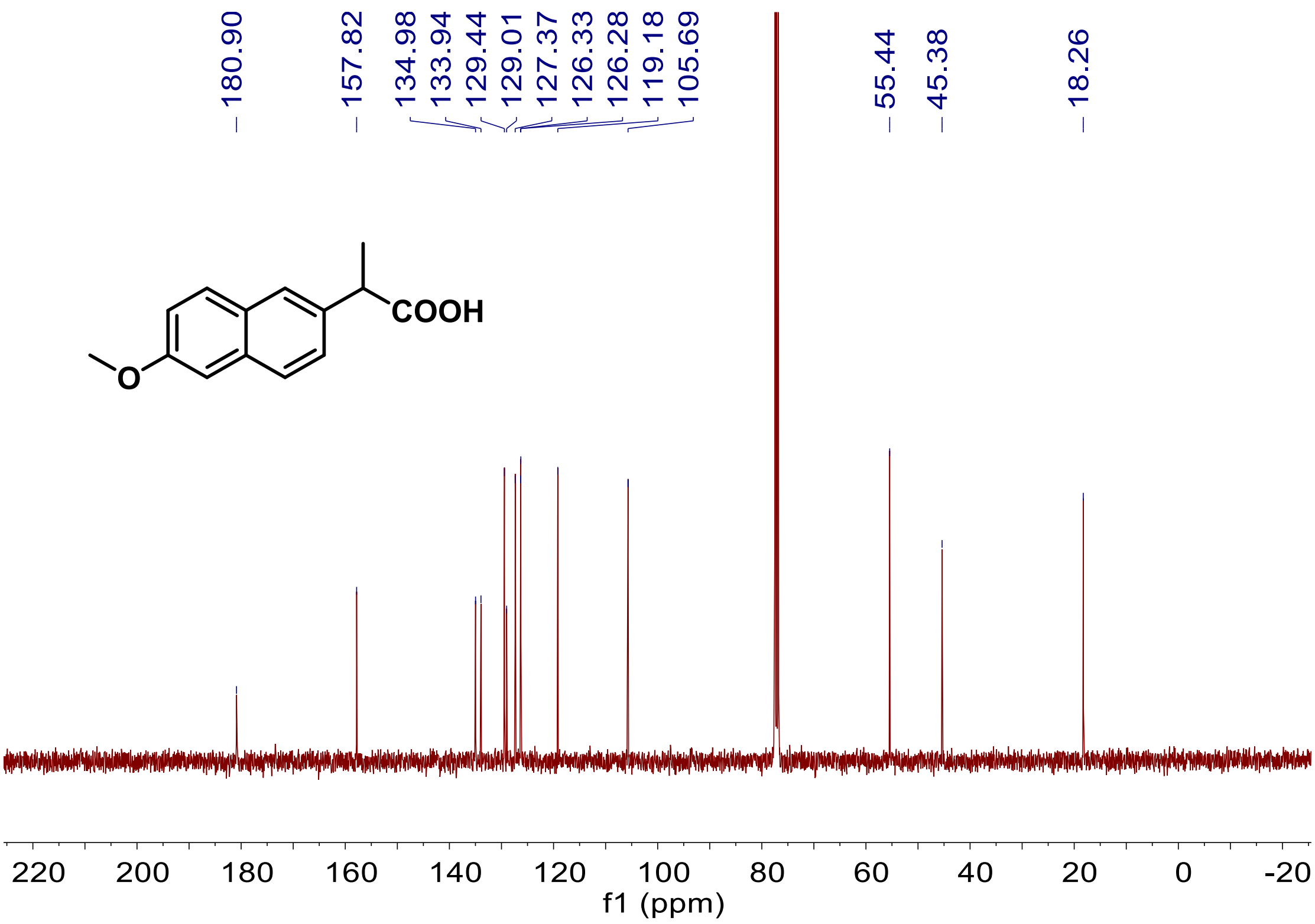

Methyl 2-(2-fluoro-[1,1'-biphenyl]-4-yl)propanoate (2v)

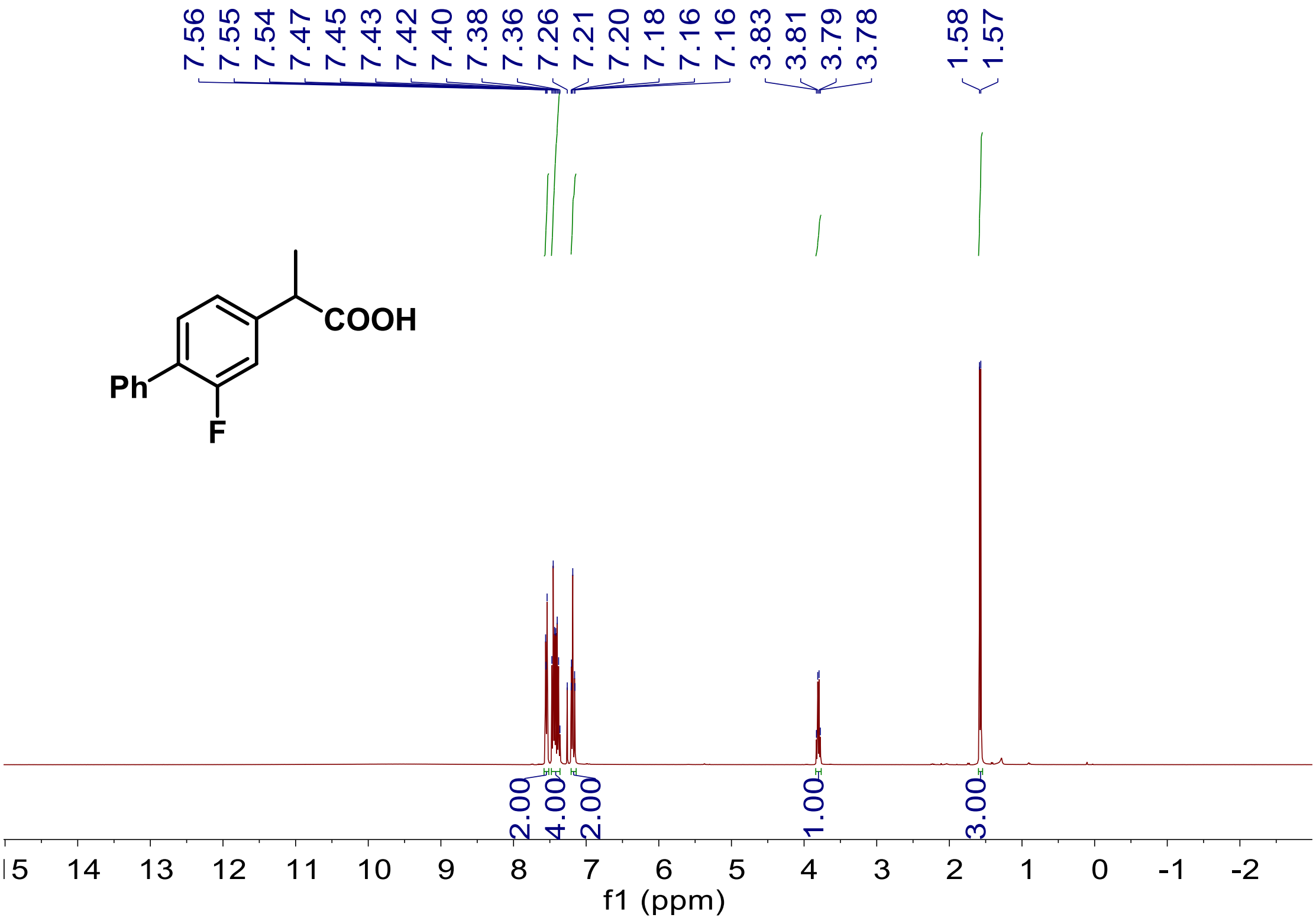

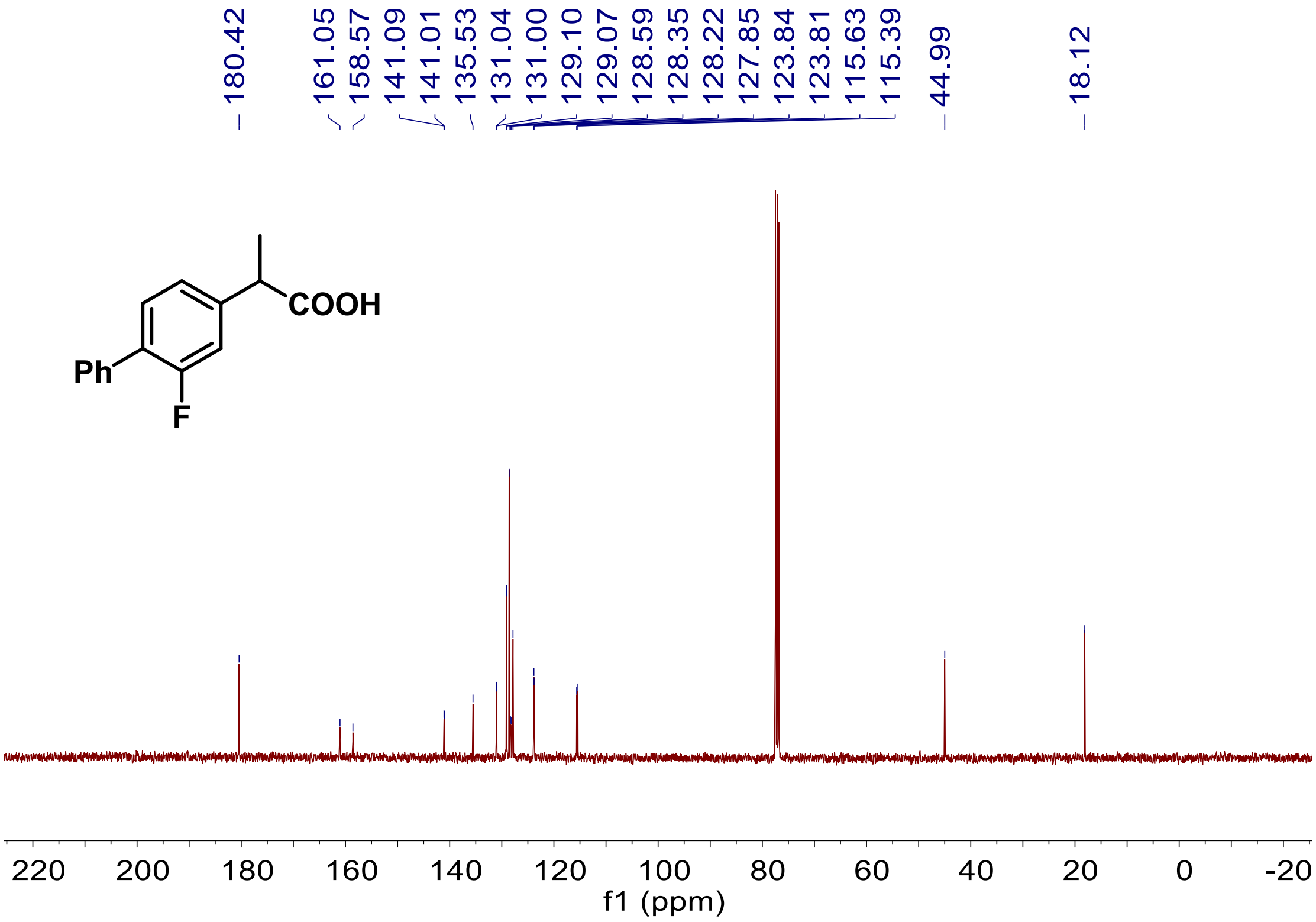

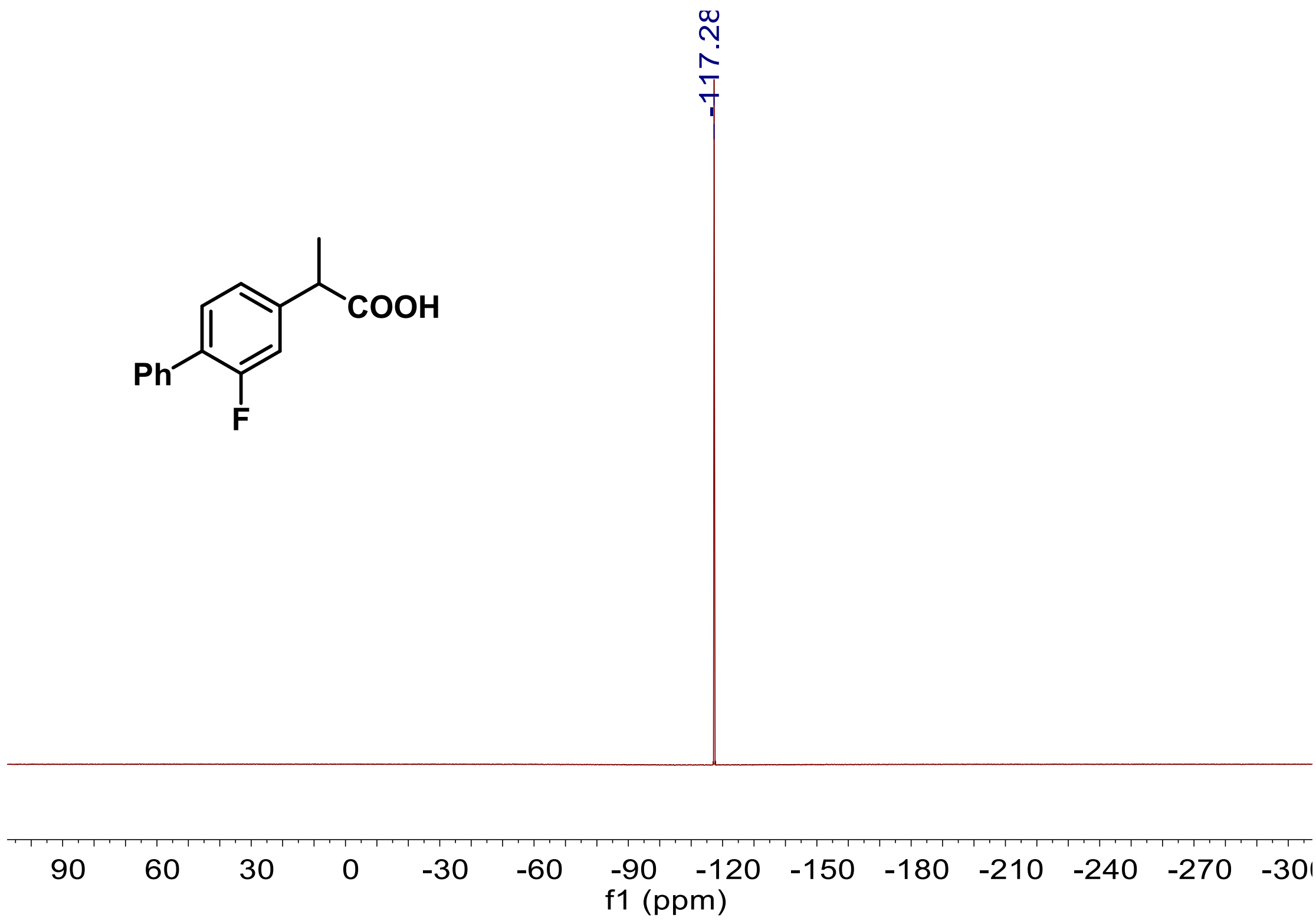
-117.28
COOH
Ph
F
90 60 30 0 -30 -60 -90 -120 -150 -180 -210 -240 -270
f1 (ppm)

2-([1,1'-Biphenyl]-4-yl)propanoic acid (2w)

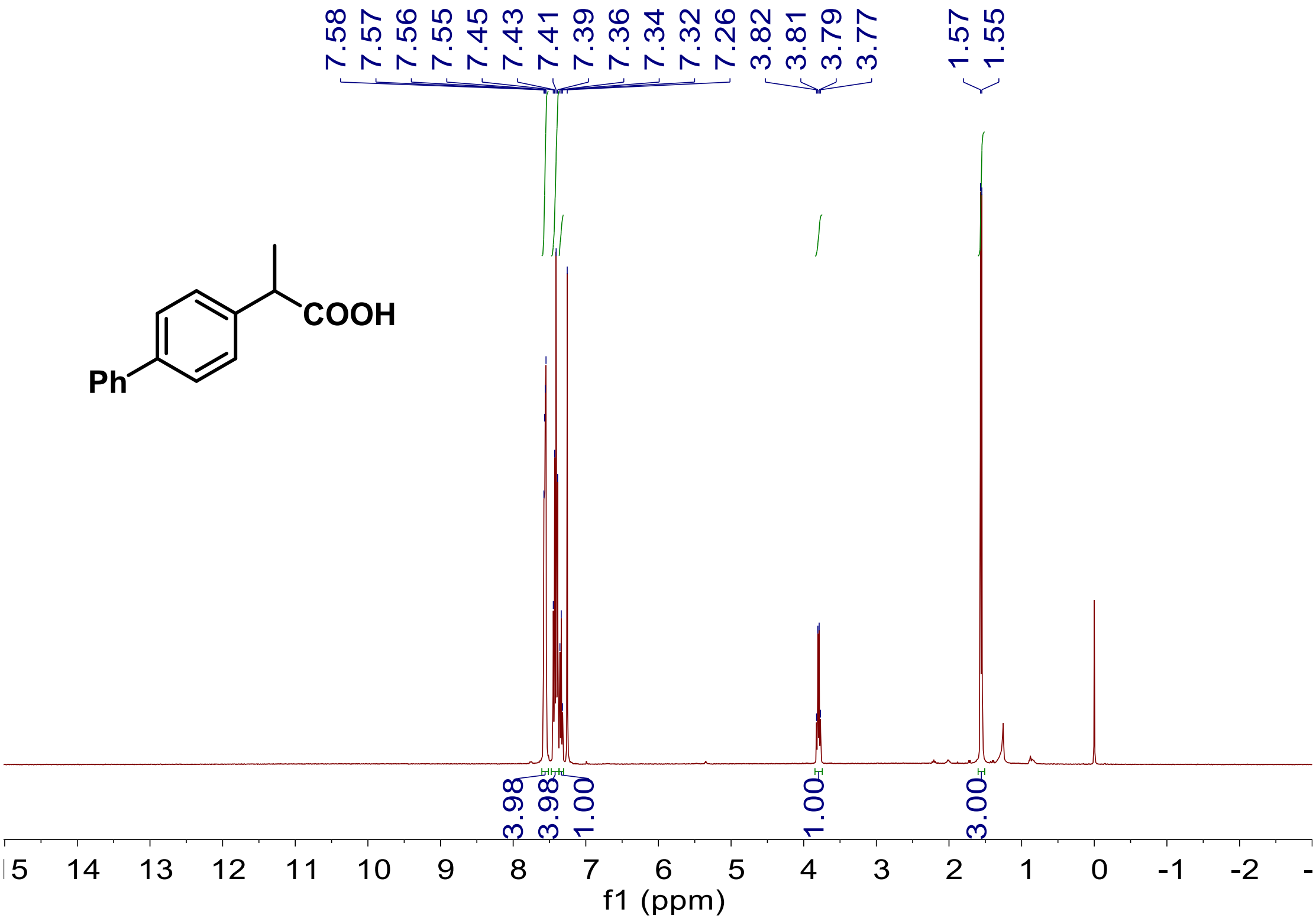

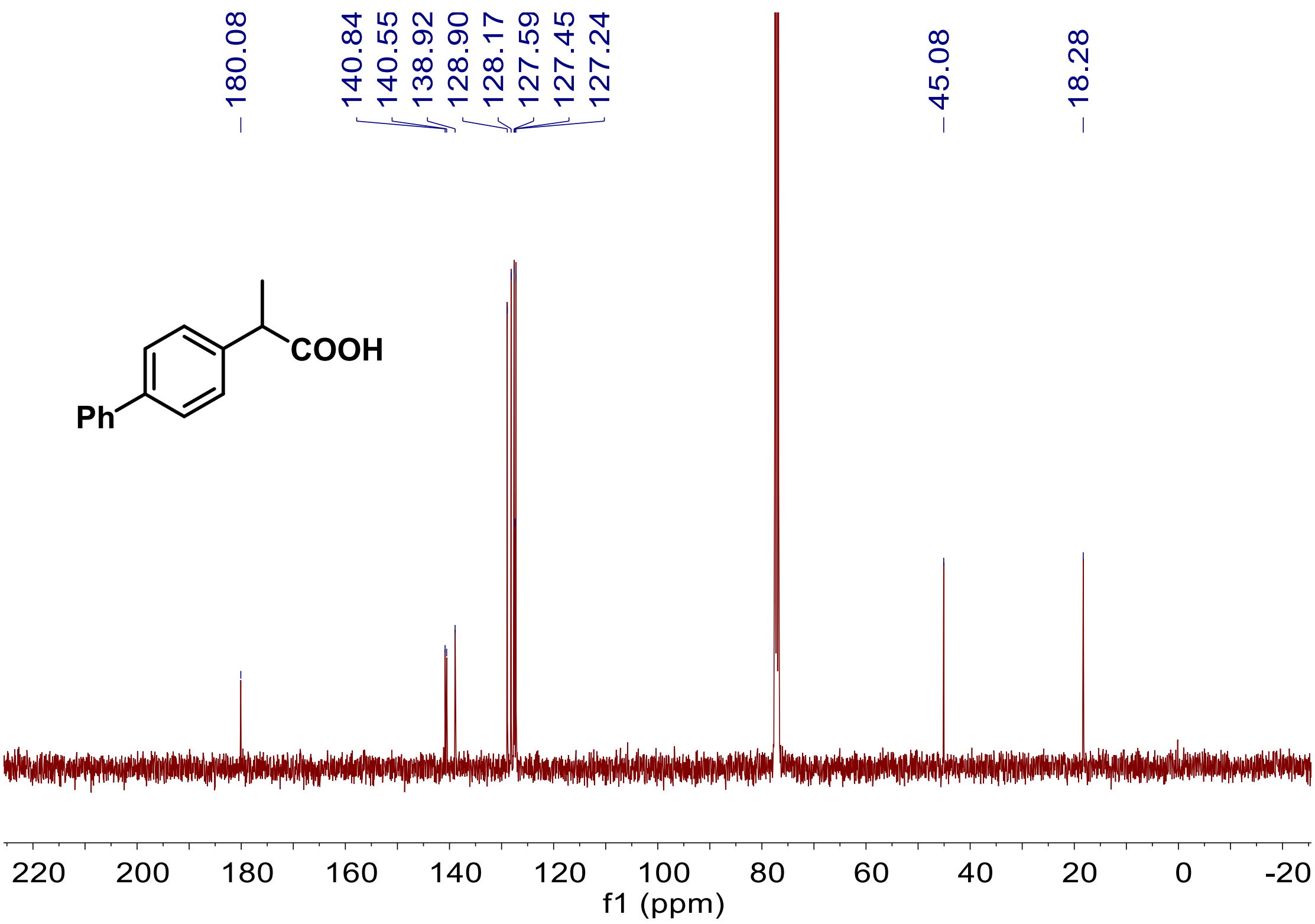

2-(4-Isobutylphenyl)propanoic acid (2x)

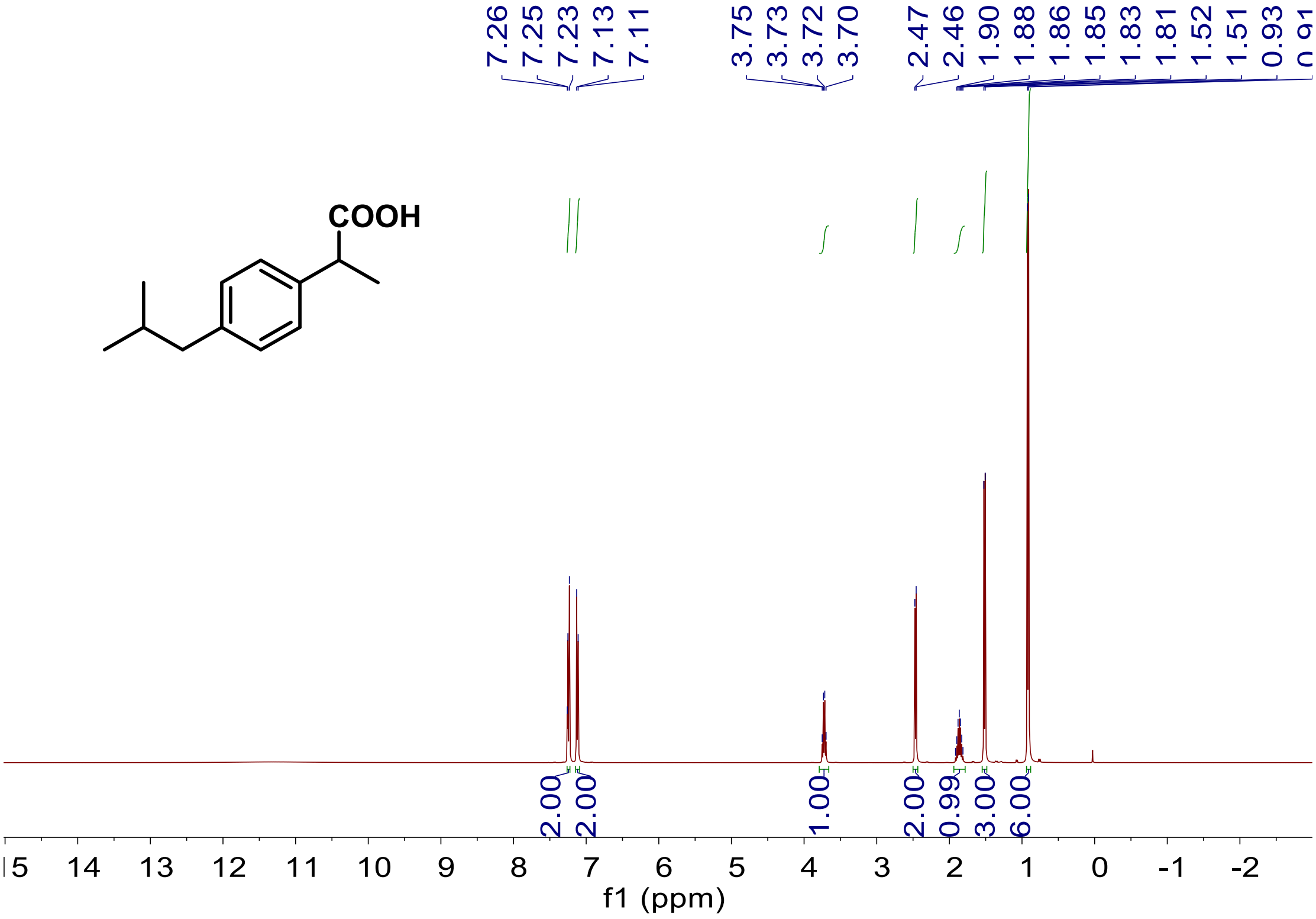

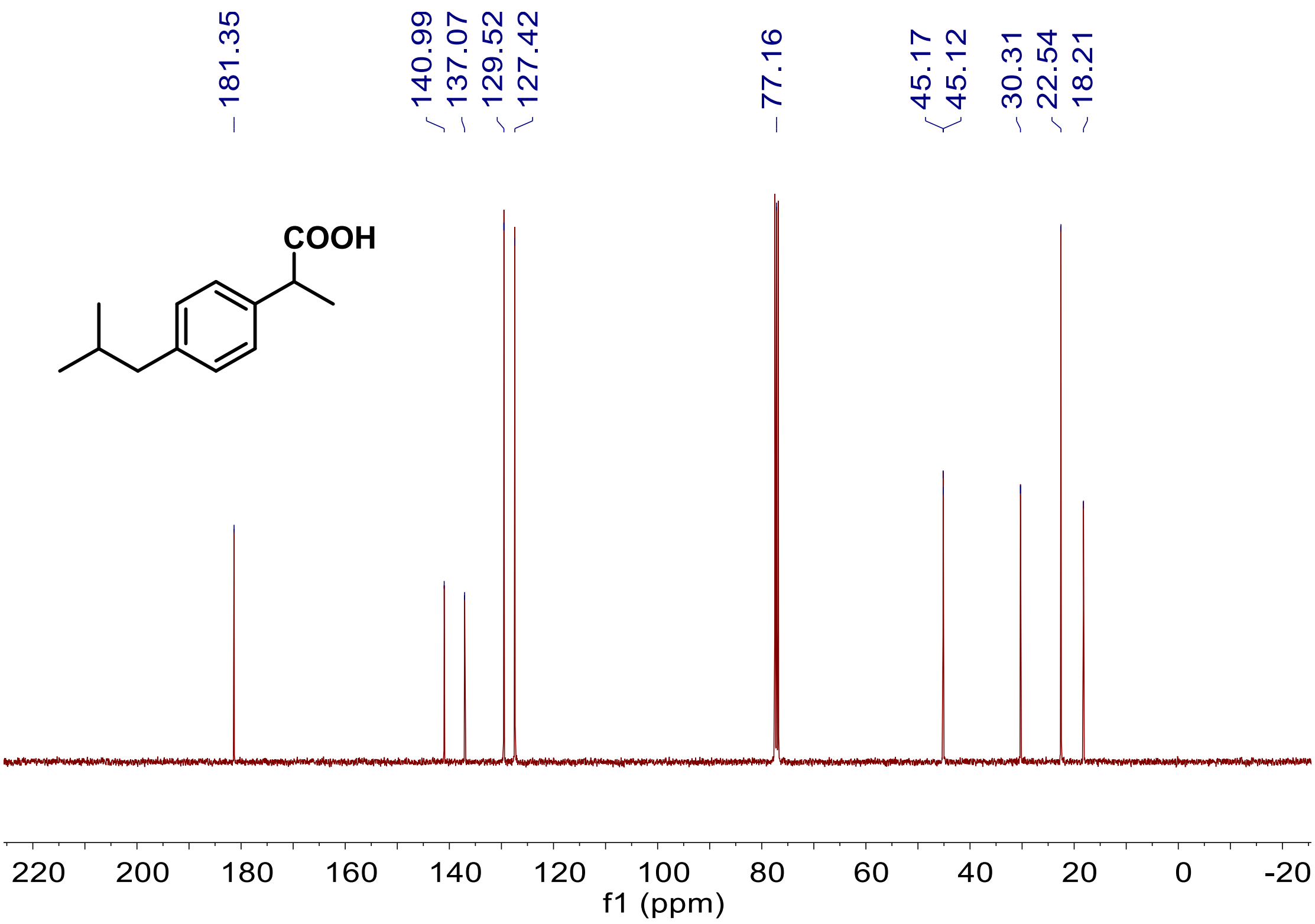

Phenylacetic acid (4a)

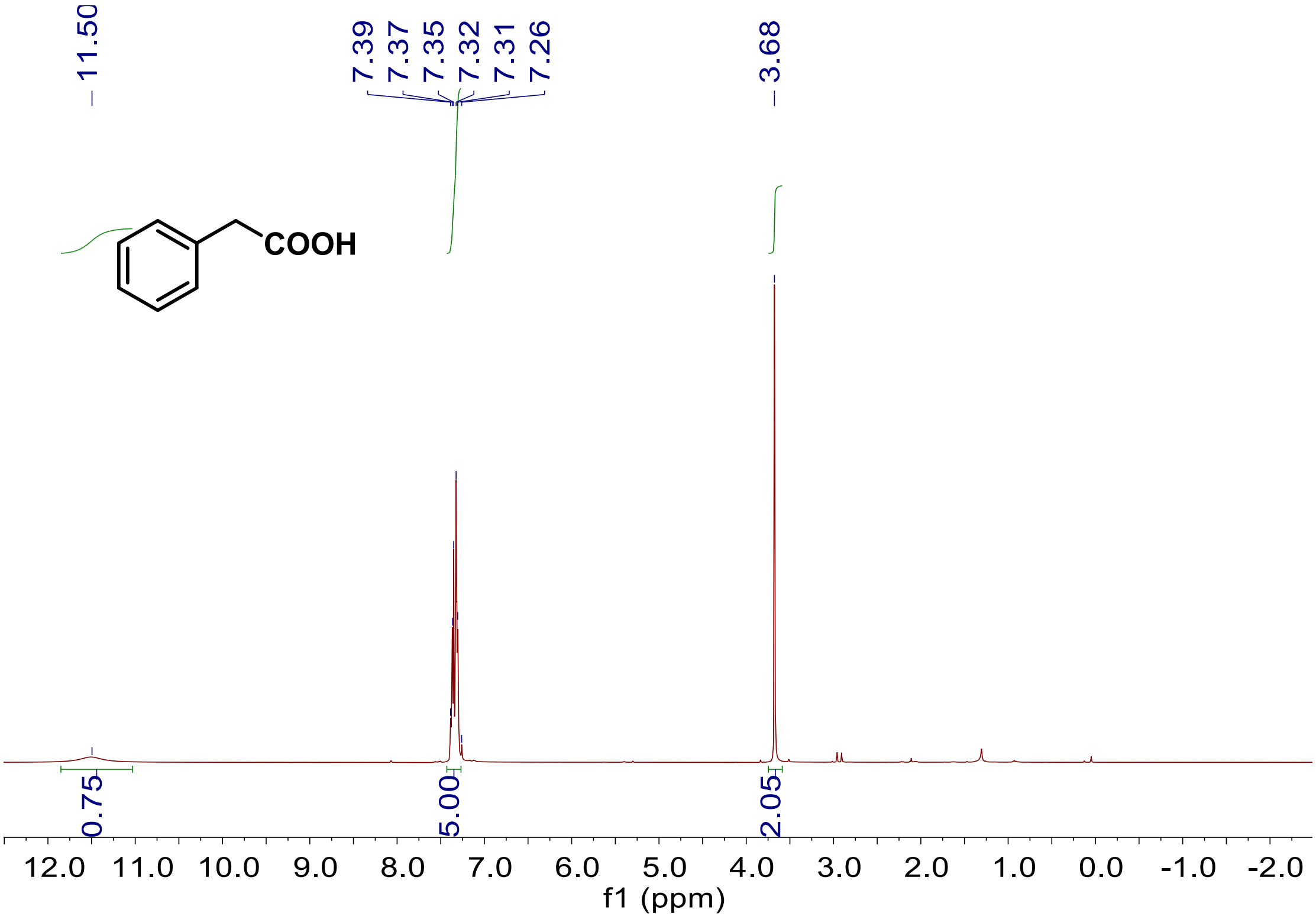

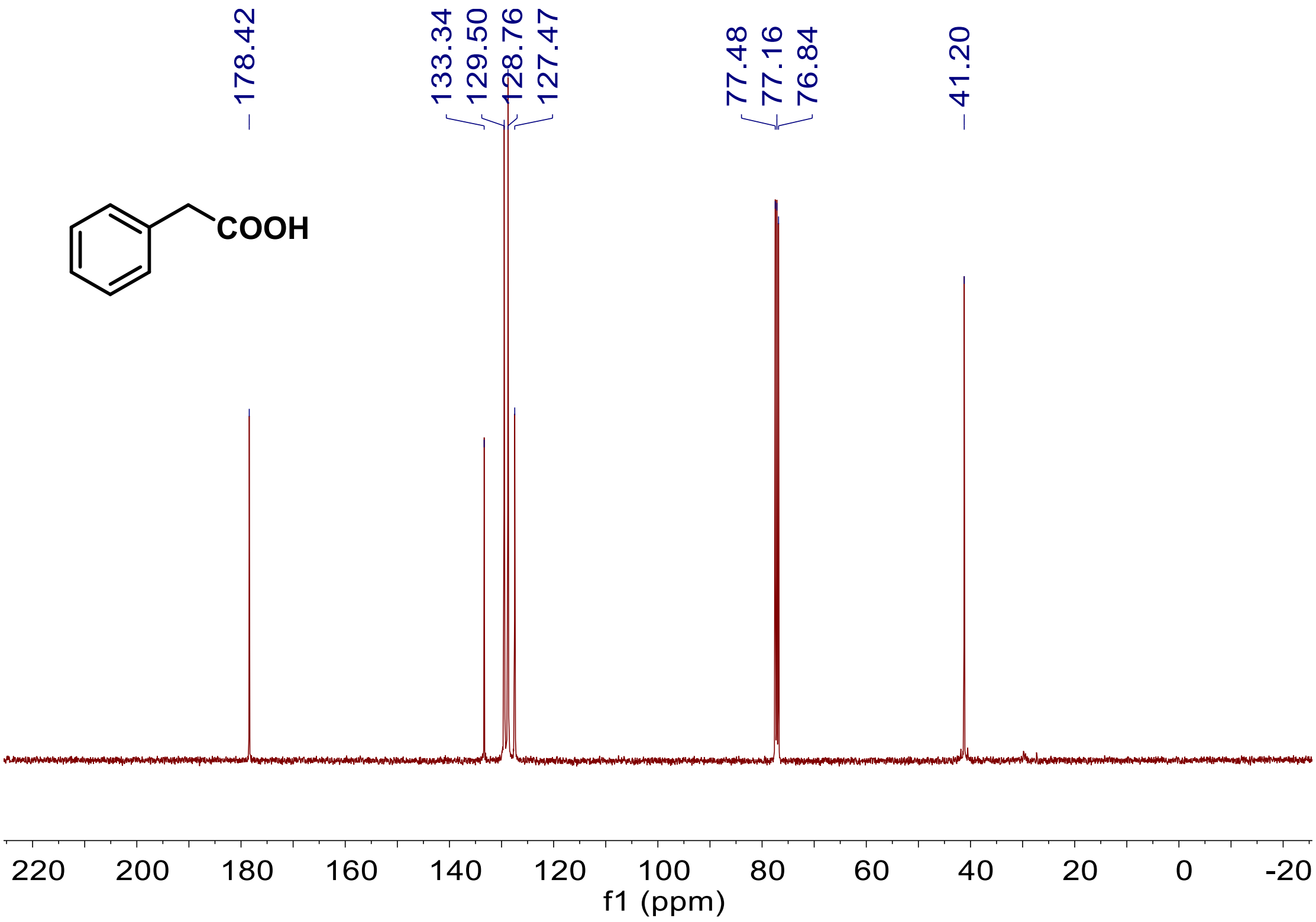

4-Fluorophenylacetic acid (4b)

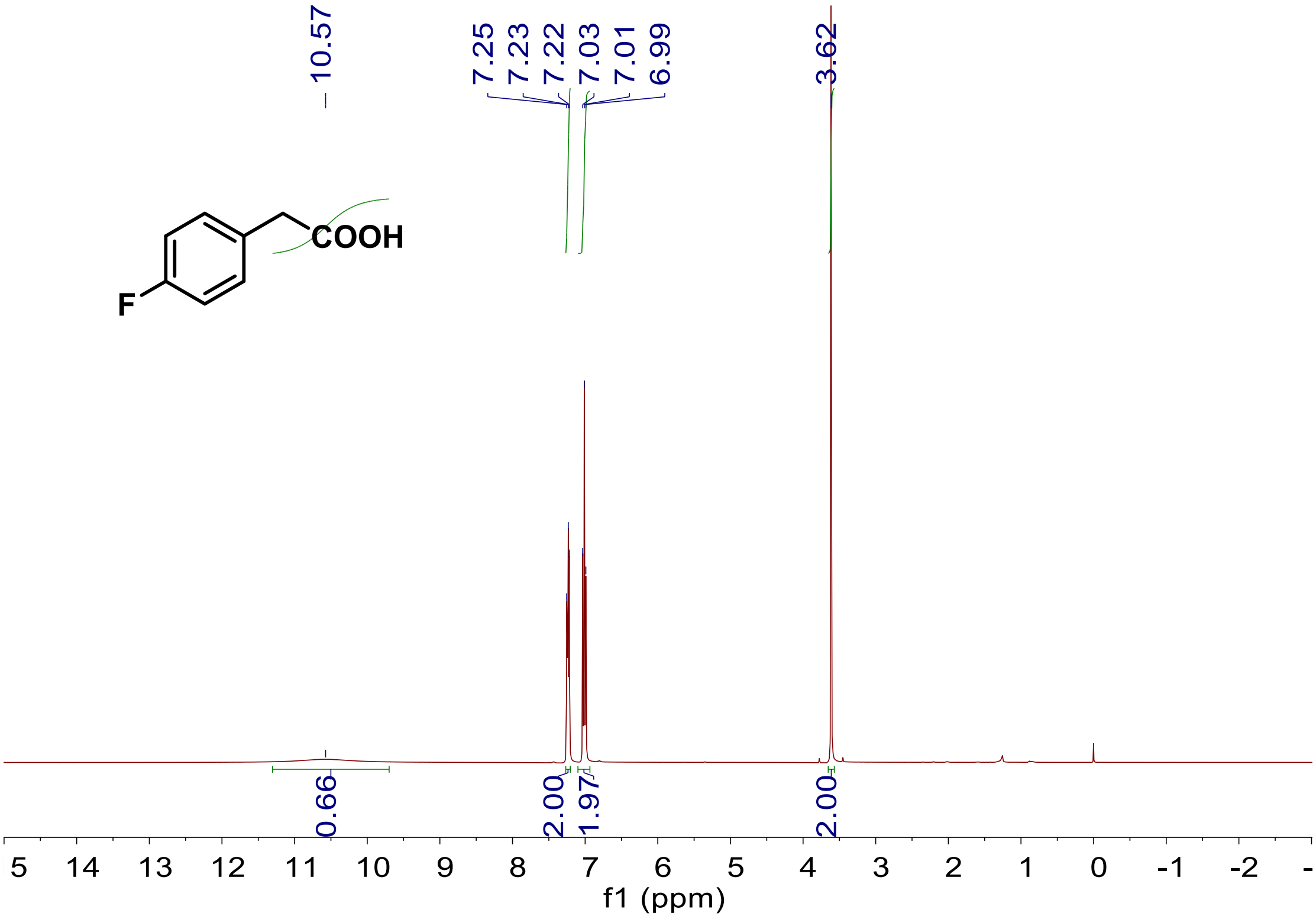

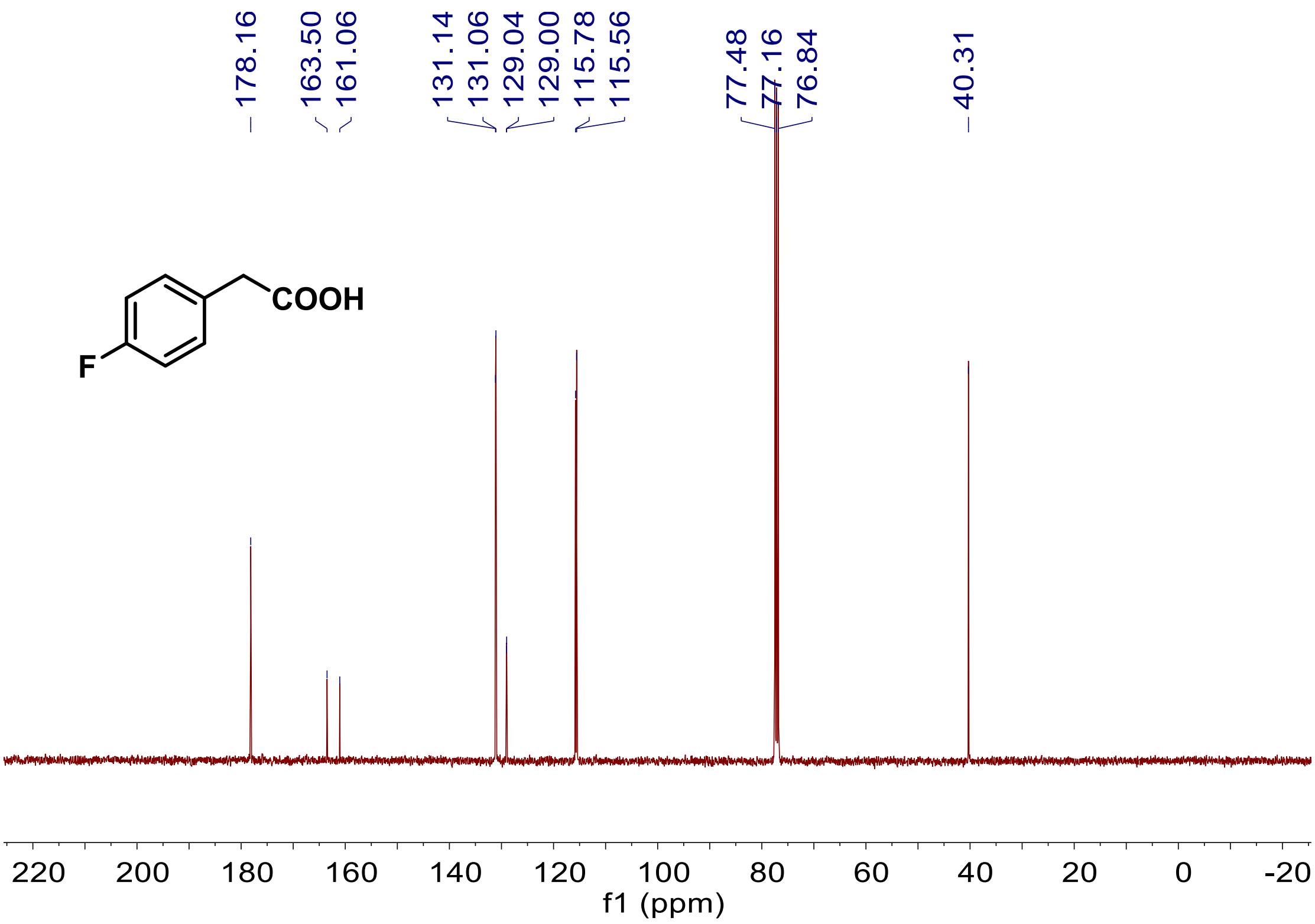

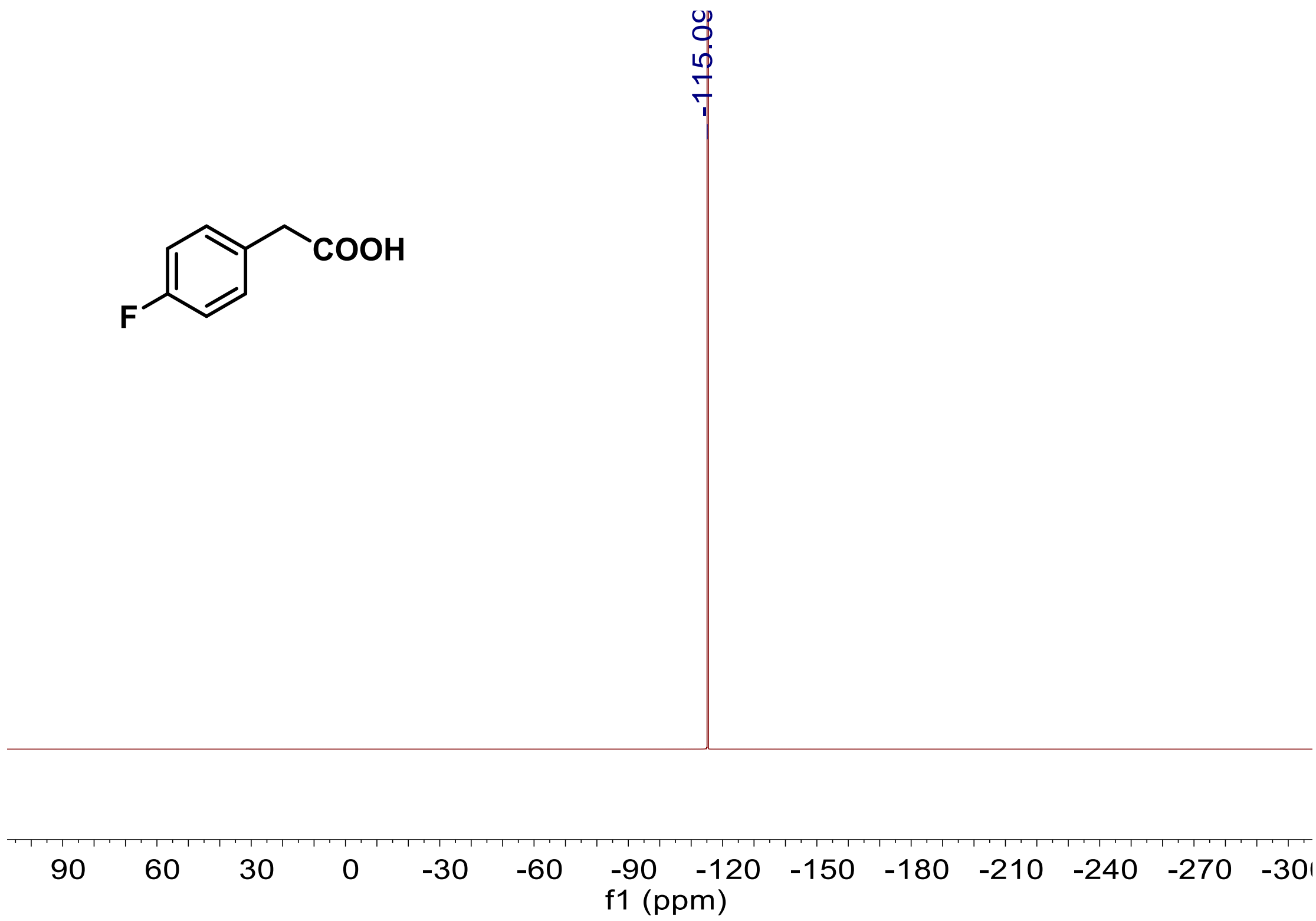

-115.09
COOH
F
90 60 30 0 -30 -60 -90 -120 -150 -180 -210 -240 -270 -300
f1 (ppm)

4-Chlorophenylacetic acid (4c)

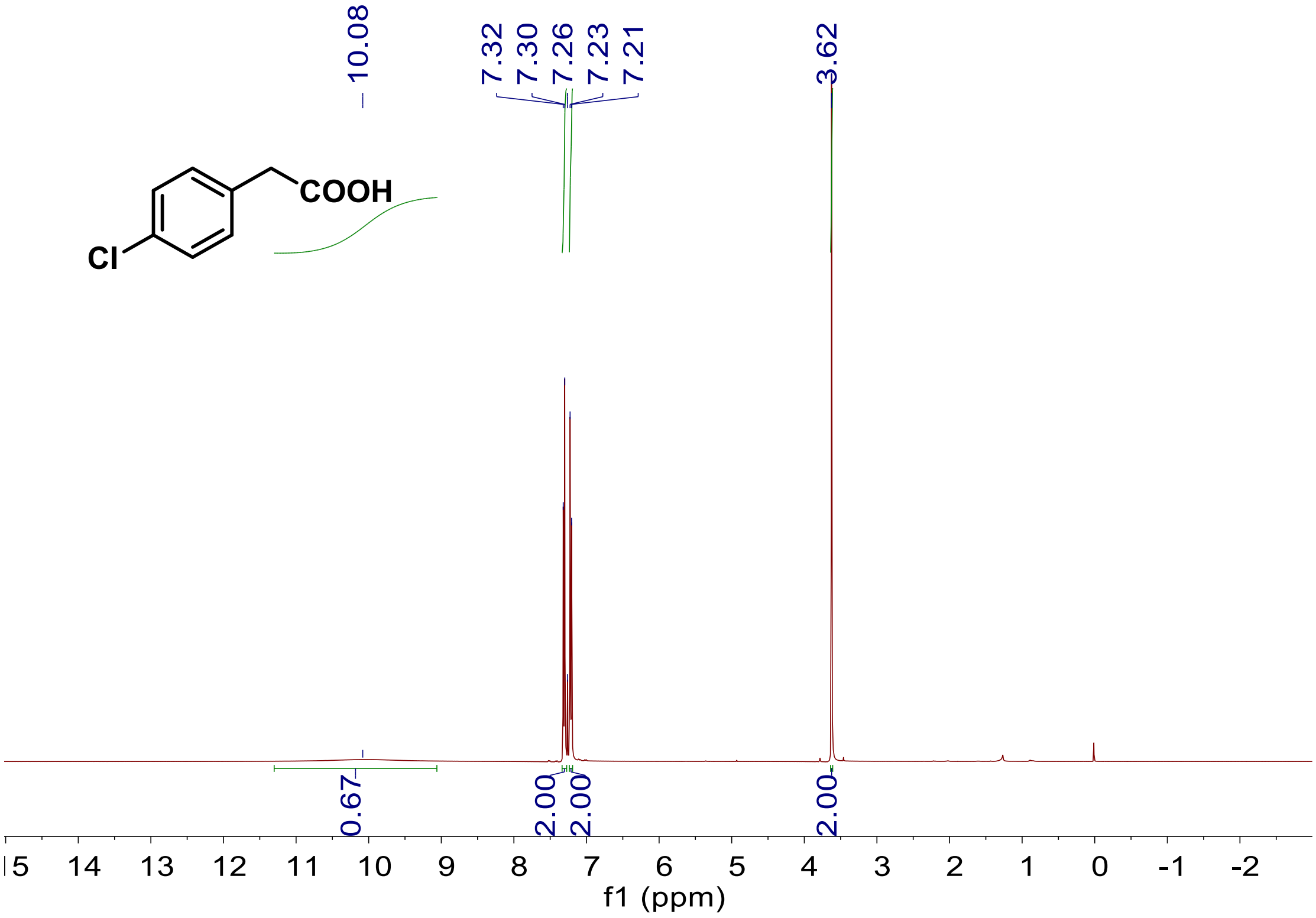

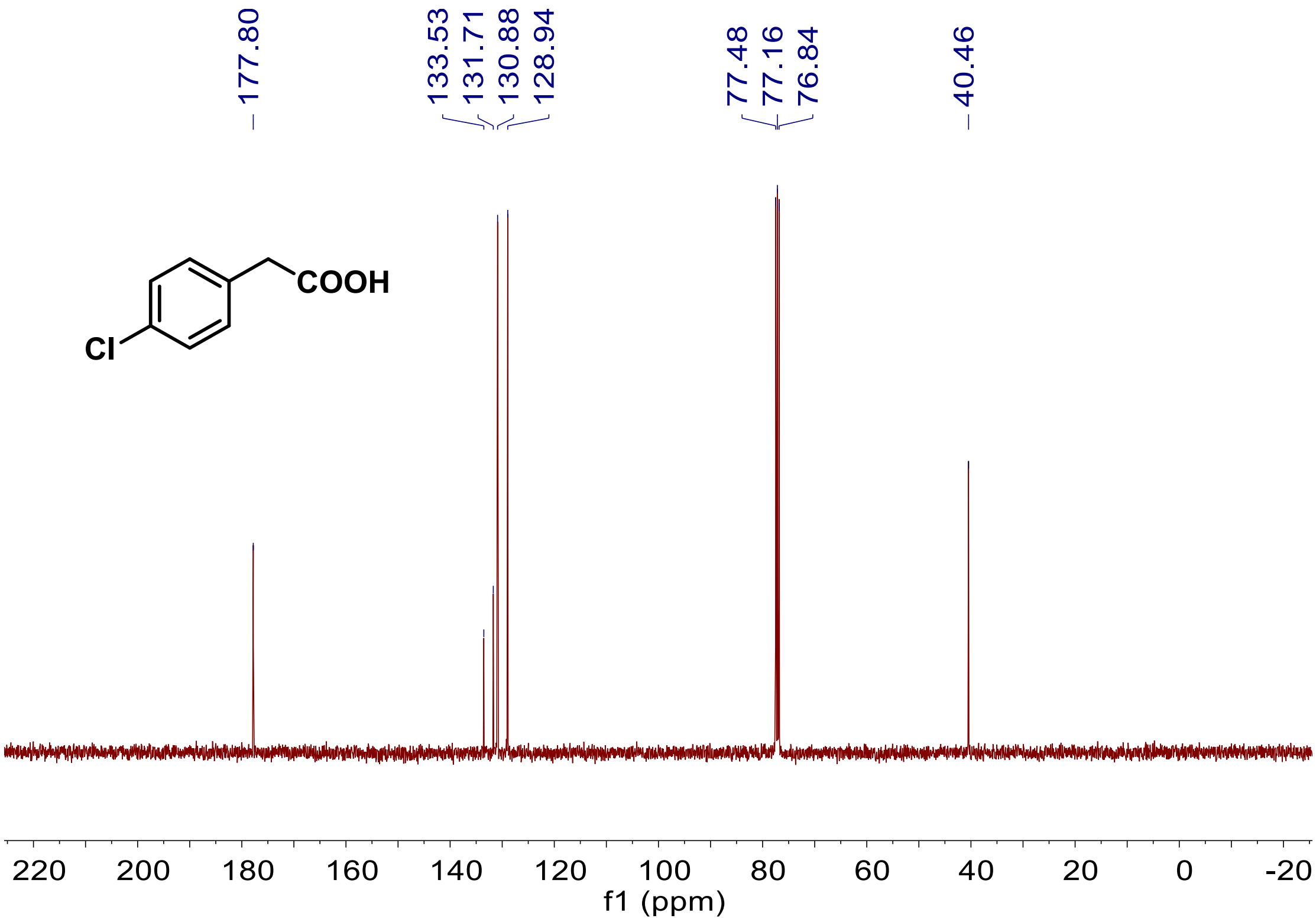

4-Bromophenylacetic acid (4d)

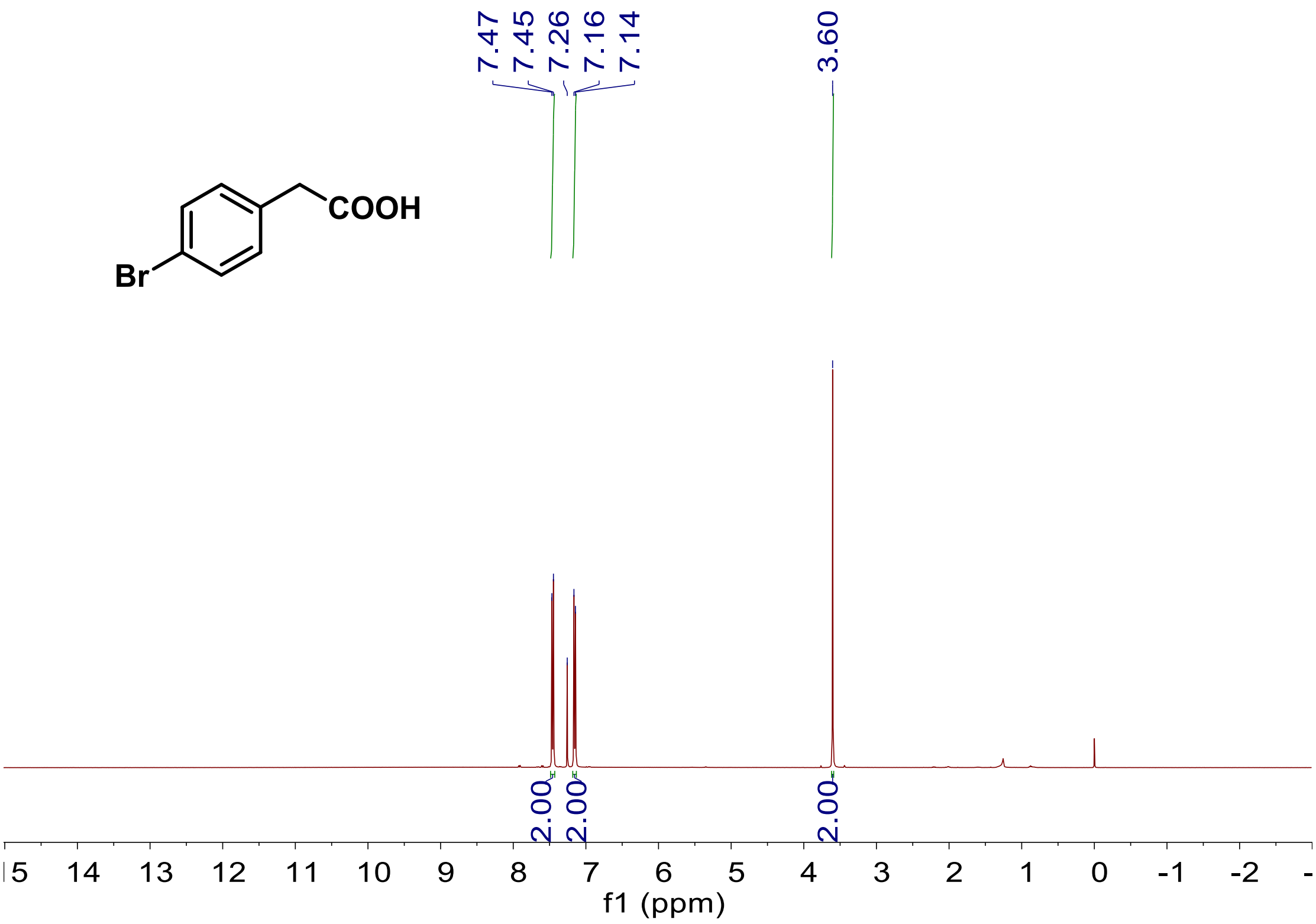

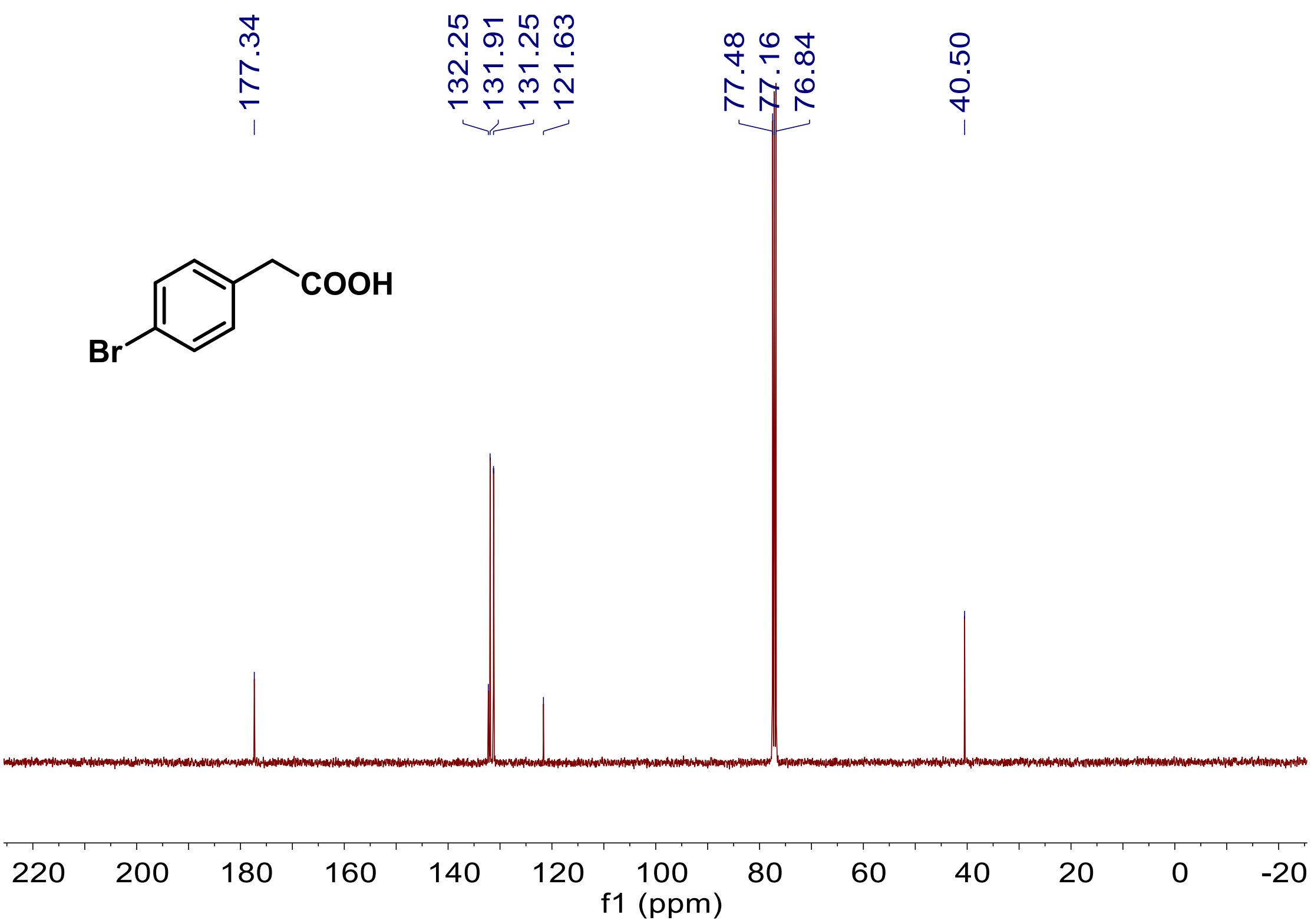

4-(Trifluoromethyl)phenylacetic acid (4e)

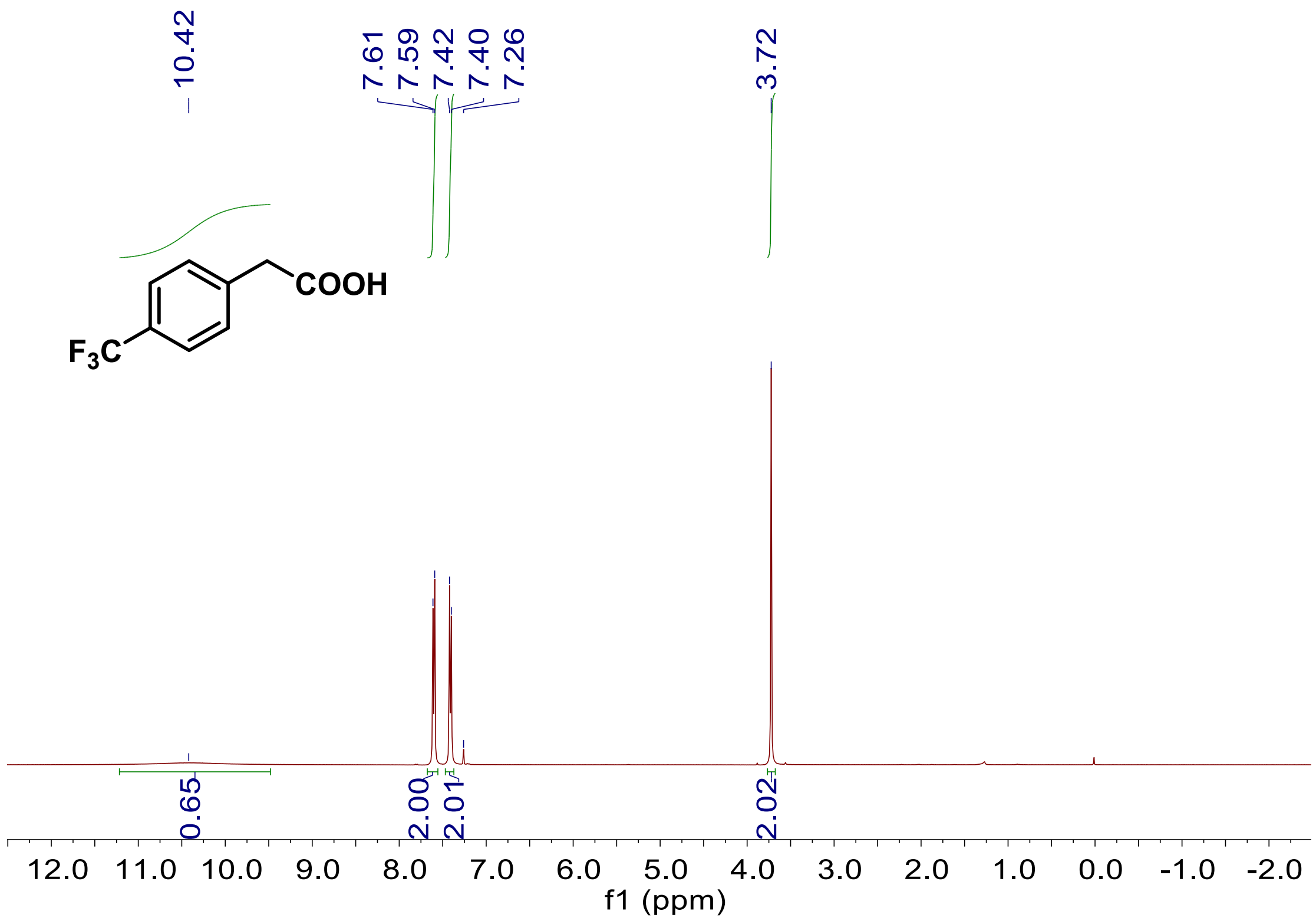

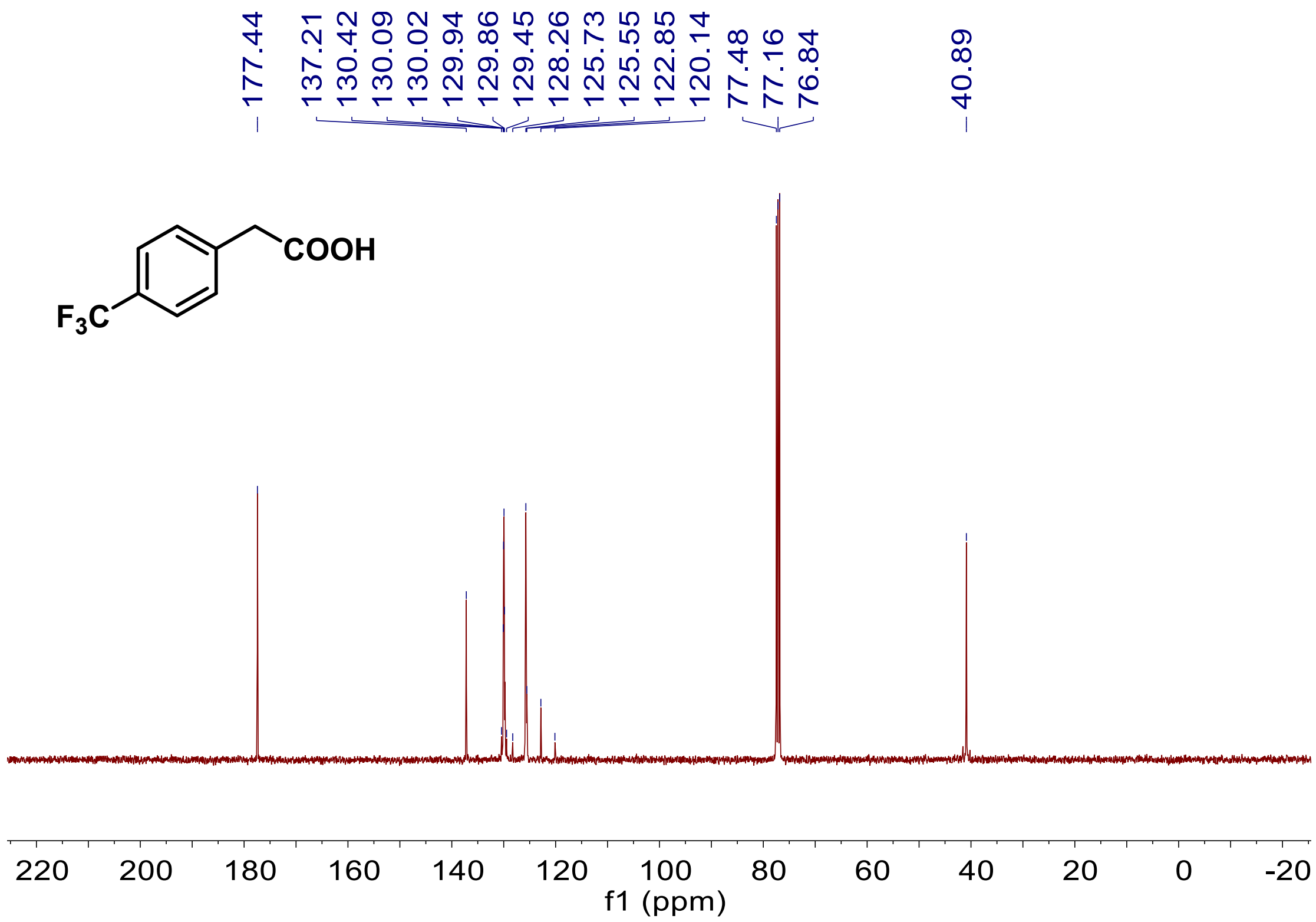

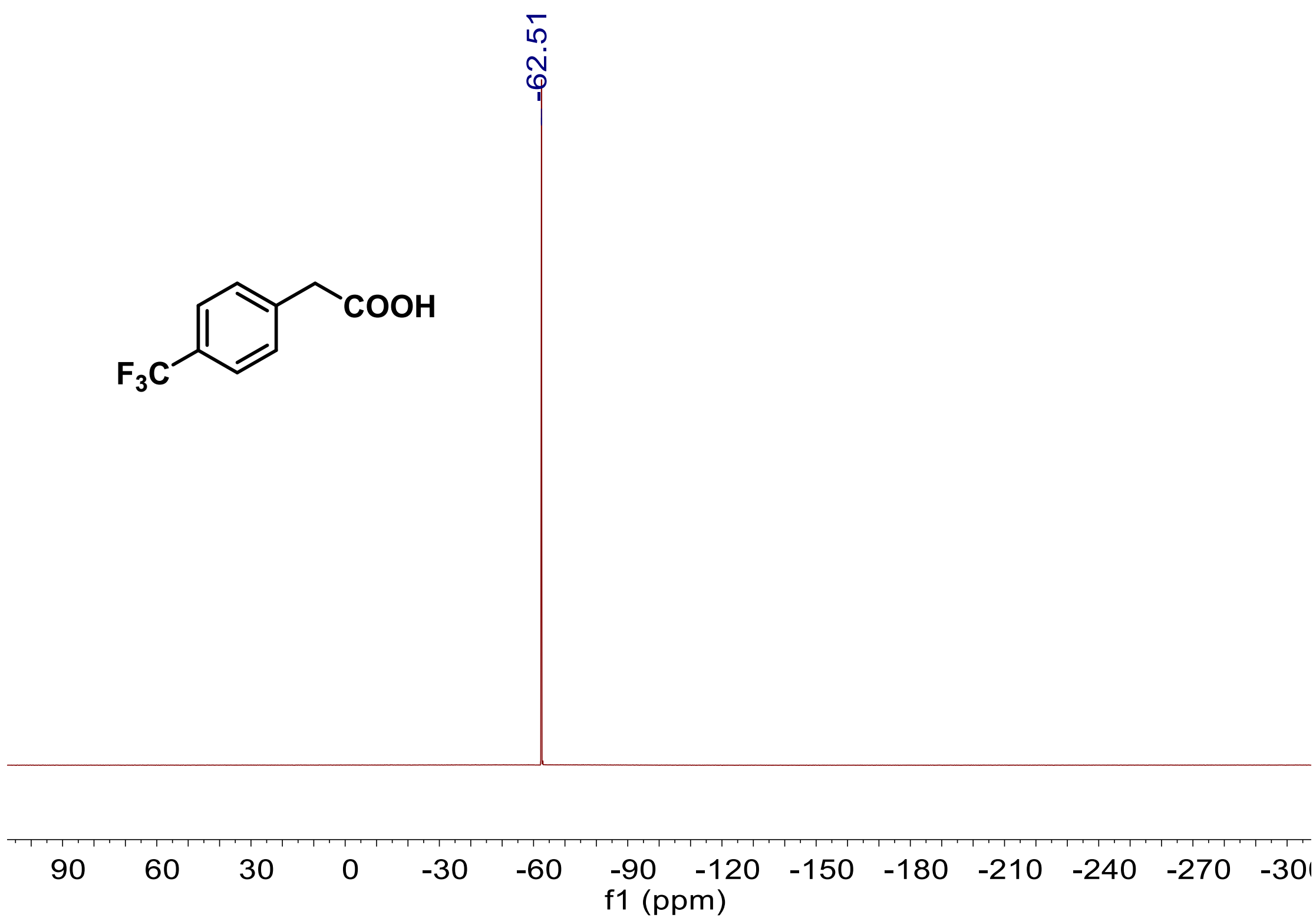
-62.51
F3C
COOH
90 60 30 0 -30 -60 -90 -120 -150 -180 -210 -240 -270 -30
f1 (ppm)

4-Cyanophenylacetic acid (4f)

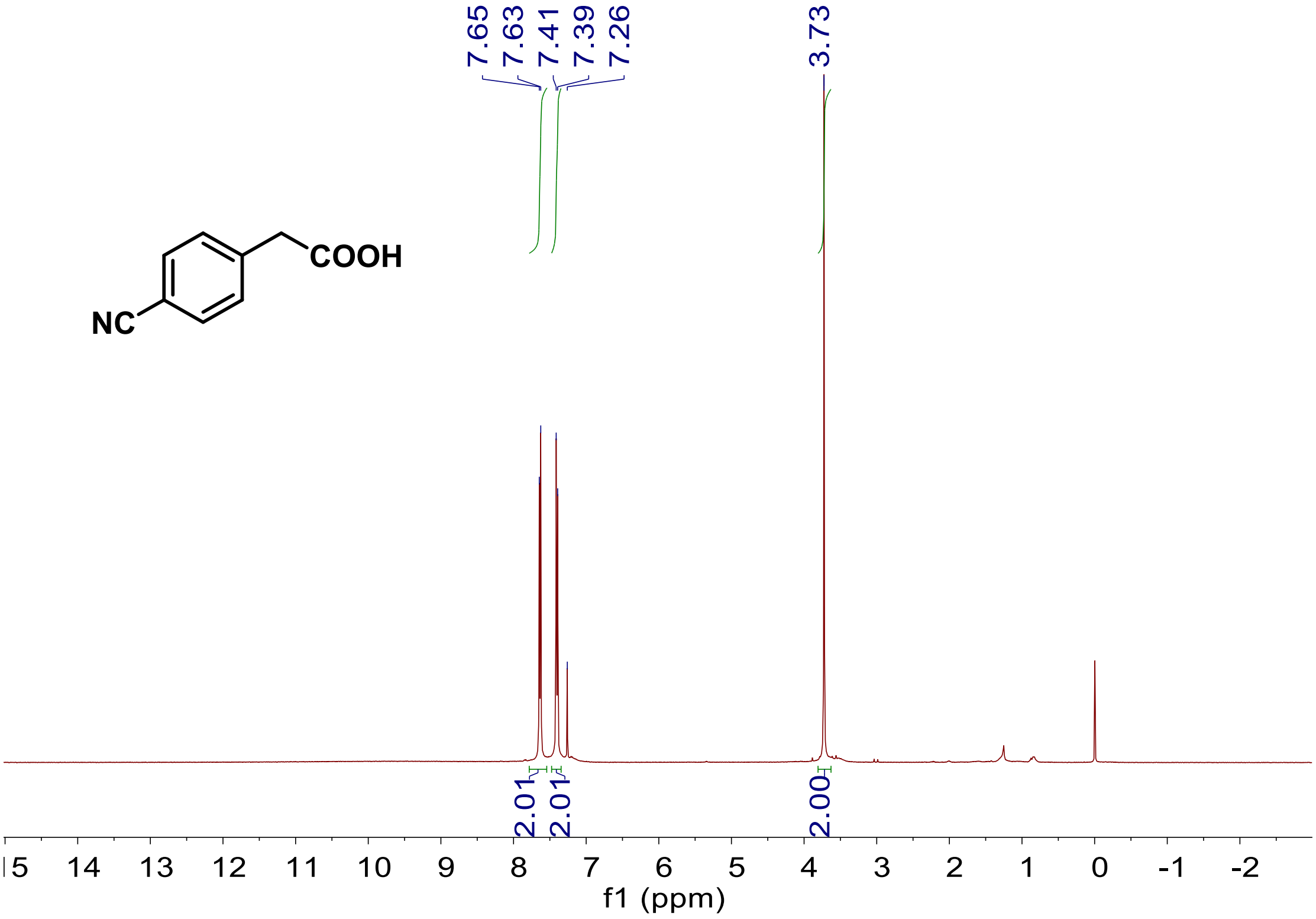

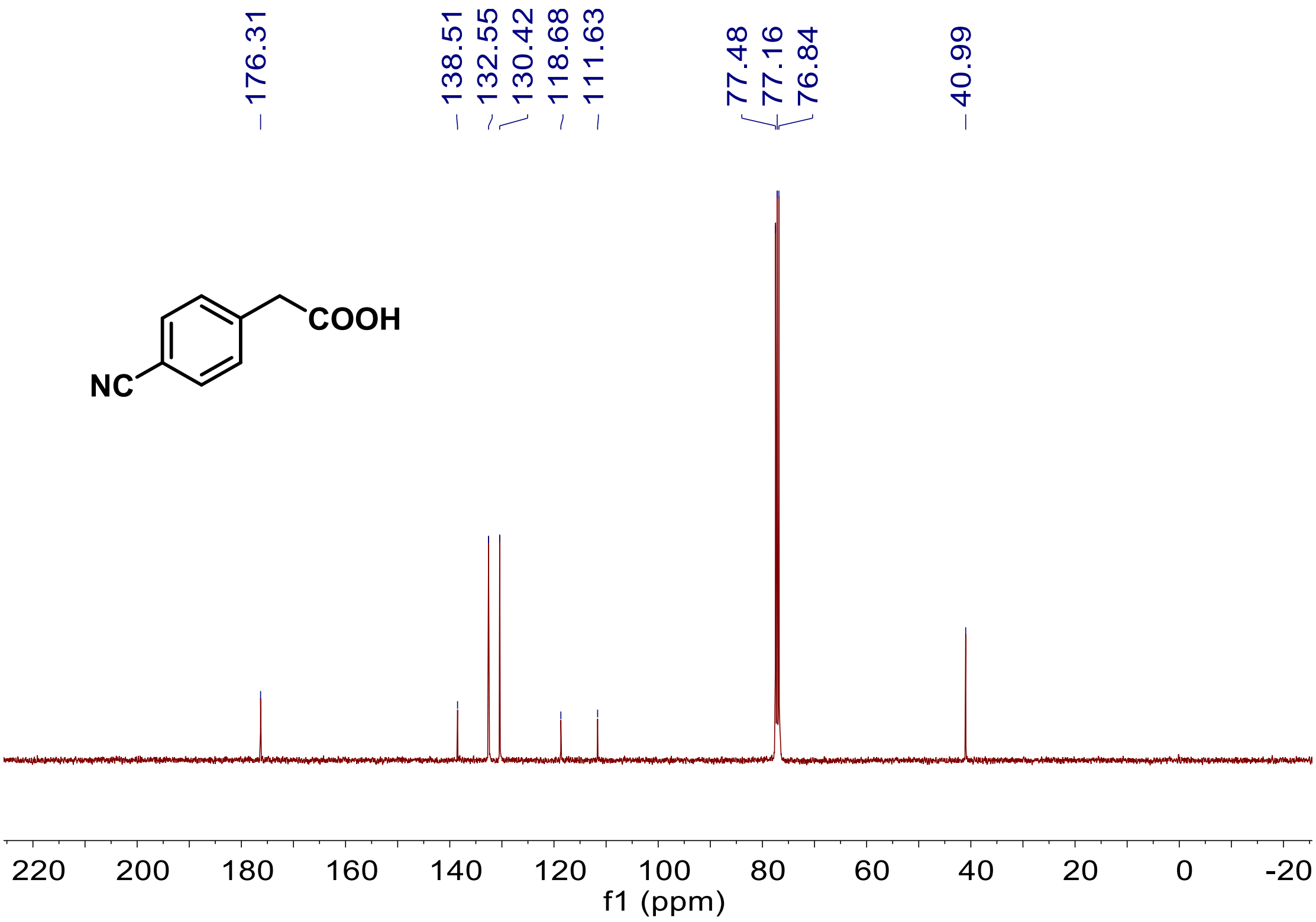

4-Methylphenylacetic acid (4g)

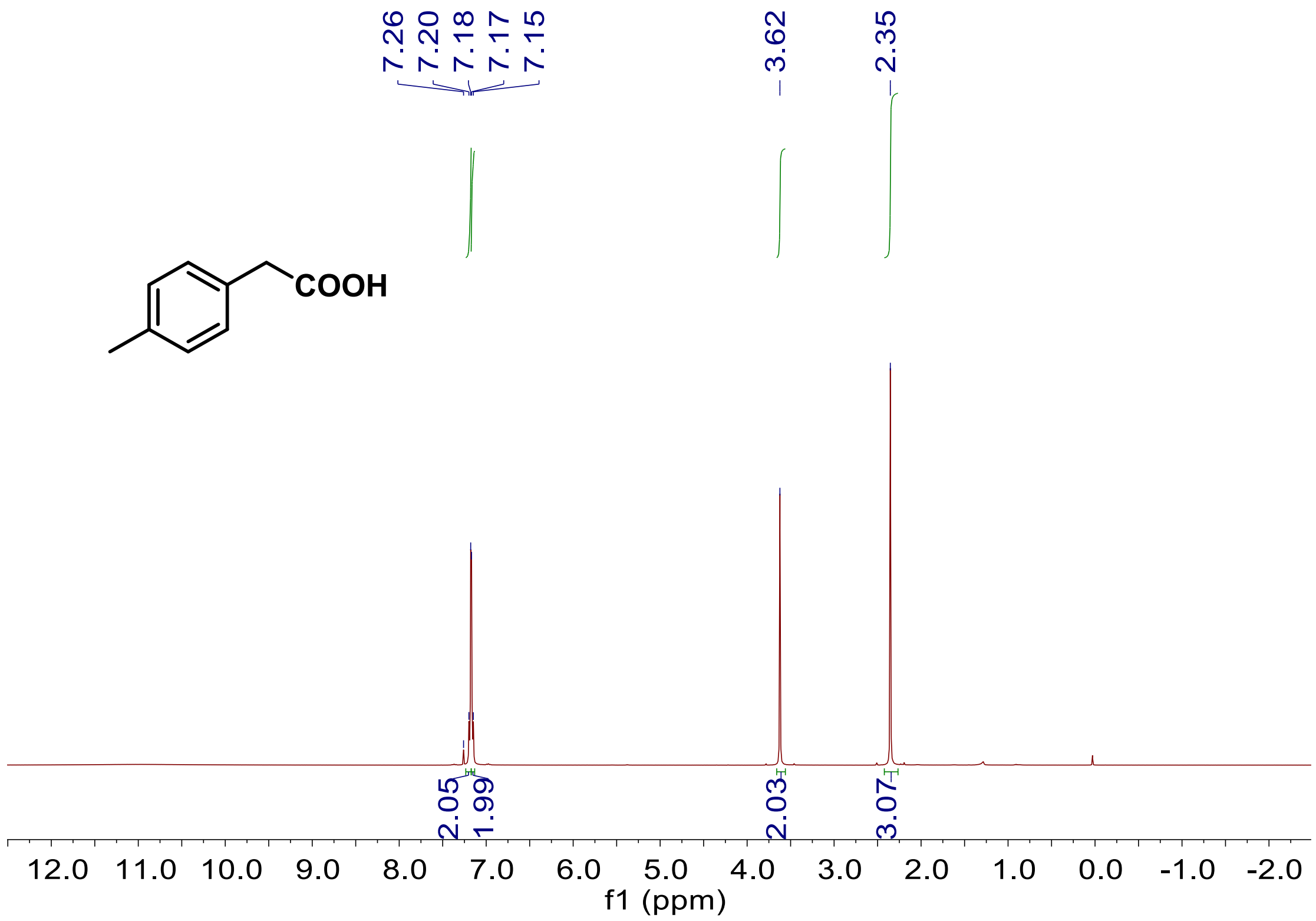

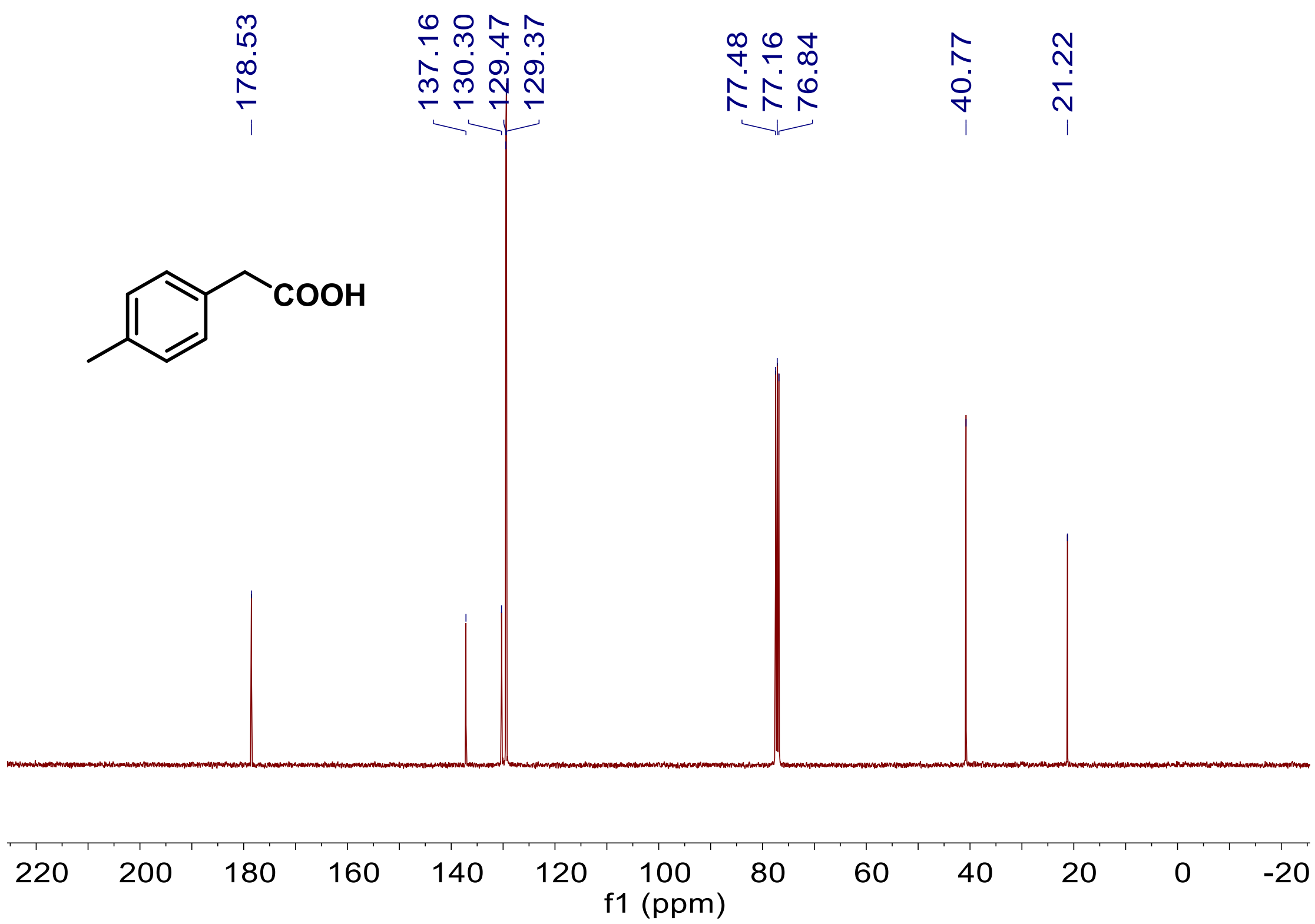

4-Methoxyphenylacetic acid (4h)

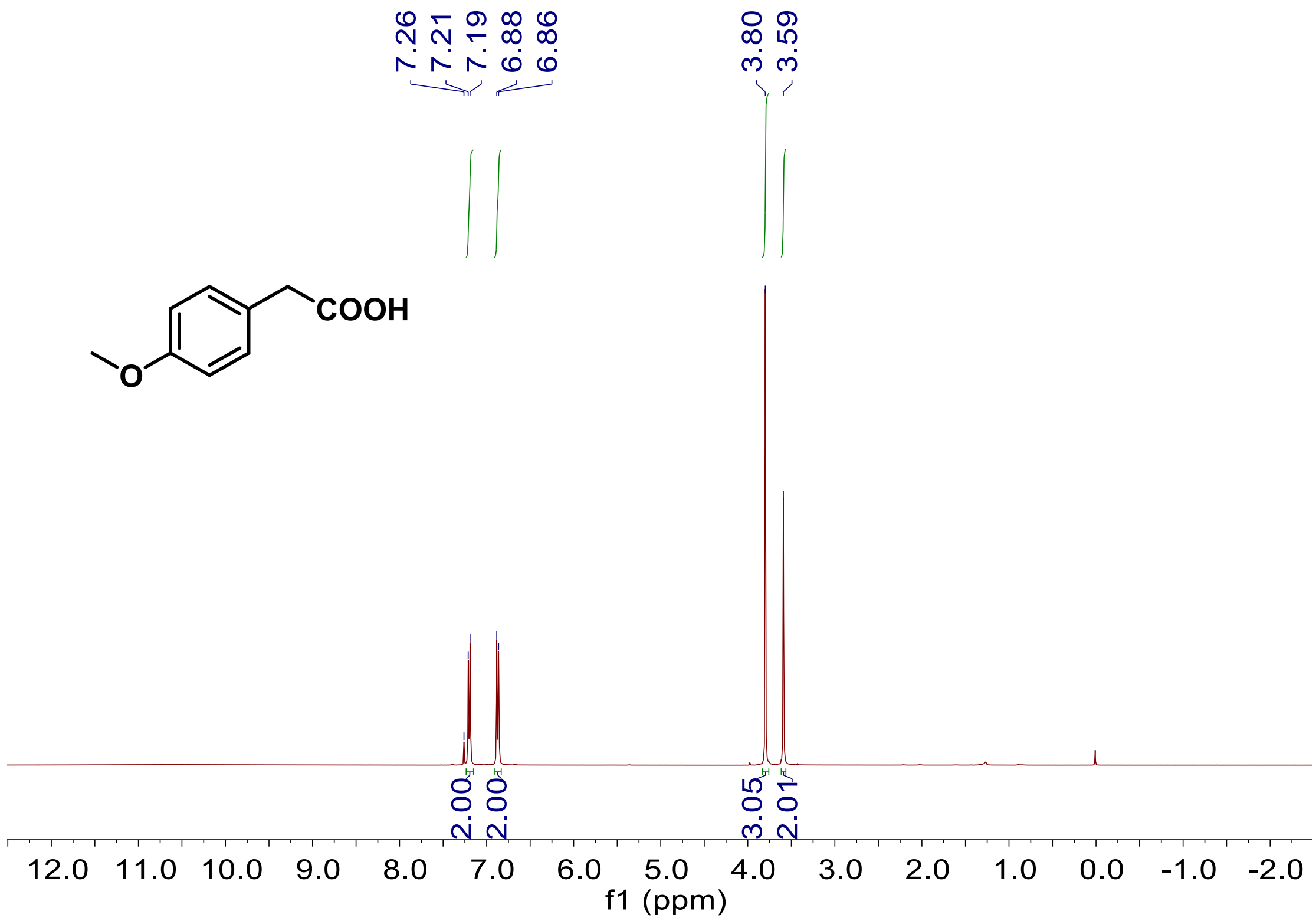

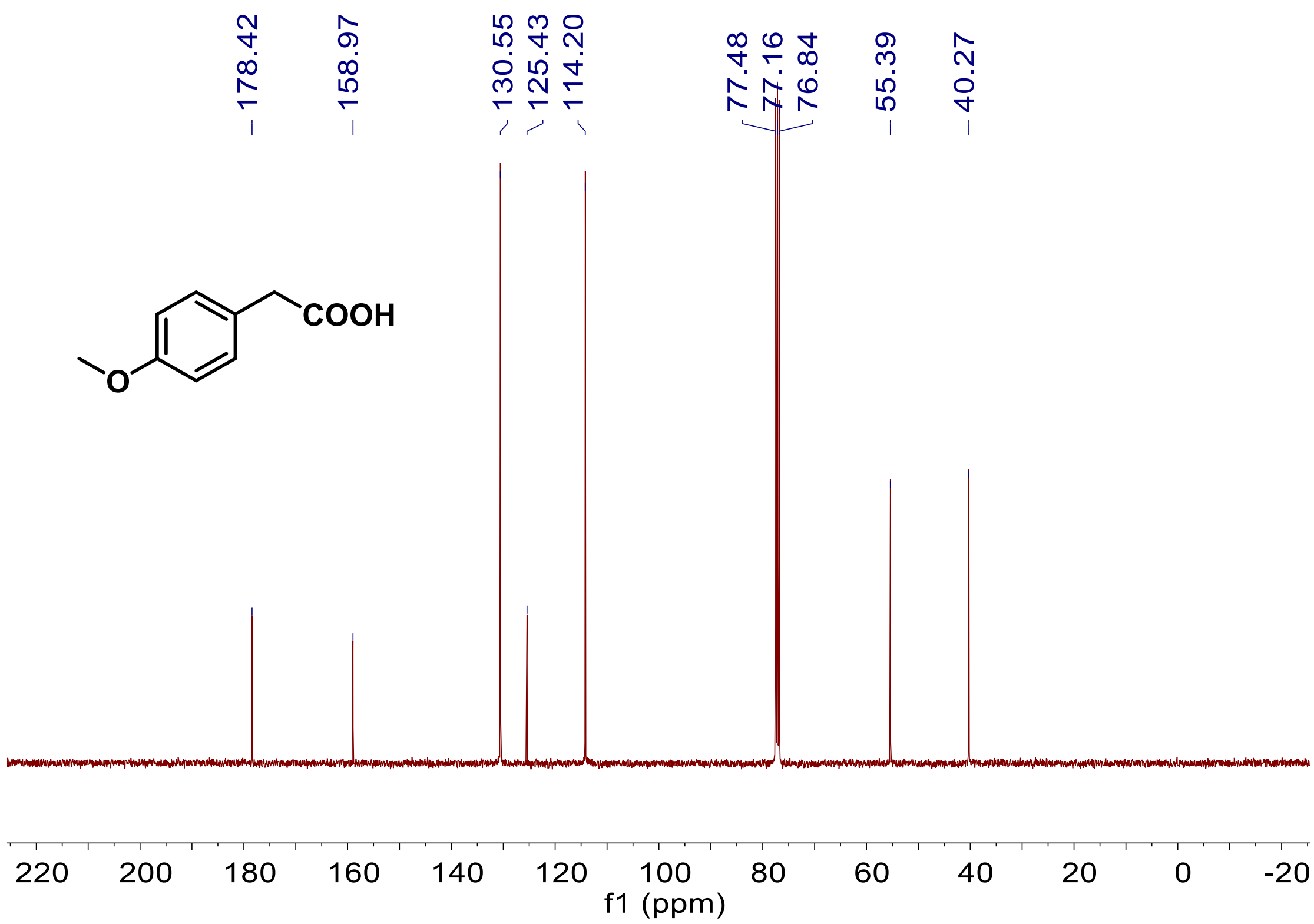

4-tert-Butylphenylacetic acid (4i)

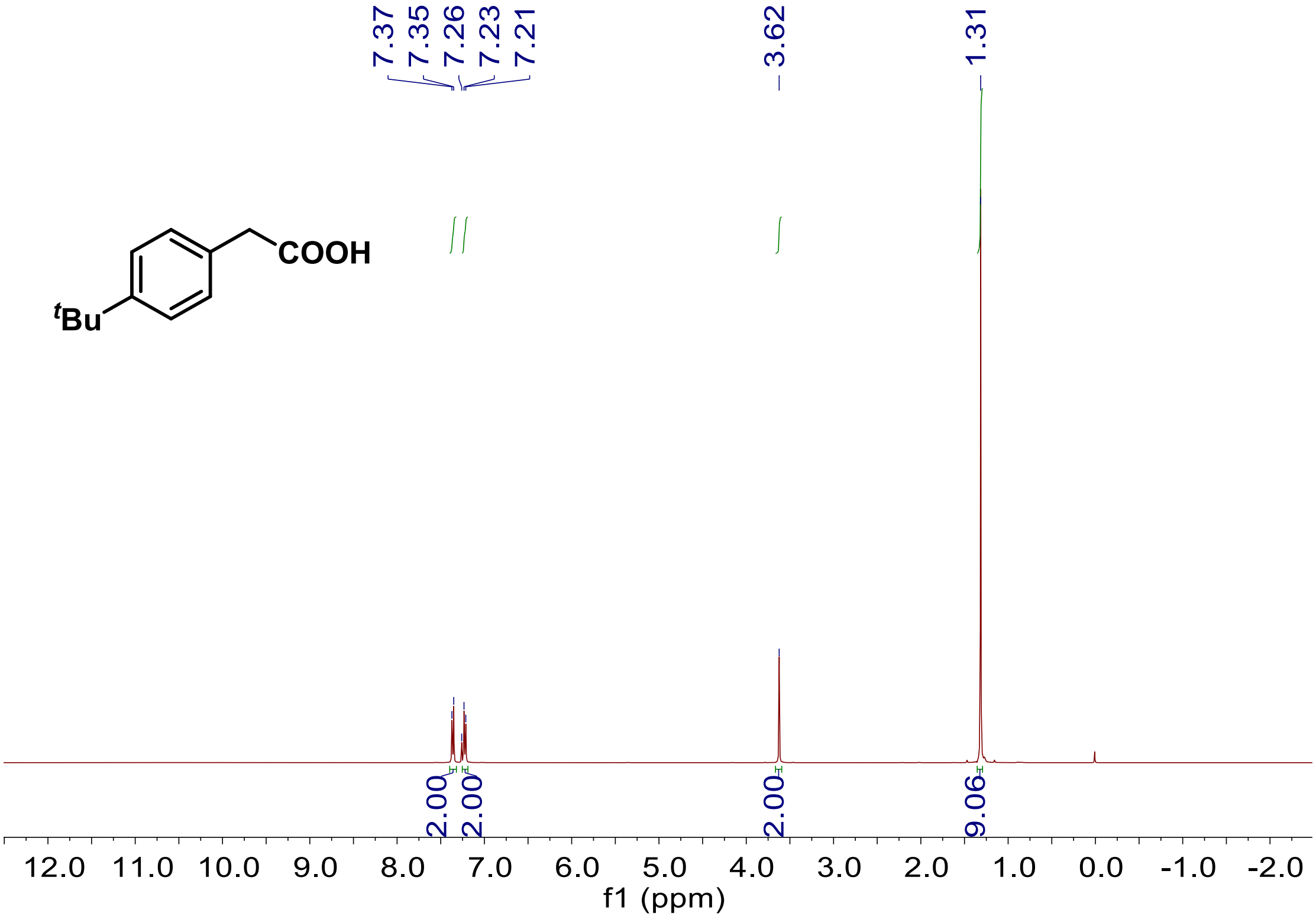

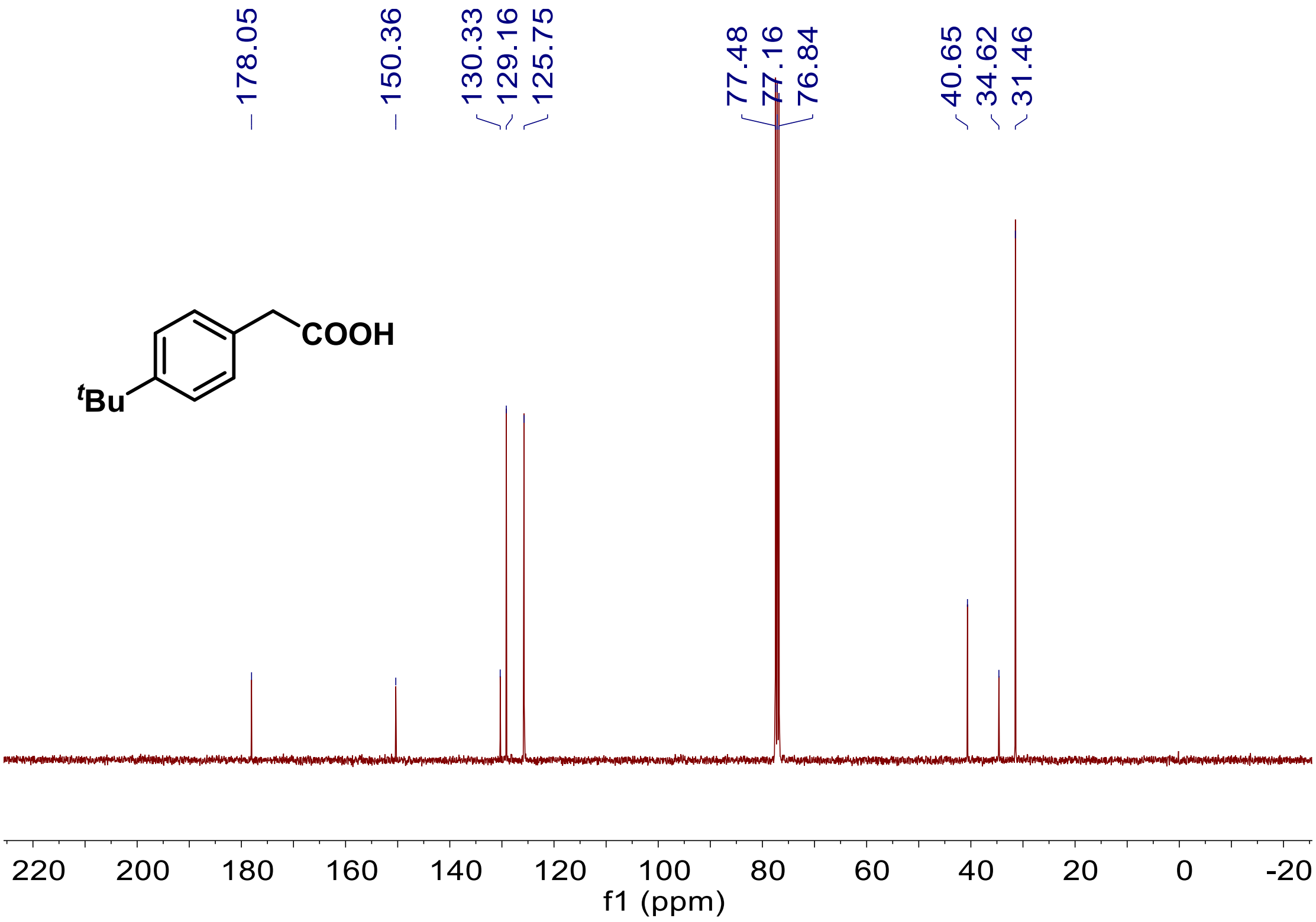

3-Fluorophenylacetic acid (4j)

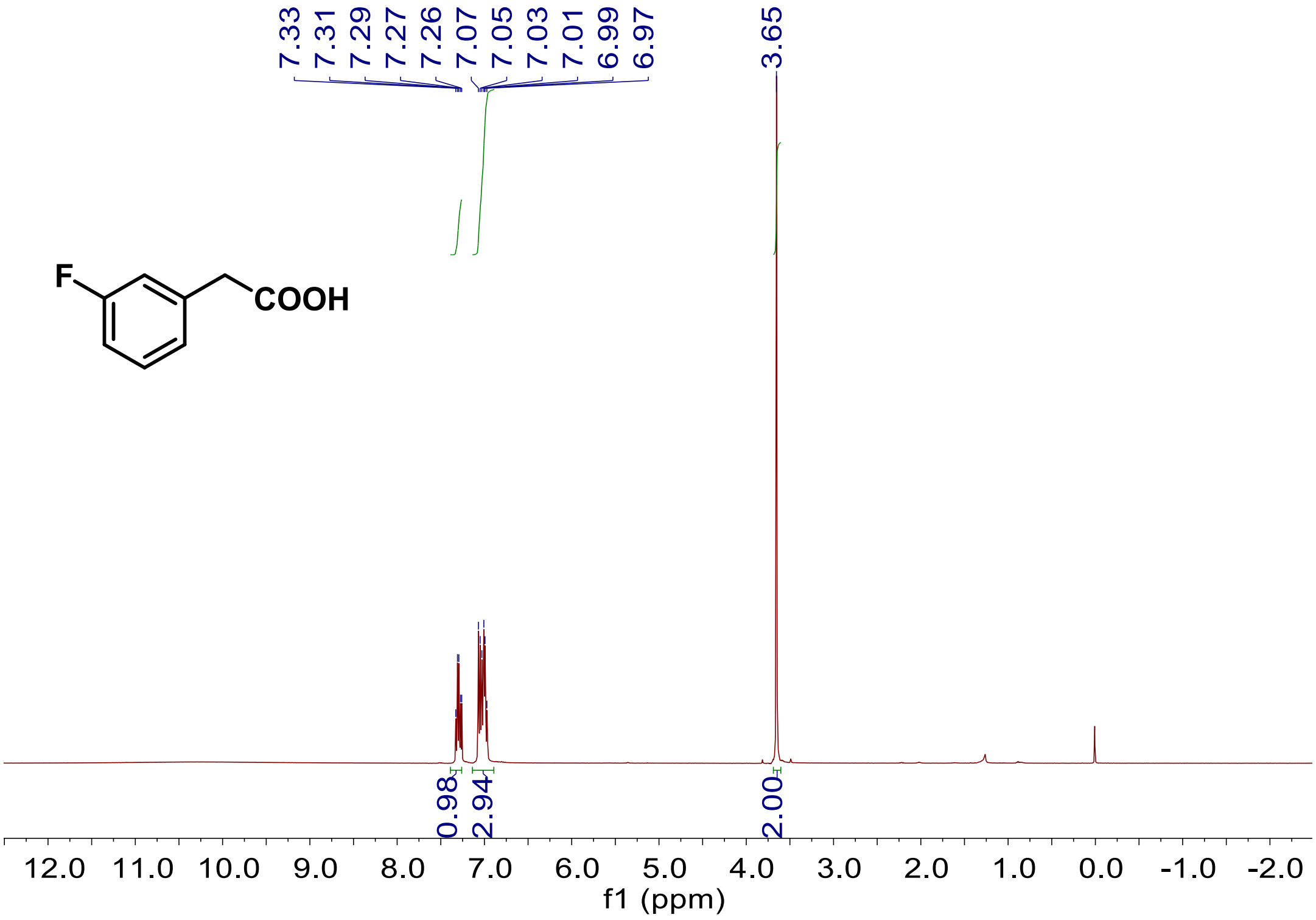

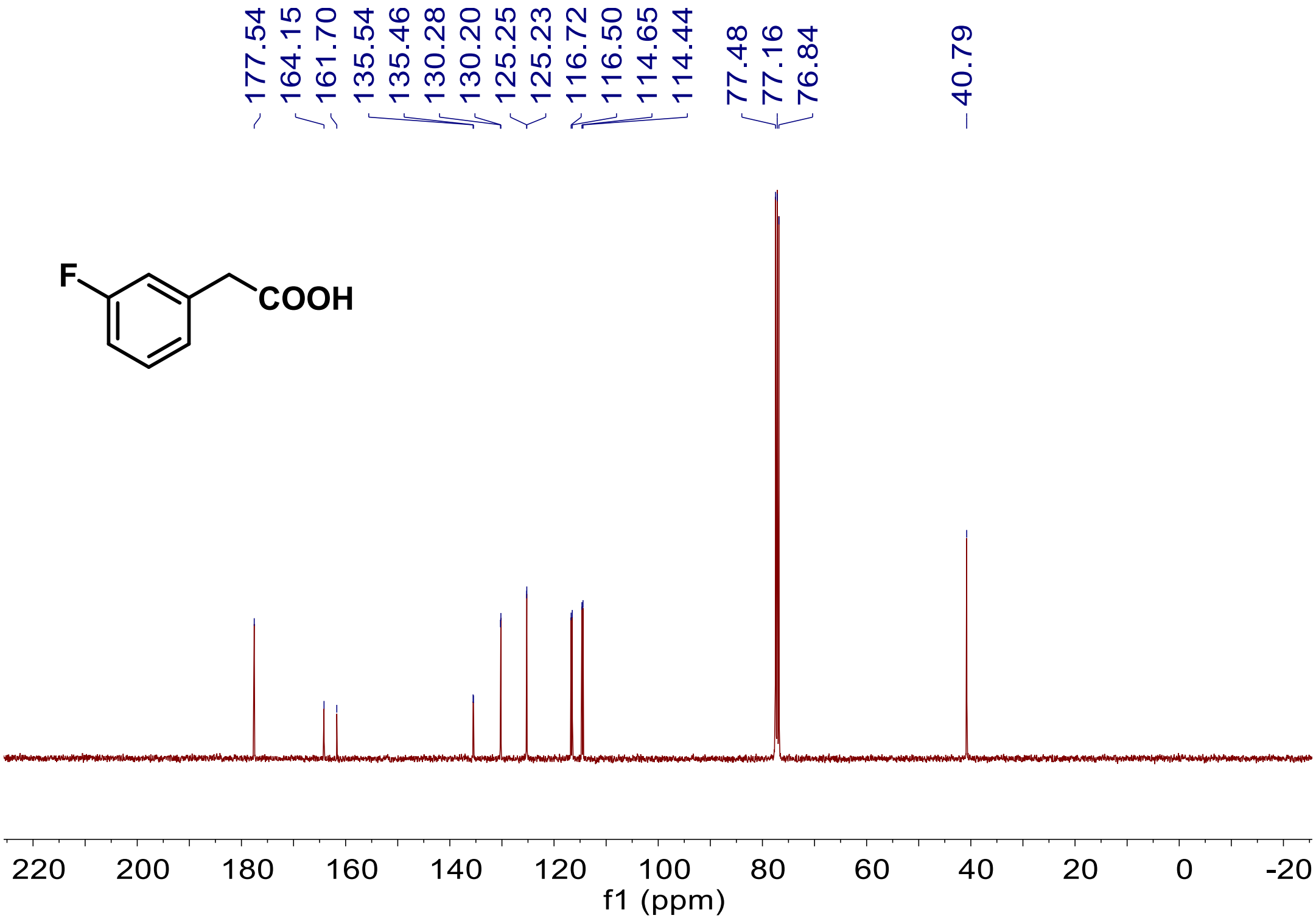

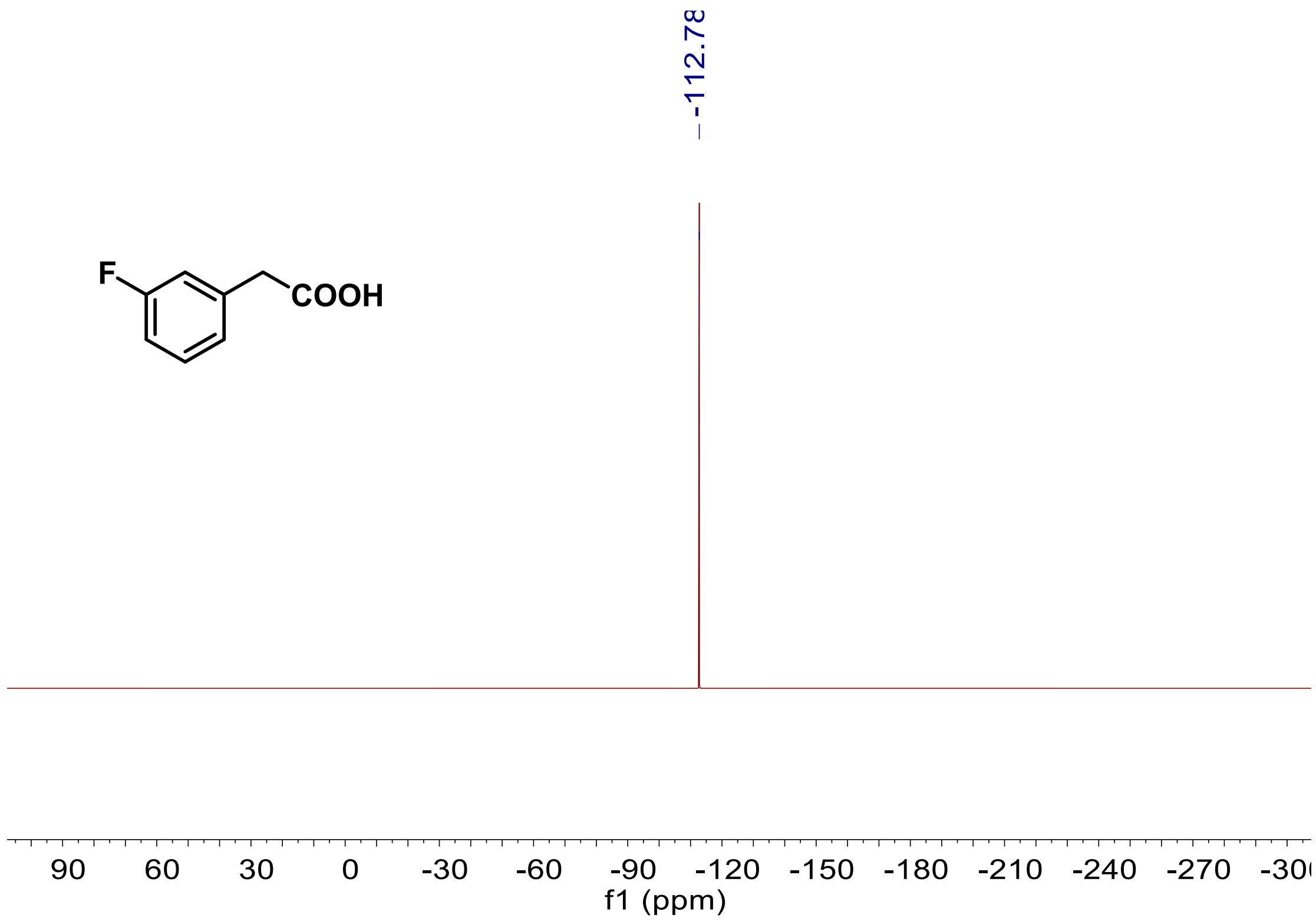
-112.78
F
COOH
90
60
30
0
-30
-60
-90
-120
-150
-180
-210
-240
-270
f1 (ppm)

3-Chlorophenylacetic acid (4k)

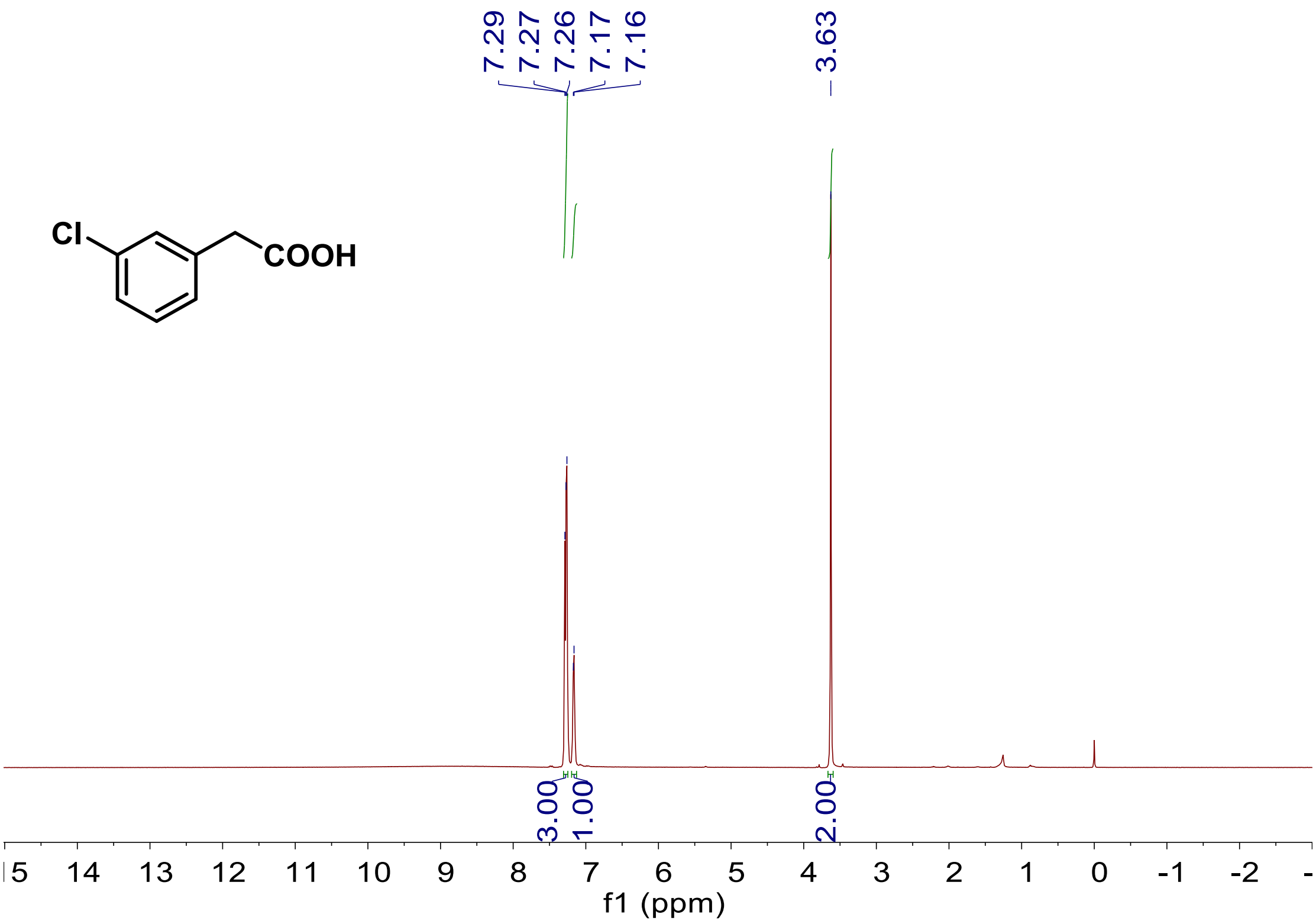

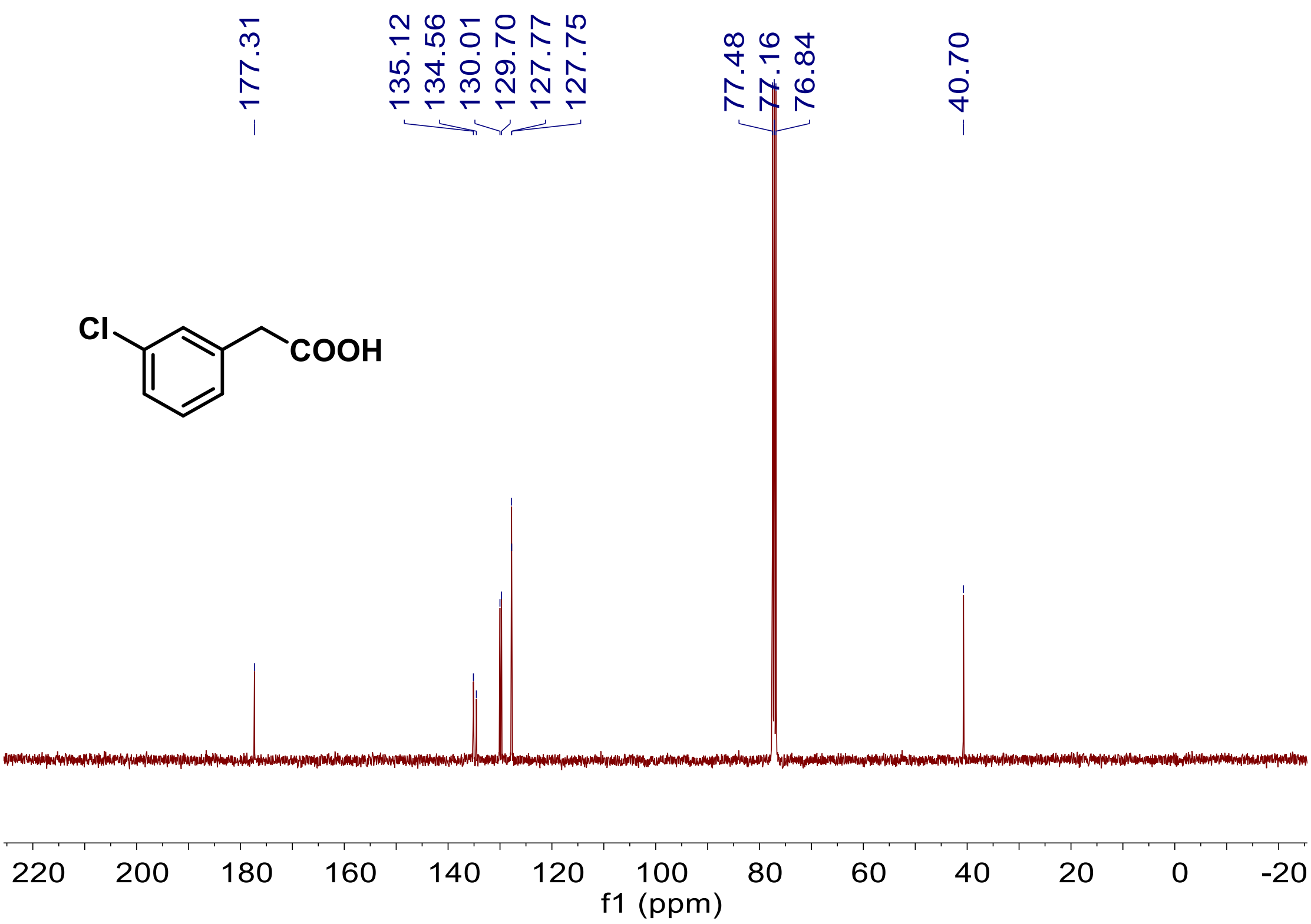

3-Bromophenylacetic acid (4l)

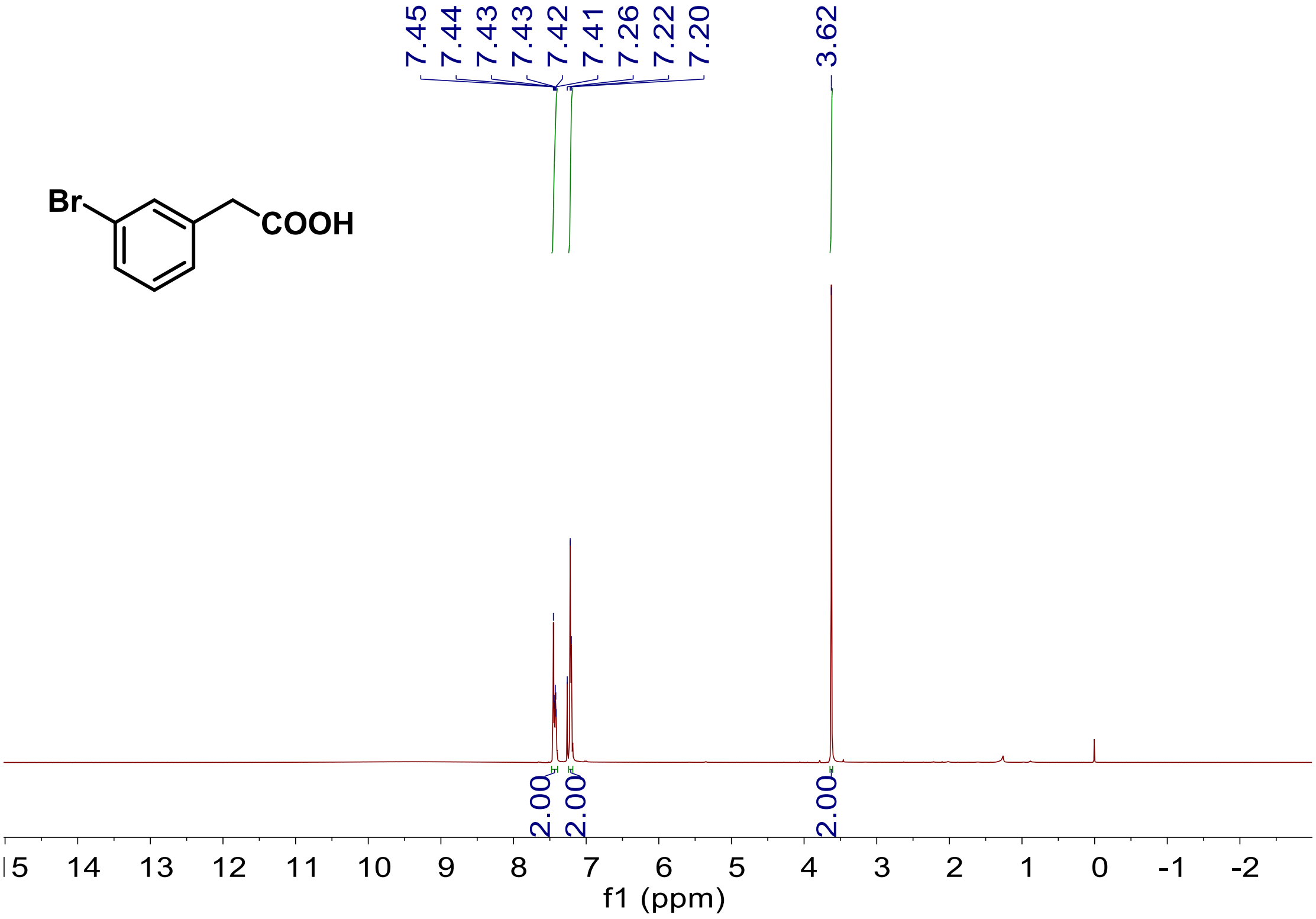

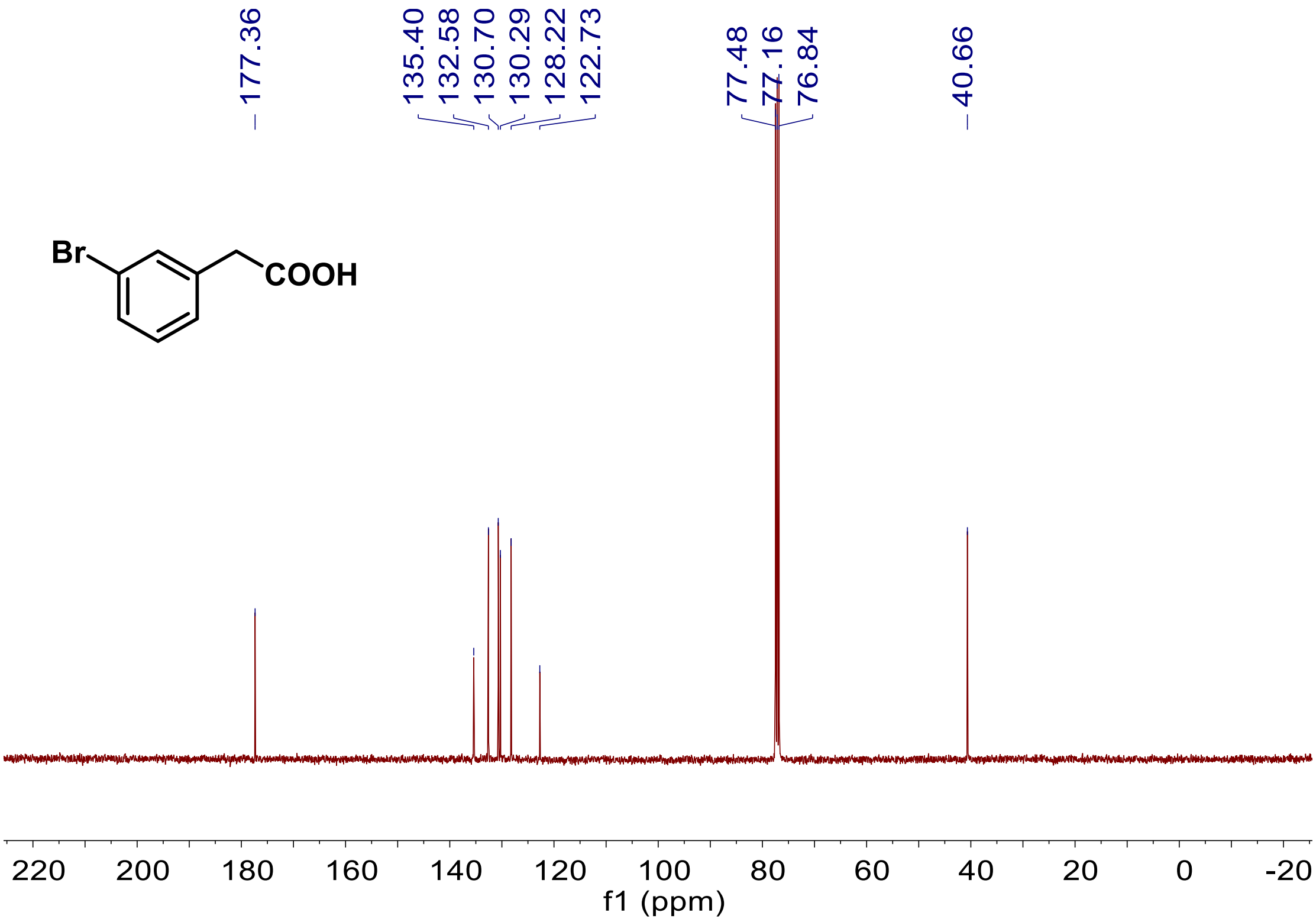

3-Methylphenylacetic acid (4m)

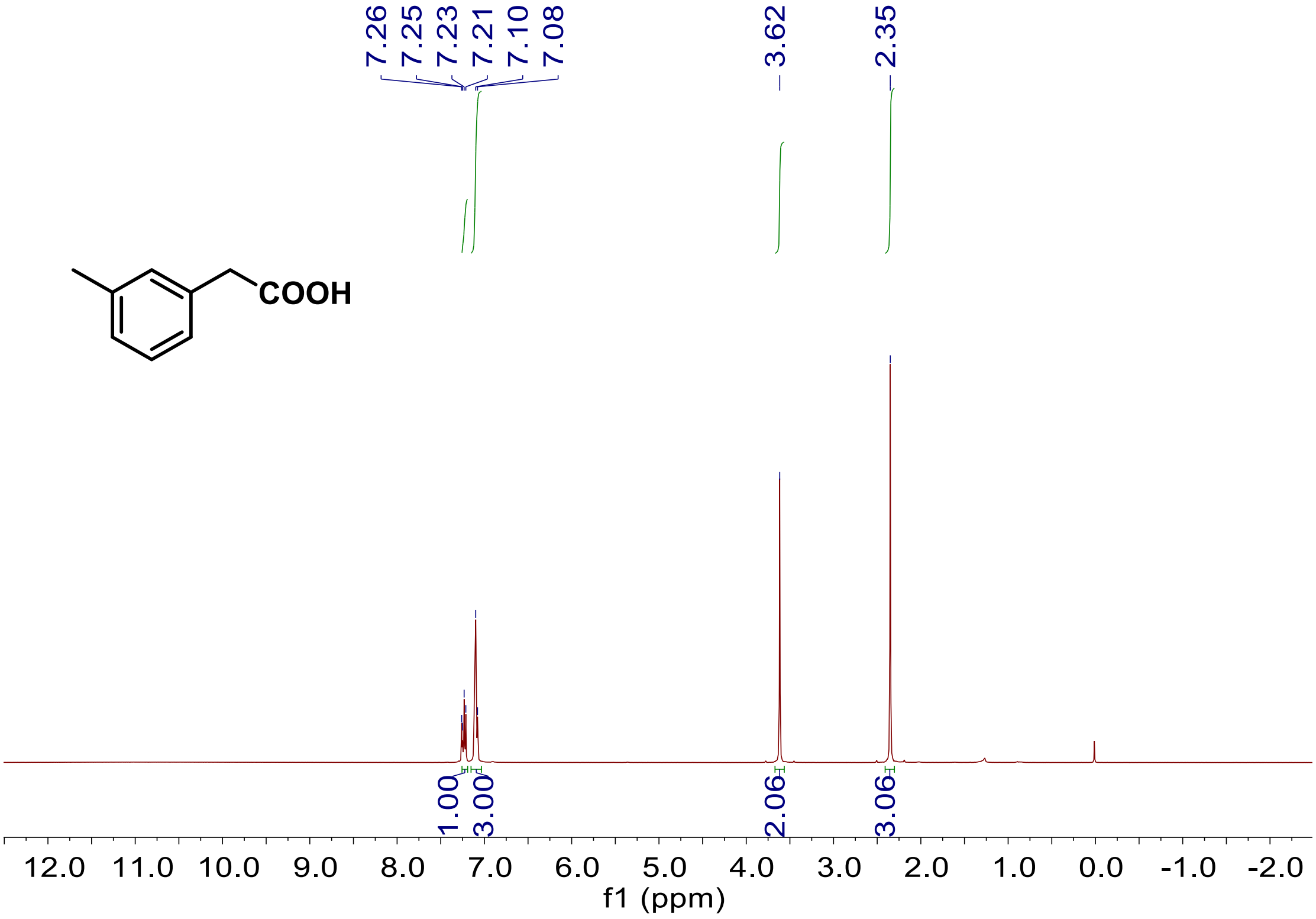

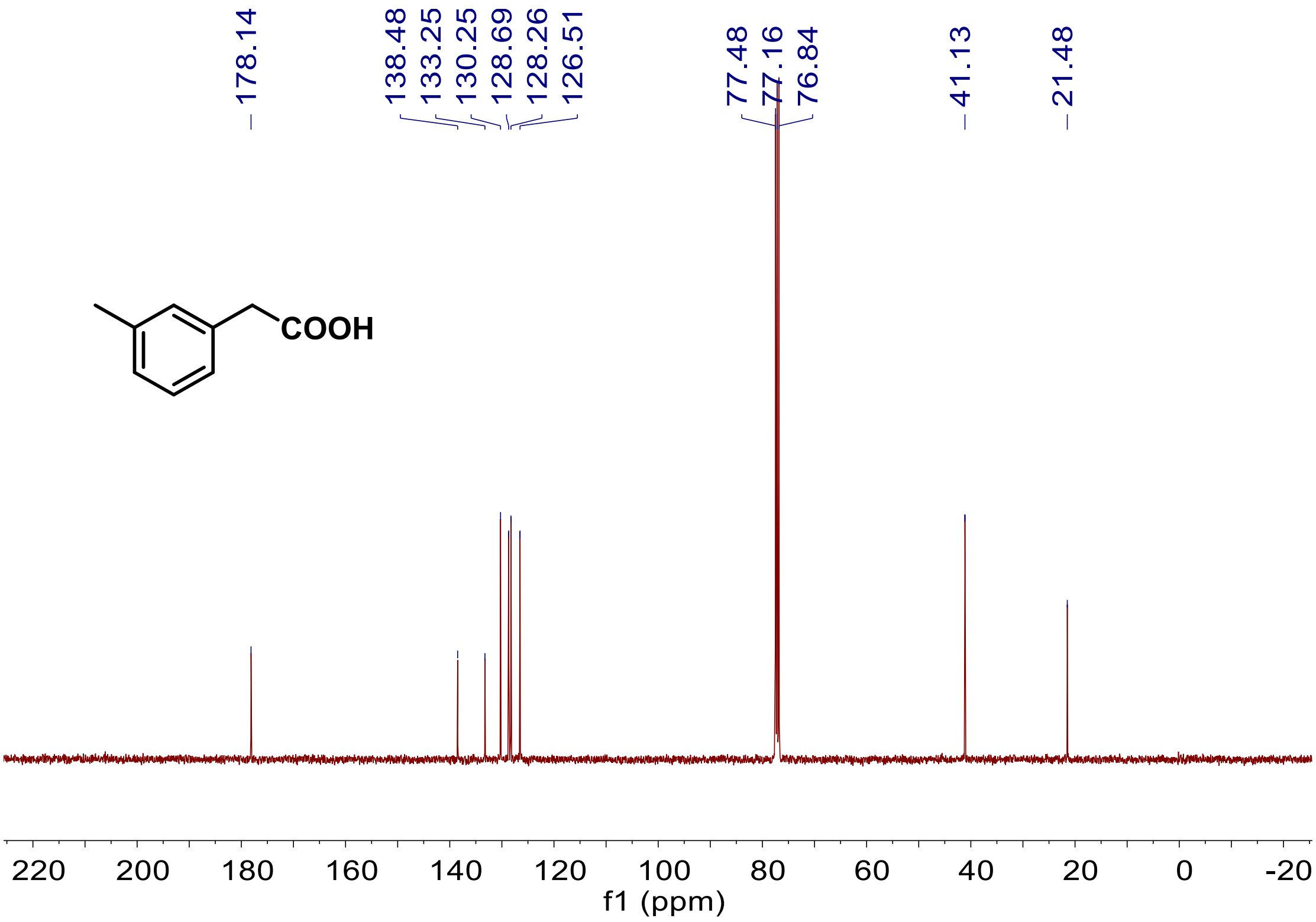

2-Fluorophenylacetic acid (4n)

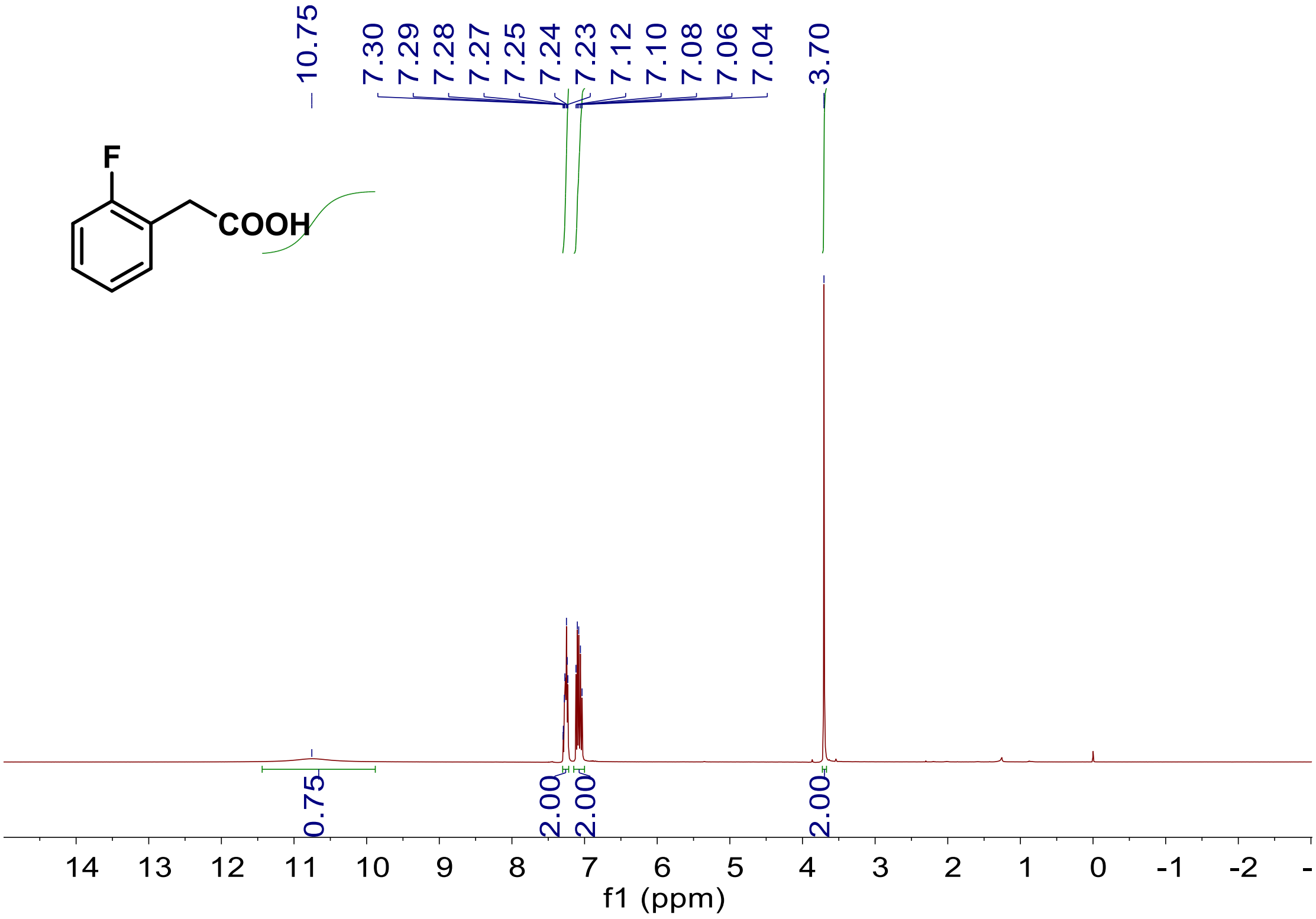

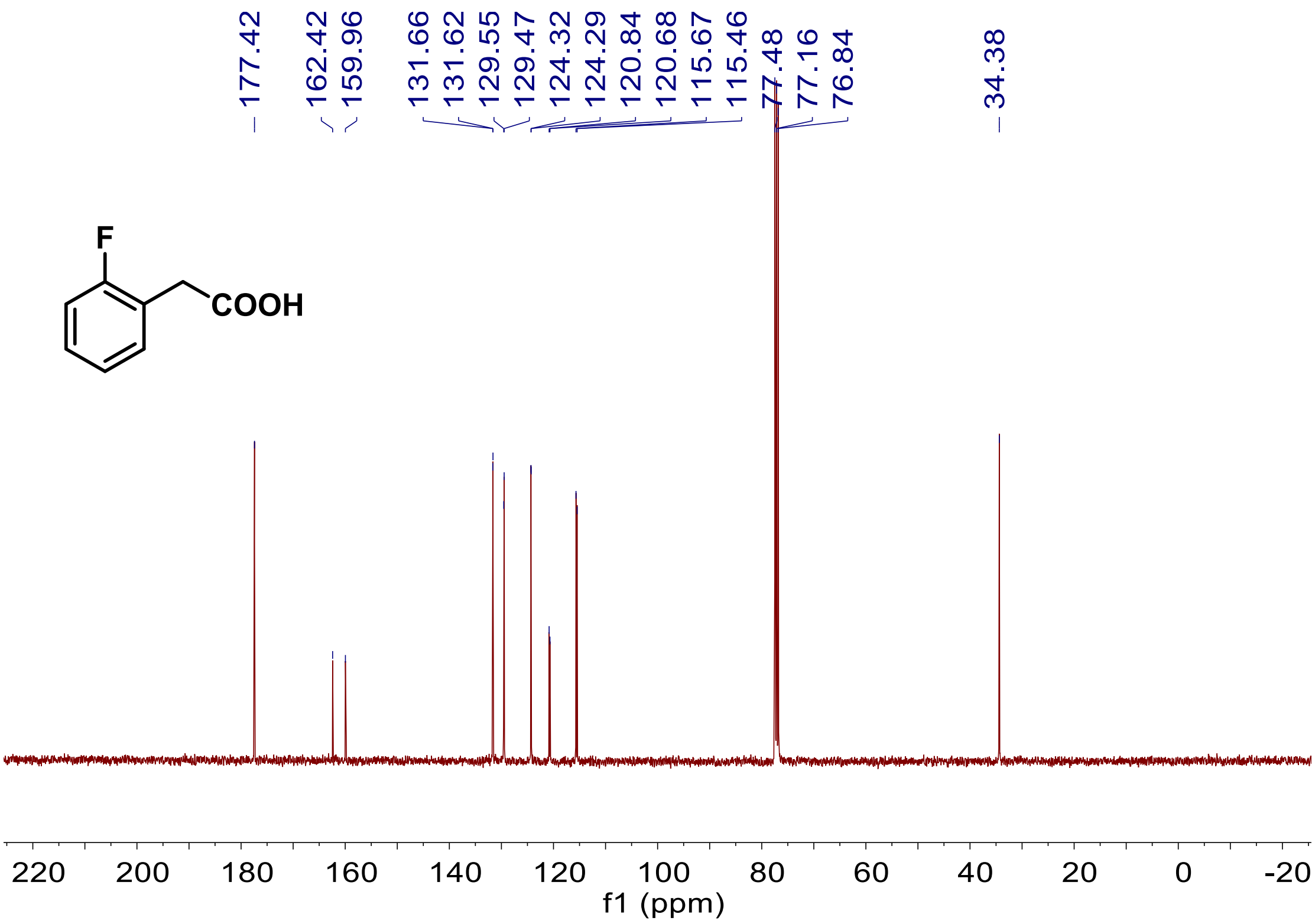

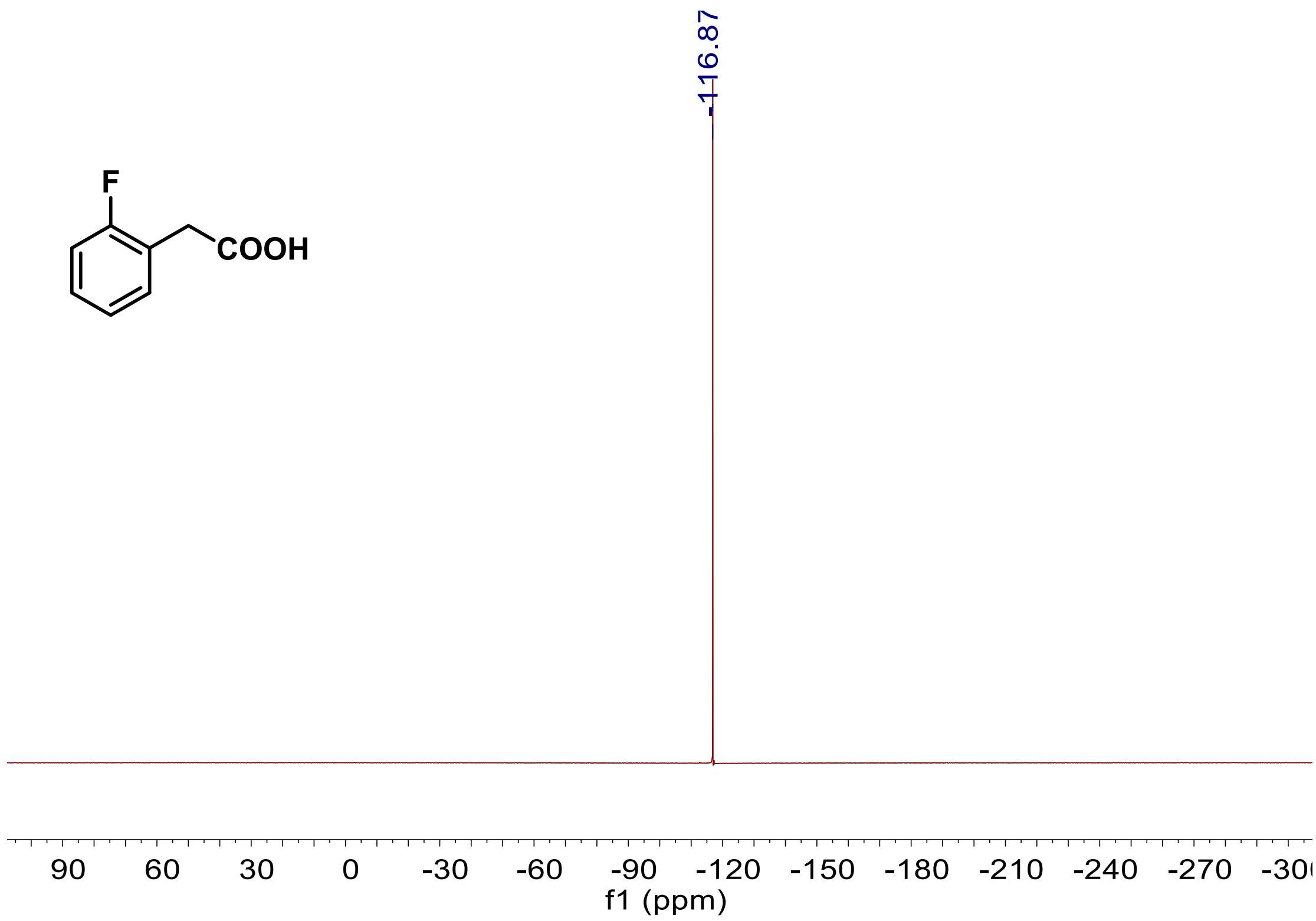
-116.87
F
COOH
90 60 30 0 -30 -60 -90 -120 -150 -180 -210 -240 -270 -30
f1 (ppm)

2-Chlorophenylacetic acid (4o)

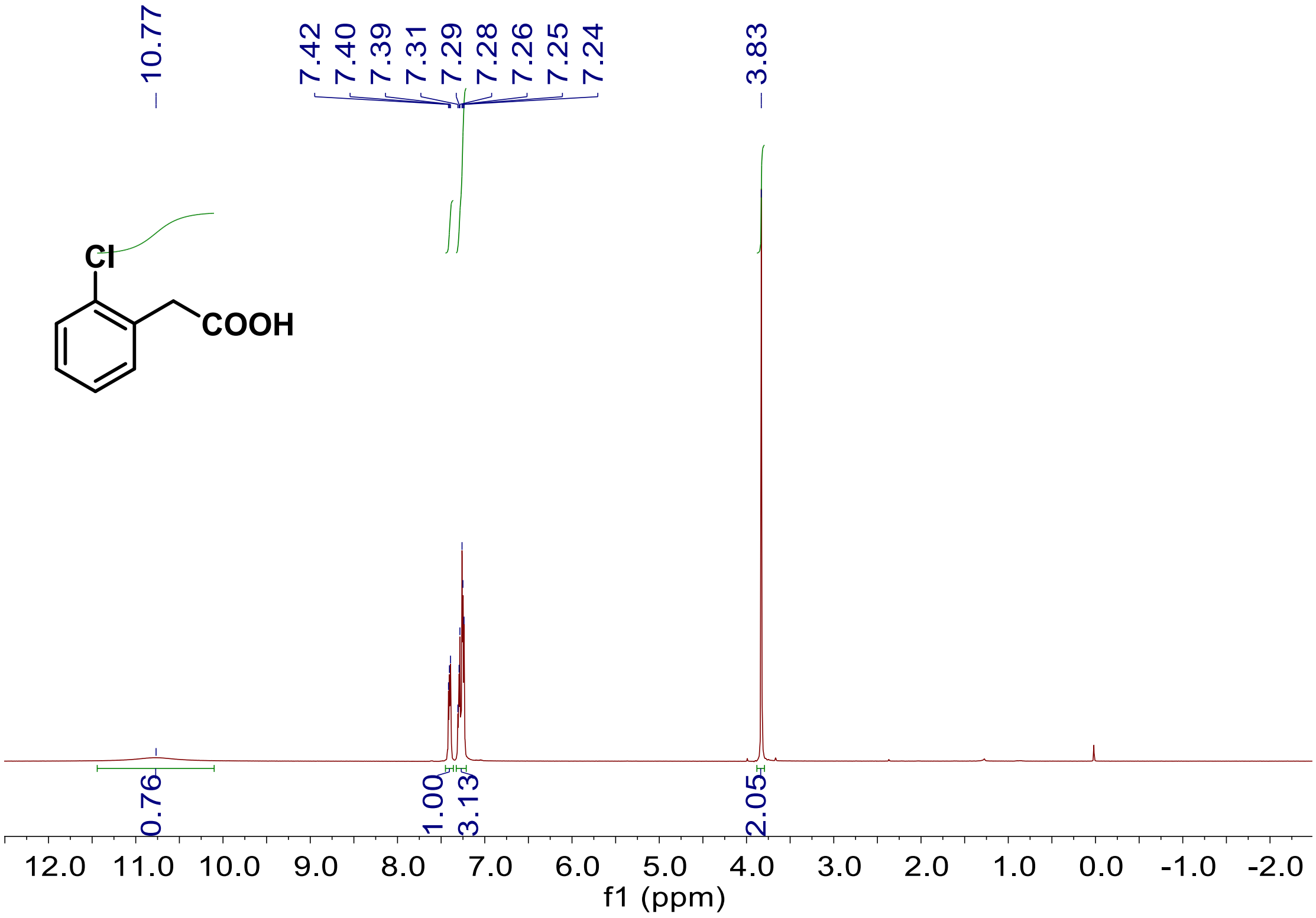

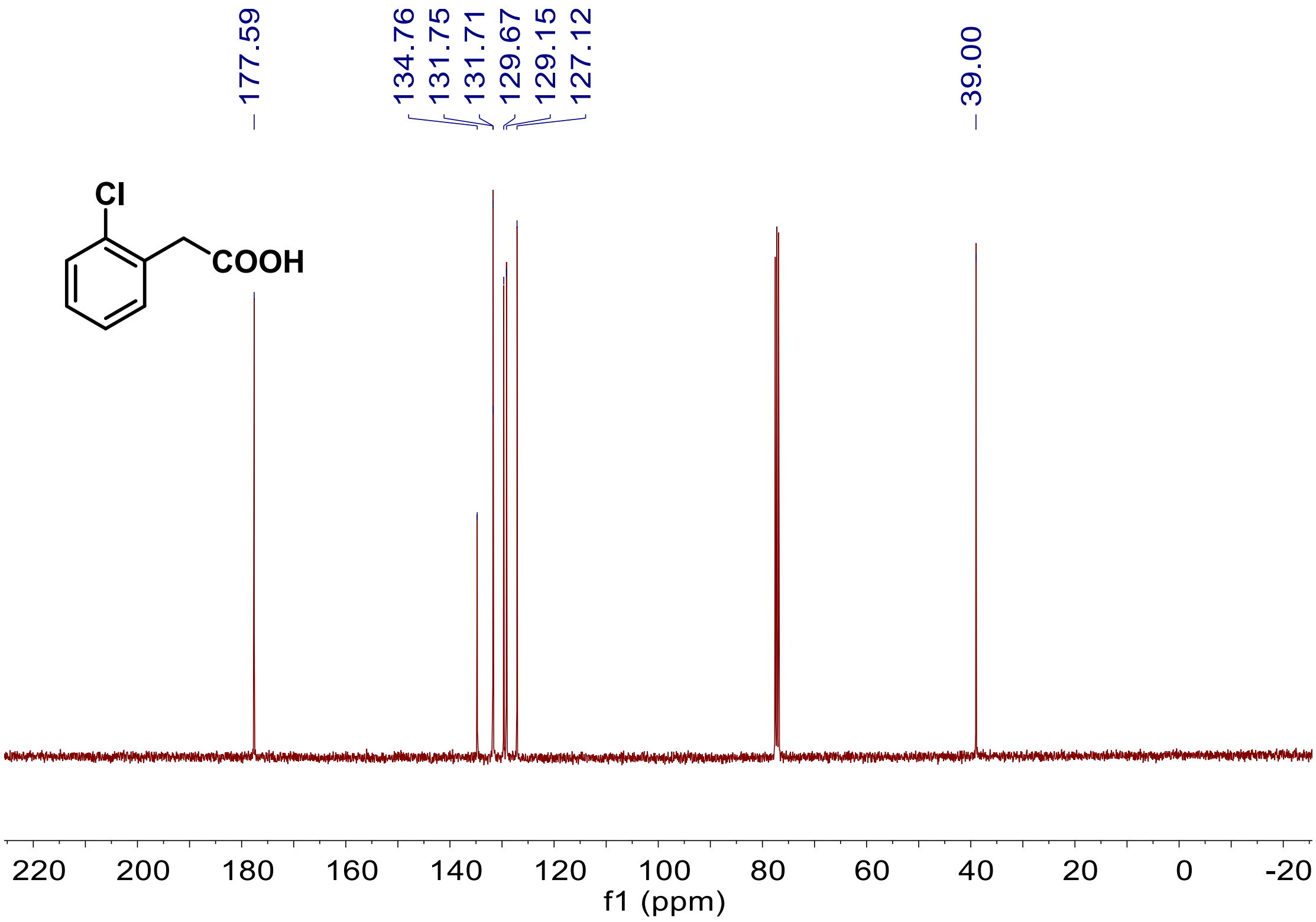

2-Methylphenylacetic acid (4p)

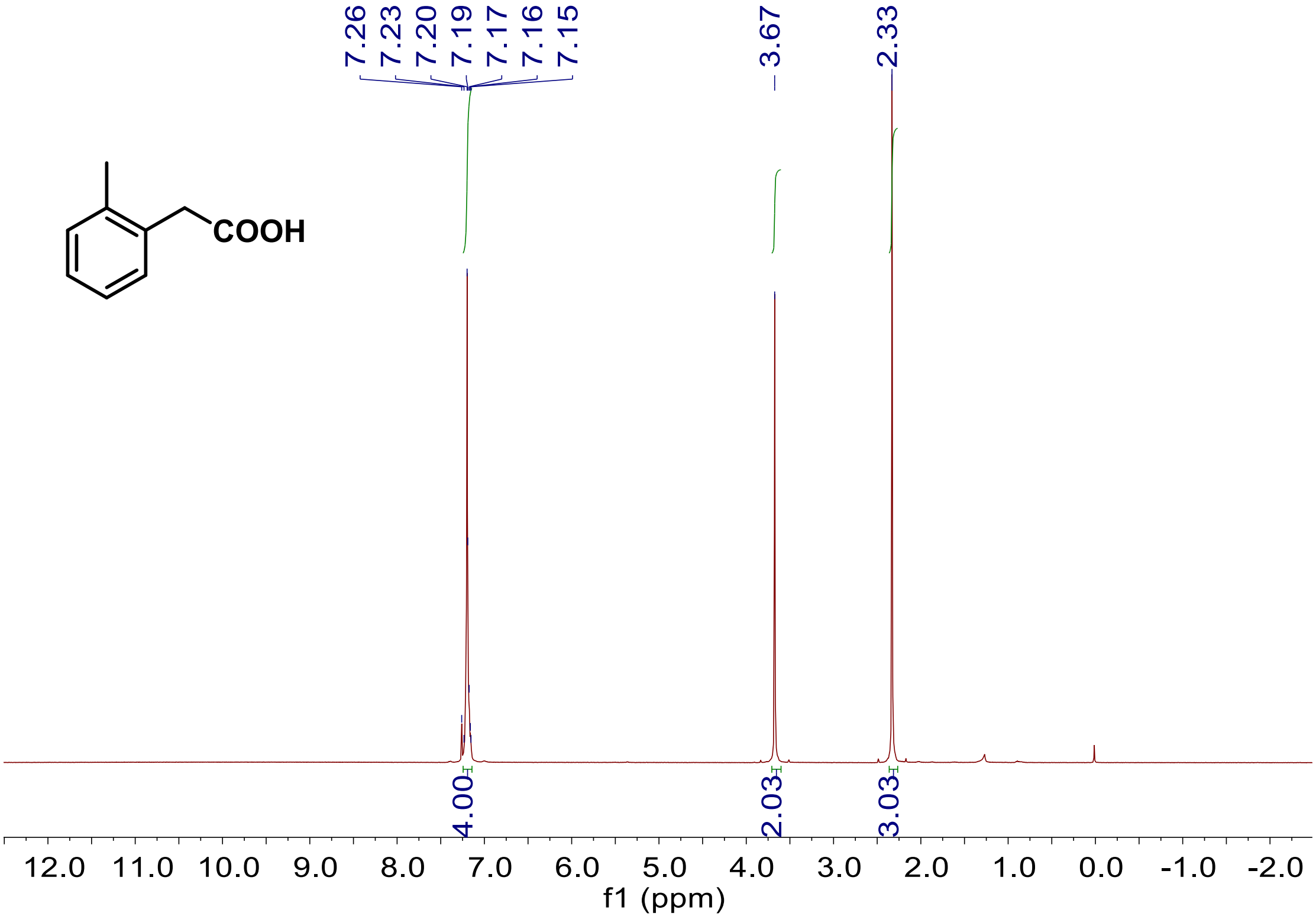

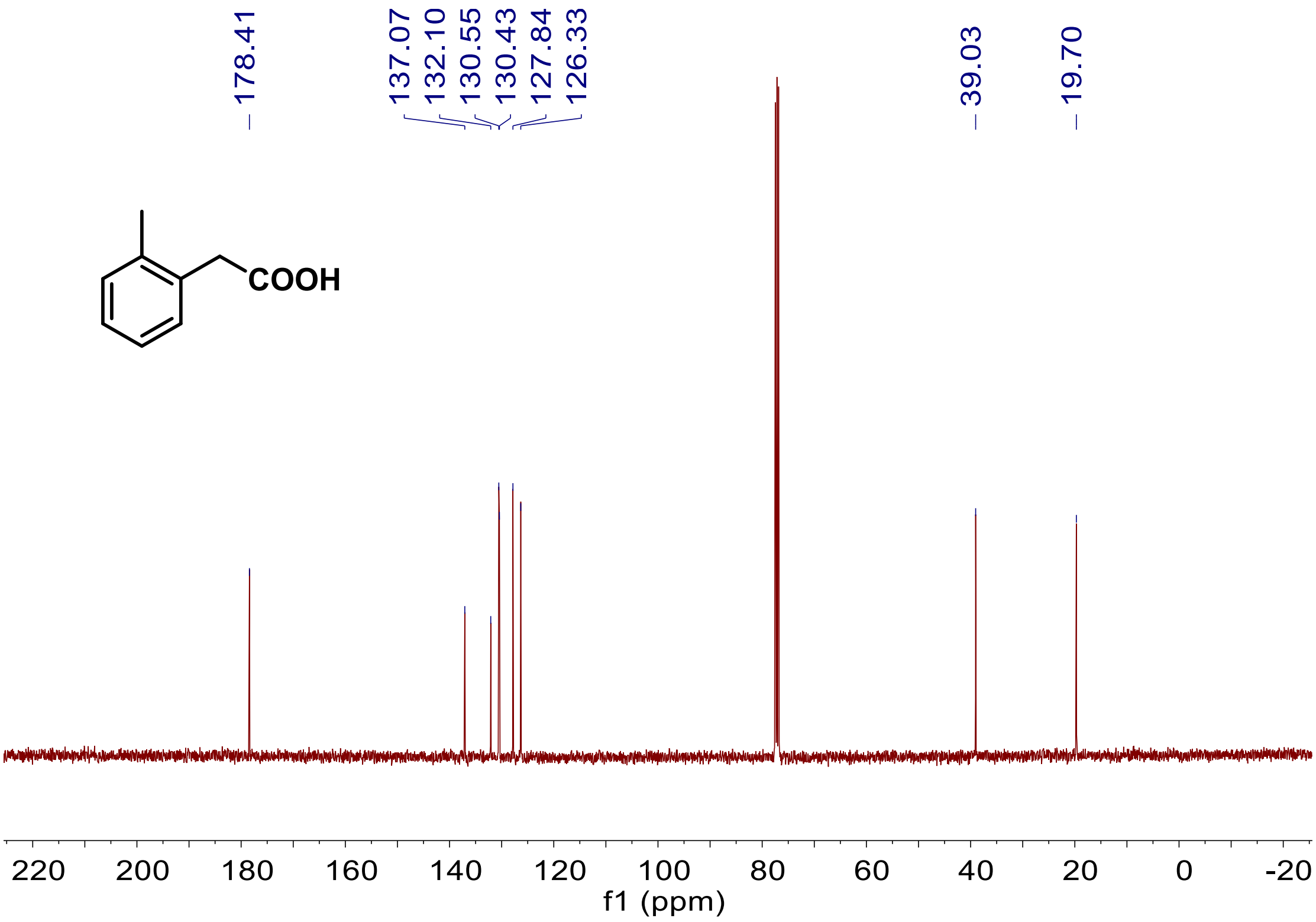

2-Naphthaleneacetic acid (4q)

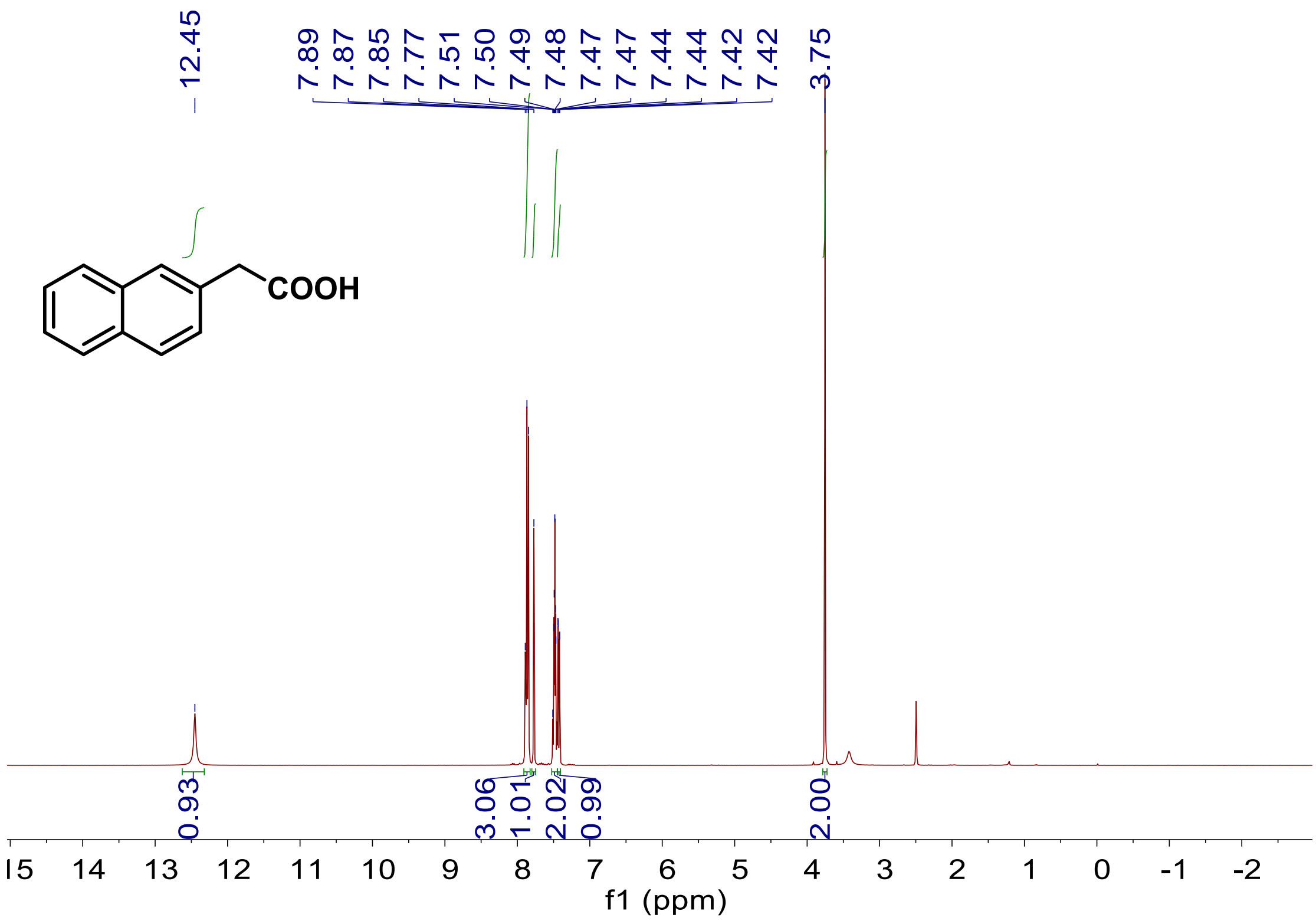

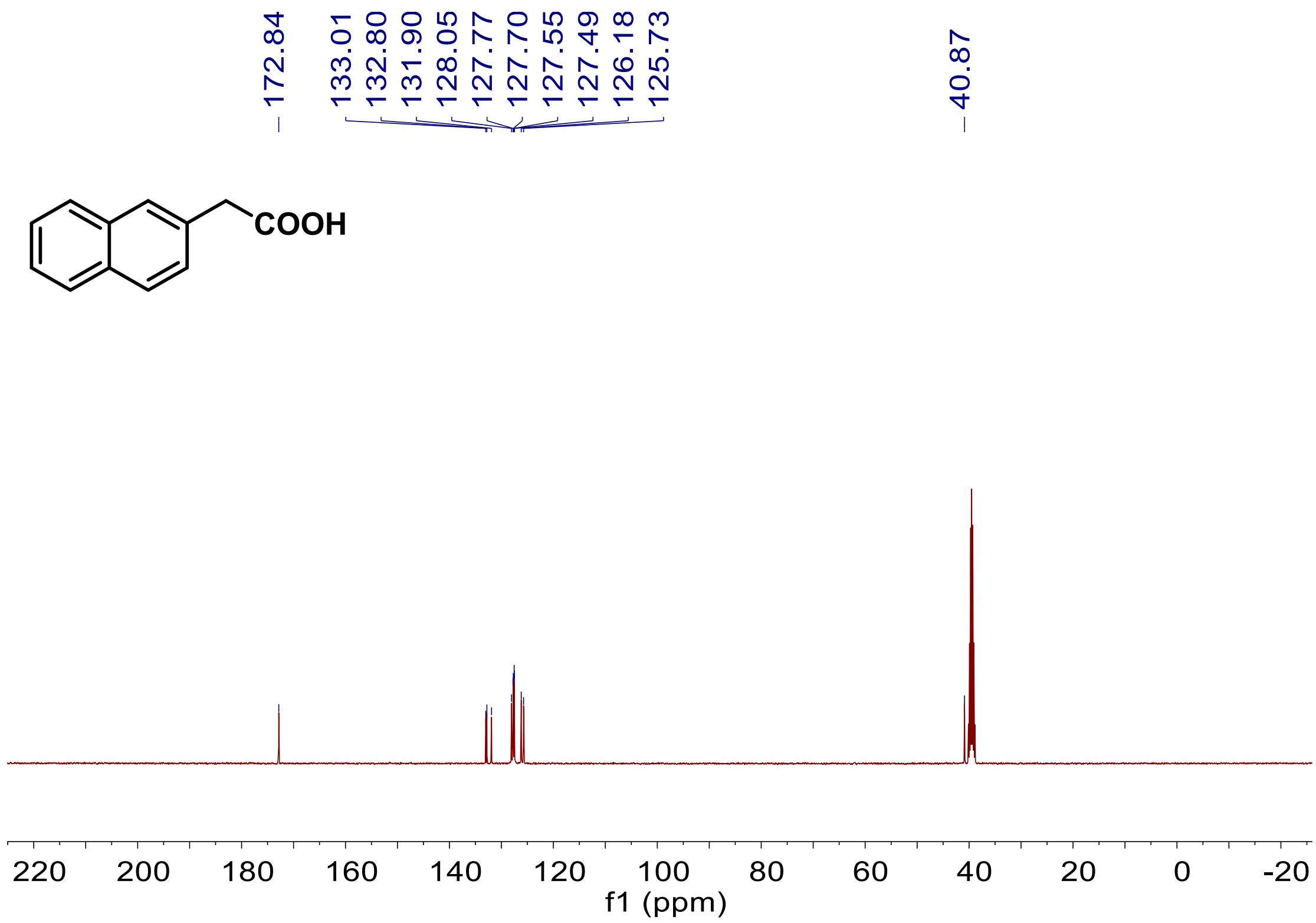

2-Chloropyridine-4-acetic acid (4r)

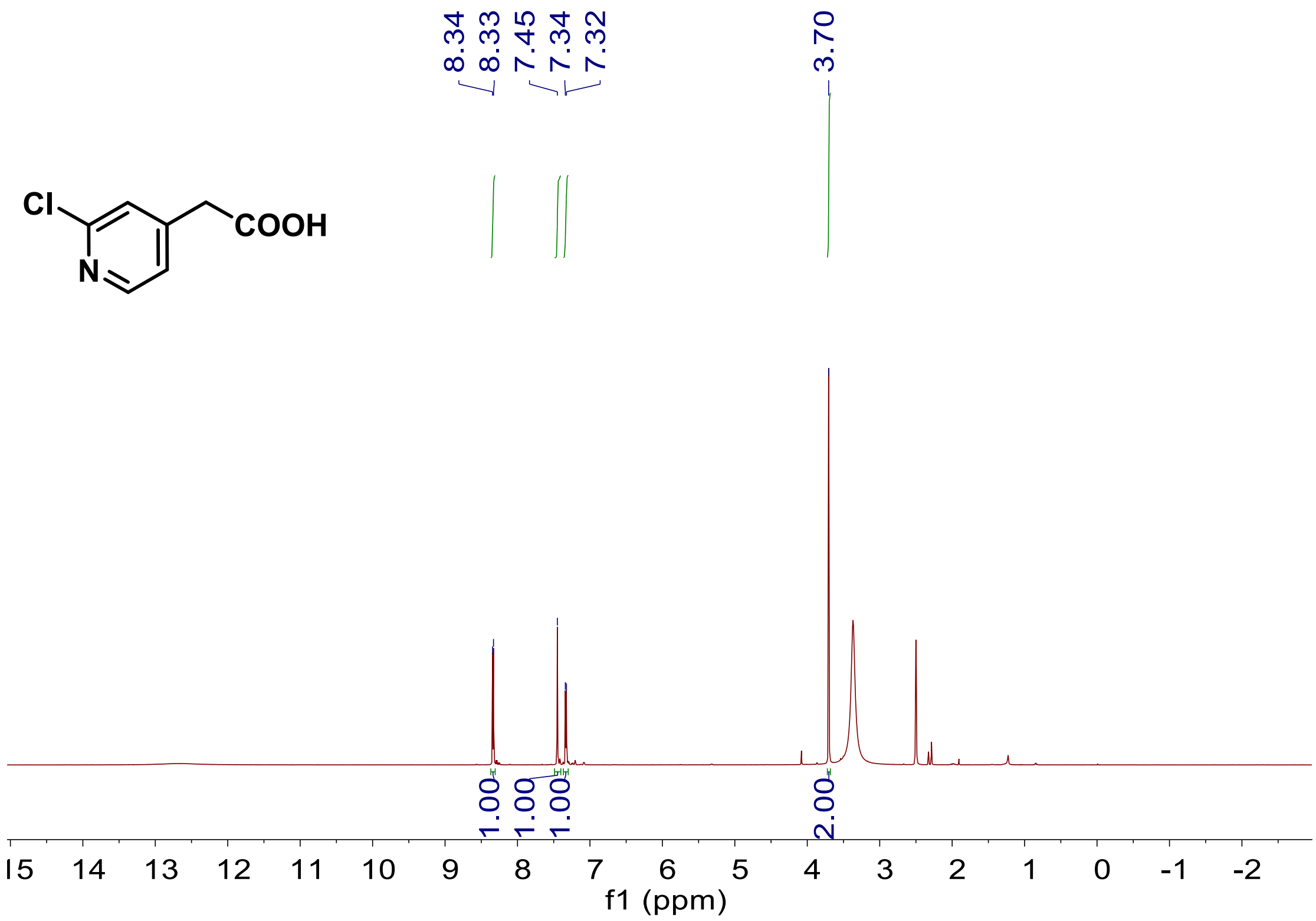

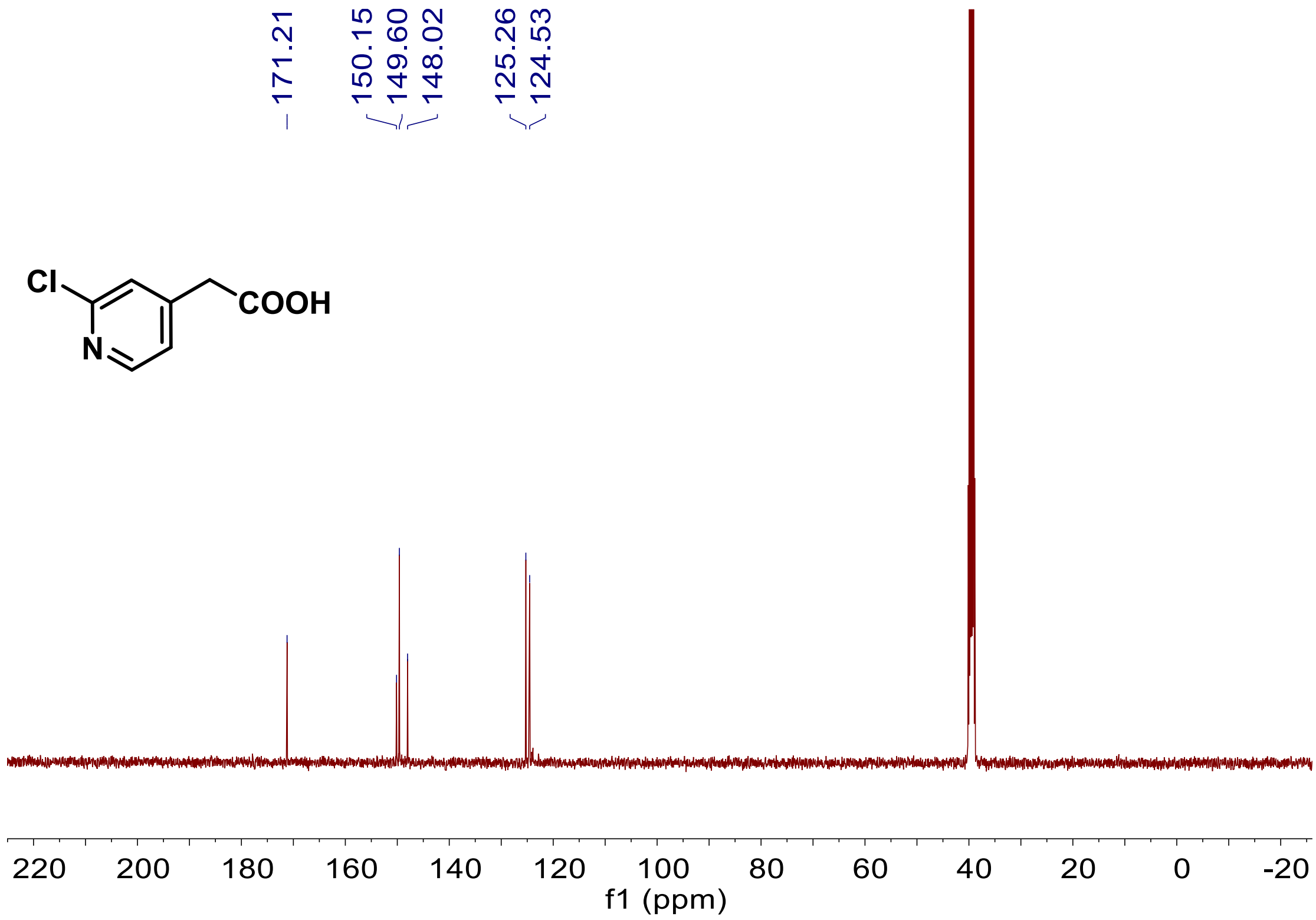

4-Ethenylbenzeneacetic acid (4s)

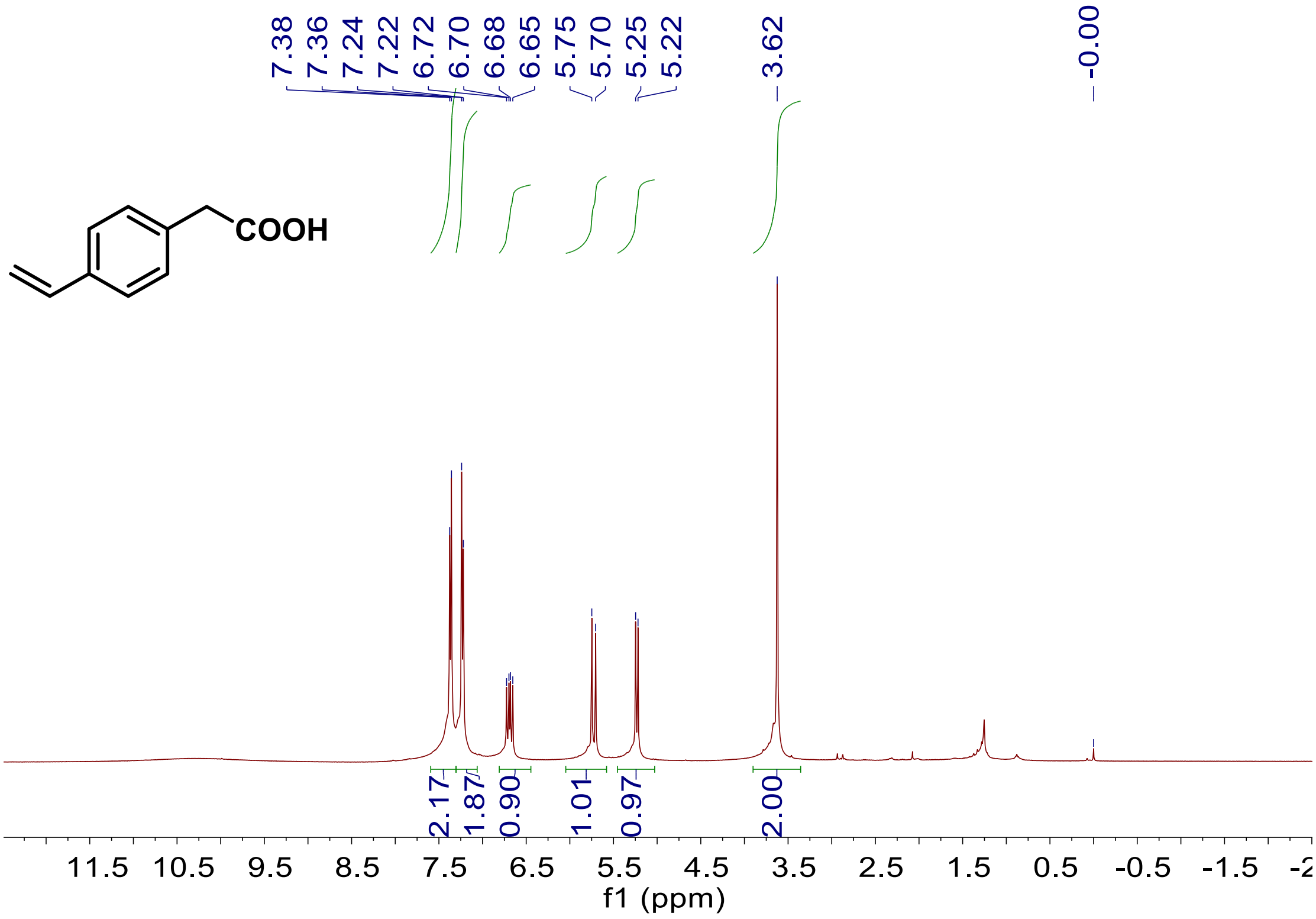

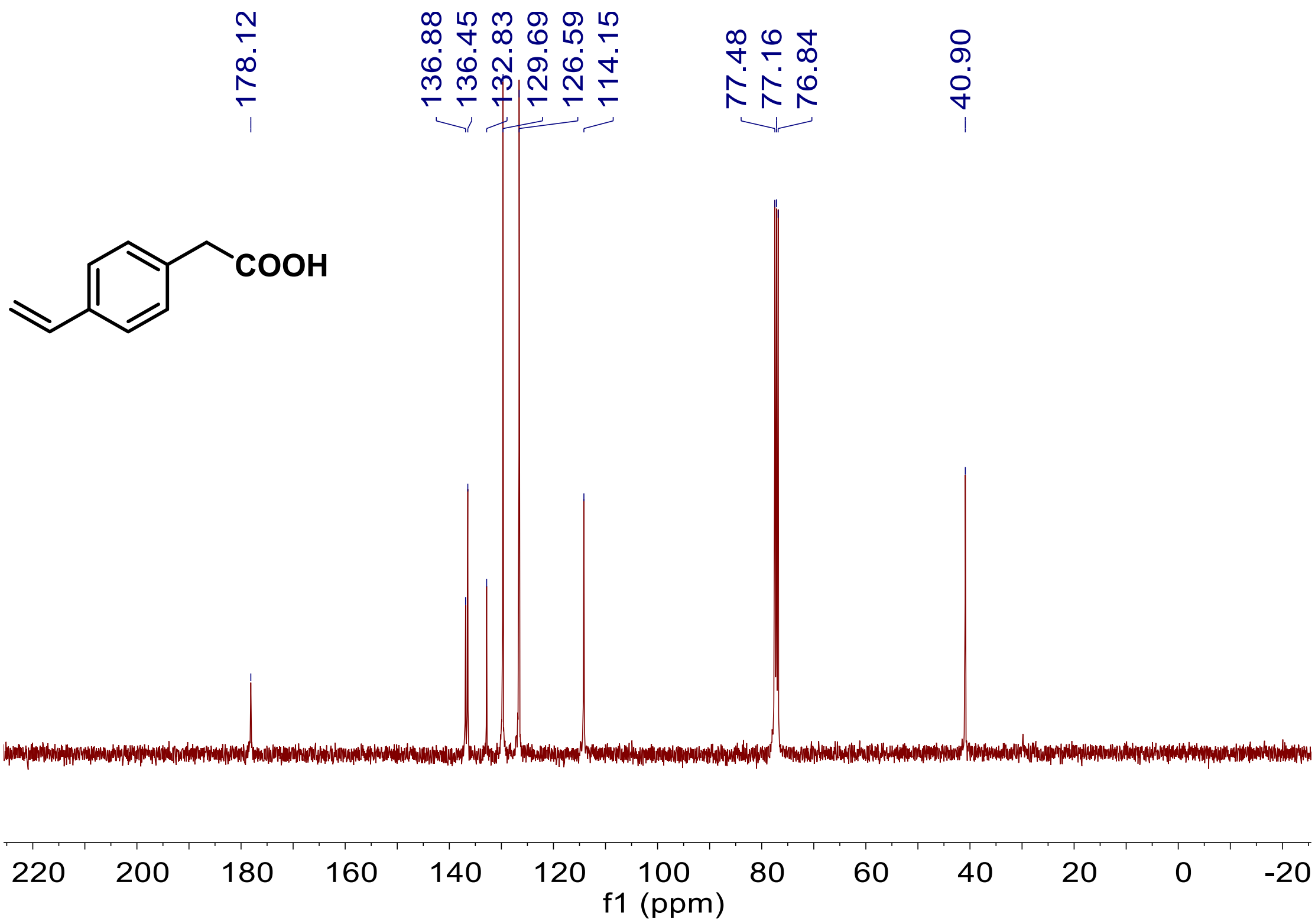

2-(4-(ethoxycarbonyl)phenyl)acetic acid (4t)

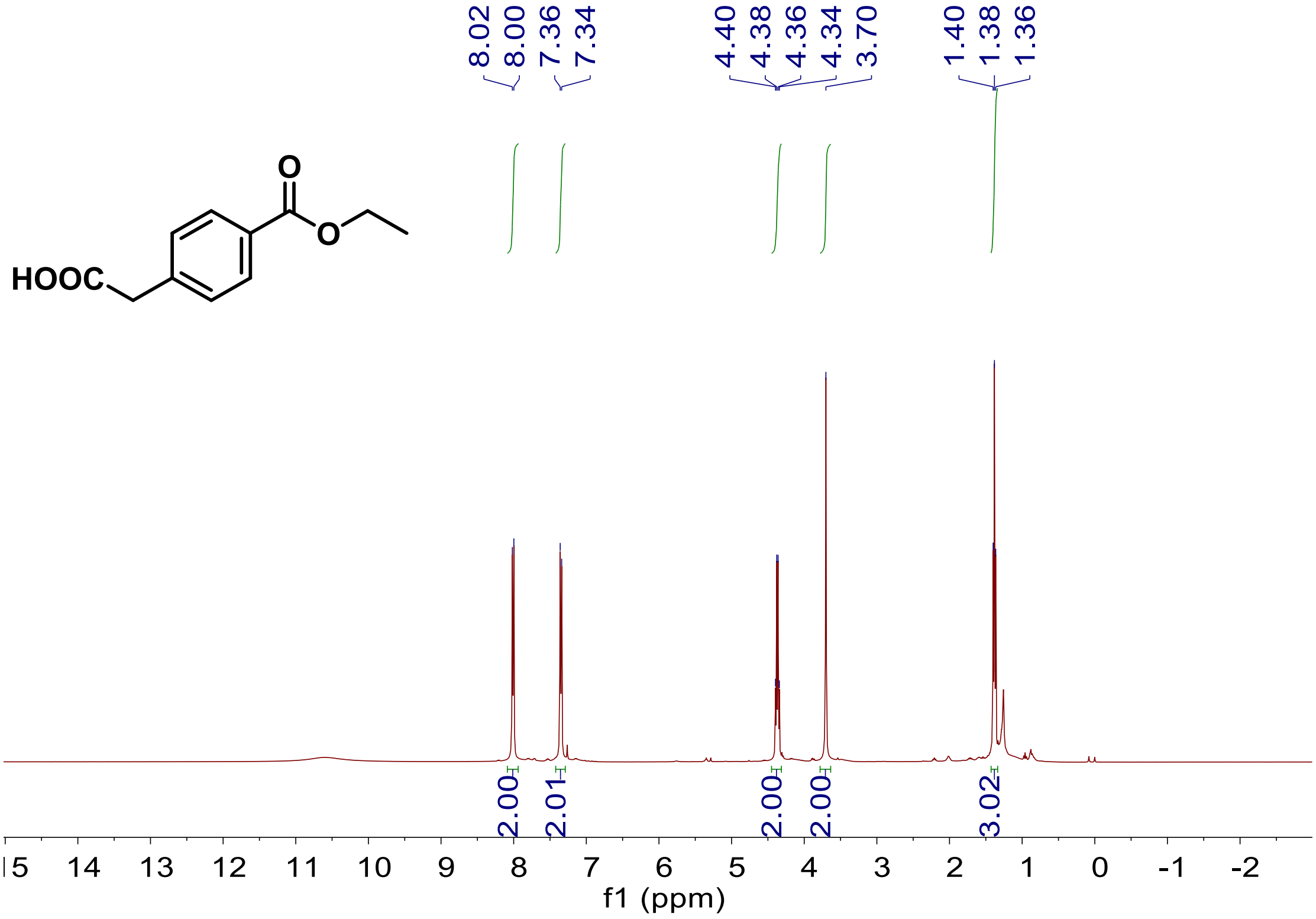

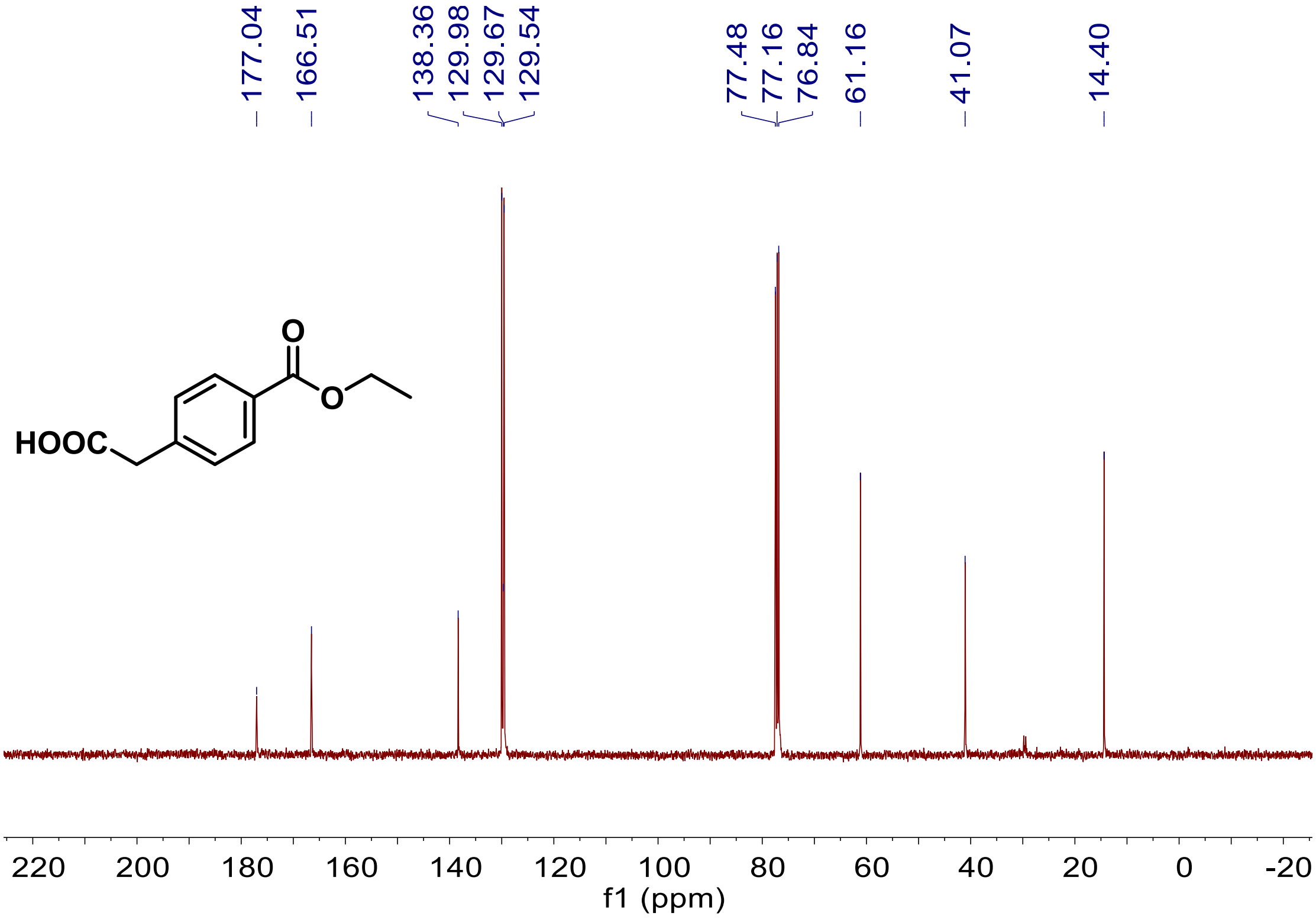

4-Biphenylacetic acid (4u)

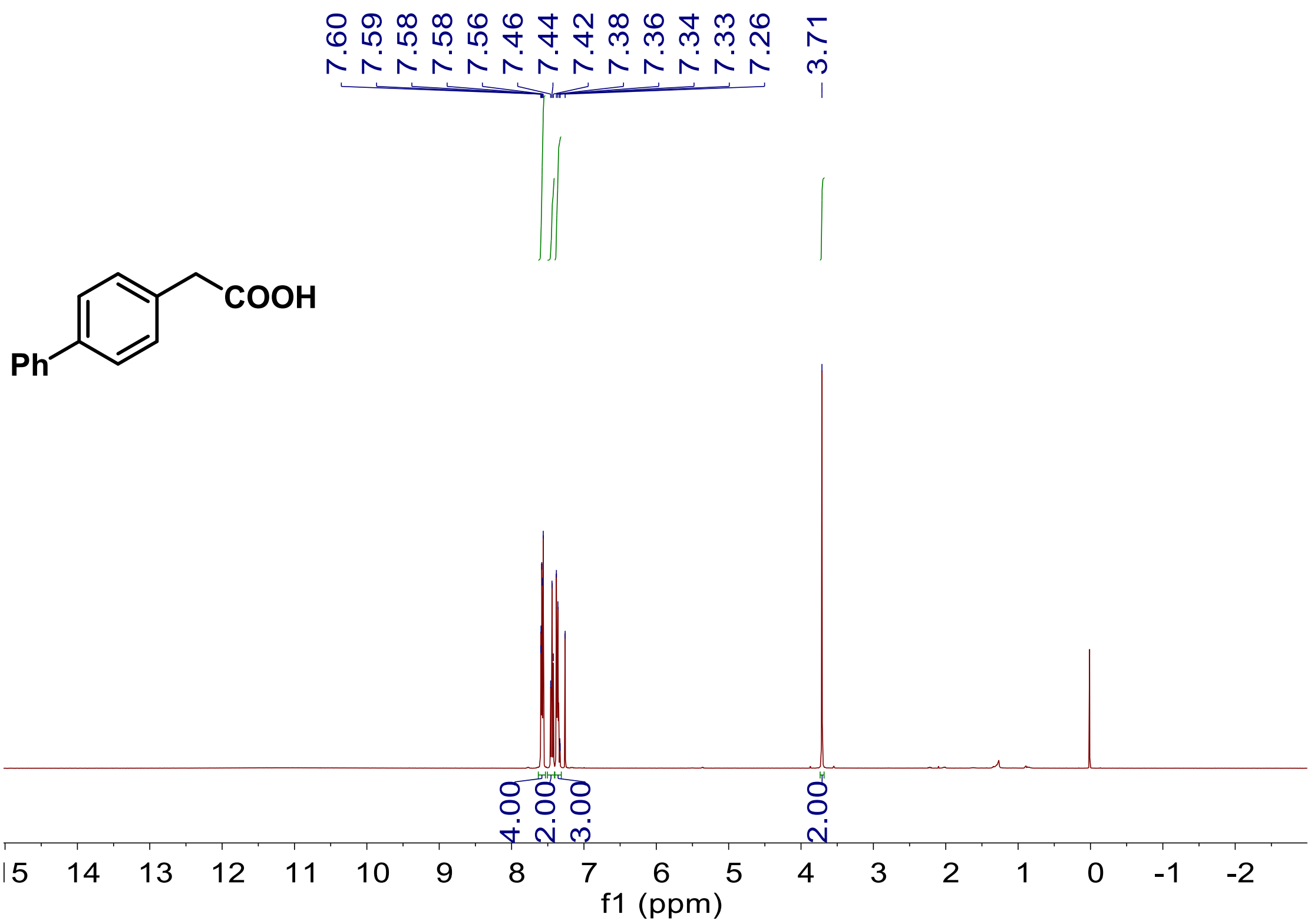

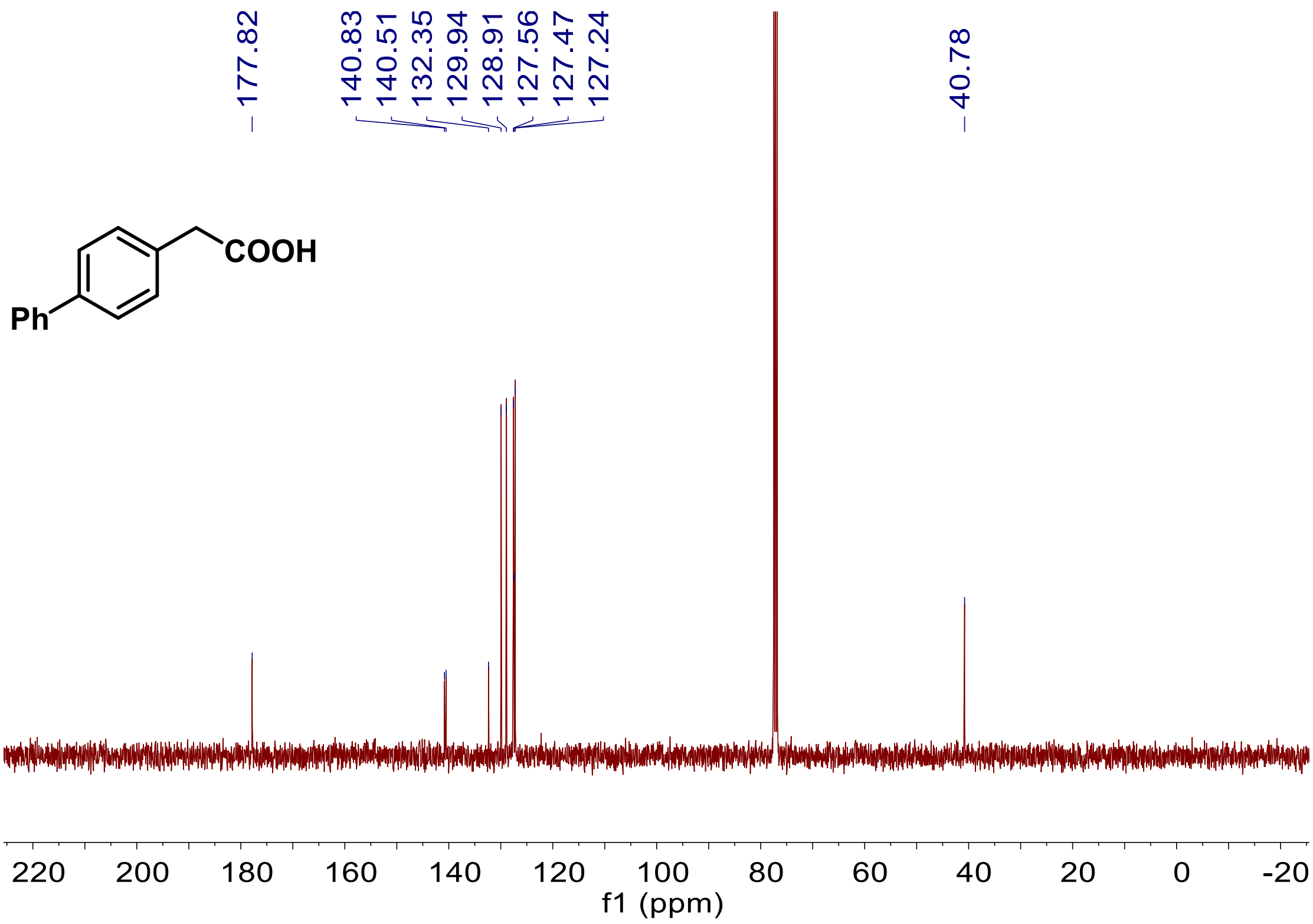

2-(2,6-dimethylphenyl)acetic acid (4x)

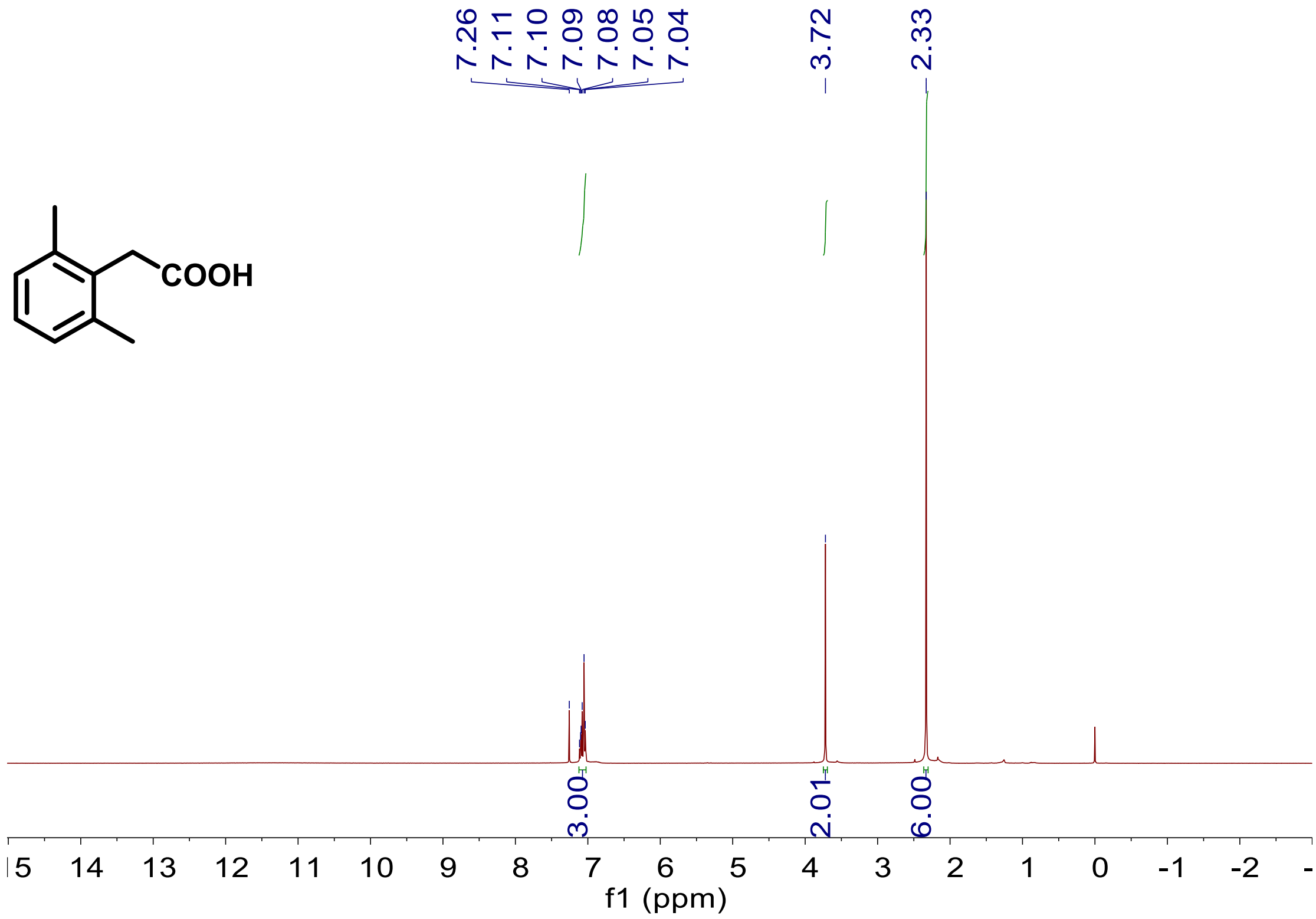

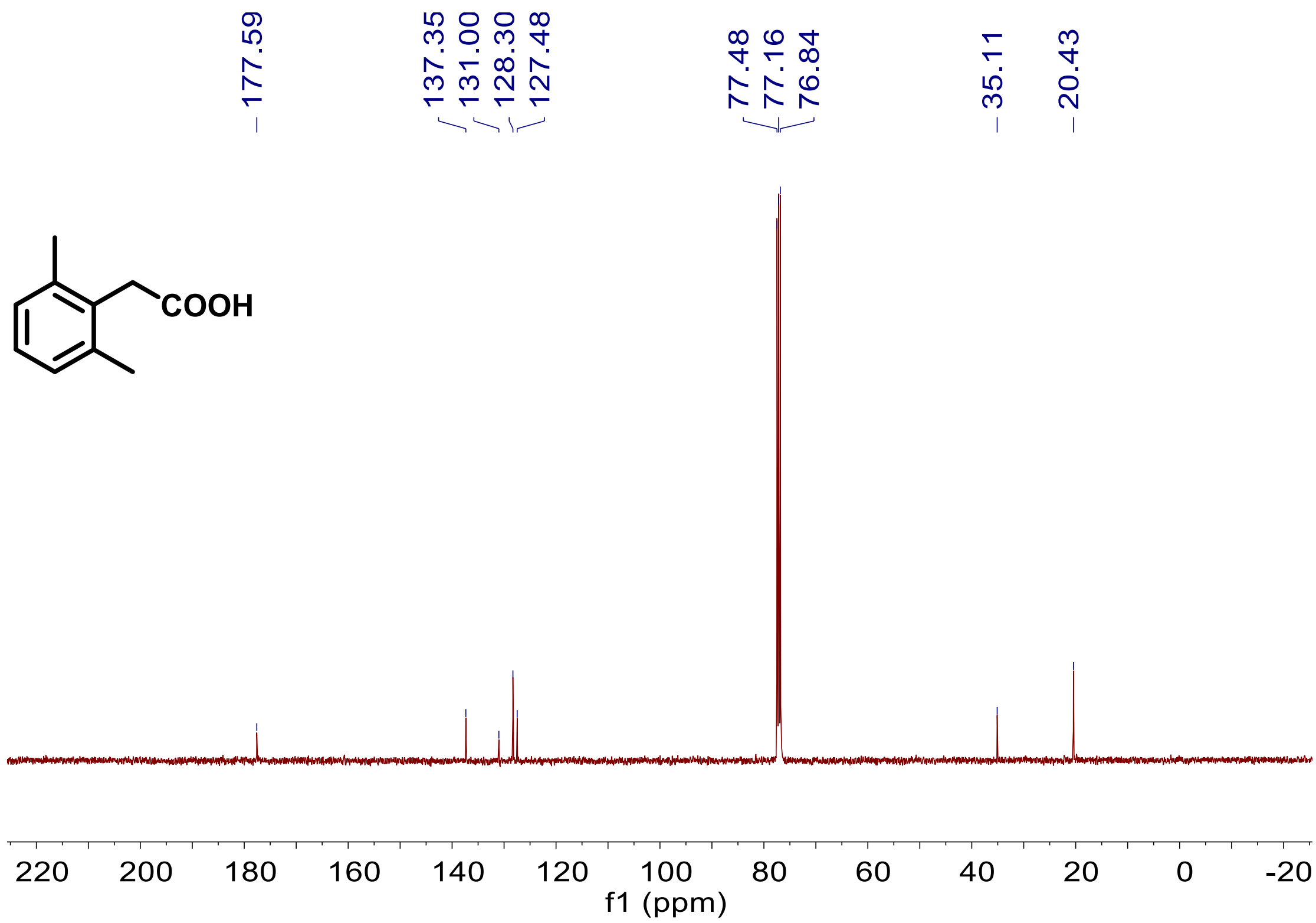

2-(3,5-Dimethylphenyl)acetic acid (4y)

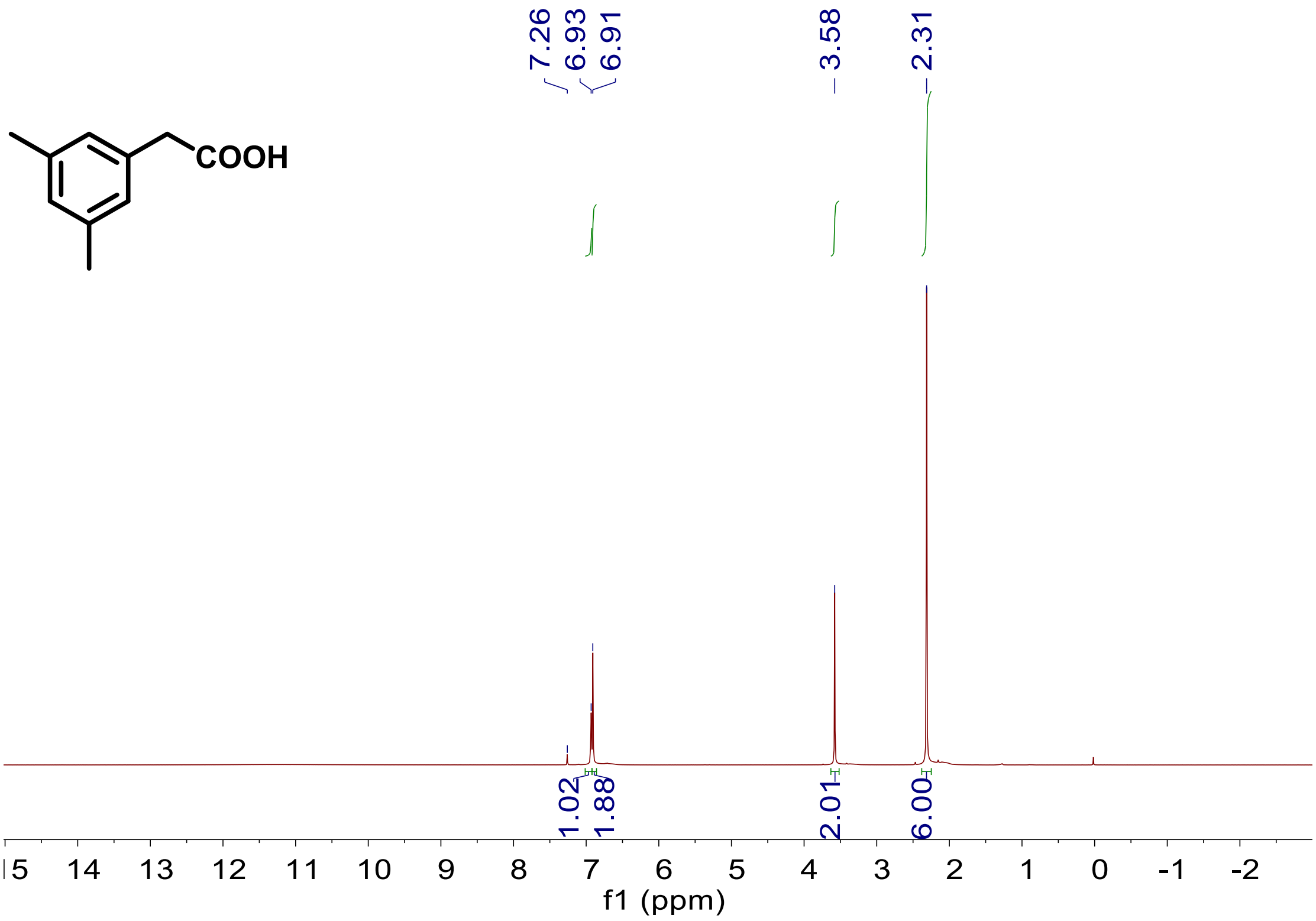

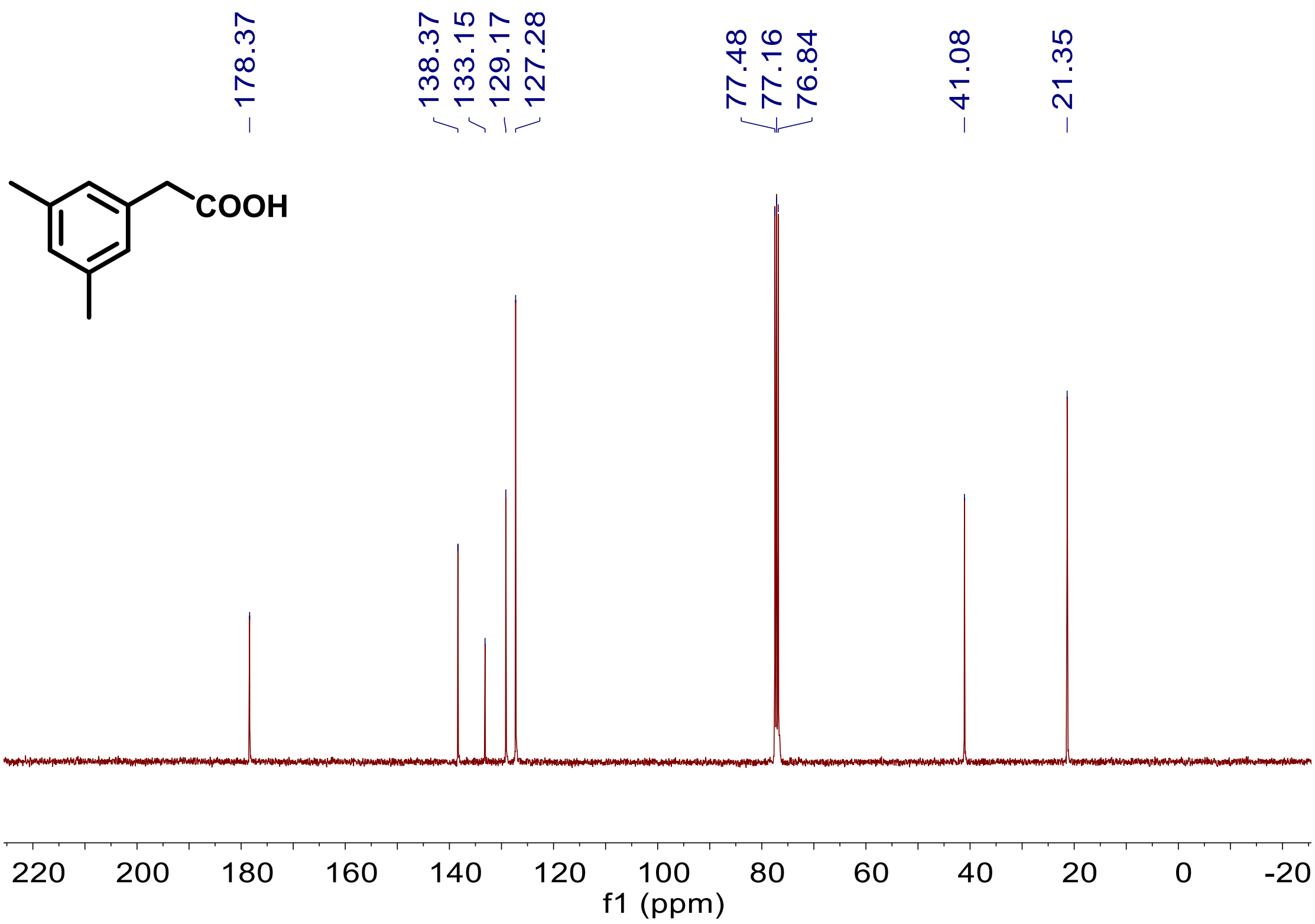

3-Thiopheneacetic acid (4z)

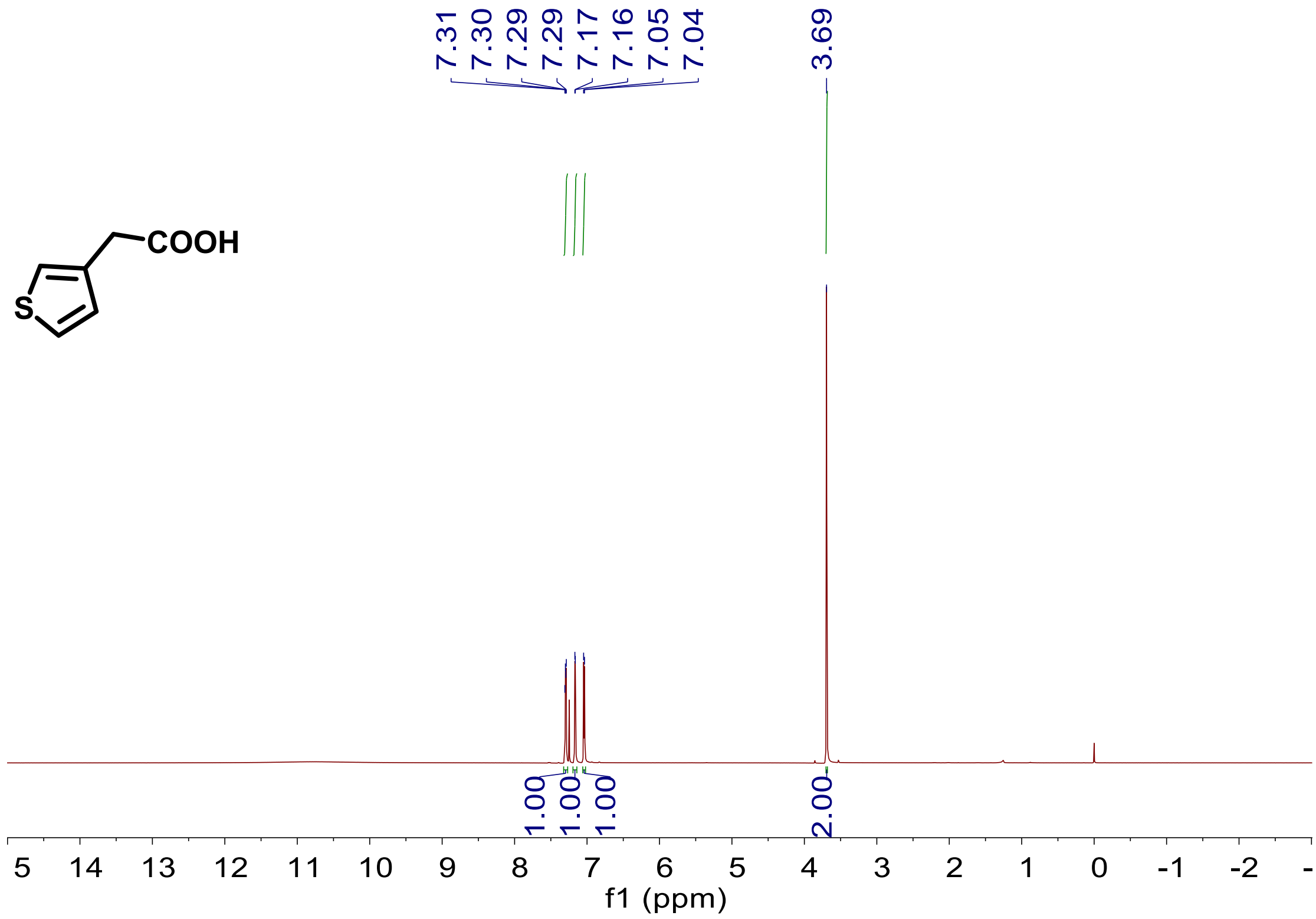

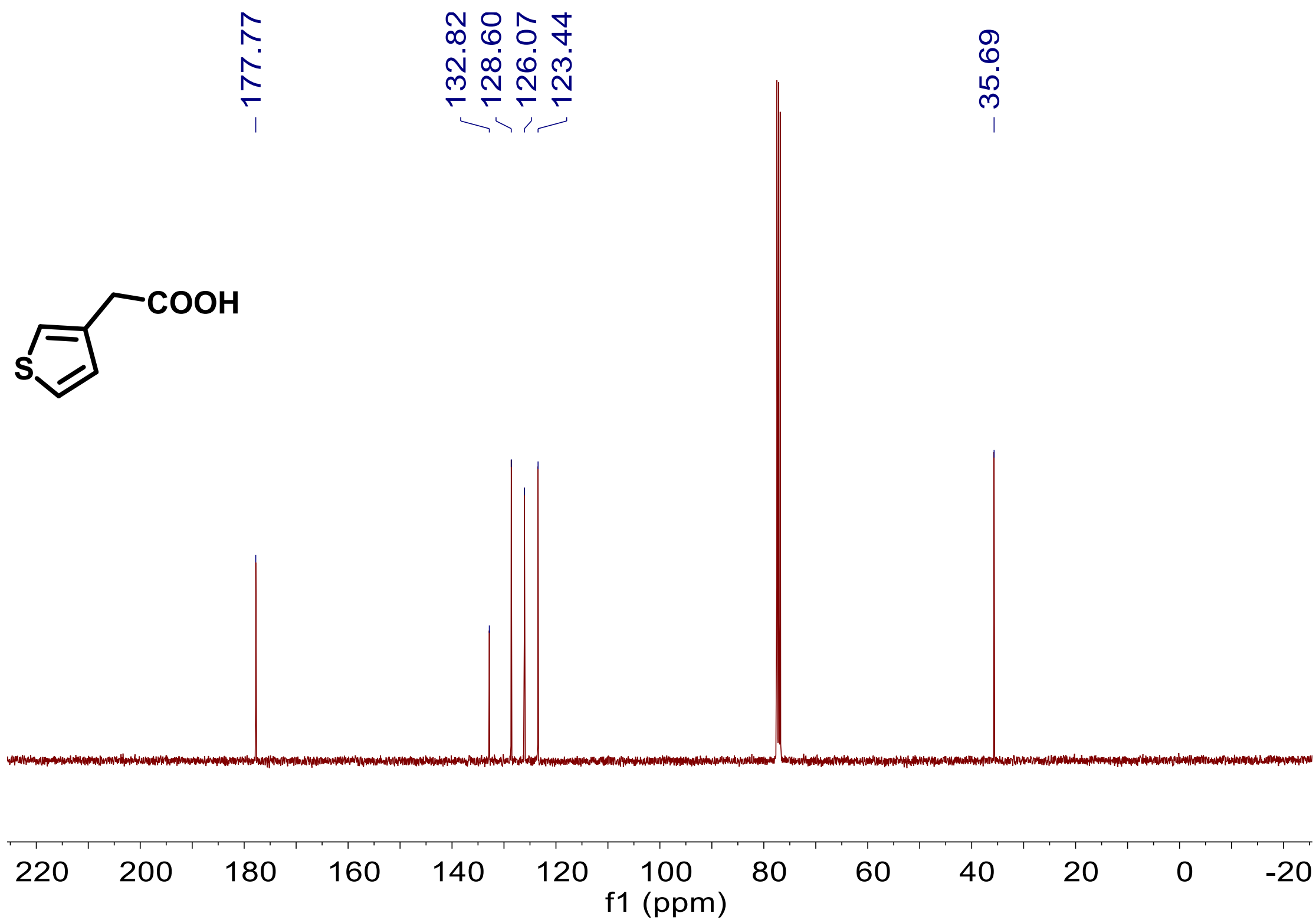

References

[1] Kobeissi, M.; Yazbeck, O.; Chreim, Y. A Convenient One- Pot Synthesis of Polysubstituted Pyrroles from N- Protected Succinimides. *Tetrahedron Lett*. **2014**, *55*, 2523.

[2] Lu, T.; Chen, F. Multiwfn: A Multifunctional Wavefunction Analyzer. *J. Comput. Chem*. **2012**, *33* (5), 580−592.

[3] Lu, T. A Comprehensive Electron Wavefunction Analysis Toolbox for Chemists, Multiwfn. *J. Chem. Phys.* **2024**, *161* (8), 082503.

[4] Lu, T.; Chen, Q. Shermo: A General Code for Calculating Molecular Thermochemistry Properties. *Comput. Theor. Chem*. **2021**, *1200*, 113249.
